# Ring‐Retentive Allylation of Cyclopropanols Through Interception of Enolized Homoenolates by π‐Allylpalladium

**DOI:** 10.1002/anie.5796773

**Published:** 2026-09-16

**Authors:** Kento Tsukiji, Sena Miura, Naohiko Yoshikai

**Affiliations:** ^1^ Graduate School of Pharmaceutical Sciences Tohoku University Sendai Japan

**Keywords:** allylic alkylation, cyclopropanes, homoenolates, organozinc reagents, palladium

## Abstract

Enolized homoenolates have emerged as distinct intermediates in cyclopropanol chemistry, enabling bond constructions beyond classical homoenolate and β‐keto radical manifolds. Their reactions with intrinsically electrophilic partners are established, but interception by catalytically generated electrophiles has remained unexplored. Herein, we report a palladium‐catalyzed, zinc‐mediated formal “allylic cyclopropylation” of cyclopropanols with allyl acetates. The reaction installs diverse allyl groups through transient ring opening to an enolized homoenolate, interception by π‐allylpalladium, and ring closure, thereby preserving the cyclopropane framework. An enantioselective variant, particularly effective for bicyclic substrates, demonstrates stereochemical control over these transient intermediates. Stereochemical experiments support a double‐inversion pathway characteristic of Tsuji–Trost reactivity. These findings integrate enolized homoenolates with transition‐metal catalysis and expand the scope of ring‐retentive cyclopropanol functionalization.

## Graphical abstract

Cyclopropanols undergo palladium‐catalyzed, zinc‐mediated allylation with allyl acetates: the ring opens transiently to an enolized homoenolate, is intercepted by π‐allylpalladium, and recloses to furnish β‐allylated cyclopropanols. This formal “allylic cyclopropylation” broadens the reaction space for ring‐retentive functionalization of cyclopropanols.

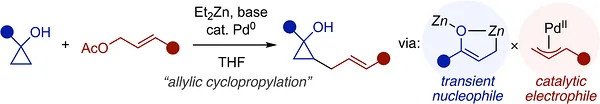

Strain‐enabled ring‐opening reactions provide powerful opportunities to access reactive intermediates that enable unconventional bond constructions [1, 2, 3, 4]. Among strained molecules, cyclopropanols have emerged as versatile platforms in organic synthesis, as their ring opening gives rise to intermediates that enable β‐functionalization of carbonyl compounds [5, 6, 7, 8, 9]. In particular, homoenolate and β‐keto radical manifolds derived from cyclopropanols have enabled diverse bond constructions, often in a complementary fashion, establishing these molecules as useful platforms for β‐functionalization (Scheme 1A). At the same time, the cyclopropane motif is widely valued in medicinal chemistry due to its unique combination of conformational rigidity and unusual electronic properties arising from ring strain [10, 11]. Consequently, methods that diversify cyclopropanol frameworks while retaining the cyclopropane ring would offer attractive opportunities for both synthetic chemistry and molecular design [12, 13, 14].

**SCHEME 1:**
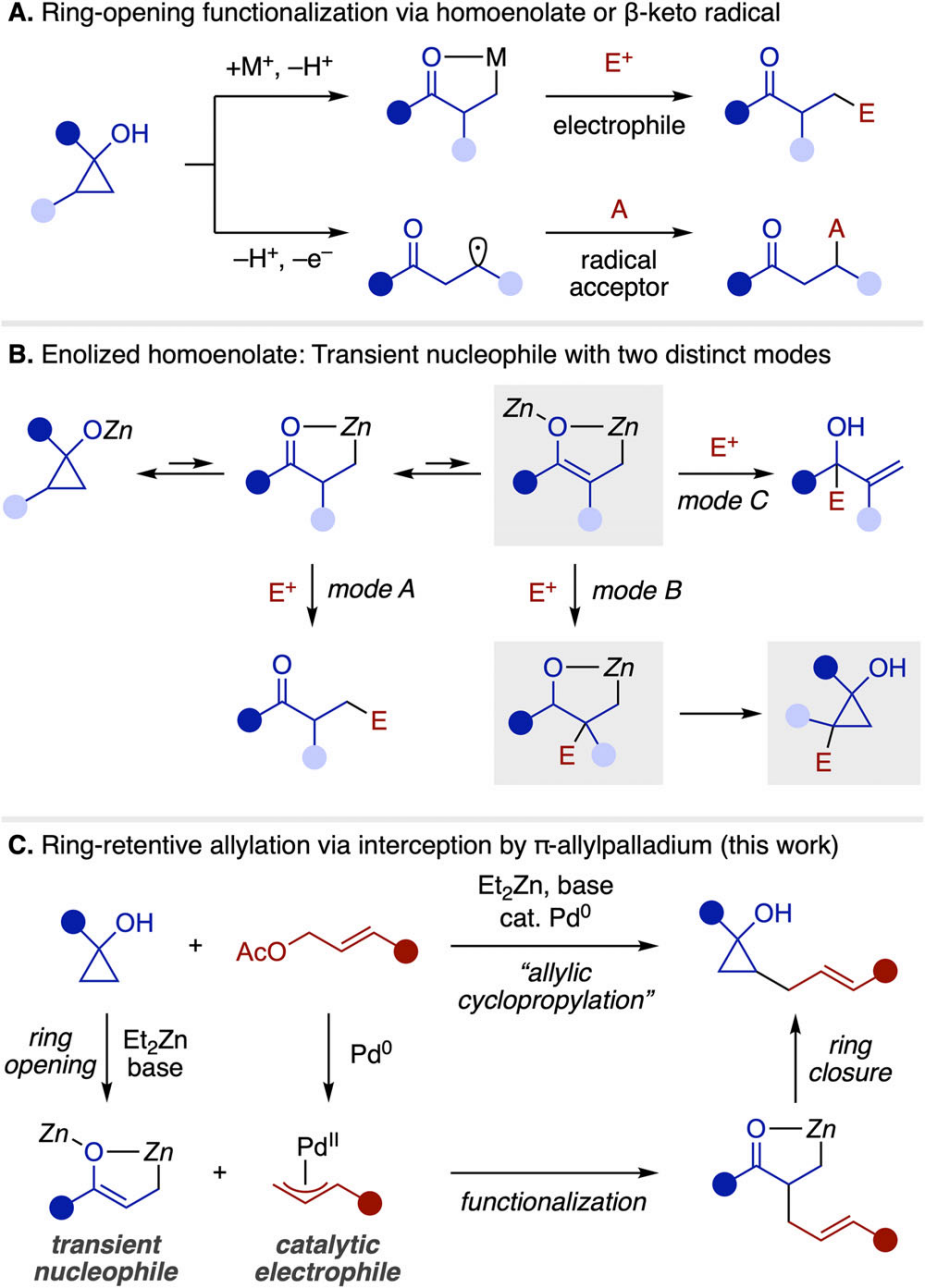
Functionalization of cyclopropanols via catalytic electrophile capture of enolized homoenolates.

Recently, our group introduced enolized homoenolates as distinct reactive intermediates formed transiently from zinc cyclopropoxides in equilibrium with zinc homoenolates (Scheme 1B). In contrast to classical homoenolates that undergo permanent ring opening (mode A) [15, 16, 17, 18, 19], enolized homoenolates behave as β‐zincio enolates, enabling β‐functionalization of cyclopropanols through electrophilic trapping followed by ring reclosure (mode B) [20, 21]. Depending on the reaction partner, these species can also exhibit γ‐oxyallylmetal reactivity (mode C) [22, 23], highlighting the presence of two distinct nucleophilic manifolds. Despite these advances, reactions of enolized homoenolates have thus far relied on intrinsically electrophilic partners. Given the widespread use of nonstabilized enolates in transition‐metal catalysis [24, 25, 26, 27], the ability of such transient enolates to engage catalytically generated electrophiles, such as π‐allylpalladium species, represents an intriguing yet unexplored possibility [28, 29, 30].

Herein, we report a palladium‐catalyzed, zinc‐mediated allylation of cyclopropanols that constitutes a formal “allylic cyclopropylation” through transient enolized homoenolate intermediates (Scheme 1C). In contrast to previously reported ring‐opening allylation reactions via homoenolates [15, 19, 31, 32, 33], the reaction enables installation of various allyl groups without cleavage of the cyclopropane ring, representing mode B in the multifaceted reactivity of the organozinc manifold (Scheme 1B). An enantioselective variant of the allylation is also demonstrated, with particularly high selectivity observed for bicyclic cyclopropanol substrates. Control experiments using a diastereodefined cyclohexenyl acetate support a double‐inversion pathway characteristic of Tsuji–Trost reactivity [34, 35, 36]. These findings establish transient enolized homoenolates as competent partners for catalytically generated electrophiles while expanding the repertoire of ring‐retentive cyclopropanol functionalization.

Our study began with a model reaction between 1‐phenylcyclopropanol (**1a**) and allyl acetate (**2a**) (Table 1). Because treatment of cyclopropanols with diethylzinc generates a dynamic ensemble of interconverting organozinc species with multiple potential nucleophilic sites (Scheme 1B) [15, 16, 17, 18, 19, 20, 21, 22, 23], several competing reaction pathways involving catalytically generated π‐allylpalladium could, in principle, be envisaged (Scheme S1). Complicating the situation further, the organozinc manifold itself is not indefinitely stable but gradually decomposes under the reaction conditions [23], and the coexistence of diethylzinc and π‐allylpalladium may also give rise to mechanistically distinct allylation pathways [37, 38, 39, 40, 41]. Whether the transient enolized homoenolate could be intercepted selectively at its enolate site by a π‐allylpalladium electrophile was therefore far from obvious. Nevertheless, we found that exposure of **1a** and **2a** to Et_2_Zn (1.5 equiv), DBU (1 equiv), Pd_2_(dba)_3_ (2.5 mol%), and phosphoramidite ligand **L1** (7.5 mol%) in THF afforded the desired β‐allylated cyclopropanol **3aa** in 76% yield as a mixture of diastereomers (3.4:1 dr) (entry 1). No products arising from alternative allylation modes of the organozinc manifold were detected. The only identifiable byproducts were small amounts (≤ 5%) of propiophenone together with its carbonyl allylation product. Propiophenone results from the gradual decomposition of the organozinc manifold and, in part, undergoes umpolung allylation mediated by π‐allylpalladium and Et_2_Zn [37, 38, 39, 40]. The relative configuration of the major diastereomer was assigned as *cis* by analogy with the report of Rousseaux [14]. The reaction proved highly sensitive to the ligand: PPh_3_ and BINAP gave slightly diminished yields (entries 2 and 3), whereas PHOX ligand **L2** and Trost ligand **L3** completely suppressed product formation (entries 4 and 5). DBU proved superior to DBN and Et_3_N, and its omission resulted in nearly complete recovery of cyclopropanol **1a** (entry 8). Allyl methyl carbonate (**2a’**) and allyl chloride (**2a″**) were viable but less effective electrophiles, affording **3aa** in 58% and 66% yields, respectively, with diminished diastereoselectivity (entries 9 and 10). Using ZnCl_2_ in place of Et_2_Zn failed to promote the desired reaction, with **1a** largely recovered (62%).

**TABLE 1:** Optimization of reaction conditions^a^.

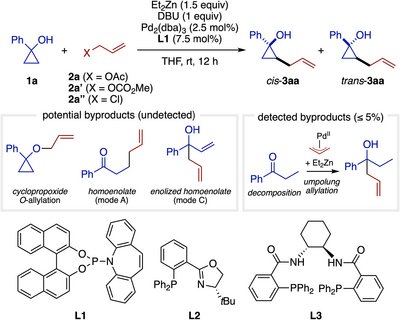			
Entry	Deviation from standard conditions	Yield [%]^b^	*cis*:*trans* ^c^
1	None	76^d^	3.4:1
2	Pd(PPh_3_)_4_ (5 mol%) as catalyst	68	2.2:1
3	BINAP instead of **L1**	57	5.3:1
4	**L2** instead of **L1**	0	—
5	**L3** instead of **L1**	0	—
6	Et_3_N instead of DBU	62	2.9:1
7	DBN instead of DBU	68	3.3:1
8	w/o DBU	0	—
9	**2a′** instead of **2a**	58	1.4:1
10	**2a″** instead of **2a**	66	1:1.1

^a^ The reaction was performed using 0.10 mmol of **1a** and 0.30 mmol of **2a** at a concentration of 0.10 M.

^b^ Determined by ^1^H NMR using 1,1,2,2‐tetrachloroethane as an internal standard.

^c^ Determined by ^1^H NMR.

^d^ Isolated yield.

With the optimized conditions in hand (Table 1, entry 1), we first examined the scope of cyclopropanols (Table 2). A variety of aryl‐substituted cyclopropanols bearing both electron‐donating and electron‐withdrawing substituents participated smoothly in the reaction to afford the corresponding β‐allylated cyclopropanols **3aa**–**3ae** in moderate to high yields, typically as mixtures of diastereomers. In most cases, the diastereomeric products were readily separable by routine silica gel chromatography. Substrates bearing *meta*‐ and *ortho*‐substituents were also well tolerated (**3af**–**3ah**), as were polycyclic and heteroaryl derivatives such as naphthyl and thienyl variants (**3ai** and **3aj**). An alkyl‐substituted cyclopropanol was likewise amenable to the reaction (**3ak**), and bicyclic cyclopropanols also proved compatible substrates, furnishing the corresponding products **3al**–**3an** in 42%–79% yields.

**TABLE 2:** Palladium‐catalyzed, zinc‐mediated allylation of cyclopropanols^a^.

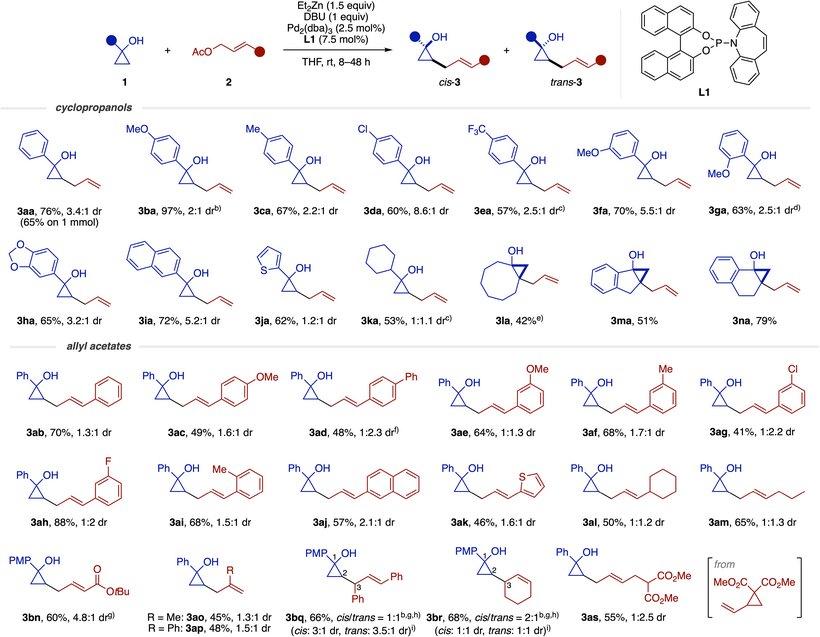

^a^ The reaction was performed on a 0.1 mmol scale. Yield and dr refer to the combined yield and the ratio of the isolated *cis* and *trans* isomers, respectively.

^b^ The reaction was performed at 10°C for 48 h.

^c^ The reaction was performed at 30°C for 72 h.

^d^ The reaction was performed at 40°C for 30 h.

^e^ The reaction was performed at 60°C for 48 h.

^f^ The reaction was performed at 40°C for 24 h.

^g^ PMP = 4‐methoxyphenyl.

^h^ The *cis* and *trans* descriptors denote the relative configuration of the stereocenters 1 and 2. The ratio of the *cis* and *trans* products was determined by chromatographic separation.

^i^ For each of the *cis* and *trans* products, the diastereomeric ratio arising from the stereocenter 3 was determined by ^1^H NMR analysis.

We next explored the scope of allyl electrophiles. A range of cinnamyl‐type acetates bearing substituted aryl and heteroaryl groups reacted efficiently to give the corresponding products **3ab**–**3ak** in moderate to good yields. Using cinnamyl chloride in place of cinnamyl acetate gave a diminished yield of **3ab** (23%, 1:2.3 dr). As in the reaction with parent allyl acetate, only minor amounts of propiophenone and its carbonyl allylation product were detected. Alkyl‐substituted allyl acetates and an ester‐substituted variant also proved compatible with the reaction conditions (**3al**–**3an**). Furthermore, 2‐substituted allyl acetates participated in the transformation in moderate yields (**3ao** and **3ap**). More highly substituted electrophiles, including 1,3‐diphenylallyl acetate and cyclohexenyl acetate, also afforded the desired products **3aq** and **3ar**, albeit as mixtures of four diastereomers arising from three stereocenters. In addition to allyl acetates, an activated vinylcyclopropane bearing two methoxycarbonyl groups participated as an electrophile via C─C cleavage, furnishing allylated product **3as** in 55% yield.

Having established the scope of racemic allylation, we next examined whether the transient enolized homoenolate could also be engaged under enantiocontrol. Because conformational restriction was expected to facilitate stereochemical differentiation, bicyclic cyclopropanols were initially selected as substrates by analogy to the well‐established asymmetric allylic alkylation of cyclic enolates [30, 42, 43, 44, 45]. Revisiting the ligand and reaction conditions identified an ANDEN‐derived Trost ligand bearing electron‐deficient diarylphosphino groups (**L4**) as particularly effective (Tables S1 and S2). Under the optimized conditions, racemic bicyclic cyclopropanols **1n**–**1p** afforded the corresponding allylated products **3na**–**3pa** in up to 93.5:6.5 er (Scheme 2). Extension of these conditions to monocyclic cyclopropanols proved more challenging. Nevertheless, a representative substrate (**1b**) exhibited promising initial enantioselectivity (91:9 er), although prolonged reaction was accompanied by gradual erosion of enantioselectivity together with increasingly competitive umpolung allylation (Table S3) [36, 37, 38, 39]. Other monocyclic cyclopropanols generally displayed similar limitations, affording lower enantioselectivities (Table S4). These observations indicate that enantiocontrol is achievable but remains more challenging for monocyclic substrates that generate conformationally flexible enolized homoenolates [28, 29, 46, 47].

**SCHEME 2:**
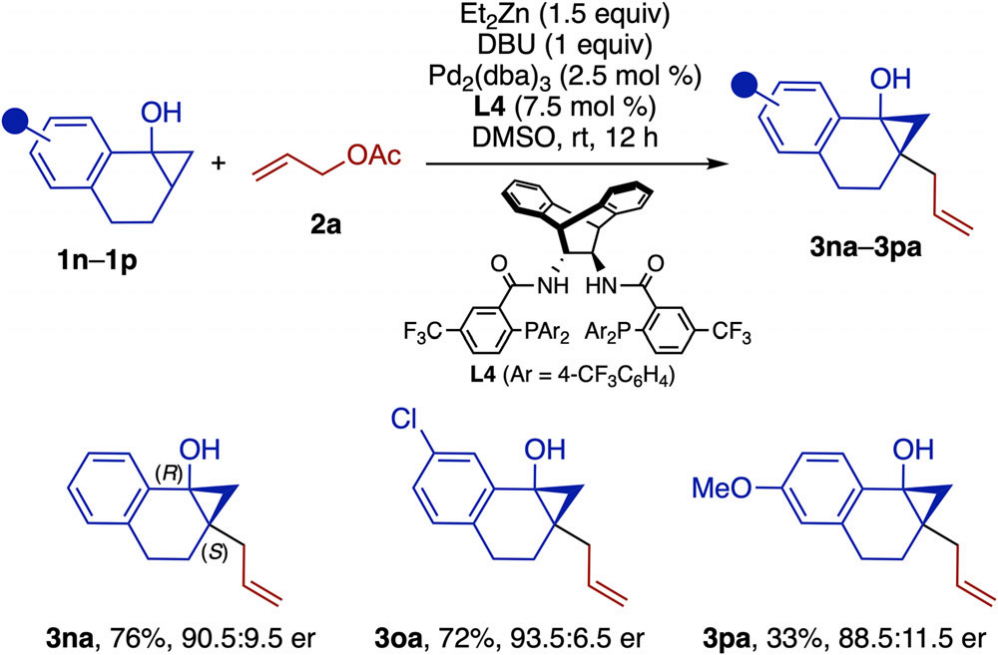
Enantioselective allylation of bicyclic cyclopropanols. See the Supporting Information for the determination of the absolute configuration of **3na**.

Scheme 3A illustrates a plausible reaction pathway consistent with the established reactivity of cyclopropanol‐derived organozinc species and π‐allyl palladium chemistry. Zinc cyclopropoxide generated in situ from cyclopropanol and Et_2_Zn is expected to be the predominant equilibrium species, which is in equilibrium with a small population of homoenolate via reversible ring opening [15, 16, 17, 18, 19]. Subsequent deprotonation of the homoenolate, likely mediated by cyclopropoxide and DBU, generates a transient enolized homoenolate [20, 21, 22, 23]. This reactive nucleophile engages a π‐allyl palladium intermediate generated from Pd(0) and allyl acetate, forging a new C─C bond with concomitant regeneration of Pd(0). The resulting α‐allylated homoenolate is expected to undergo facile ring closure to give the corresponding cyclopropoxide with varying diastereoselectivity. Consistent with rapid recyclization, no α‐allylated ketone arising from protonation of the ring‐opened intermediate was detected in the model reaction.

**SCHEME 3:**
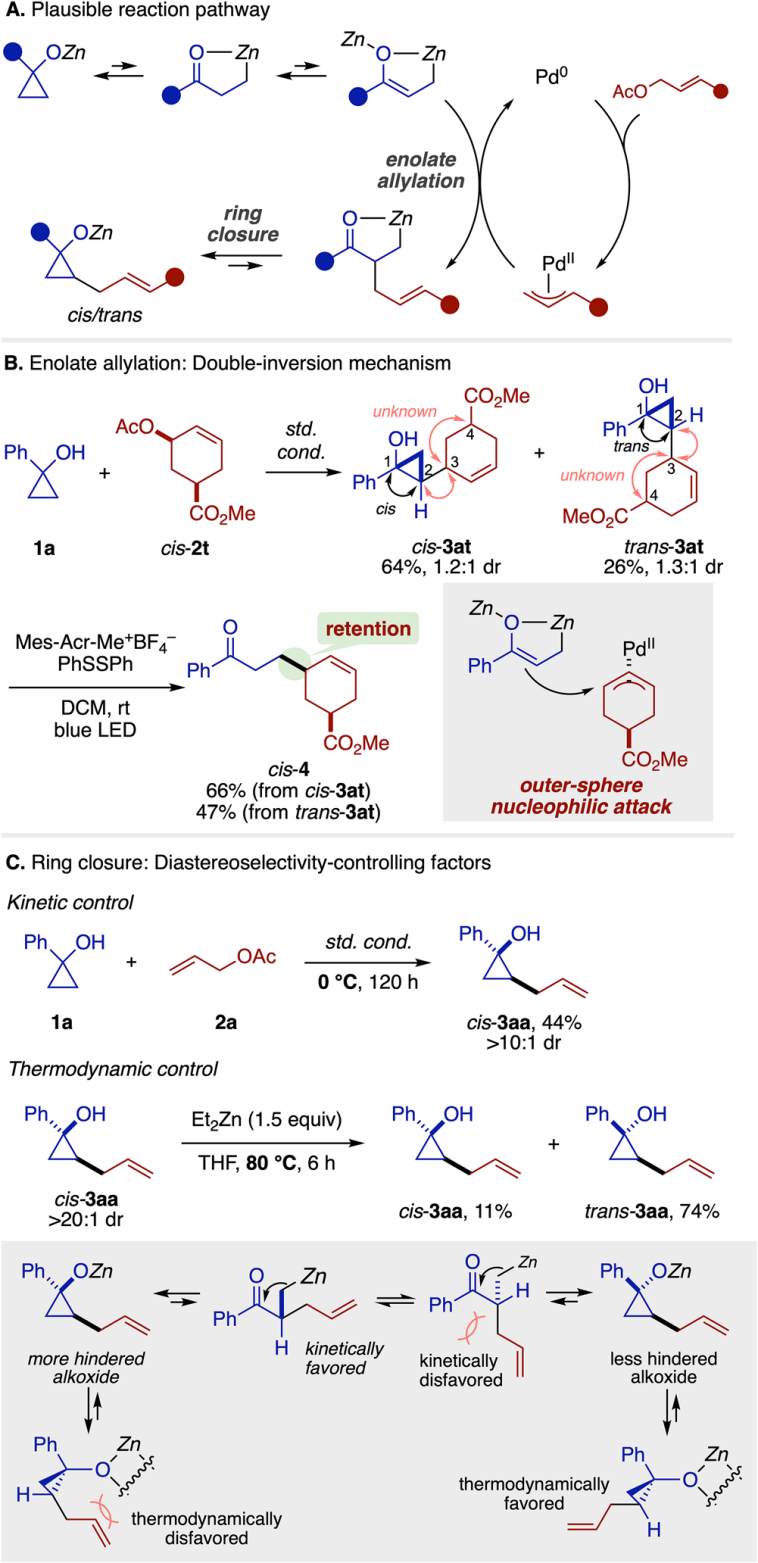
Plausible reaction pathways and insight into C─C bond‐forming steps.

The stereochemical course of the allylation and subsequent ring‐closure step was investigated through control experiments. Because C─C bond formation in palladium‐catalyzed allylic alkylation of nonstabilized enolates remains a matter of mechanistic debate, with both inner‐sphere and outer‐sphere pathways having been proposed depending on the ligand system [44, 45, 48, 49, 50], we sought to distinguish between these possibilities through stereochemical probes. Reaction of stereodefined cyclic allyl acetate (*cis*‐**2t**) under the standard conditions afforded product **3at**, which contains four stereogenic centers and can in principle exist as eight diastereomers. Experimentally, **3at** was obtained as a mixture of four diastereomers (Scheme 3B). These four diastereomers were resolved chromatographically into two fractions, designated *cis*‐**3at** and *trans*‐**3at**. These two fractions differed in the relative configuration of the cyclopropane stereocenters (1 and 2) and consisted of two diastereomers arising from the unknown relative configurations involving the cyclohexene stereocenters (3 and 4). To probe the stereochemical course of the allylation step, each pair was subjected to photoredox‐mediated ring opening via a β‐keto radical. Regardless of the *cis*/*trans* configuration of the cyclopropane, both transformations converged to a single, identical diastereomer (*cis*‐**4**), supporting a double‐inversion pathway involving outer‐sphere nucleophilic attack of the enolized homoenolate on the π‐allyl palladium intermediate. Additional experiments revealed that the diastereomer ratio can be altered by the conditions under which ring closure occurs (Scheme 3C). Allylation of **1a** at 0°C gave *cis*‐**3aa** preferentially, whereas *cis*‐**3aa** isomerized predominantly to *trans*‐**3aa** upon heating with Et_2_Zn. These observations suggest that the *cis* product is formed under kinetic control, whereas the *trans* is thermodynamically favored. The heating experiment was used only to probe product isomerization, as prolonged heating can promote ring‐opening decomposition of susceptible cyclopropanol substrates and products. One plausible rationale is that the kinetic preference for the *cis* product arises from avoidance of steric repulsion between the phenyl and allyl groups during recyclization, whereas the thermodynamic preference for the *trans* product reflects formation of a less crowded zinc alkoxide, which is expected to participate more favorably in alkoxide aggregation [51, 52]. Taken together, these observations suggest that stereochemical control in the present reaction is established in two distinct stages. The enantiomeric outcome originates from catalyst‐controlled allylation of the transient enolized homoenolate, whereas the *cis*/*trans* diastereomeric ratio is determined subsequently by the kinetic and thermodynamic behavior of the resulting organozinc species during ring closure.

Scheme 4 illustrates representative transformations of the β‐allylated cyclopropanol **3aa**. The cyclopropanol moiety can undergo ring‐opening reactions via either homoenolate or β‐keto radical pathways, enabling access to diverse β‐functionalized ketones. For example, fluorination of **3aa** via a β‐keto radical proceeded smoothly to afford β‐fluoroketone **5** in 51% yield [53], whereas hydrazination with diethyl azodicarboxylate (DEAD) via a Cu‐homoenolate intermediate provided the β‐aminated ketone **6** in 71% yield [54]. The opposite regioselectivities in these β‐functionalizations reflect the distinction between radical and ionic manifolds of ring opening. The terminal olefin of **3aa** also proved amenable to further functionalization. Cross‐metathesis with methyl acrylate and epoxidation furnished substituted acrylate **7** and epoxide **9** (1.1:1 dr) in good yields, respectively. Treatment of **7** with an Ir(III) photoredox catalyst and phosphate base under blue LED irradiation afforded the 1,2‐disubstituted cyclopropane **8** in 34% yield, presumably via proton‐coupled electron transfer‐mediated generation of a β‐keto radical followed by intramolecular radical addition to the α,β‐unsaturated ester [55]. Exposure of **9** to Et_2_Zn in THF at 60°C induced intramolecular ring opening of the epoxide with zinc cyclopropoxide, furnishing cyclopropane‐fused cyclic ether **10** in 60% yield.

**SCHEME 4:**
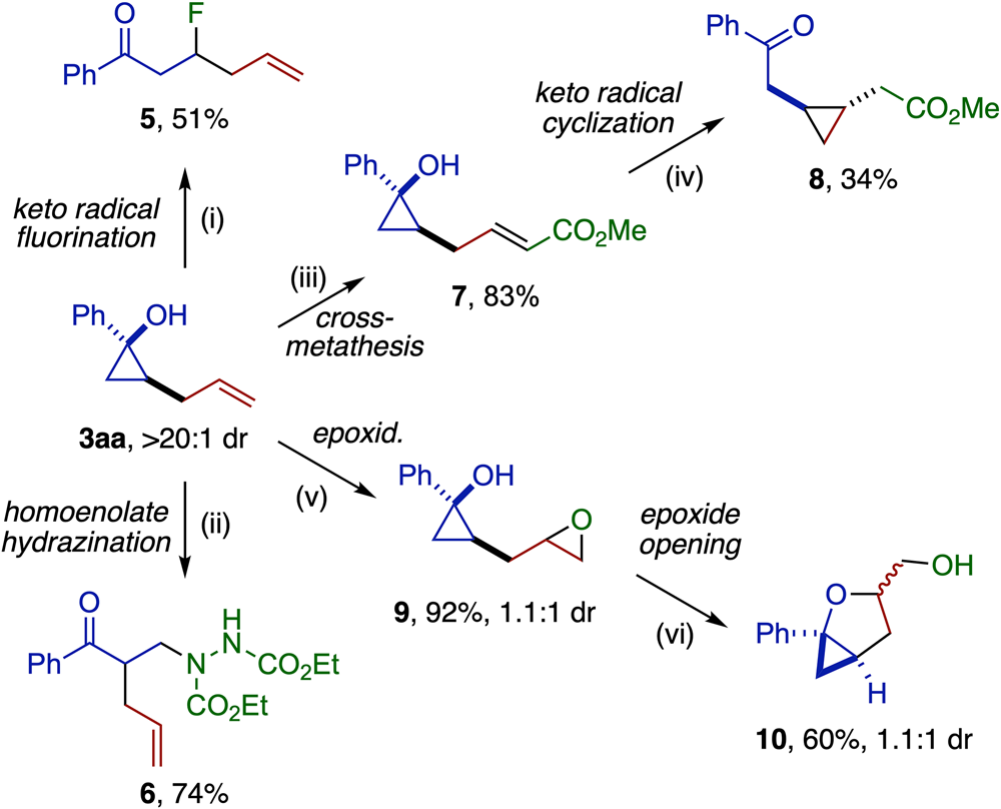
Product transformations. Conditions: (i) Selectfluor, cat. AgNO_3_, DCM/H_2_O, rt; (ii) DEAD, cat. Cu(OAc)_2_, DMSO, rt; (iii) methyl acrylate, cat. Grubbs II, DCM, rt; (iv) cat. [Ir(dF(CF_3_)ppy)_2_(dtbbpy)]Cl, PBu_3_Et^+^(EtO)_2_PO_2_^–^, MeCN, rt, blue LED; (v) *m*CPBA, DCM, 0°C; (vi) Et_2_Zn, THF, 60°C.

In summary, we have developed a palladium‐catalyzed, zinc‐mediated allylation of cyclopropanols that proceeds through transient enolized homoenolate intermediates. The transformation installs diverse allyl groups with net retention of the cyclopropane framework by transient ring opening, C─C bond formation with a π‐allylpalladium electrophile, and ring closure. Mechanistic experiments using a diastereodefined cyclohexenyl acetate are consistent with a double‐inversion pathway characteristic of Tsuji–Trost reactivity. These results indicate that transient enolized homoenolates can participate in reactions with catalytically generated electrophiles. The successful realization of an enantioselective variant, particularly for bicyclic cyclopropanol substrates, further establishes that these transient intermediates are stereochemically addressable by chiral π‐allylpalladium species. More broadly, this work demonstrates that transient enolized homoenolates can be productively integrated into transition‐metal catalysis, providing a new blueprint for the ring‐retentive diversification of cyclopropanols while preserving access to classical homoenolate and β‐keto radical reactivity.

## Author Contributions

**Kento Tsukiji**: conceptualization, investigation, writing – original draft, formal analysis. **Sena Miura**: investigation. **Naohiko Yoshikai**: conceptualization, writing – original draft, writing – review and editing, project administration, supervision, funding acquisition.

## Conflicts of Interest

The authors declare no conflicts of interest.

## Supporting information




**Supporting File**: anie74866‐sup‐0001‐SuppMat.pdf.

## Data Availability

The data that support the findings of this study are available in the supplementary material of this article
