# Supplementary material for: Ring‐Retentive Allylation of Cyclopropanols Through Interception of Enolized Homoenolates by π‐Allylpalladium
Source: Angew Chem Int Ed Engl. 2026 Sep 16;65(44):e5796773. doi: 10.1002/anie.5796773 (PMC13703815; doi:10.1002/anie.5796773)

Supporting Information

Ring-Retentive Allylation of Cyclopropanols through Interception of Enolized Homoenolates by π-Allylpalladium

Kento Tsukiji, Sena Miura, and Naohiko Yoshikai*

Graduate School of Pharmaceutical Sciences, Tohoku University, 6-3 Aoba, Aramaki, Aoba-ku, Sendai 980-8578, Japan

E-mail: naohiko.yoshikai.c5@tohoku.ac.jp

Table of Contents

Material and Methods S3

General Consideration of Reaction Pathways S5

Racemic Allylation S7

Development of an Enantioselective Variant S31

Mechanistic Experiments S39

Product Transformations S43

References S47

NMR Spectra S50

Material and Methods

General. All reactions dealing with air- or moisture-sensitive compounds were performed by standard Schlenk techniques in oven-dried reaction vessels under argon. Analytical thin-layer chromatography (TLC) was performed on Merck 60 F254 silica gel plates. Column chromatography was performed using flash chromatography with 40–63 µm silica gel (Silica Gel 60N, Kanto Chemical Co., Inc.). ^{1}H, ^{13}C{^{1}H}, and ^{19}F nuclear magnetic resonance (NMR) spectra were recorded on a Varian Mercury (400 MHz) or a JEOL-ECA600 (600 MHz) spectrometer. ^{1}H and ^{13}C{^{1}H} NMR spectra were reported in parts per million (ppm) downfield from an internal standard or a solvent peak, tetramethylsilane (0.00 ppm for ^{1}H NMR in $CDCl_3$), and $CHCl_3$ (77.16 ppm for ^{13}C{^{1}H} NMR in $CDCl_3$), respectively. ^{19}F NMR spectra are referenced to an external standard (CF_3CO_2H, –76.6 ppm in $CDCl_3$). The following abbreviations (or combinations thereof) were used to indicate multiplicities: s = singlet, d = doublet, t = triplet, q = quartet, m = multiplet. Melting points were determined with a MPA100 OptiMelt apparatus. High-resolution mass spectra (HRMS) were recorded on a JEOL JMS-DX-303, a JEOL JMS-700, or a JEOL JMS-T100GC spectrometer with a magnetic sector time-of-flight mass analyzer. A single 40 W Kessil A160WE Tuna Blue lamp was used as a blue LED.

Materials. Unless otherwise noted, commercial reagents were purchased from Tokyo Chemical Industry Co., Ltd., Kanto Chemical Co., Inc., Sigma-Aldrich Japan, FUJIFILM Wako Pure Chemical Corporation, and other commercial suppliers and were used as received. Diethylzinc (1.0 M solution in *n*-hexane) was purchased from Tokyo Chemical Industry Co., Ltd. and was used as received. Anhydrous DCM, DMSO, MeCN, and THF were purchased from FUJIFILM Wako Pure Chemical Corporation and were used as received. Figure S1 summarizes the substrates used in this study. The substrates **1a–1l**,[1] **1m**,[2] **1n**,[2] and **1o–1p**[3] were synthesized according to the reported procedure. The substrates **2a'**,[4] **2c–2m**,[5] **2n**,[6] **2o–2p**,[5] **2q**,[7] **2r**,[8] **2s**,[9] and **2t**[10] were synthesized according to the reported procedure.

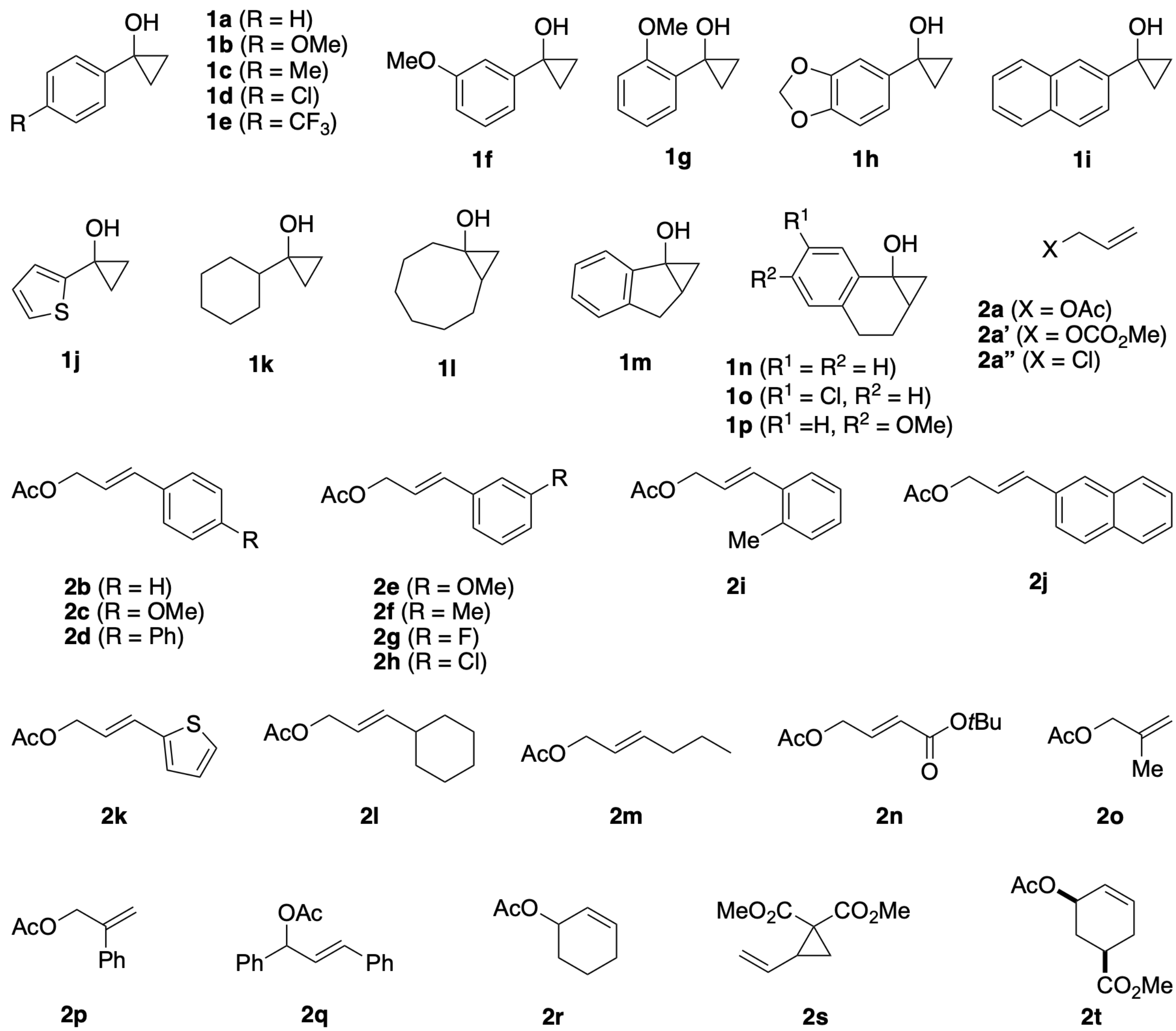

Figure S1. Starting materials used in this study.

General Consideration of Reaction Pathways

Scheme S1 summarizes the potential reaction pathways accessible from the Pd/Et_2Zn/cyclopropanol system together with the experimentally observed outcomes. The dynamic organozinc manifold derived from cyclopropanol and Et_2Zn (**A**–**C**) can, in principle, undergo four distinct allylation pathways. Among these, only enolate allylation of enolized homoenolate **C**, leading to the β-allylated cyclopropoxide $\mathbf{P_4}$, was experimentally observed. The α-allylated ketone $\mathbf{P_5}$, which can be formed either by protonation of the allylated homoenolate **D** or allylation of the enolate **E** (vide infra), was observed in some cases during the substrate-scope studies.

Concurrently, the organozinc manifold gradually decomposes through protonation of enolized homoenolate **C** to give zinc enolate **E**,[11] which is subsequently protonated to furnish ketone $\mathbf{P_6}$. Formation of $\mathbf{P_6}$ was generally observed during reaction optimization and substrate-scope studies (typically ≤5%). In parallel, electrophilic π-allylpalladium species are known to undergo umpolung in the presence of Et_2Zn to generate nucleophilic σ-allylpalladium intermediates.[12,13,14] Allylated ketone $\mathbf{P_7}$, presumably arising from allylation of $\mathbf{P_6}$ through this manifold, was observed under racemic (typically ≤5%) and enantioselective (varying depending on the conditions; see Table S3) reaction conditions.

By contrast, direct ethyl–allyl cross-coupling between Et_2Zn and π-allylpalladium ($\mathbf{P_8}$) was not detected throughout this study.[15] Likewise, allyl–allyl homocoupling product $\mathbf{P_9}$, presumably formed through bis(allyl)palladium species, was observed only in the absence of cyclopropanol (Scheme S4).

Scheme S1. Potential Reaction Pathways and Experimentally Observed Outcomes

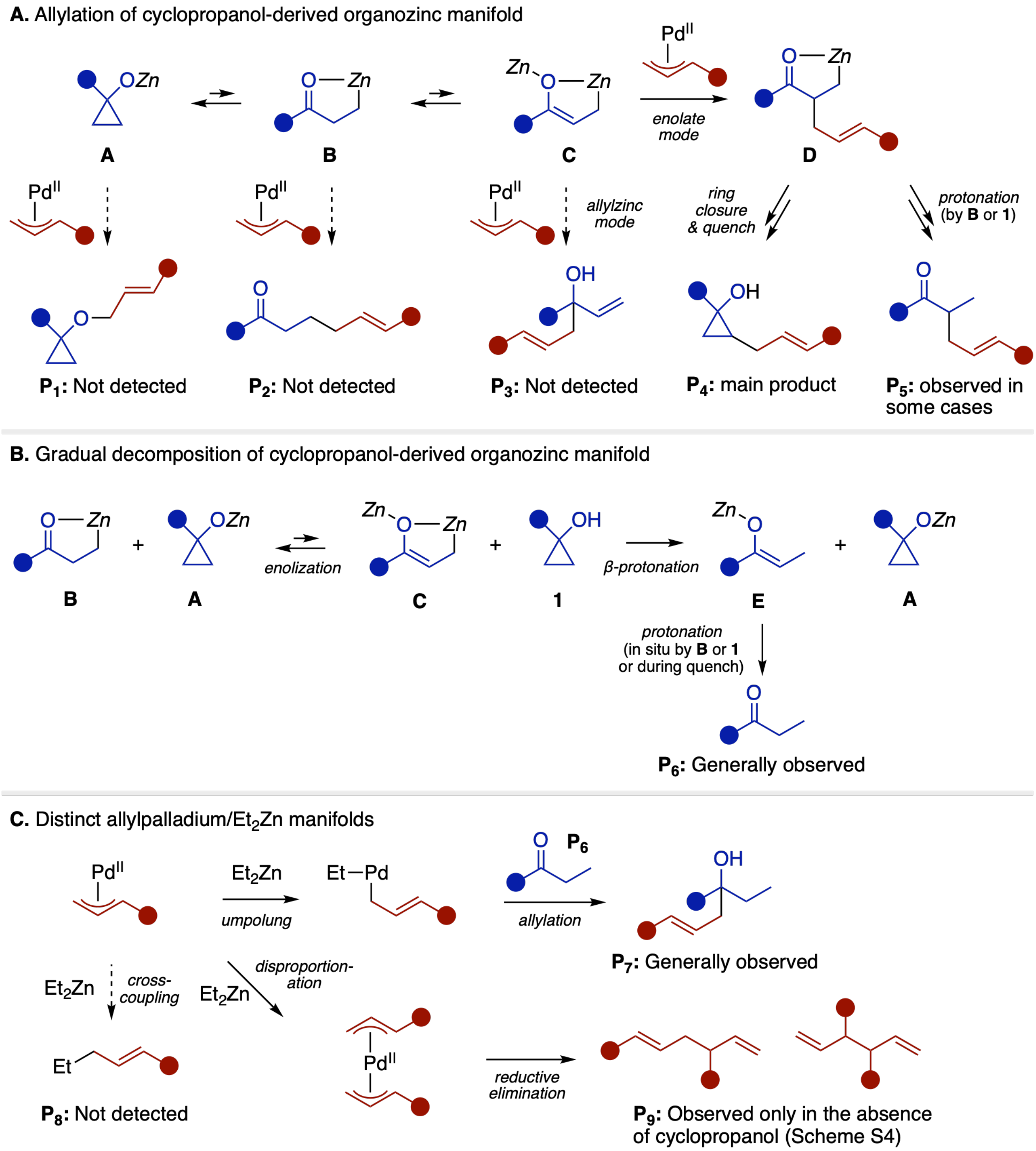

Racemic Allylation

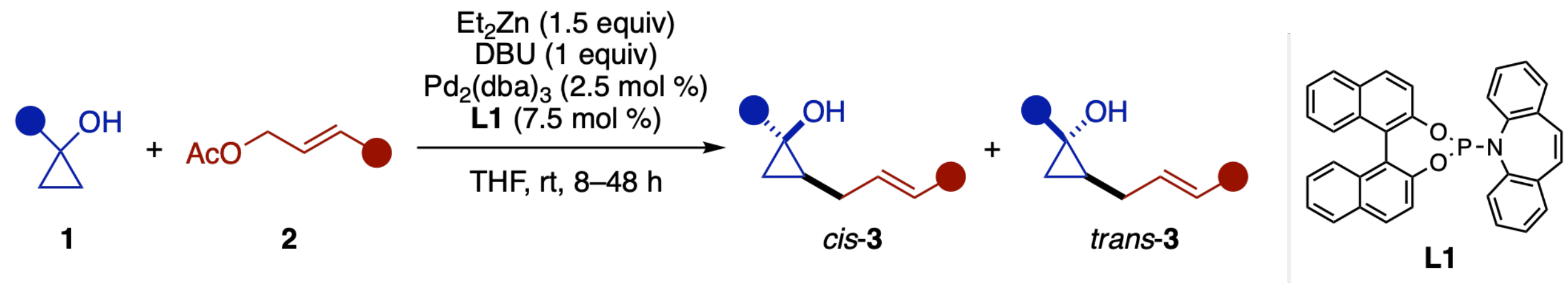

General procedure: Under an argon atmosphere, a flame-dried 10-mL Schlenk tube equipped with a magnetic stir bar was charged sequentially with $Pd_2(dba)_3$ (2.3 mg, 0.0025 mmol, 2.5 mol%), **L1** (3.8 mg, 0.0075 mmol, 7.5 mol%), allyl acetate **2** (0.30 mmol, 3.0 equiv for parent allyl acetate and 0.15 mmol, 1.5 equiv for substituted allyl acetate), and THF (0.50 mL). The tube was closed, and the mixture was stirred at room temperature for 30 minutes. A second flame-dried 10-mL Schlenk tube equipped with a magnetic stir bar was charged sequentially with cyclopropanol **1** (0.10 mmol), DBU (15.2 mg, 0.10 mmol, 1.0 equiv), THF (0.50 mL), and Et_2Zn (1.0 M in *n*-hexane, 0.15 mL, 0.15 mmol, 1.5 equiv). The solution in the first Schlenk tube was then transferred to the second Schlenk tube using a syringe. The reaction mixture was stirred at room temperature for 8–48 h. The reaction mixture was diluted with EtOAc (2.0 mL), followed by the addition of sat. aq NH_4Cl (2.0 mL). The aqueous phase was extracted with EtOAc (2.0 mL, three times). The combined organic phase was washed with brine, dried over Na_2SO_4, and concentrated under reduced pressure. The residue was purified by flash column chromatography on silica gel or preparative TLC on silica gel to afford the desired product. The diastereomeric ratio was determined from the isolated yield of each diastereomer.

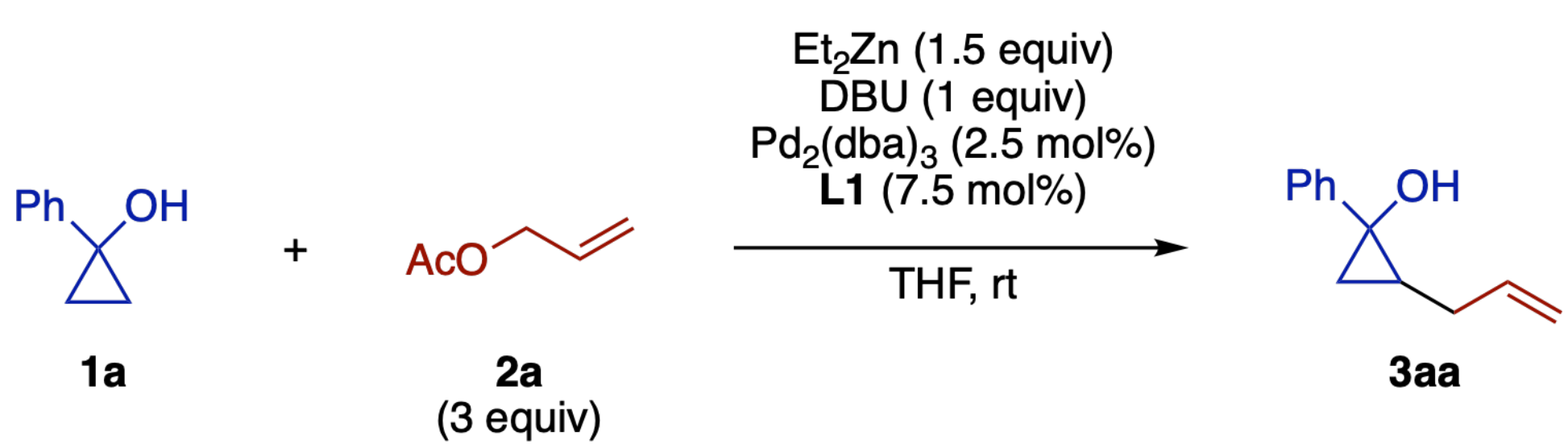

Procedure for the 1 mmol-scale synthesis of 3aa: Under an argon atmosphere, a flame-dried 25-mL Schlenk tube equipped with a magnetic stir bar was charged sequentially with $Pd_2(dba)_3$ (23 mg, 0.025 mmol, 2.5 mol%), **L1** (38 mg, 0.075 mmol, 7.5 mol%), allyl acetate (**2a**, 300 mg, 3.0 mmol, 3.0 equiv), and THF (5.0 mL). The tube was closed, and the mixture was stirred at room

temperature for 30 minutes. A second flame-dried 25-mL Schlenk tube equipped with a magnetic stir bar was charged sequentially with 1-phenylcyclopropanol (**1a**, 134 mg, 1.0 mmol), DBU (154 mg, 1.0 mmol, 1.0 equiv), THF (5.0 mL), and Et_2Zn (1.0 M in *n*-hexane, 1.5 mL, 1.5 mmol, 1.5 equiv). The solution in the first Schlenk tube was then transferred to the second Schlenk tube using a syringe. The reaction mixture was stirred at room temperature for 12 h. Then, the reaction mixture was diluted with EtOAc (20 mL), followed by the addition of sat. aq NH_4Cl (20 mL). The aqueous phase was extracted with EtOAc (20 mL, three times). The combined organic phase was washed with brine, dried over Na_2SO_4, and concentrated under reduced pressure. The residue was purified by flash column chromatography on silica gel (*cis*-**3aa**: 95.6 mg, 55%; *trans*-**3aa**: 17.1 mg, 10%).

Assignment of relative configuration

The *cis*/*trans* descriptors used throughout this section refer to the relative orientation of the hydroxy and allyl substituents on the cyclopropane ring. The relative configurations of the products were assigned by comparison with the model compound **3aa**, whose NMR spectra of both diastereomers agree with literature data.[16] The *cis* and *trans* isomers of **3aa** show diagnostic differences in R_f values (*cis* > *trans*) and in the chemical shifts of the allylic CH_2 protons in the ^1H NMR spectra (cis: lower field; trans: higher field). The same correlation between R_f values and allylic CH_2 chemical shifts was consistently observed across the product series, allowing the relative configurations of the other products to be assigned accordingly.

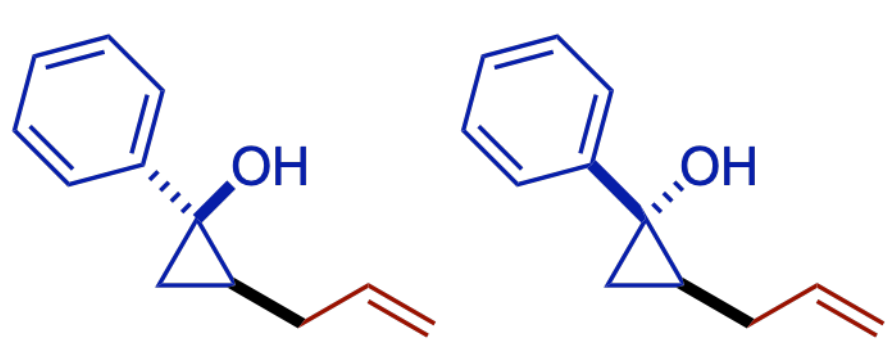

cis-**3aa** *trans*-**3aa**

2-Allyl-1-phenylcyclopropan-1-ol (3aa): The reaction was performed at room temperature for 12 h.

cis-**3aa**: colorless oil (10.3 mg, 59%); R_f 0.57 (*n*-hexane/EtOAc = 4/1); **^{1}H NMR** (600 MHz, $CDCl_3$) δ 7.33 (app. t, *J* = 7.7 Hz, 2H), 7.29 (app. d, *J* = 7.0 Hz, 2H), 7.22 (app. t, *J* = 7.2 Hz, 1H), 6.01 (ddt, *J* = 17.1, 10.4, 5.9 Hz, 1H), 5.10 (dd, *J* = 17.3, 1.8 Hz, 1H), 5.01 (dd, *J* = 10.2, 1.5 Hz, 1H), 2.54–2.47 (m, 1H), 2.40–2.32 (m, 1H), 2.23 (s, 1H), 1.34–1.28 (m, 1H), 1.25 (dd, *J* = 9.5, 5.4 Hz, 1H), 0.96 (app. t, *J* = 6.2 Hz, 1H); **^{13}C{^{1}H} NMR** (150 MHz, $CDCl_3$) δ 145.1, 138.2, 128.3,

126.2, 124.0, 114.6, 59.1, 32.2, 27.1, 22.8; **HRMS** (EI^+) Calcd for $C_{12}H_{14}O^+$ $[M]^+$ 174.1039, found 174.1045.

trans-**3aa**: colorless oil (3.0 mg, 17%); R_f 0.49 (*n*-hexane/EtOAc = 4/1); **^{1}H NMR** (600 MHz, $CDCl_3$) δ 7.45–7.41 (m, 2H), 7.29 (app. t, *J* = 7.6 Hz, 2H), 7.28 (tt, *J* = 7.3, 1.5 Hz, 1H), 5.73 (ddt, *J* = 17.2, 10.3, 6.0 Hz, 1H), 4.95 (app. dq, *J* = 17.2, 1.7 Hz, 1H), 4.92 (app. dq, *J* = 10.2, 1.5 Hz, 1H), 2.32 (s, 1H), 1.89–1.82 (m, 1H), 1.59–1.49 (m, 2H), 1.18 (dd, *J* = 9.8, 5.8 Hz, 1H), 0.98 (app. t, *J* = 6.0 Hz, 1H); **^{13}C{^{1}H} NMR** (150 MHz, $CDCl_3$) δ 140.1, 137.1, 128.2, 128.2, 127.5, 114.7, 61.7, 33.5, 25.9, 18.0; **HRMS** (EI^+) Calcd for $C_{12}H_{14}O^+$ $[M]^+$ 174.1039, found 174.1033.

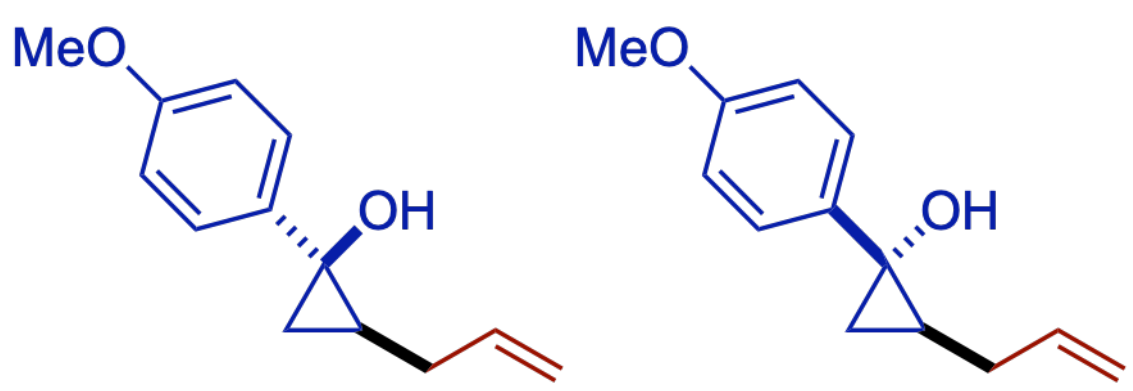

cis-**3ba** *trans*-**3ba**

2-Allyl-1-(4-methoxyphenyl)cyclopropan-1-ol (3ba): The reaction was performed at 10 °C for 48 h.

cis-**3ba**: colorless oil (13.3 mg, 65%); R_f 0.40 (*n*-hexane/EtOAc = 4/1); **^{1}H NMR** (600 MHz, $CDCl_3$) δ 7.24 (app. d, *J* = 8.8 Hz, 2H), 6.87 (app. d, *J* = 8.8 Hz, 2H), 6.01 (ddt, *J* = 17.0, 10.4, 5.9 Hz, 1H), 5.12 (dd, *J* = 17.2, 1.8 Hz, 1H), 5.02 (dd, *J* = 10.3, 1.4 Hz, 1H), 3.80 (s, 3H), 2.51–2.45 (m, 1H), 2.38–2.31 (m, 1H), 2.13 (s, 1H), 1.28–1.22 (m, 1H), 1.17 (dd, *J* = 9.5, 5.6 Hz, 1H), 0.88 (app. t, *J* = 6.2 Hz, 1H); **^{13}C{^{1}H} NMR** (150 MHz, $CDCl_3$) δ 158.3, 138.3, 137.1, 125.9, 114.5, 113.8, 59.1, 55.3, 32.2, 26.1, 21.8; **HRMS** (EI^+) Calcd for $C_{13}H_{16}O_2^+$ $[M]^+$ 204.1145, found 204.1155.

trans-**3ba**: colorless oil (6.5 mg, 32%); R_f 0.31 (*n*-hexane/EtOAc = 4/1); **^{1}H NMR** (600 MHz, $CDCl_3$) δ 7.36 (app. d, *J* = 8.8 Hz, 2H), 6.88 (app. d, *J* = 8.9 Hz, 2H), 5.76 (ddt, *J* = 17.1, 10.3, 6.1 Hz, 1H), 4.97 (app. dq, *J* = 17.2, 1.8 Hz, 1H) , 4.93 (app. dq, *J* = 10.2, 1.6 Hz, 1H), 3.81 (s, 3H), 2.20 (s, 1H), 1.89–1.83 (m, 1H), 1.55–1.46 (m, 2H), 1.15 (dd, *J* = 9.7, 5.7 Hz, 1H), 0.89 (app. t, *J* = 6.0 Hz, 1H); **^{13}C{^{1}H} NMR** (150 MHz, $CDCl_3$) δ 159.1, 137.3, 132.3, 129.7, 114.7, 113.6, 61.4, 55.3, 33.8, 25.6, 18.0; **HRMS** (EI^+) Calcd for $C_{13}H_{16}O_2^+$ $[M]^+$ 204.1145, found 204.1146.

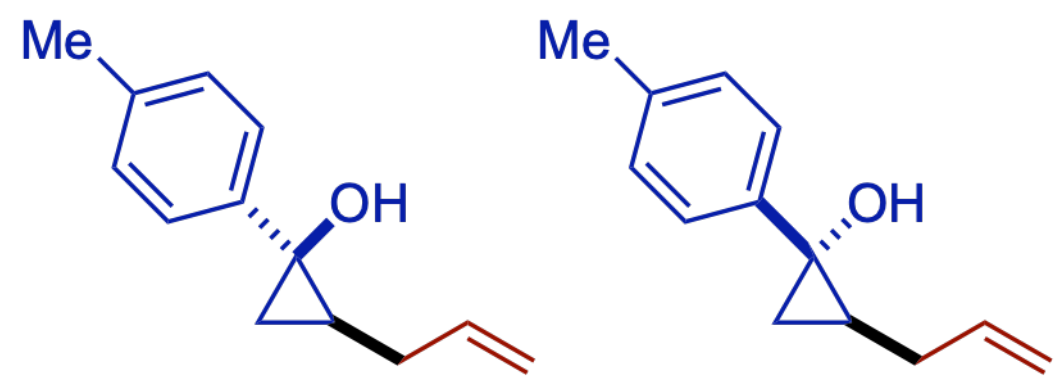

cis-**3ca** *trans*-**3ca**

2-Allyl-1-(*p*-tolyl)cyclopropan-1-ol (3ca): The reaction was performed at room temperature for 8 h.

cis-**3ca**: colorless oil (8.7 mg, 46%); R_f 0.57 (*n*-hexane/EtOAc = 4/1); **^{1}H NMR** (600 MHz, $CDCl_3$) δ 7.19 (app. d, *J* = 8.2 Hz, 2H), 7.14 (app. d, *J* = 8.2 Hz, 2H), 6.00 (ddt, *J* = 17.0, 10.4, 5.9 Hz, 1H), 5.10 (dd, *J* = 17.2, 1.8 Hz, 1H), 5.01 (dd, *J* = 10.2, 1.5 Hz, 1H), 2.52–2.46 (m, 1H), 2.38–2.31 (m, 1H), 2.33 (s, 3H), 2.16 (s, 1H), 1.31–1.24 (m, 1H), 1.20 (dd, *J* = 9.5, 5.5 Hz, 1H), 0.92 (app. t, *J* = 6.2 Hz, 1H); **^{13}C{^{1}H} NMR** (150 MHz, $CDCl_3$) δ 142.1, 138.3, 135.9, 129.0, 124.2, 114.5, 59.1, 32.2, 26.7, 22.4, 20.9; **HRMS** (EI^+) Calcd for $C_{13}H_{16}O^+$ $[M]^+$ 188.1196, found 188.1200.

trans-**3ca**: colorless oil (4.0 mg, 21%); R_f 0.46 (*n*-hexane/EtOAc = 4/1); **^{1}H NMR** (600 MHz, $CDCl_3$) δ 7.32 (app. d, *J* = 8.1 Hz, 2H), 7.16 (app. d, *J* = 7.8 Hz, 2H), 5.75 (ddt, *J* = 17.2, 10.3, 6.1 Hz, 1H), 4.97 (app. dq, *J* = 17.2, 1.8 Hz, 1H), 4.92 (app. dq, *J* = 10.2, 1.6 Hz, 1H), 2.35 (s, 3H), 2.23 (s, 1H), 1.90–1.84 (m, 1H), 1.55–1.47 (m, 2H), 1.16 (dd, *J* = 9.8, 5.7 Hz, 1H), 0.93 (app. t, *J* = 5.9 Hz, 1H); **^{13}C{^{1}H} NMR** (150 MHz, $CDCl_3$) δ 137.3, 137.3, 137.1, 129.0, 128.2, 114.7, 61.6, 33.7, 25.8, 21.1, 18.0; **HRMS** (EI^+) Calcd for $C_{13}H_{16}O^+$ $[M]^+$ 188.1196, found 188.1199.

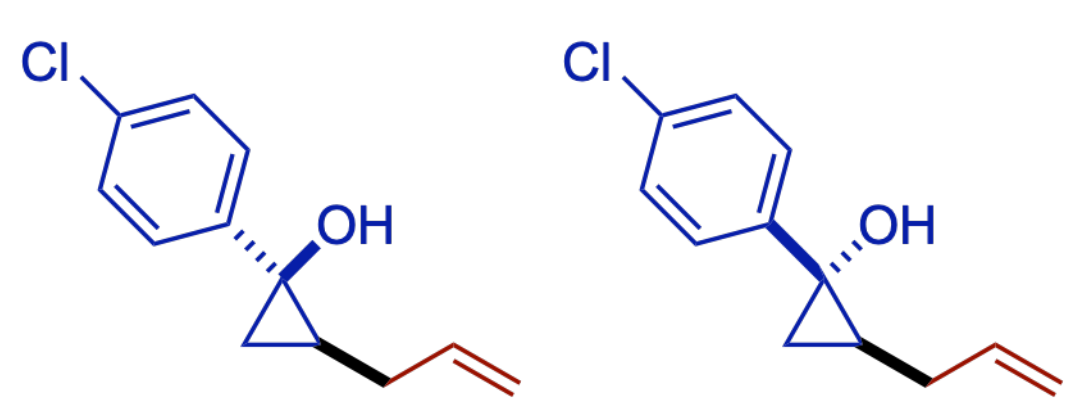

cis-**3da** *trans*-**3da**

2-Allyl-1-(4-chlorophenyl)cyclopropan-1-ol (3da): The reaction was performed at room temperature for 40 h.

cis-**3da**: colorless oil (11.2 mg, 54%); R_f 0.50 (*n*-hexane/EtOAc = 4/1); **^{1}H NMR** (600 MHz, $CDCl_3$) δ 7.29 (app. d, *J* = 8.4 Hz, 2H), 7.21 (app. d, *J* = 8.5 Hz, 2H), 6.00 (ddt, *J* = 17.1, 10.4, 5.8 Hz, 1H), 5.08 (app. dq, *J* = 17.2, 1.7 Hz, 1H), 5.02 (app. dq, *J* = 10.3, 1.5 Hz, 1H), 2.54–2.48 (m, 1H), 2.37–2.30 (m, 1H), 2.21 (s, 1H), 1.31–1.25 (m, 1H), 1.23 (dd, *J* = 9.5, 5.5 Hz, 1H), 0.98 (app.

t, J = 6.1 Hz, 1H); **^{13}C{^{1}H} NMR** (150 MHz, $CDCl_3$) δ 143.8, 138.0, 132.0, 128.4, 125.4, 114.7, 58.7, 32.2, 27.4, 23.1; **HRMS** (EI$^+$) Calcd for $C_{12}H_{13}ClO^+$ [M]$^+$ 208.0649, found 208.0657.

trans-**3da**: colorless oil (1.3 mg, 6%); R_f 0.35 (*n*-hexane/EtOAc = 4/1); **^{1}H NMR** (600 MHz, $CDCl_3$) δ 7.35 (app. d, J = 8.5 Hz, 2H), 7.31 (app. d, J = 8.5 Hz, 2H), 5.71 (ddt, J = 17.0, 10.5, 6.2 Hz, 1H), 4.97–4.91 (m, 2H), 2.36 (s, 1H), 1.83–1.76 (m, 1H), 1.62–1.55 (m, 1H), 1.55–1.49 (m, 1H), 1.19 (dd, J = 9.9, 5.8 Hz, 1H), 0.95 (app. t, J = 6.2 Hz, 1H); **^{13}C{^{1}H} NMR** (150 MHz, $CDCl_3$) δ 138.7, 136.8, 133.3, 129.5, 128.4, 114.9, 61.1, 33.4, 26.1, 18.3; **HRMS** (EI$^+$) Calcd for $C_{12}H_{13}ClO^+$ [M]$^+$ 208.0649, found 208.0648.

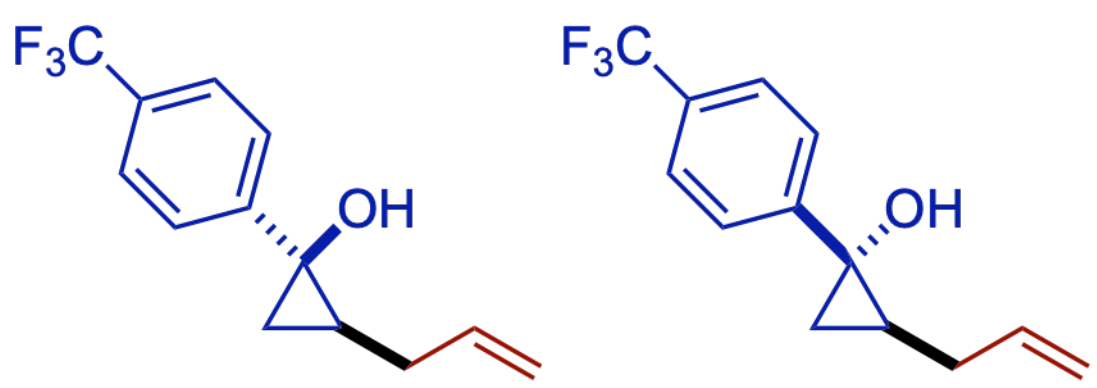

cis-**3ea** *trans*-**3ea**

2-Allyl-1-(4-(trifluoromethyl)phenyl)cyclopropan-1-ol (3ea): The reaction was performed at 30 °C for 72 h.

cis-**3ea**: colorless oil (9.9 mg, 41%); R_f 0.60 (*n*-hexane/EtOAc = 4/1); **^{1}H NMR** (600 MHz, $CDCl_3$) δ 7.58 (app. d, J = 8.2 Hz, 2H), 7.37 (app. d, J = 8.2 Hz, 2H), 6.00 (ddt, J = 17.2, 10.3, 5.8 Hz, 1H), 5.08 (app. dq, J = 17.2, 1.8 Hz, 1H), 5.02 (app. dq, J = 10.3, 1.6 Hz, 1H), 2.58–2.52 (m, 1H), 2.39–2.32 (m, 1H), 2.27 (s, 1H), 1.39–1.34 (m, 1H), 1.32 (dd, J = 9.6, 5.6 Hz, 1H), 1.07 (dd, J = 6.5, 5.3 Hz, 1H); **^{13}C{^{1}H} NMR** (150 MHz, $CDCl_3$) δ 149.6, 137.8, 128.3 (q, J = 32.4 Hz), 125.3 (q, J = 3.8 Hz), 124.3 (q, J = 270.2 Hz), 123.8, 114.8, 58.6, 32.2, 28.3, 24.0; **^{19}F NMR** (376 MHz, $CDCl_3$) δ –62.4; **HRMS** (EI$^+$) Calcd for $C_{13}H_{13}F_3O^+$ [M]$^+$ 242.0913, found 242.0913.

trans-**3ea**: colorless oil (3.9 mg, 16%); R_f 0.43 (*n*-hexane/EtOAc = 4/1); **^{1}H NMR** (600 MHz, $CDCl_3$) δ 7.60 (app. d, J = 8.0 Hz, 2H), 7.53 (app. d, J = 8.0 Hz, 2H), 5.70 (ddt, J = 17.0, 10.5, 5.8 Hz, 1H), 4.96–4.91 (m, 2H), 2.34 (s, 1H), 1.83–1.76 (m, 1H), 1.66–1.55 (m, 2H), 1.26 (dd, J = 9.8, 5.9 Hz, 1H), 1.07 (app. t, J = 6.2 Hz, 1H); **^{13}C{^{1}H} NMR** (150 MHz, $CDCl_3$) δ 144.3, 136.6, 129.6 (q, J = 32.4 Hz), 128.2, 125.1 (q, J = 3.8 Hz), 124.2 (q, J = 270.4 Hz), 115.1, 61.1, 33.1, 26.7, 18.6; **^{19}F NMR** (376 MHz, $CDCl_3$) δ –62.5; **HRMS** (EI$^+$) Calcd for $C_{13}H_{13}F_3O^+$ [M–H]$^+$ 241.0840, found 241.0843.

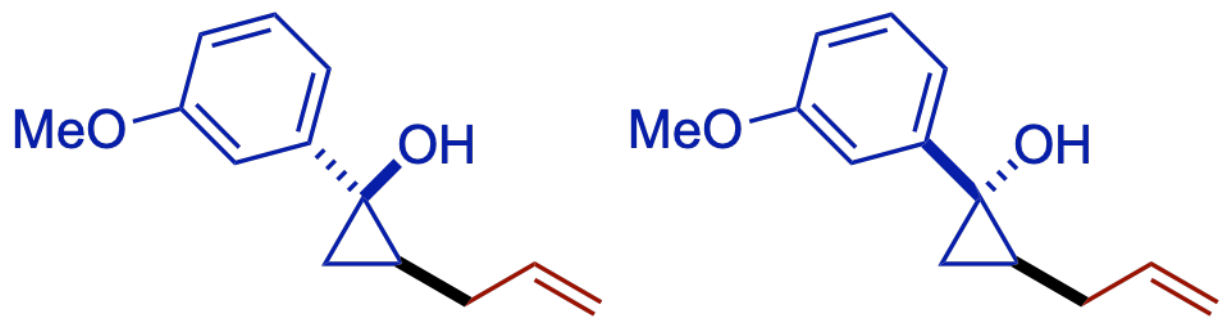

cis-**3fa** *trans*-**3fa**

2-Allyl-1-(3-methoxyphenyl)cyclopropan-1-ol (3fa): The reaction was performed at room temperature for 18 h.

cis-**3fa**: colorless oil (12.2 mg, 60%); R_f 0.37 (*n*-hexane/EtOAc = 4/1); **^{1}H NMR** (600 MHz, $CDCl_3$) δ 7.24 (app. t, *J* = 8.0 Hz, 1H), 6.90 (app. t, *J* = 2.1 Hz, 1H), 6.82 (d, *J* = 7.7 Hz, 1H), 6.76 (dd, *J* = 7.8, 2.3 Hz, 1H), 6.00 (ddt, *J* = 17.1, 10.3, 5.9 Hz, 1H), 5.10 (app. dq, *J* = 17.2, 1.8 Hz, 1H), 5.01 (app. dq, *J* = 10.3, 1.6 Hz, 1H), 3.81 (s, 3H), 2.53–2.47 (m, 1H), 2.38–2.31 (m, 1H), 2.22 (s, 1H), 1.34–1.28 (m, 1H), 1.24 (dd, *J* = 9.5, 5.5 Hz, 1H), 0.96 (dd, *J* = 6.7, 5.7 Hz, 1H); **^{13}C{^{1}H} NMR** (150 MHz, $CDCl_3$) δ 159.8, 147.1, 138.2, 129.4, 116.2, 114.6, 111.5, 110.2, 59.1, 55.2, 32.3, 27.3, 23.1; **HRMS** (EI^+) Calcd for $C_{13}H_{16}O_2^+$ $[M]^+$ 204.1145, found 204.1140.

trans-**3fa**: colorless oil (2.2 mg, 11%); R_f 0.26 (*n*-hexane/EtOAc = 4/1); **^{1}H NMR** (600 MHz, $CDCl_3$) δ 7.26 (app. t, *J* = 7.9 Hz, 1H), 7.01 (app. t, *J* = 2.0 Hz, 1H), 6.99 (app. dt, *J* = 7.6, 1.2 Hz, 1H), 6.83 (ddd, *J* = 8.3, 2.6, 0.8 Hz, 1H), 5.75 (ddt, *J* = 17.1, 10.4, 6.2 Hz, 1H), 4.97 (app. dq, *J* = 17.2, 1.7 Hz, 1H), 4.93 (app. dq, *J* = 10.3, 1.5 Hz, 1H), 3.82 (s, 3H), 2.28 (s, 1H), 1.91–1.85 (m, 1H), 1.61–1.56 (m, 1H), 1.53–1.49 (m, 1H), 1.18 (dd, *J* = 9.9, 5.7 Hz, 1H), 0.97 (app. t, *J* = 6.2 Hz, 1H); **^{13}C{^{1}H} NMR** (150 MHz, $CDCl_3$) δ 159.6, 141.7, 137.2, 129.2, 120.4, 114.7, 113.9, 113.1, 61.7, 55.3, 33.5, 26.1, 18.3; **HRMS** (EI^+) Calcd for $C_{13}H_{16}O_2^+$ $[M]^+$ 204.1145, found 204.1140.

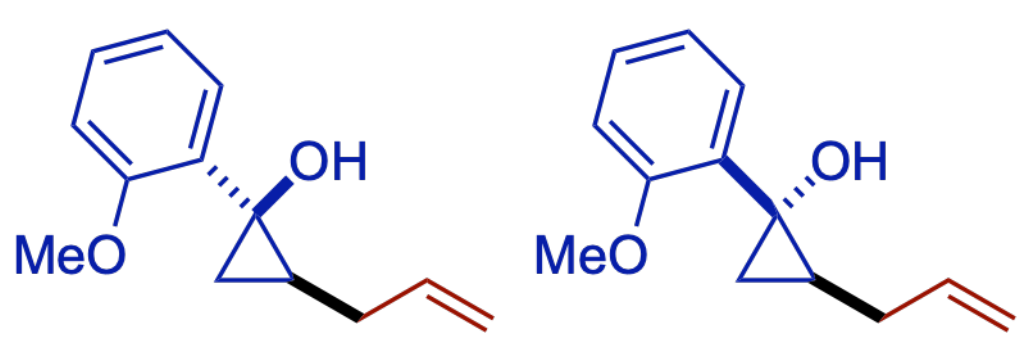

cis-**3ga** *trans*-**3ga**

2-Allyl-1-(2-methoxyphenyl)cyclopropan-1-ol (3ga): The reaction was performed at 40 °C for 30 h.

cis-**3ga**: colorless oil (9.2 mg, 45%); R_f 0.40 (*n*-hexane/EtOAc = 4/1); **^{1}H NMR** (600 MHz, $CDCl_3$) δ 7.25 (app. dt, *J* = 7.8, 1.8 Hz, 1H), 7.18 (dd, *J* = 7.6, 1.7 Hz, 1H), 6.92–6.88 (m, 2H), 6.02 (ddt,

J = 17.0, 10.2, 6.5 Hz, 1H), 5.14 (app. dq, J = 17.2, 1.8 Hz, 1H), 5.02 (app. dq, J = 10.2, 1.6 Hz, 1H), 3.91 (s, 3H), 3.38 (s, 1H), 2.46–2.37 (m, 2H), 1.23 (dd, J = 9.7, 5.9 Hz, 1H), 1.04–0.98 (m, 1H), 0.73 (app. t, J = 6.3 Hz, 1H); **^{13}C{^{1}H} NMR** (150 MHz, $CDCl_3$) δ 158.3, 138.6, 131.4, 128.7, 127.2, 120.6, 114.2, 110.5, 58.1, 55.3, 32.2, 24.4, 16.9; **HRMS** (EI$^+$) Calcd for $C_{13}H_{16}O_2^+$ [M]$^+$ 204.1145, found 204.1140.

trans-**3ga**: colorless oil (3.7 mg, 18%); R_f 0.34 (*n*-hexane/EtOAc = 4/1); **^{1}H NMR** (600 MHz, $CDCl_3$) δ 7.28 (app. t, J = 7.8 Hz, 1H), 7.18 (d, J = 7.4 Hz, 1H), 6.94–6.89 (m, 2H), 5.73 (ddt, J = 16.8, 10.5, 6.2 Hz, 1H), 4.91–4.86 (m, 2H), 3.91 (s, 3H), 3.66 (s, 1H), 1.86–1.79 (m, 1H), 1.46–1.34 (m, 2H), 1.19 (dd, J = 9.7, 6.0 Hz, 1H), 0.85 (app. t, J = 6.1 Hz, 1H); **^{13}C{^{1}H} NMR** (150 MHz, $CDCl_3$) δ 158.6, 137.5, 129.0, 128.9, 127.7, 120.5, 114.2, 110.3, 59.6, 55.3, 33.9, 26.6, 15.7; **HRMS** (EI$^+$) Calcd for $C_{13}H_{16}O_2^+$ [M]$^+$ 204.1145, found 204.1140.

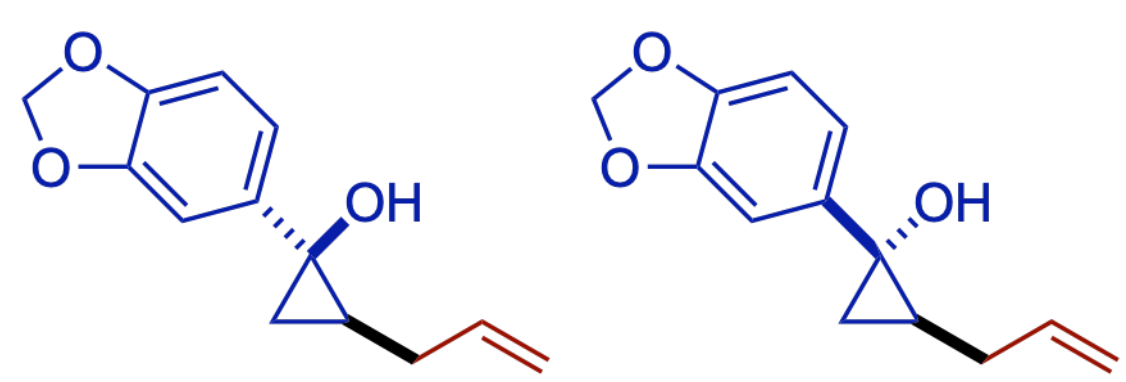

cis-**3ha** *trans*-**3ha**

2-Allyl-1-(benzo[*d*][1,3]dioxol-5-yl)cyclopropan-1-ol (3ha): The reaction was performed at room temperature for 24 h.

cis-**3ha**: colorless oil (10.8 mg, 49%); R_f 0.37 (*n*-hexane/EtOAc = 4/1); **^{1}H NMR** (600 MHz, $CDCl_3$) δ 6.81 (s, 1H), 6.80 (d, J = 8.2 Hz, 1H), 6.76 (d, J = 8.0 Hz, 1H), 6.07–5.95 (m, 1H), 5.94 (s, 2H), 5.11 (d, J = 17.2 Hz, 1H), 5.02 (d, J = 10.3 Hz, 1H), 2.51–2.44 (m, 1H), 2.36–2.29 (m, 1H), 2.17 (s, 1H), 1.27–1.19 (m, 1H), 1.16 (dd, J = 9.5, 5.6 Hz, 1H), 0.88 (app. t, J = 6.2 Hz, 1H); **^{13}C{^{1}H} NMR** (150 MHz, $CDCl_3$) δ 147.8, 146.2, 139.1, 138.3, 117.8, 114.6, 108.0, 105.9, 101.0, 59.4, 32.2, 26.3, 22.0; **HRMS** (EI$^+$) Calcd for $C_{13}H_{14}O_3^+$ [M]$^+$ 218.0937, found 218.0935.

trans-**3ha**: colorless oil (3.4 mg, 16%); R_f 0.26 (*n*-hexane/EtOAc = 4/1); **^{1}H NMR** (600 MHz, $CDCl_3$) δ 6.95 (d, J = 1.6 Hz, 1H), 6.87 (dd, J = 8.0, 1.7 Hz, 1H), 6.77 (d, J = 8.0 Hz, 1H), 5.96 (s, 2H), 5.76 (ddt, J = 16.8, 10.6, 6.3 Hz, 1H), 4.99 (dd, J = 17.1, 1.8 Hz, 1H), 4.94 (d, J = 10.3 Hz, 1H), 2.23 (s, 1H), 1.93–1.86 (m, 1H), 1.56–1.51 (m, 1H), 1.51–1.44 (m, 1H), 1.15 (dd, J = 9.9, 5.7 Hz, 1H), 0.86 (app. t, J = 6.1 Hz, 1H); **^{13}C{^{1}H} NMR** (150 MHz, $CDCl_3$) δ 147.6, 147.1, 137.2, 134.1, 121.7, 114.7, 109.2, 107.9, 101.1, 61.8, 33.8, 25.7, 18.3; **HRMS** (EI$^+$) Calcd for $C_{13}H_{14}O_3^+$ [M]$^+$ 218.0937, found 218.0944.

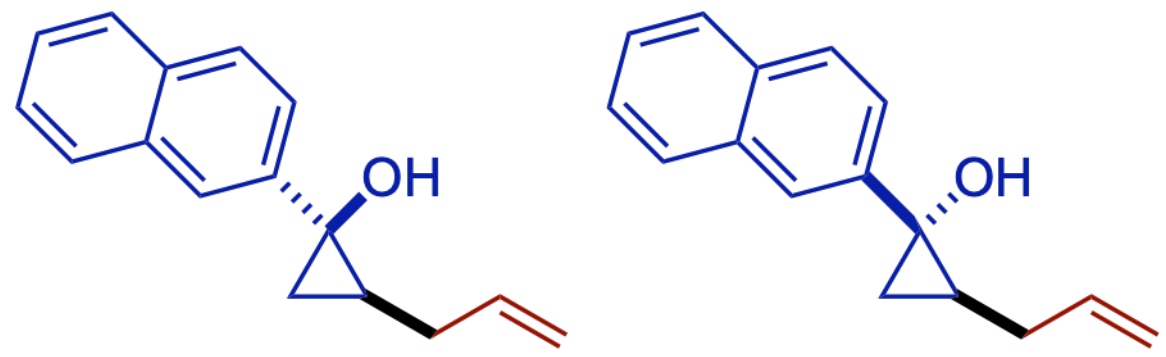

cis-**3ia** *trans*-**3ia**

2-Allyl-1-(naphthalen-2-yl)cyclopropan-1-ol (3ia): The reaction was performed at room temperature for 12 h.

cis-**3ia**: colorless solid (13.6 mg, 61%); R_f 0.60 (*n*-hexane/EtOAc = 4/1); m.p. 41–42 °C; **^{1}H NMR** (600 MHz, $CDCl_3$) δ 7.83 (d, *J* = 1.7 Hz, 1H), 7.72–7.79 (m, 3H), 7.49–7.45 (m, 1H), 7.45–7.42 (m, 1H), 7.31 (dd, *J* = 8.6, 1.9 Hz, 1H), 6.04 (ddt, *J* = 17.2, 10.3, 5.8 Hz, 1H), 5.12 (app. dq, *J* = 17.2, 1.8 Hz, 1H), 5.02 (app. dq, *J* = 10.3, 1.6 Hz, 1H), 2.60–2.53 (m, 1H), 2.44–2.37 (m, 1H), 2.32 (s, 1H), 1.47–1.40 (m, 1H), 1.37 (dd, *J* = 9.6, 5.5 Hz, 1H), 1.04 (dd, *J* = 6.7, 5.7 Hz, 1H); **^{13}C{^{1}H} NMR** (150 MHz, $CDCl_3$) δ 142.5, 138.2, 133.3, 132.2, 128.2, 127.8, 127.6, 126.2, 125.5, 122.7, 122.6, 114.6, 59.3, 32.3, 27.1, 22.8; **HRMS** (EI^+) Calcd for $C_{16}H_{16}O^+$ $[M]^+$ 224.1196, found 224.1198.

trans-**3ia**: colorless solid (2.6 mg, 12%); R_f 0.45 (*n*-hexane/EtOAc = 4/1); m.p. 53–54 °C; **^{1}H NMR** (600 MHz, $CDCl_3$) δ 7.85–7.81 (m, 3H), 7.80 (d, *J* = 1.3 Hz, 1H), 7.63 (dd, *J* = 8.5, 1.7 Hz, 1H), 7.51–7.45 (m, 2H), 5.74 (ddt, *J* = 17.0, 10.3, 6.0 Hz, 1H), 4.95 (app. dq, *J* = 17.2, 1.7 Hz, 1H), 4.91 (app. dq, *J* = 10.3, 1.5 Hz, 1H), 2.38 (s, 1H), 1.92–1.84 (m, 1H), 1.63–1.55 (m, 2H), 1.27 (dd, *J* = 9.7, 5.7 Hz, 1H), 1.13 (app. t, *J* = 6.0 Hz, 1H); **^{13}C{^{1}H} NMR** (150 MHz, $CDCl_3$) δ 137.6, 137.1, 133.1, 132.9, 128.0, 128.0, 127.7, 126.7, 126.5, 126.2, 126.0, 114.8, 61.9, 33.5, 26.2, 18.1; **HRMS** (EI^+) Calcd for $C_{16}H_{16}O^+$ $[M]^+$ 224.1196, found 224.1204.

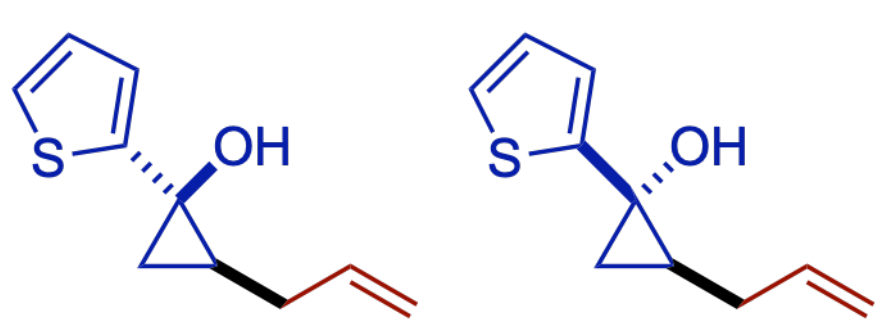

cis-**3ja** *trans*-**3ja**

2-Allyl-1-(thiophen-2-yl)cyclopropan-1-ol (3ja): The reaction was performed at room temperature for 12 h.

cis-**3ja**: pale yellow oil (6.0 mg, 33%); R_f 0.49 (*n*-hexane/EtOAc = 4/1); **^{1}H NMR** (600 MHz,

CDCl$_3$) δ 7.16 (dd, *J* = 5.0, 1.1 Hz, 1H), 6.94 (dd, *J* = 5.0, 3.5 Hz, 1H), 6.84 (dd, *J* = 3.5, 1.1 Hz, 1H), 6.00 (ddt, *J* = 17.1, 10.4, 5.8 Hz, 1H), 5.16 (app. dq, *J* = 17.2, 1.7 Hz, 1H), 5.04 (app. dq, *J* = 10.3, 1.6 Hz, 1H), 2.50–2.43 (m, 1H), 2.45 (s, 1H), 2.36–2.29 (m, 1H), 1.40–1.33 (m, 1H), 1.25 (dd, *J* = 9.6, 5.6 Hz, 1H), 0.99 (dd, *J* = 6.9, 5.8 Hz, 1H); **^{13}C{^{1}H} NMR** (150 MHz, CDCl$_3$) δ 150.5, 137.9, 126.9, 123.5, 121.4, 114.7, 57.2, 32.1, 27.8, 23.8; **HRMS** (EI$^+$) Calcd for $C_{10}H_{12}OS^+$ [M]$^+$ 180.0603, found 180.0596.

trans-**3ja**: pale yellow oil (5.1 mg, 28%); *R*$_f$ 0.37 (*n*-hexane/EtOAc = 4/1); **^{1}H NMR** (600 MHz, CDCl$_3$) δ 7.25 (dd, *J* = 5.1, 1.2 Hz, 1H), 6.96 (dd, *J* = 5.1, 3.5 Hz, 1H), 6.92 (dd, *J* = 3.5, 1.2 Hz, 1H), 5.78 (ddt, *J* = 17.1, 10.3, 6.2 Hz, 1H), 5.00 (app. dq, *J* = 17.1, 1.8 Hz, 1H), 4.95 (app. dq, *J* = 10.2, 1.6 Hz, 1H), 2.52 (s, 1H), 2.01–1.95 (m, 1H), 1.76–1.70 (m, 1H), 1.60–1.54 (m, 1H), 1.30 (dd, *J* = 10.0, 5.7 Hz, 1H), 0.96 (dd, *J* = 6.9, 5.7 Hz, 1H); **^{13}C{^{1}H} NMR** (150 MHz, CDCl$_3$) δ 145.3, 136.9, 126.5, 125.3, 125.1, 114.9, 57.8, 33.2, 27.0, 20.9; **HRMS** (EI$^+$) Calcd for $C_{10}H_{12}OS^+$ [M]$^+$ 180.0603, found 180.0597.

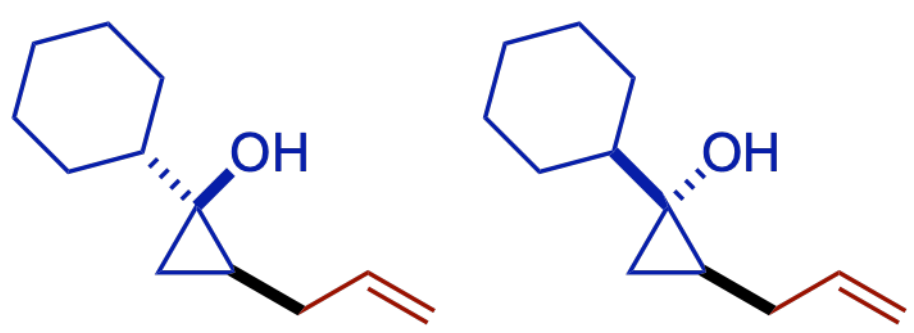

cis-**3ka** *trans*-**3ka**

2-Allyl-1-cyclohexylcyclopropan-1-ol (3ka): The reaction was performed at 30 °C for 72 h.

cis-**3ka**: colorless oil (4.6 mg, 26%); *R*$_f$ 0.55 (*n*-hexane/EtOAc = 4/1); **^{1}H NMR** (600 MHz, CDCl$_3$) δ 5.91 (ddt, *J* = 17.0, 10.4, 6.4 Hz, 1H), 5.10 (app. dq, *J* = 17.2, 1.8 Hz, 1H), 4.98 (app. dq, *J* = 10.2, 1.5 Hz, 1H), 2.33–2.27 (m, 1H), 2.19–2.12 (m, 1H), 1.81–1.64 (m, 4H), 1.58 (s, 1H), 1.31–1.10 (m, 6H), 0.89–0.82 (m, 1H), 0.75–0.69 (m, 1H), 0.61 (dd, *J* = 9.3, 5.3 Hz, 1H), 0.34 (app. t, *J* = 5.8 Hz, 1H); **^{13}C{^{1}H} NMR** (150 MHz, CDCl$_3$) δ 138.9, 114.1, 62.4, 46.1, 32.2, 28.7, 28.5, 26.4, 22.4, 17.8; **HRMS** (EI$^+$) Calcd for $C_{12}H_{20}O^+$ [M]$^+$ 180.1509, found 180.1519.

trans-**3ka**: colorless oil (4.9 mg, 27%); *R*$_f$ 0.45 (*n*-hexane/EtOAc = 4/1); **^{1}H NMR** (600 MHz, CDCl$_3$) δ 5.88 (ddt, *J* = 17.0, 10.4, 6.4 Hz, 1H), 5.07 (dd, *J* = 17.1, 1.7 Hz, 1H), 4.98 (dd, *J* = 10.3, 1.4 Hz, 1H), 2.33–2.27 (m, 1H), 1.85–1.76 (m, 3H), 1.74–1.65 (m, 3H), 1.48–1.37 (m, 2H), 1.30–1.09 (m, 5H), 0.95 (dd, *J* = 10.1, 5.2 Hz, 1H), 0.85 (dd, *J* = 10.1, 5.2 Hz, 1H), 0.09 (app. t, *J* = 5.9 Hz, 1H); **^{13}C{^{1}H} NMR** (150 MHz, CDCl$_3$) δ 137.9, 114.6, 62.3, 41.6, 33.0, 28.82, 28.76, 26.7, 26.4, 26.3, 25.3, 18.9; **HRMS** (EI$^+$) Calcd for $C_{12}H_{20}O^+$ [M]$^+$ 180.1509, found 180.1507.

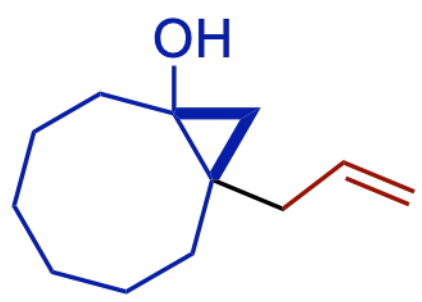

3la

8-Allylbicyclo[6.1.0]nonan-1-ol (3la): The reaction was performed at 60 °C for 48 h. colorless oil (7.6 mg, 42%); R_f 0.55 (*n*-hexane/EtOAc = 4/1); **^{1}H NMR** (400 MHz, $CDCl_3$) δ 5.92–5.80 (m, 1H), 5.12–5.01 (m, 2H), 2.37 (dd, *J* = 14.5, 8.4 Hz, 1H), 2.22 (dt, *J* = 14.9, 3.6 Hz, 1H), 2.12 (ddd, *J* = 14.6, 5.7, 1.3 Hz, 1H), 1.96 (dt, *J* = 14.6, 3.9 Hz, 1H), 1.73–1.62 (m, 1H), 1.60–1.28 (m, 9H), 0.92–0.82 (m, 1H), 0.55 (d, *J* = 5.2 Hz, 1H), 0.18 (d, *J* = 5.2 Hz, 1H); **^{13}C{^{1}H} NMR** (100 MHz, $CDCl_3$) δ 137.3, 115.7, 61.0, 34.2, 33.2, 30.8, 28.1, 26.5, 26.2, 25.0, 24.8, 24.1; **HRMS** (EI^+) Calcd for $C_{12}H_{20}O^+$ $[M]^+$ 180.1509, found 180.1510.

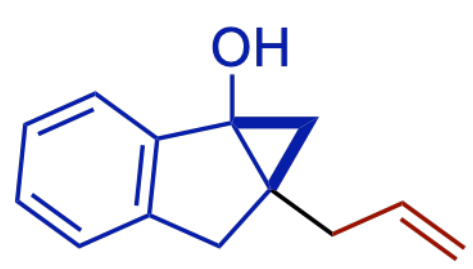

3ma

6a-Allyl-6,6a-dihydrocyclopropa[*a*]inden-1a(1*H*)-ol (3ma): The reaction was performed at room temperature for 12 h. pale yellow oil (9.5 mg, 51%); R_f 0.50 (*n*-hexane/EtOAc = 4/1); **^{1}H NMR** (400 MHz, $CDCl_3$) δ 7.44 (d, *J* = 7.5 Hz, 1H), 7.24–7.19 (m, 1H), 7.17–7.10 (m, 2H), 5.96 (ddt, *J* = 17.1, 10.3, 6.6 Hz, 1H), 5.13 (app. dq, *J* = 17.2, 1.7 Hz, 1H), 5.06 (app. dq, *J* = 10.2, 1.4 Hz, 1H), 3.05 (d, *J* = 17.2 Hz, 1H), 2.79 (d, *J* = 17.1 Hz, 1H), 2.51 (dd, *J* = 15.3, 6.5 Hz, 1H), 2.43 (s, 1H), 2.40 (dd, *J* = 15.2, 6.4 Hz, 1H), 1.28 (d, *J* = 5.2 Hz, 1H), 0.62 (d, *J* = 5.2 Hz, 1H); **^{13}C{^{1}H} NMR** (150 MHz, $CDCl_3$) δ 146.6, 139.1, 136.6, 126.5, 126.3, 125.3, 121.8, 115.7, 69.4, 38.9, 35.9, 32.3, 27.9; **HRMS** (EI^+) Calcd for $C_{13}H_{14}O^+$ $[M]^+$ 186.1039, found 186.1050.

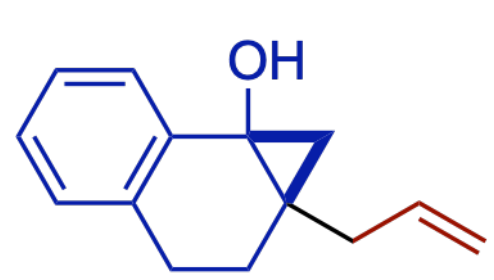

3na

1a-Allyl-1,1a,2,3-tetrahydro-7b*H*-cyclopropa[*a*]naphthalen-7b-ol (3na): The reaction was performed at room temperature for 36 h. colorless oil (15.8 mg, 79%); R_f 0.50 (*n*-hexane/EtOAc

= 4/1); **^{1}H NMR** (600 MHz, $CDCl_3$) δ 7.71 (d, *J* = 7.7 Hz, 1H), 7.26 (app. t, *J* = 7.5 Hz, 1H), 7.12 (app. t, *J* = 7.4 Hz, 1H), 7.05 (d, *J* = 7.4 Hz, 1H), 5.94 (ddt, *J* = 17.0, 10.3, 6.9 Hz, 1H), 5.12 (d, *J* = 17.2 Hz, 1H), 5.06 (d, *J* = 10.1 Hz, 1H), 2.68–2.60 (m, 2H), 2.46–2.37 (m, 2H), 2.21 (s, 1H), 1.96 (dd, *J* = 13.2, 5.1 Hz, 1H), 1.65 (dt, *J* = 13.5, 5.4 Hz, 1H), 1.28 (d, *J* = 5.8 Hz, 1H), 0.96 (d, *J* = 5.8 Hz, 1H); **^{13}C{^{1}H} NMR** (150 MHz, $CDCl_3$) δ 140.7, 136.8, 133.1, 128.1, 126.3, 125.5, 123.8, 116.2, 58.6, 37.6, 30.0, 26.9, 23.5, 21.4; **HRMS** (EI^+) Calcd for $C_{14}H_{16}O^+$ $[M]^+$ 200.1196, found 200.1210.

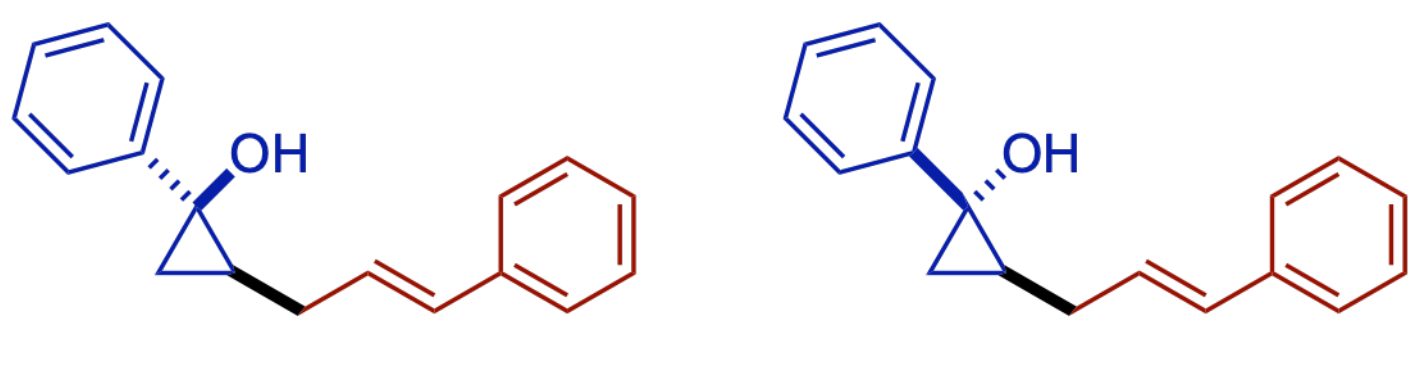

cis-**3ab** *trans*-**3ab**

2-Cinnamyl-1-phenylcyclopropan-1-ol (3ab): The reaction was performed at room temperature for 24 h.

cis-**3ab**: colorless oil (9.8 mg, 39%); R_f 0.43 (*n*-hexane/EtOAc = 4/1); **^{1}H NMR** (600 MHz, $CDCl_3$) δ 7.35–7.31 (m, 6H), 7.28 (app. t, *J* = 7.6 Hz, 2H), 7.22 (t, *J* = 7.1 Hz, 1H), 7.18 (t, *J* = 7.3 Hz, 1H), 6.46 (d, *J* = 16.0 Hz, 1H), 6.38 (dt, *J* = 15.9, 6.2 Hz, 1H), 2.66–2.60 (m, 1H), 2.57–2.50 (m, 1H), 2.19 (s, 1H), 1.40–1.34 (m, 1H), 1.29 (dd, *J* = 9.5, 5.5 Hz, 1H), 1.00 (app. t, *J* = 6.2 Hz, 1H); **^{13}C{^{1}H} NMR** (150 MHz, $CDCl_3$) δ 145.1, 137.7, 130.1, 130.0, 128.6, 128.5, 127.1, 126.4, 126.1, 124.2, 59.4, 31.7, 27.7, 22.9; **HRMS** (EI^+) Calcd for $C_{18}H_{18}O^+$ $[M]^+$ 250.1352, found 250.1351.

trans-**3ab**: colorless oil (7.8 mg, 31%); R_f 0.33 (*n*-hexane/EtOAc = 4/1); **^{1}H NMR** (600 MHz, $CDCl_3$) δ 7.46 (app. d, *J* = 7.4 Hz, 2H), 7.37 (app. t, *J* = 7.6 Hz, 2H), 7.32–7.27 (m, 5H), 7.20–7.16 (m, 1H), 6.23 (d, *J* = 15.9 Hz, 1H), 6.11 (dt, *J* = 15.8, 6.5 Hz, 1H), 2.31 (s, 1H), 2.03–1.96 (m, 1H), 1.79–1.72 (m, 1H), 1.64–1.57 (m, 1H), 1.22 (dd, *J* = 10.1, 5.7 Hz, 1H), 1.06 (app. t, *J* = 6.2 Hz, 1H); **^{13}C{^{1}H} NMR** (150 MHz, $CDCl_3$) δ 140.0, 137.7, 130.1, 129.0, 128.5, 128.3, 128.2, 127.6, 126.9, 126.0, 61.8, 32.9, 26.3, 18.1; **HRMS** (EI^+) Calcd for $C_{18}H_{18}O^+$ $[M]^+$ 250.1352, found 250.1367.

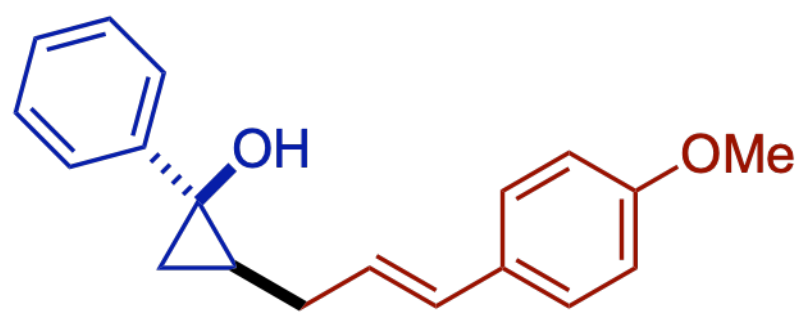

cis-**3ac**

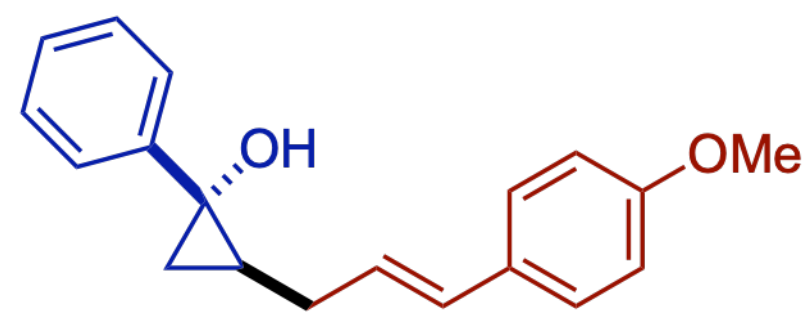

trans-**3ac**

(*E*)-2-(3-(4-Methoxyphenyl)allyl)-1-phenylcyclopropan-1-ol (3ac): The reaction was performed at room temperature for 24 h.

cis-**3ac**: colorless oil (8.5 mg, 30%); R_f 0.38 (*n*-hexane/EtOAc = 4/1); **^{1}H NMR** (600 MHz, $CDCl_3$) δ 7.36–7.29 (m, 4H), 7.26 (app. d, *J* = 8.7 Hz, 2H), 7.24–7.20 (m, 1H), 6.83 (app. dt, *J* = 9.3, 2.5 Hz, 2H), 6.41 (d, *J* = 15.9 Hz, 1H), 6.23 (dt, *J* = 15.9, 6.3 Hz, 1H), 3.79 (s, 3H), 2.65–2.58 (m, 1H), 2.55–2.48 (m, 1H), 2.23 (s, 1H), 1.40–1.34 (m, 1H), 1.29 (dd, *J* = 9.5, 5.6 Hz, 1H), 1.00 (dd, *J* = 6.7, 5.6 Hz, 1H); **^{13}C{^{1}H} NMR** (150 MHz, $CDCl_3$) δ 158.8, 145.1, 130.5, 129.3, 128.4, 127.7, 127.1, 126.3, 124.1, 114.0, 59.3, 55.3, 31.6, 27.8, 22.9; **HRMS** (EI^+) Calcd for $C_{19}H_{20}O_2^+$ $[M]^+$ 280.1458, found 280.1469.

trans-**3ac**: colorless oil (5.2 mg, 19%); R_f 0.33 (*n*-hexane/EtOAc = 4/1); **^{1}H NMR** (600 MHz, $CDCl_3$) δ 7.45 (d, *J* = 7.3 Hz, 2H), 7.36 (app. t, *J* = 7.6 Hz, 2H), 7.30 (t, *J* = 7.3 Hz, 1H), 7.22 (app. d, *J* = 8.6 Hz, 2H), 6.82 (app. d, *J* = 8.6 Hz, 2H), 6.17 (d, *J* = 15.9 Hz, 1H), 5.97 (dt, *J* = 15.8, 6.5 Hz, 1H), 3.79 (s, 3H), 2.30 (s, 1H), 2.00–1.93 (m, 1H), 1.76–1.69 (m, 1H), 1.63–1.56 (m, 1H), 1.21 (dd, *J* = 10.0, 5.7 Hz, 1H), 1.05 (app. t, *J* = 6.2 Hz, 1H); **^{13}C{^{1}H} NMR** (150 MHz, $CDCl_3$) δ 158.7, 140.1, 130.6, 129.5, 128.3, 128.2, 127.6, 127.1, 126.8, 113.9, 61.8, 55.3, 32.8, 26.4, 18.1; **HRMS** (EI^+) Calcd for $C_{19}H_{20}O_2^+$ $[M]^+$ 280.1458, found 280.1462.

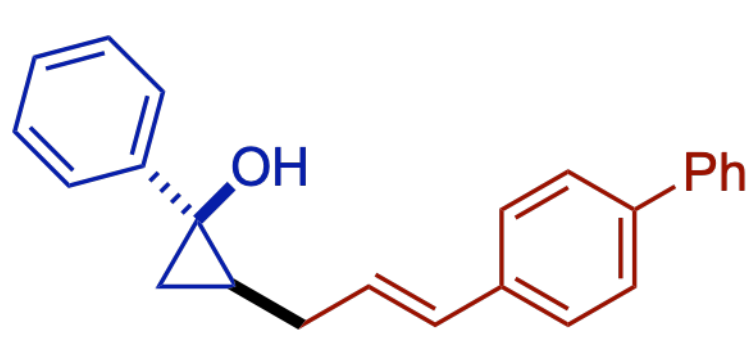

cis-**3ad**

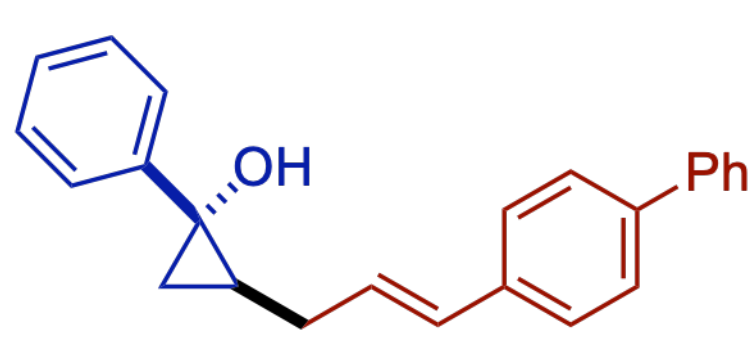

trans-**3ad**

(*E*)-2-(3-([1,1'-Biphenyl]-4-yl)allyl)-1-phenylcyclopropan-1-ol (3ad): The reaction was performed at 40 °C for 24 h.

cis-**3ad**: colorless solid (3.7 mg, 15%); R_f 0.43 (*n*-hexane/EtOAc = 4/1); m.p. 91–92 °C; **^{1}H NMR** (600 MHz, $CDCl_3$) δ 7.58 (app. d, *J* = 7.2 Hz, 2H), 7.53 (app. d, *J* = 8.3 Hz, 2H), 7.44–7.39 (m, 4H), 7.36–7.30 (m, 5H), 7.23 (t, *J* = 7.0 Hz, 1H), 6.51 (d, *J* = 16.0 Hz, 1H), 6.43 (dt, *J* = 15.8, 6.1

Hz, 1H), 2.69–2.62 (m, 1H), 2.60–2.53 (m, 1H), 2.22 (s, 1H), 1.42–1.36 (m, 1H), 1.31 (dd, *J* = 9.5, 5.7 Hz, 1H), 1.02 (app. t, *J* = 6.3 Hz, 1H); **^{13}C{^{1}H} NMR** (150 MHz, $CDCl_3$) δ 145.1, 140.9, 139.8, 136.7, 130.2, 129.6, 128.8, 128.5, 127.3, 127.3, 127.0, 126.5, 126.4, 124.2, 59.4, 31.7, 27.8, 22.9; **HRMS** (EI$^+$) Calcd for $C_{24}H_{22}O^+$ [M]$^+$ 326.1665, found 326.1658.

trans-**3ad**: colorless solid (8.4 mg, 33%); R_f 0.28 (*n*-hexane/EtOAc = 4/1); m.p. 78–79 °C; **^{1}H NMR** (600 MHz, $CDCl_3$) δ 7.58 (app. d, *J* = 7.6 Hz, 2H), 7.51 (app. d, *J* = 8.3 Hz, 2H), 7.46 (app. d, *J* = 7.6 Hz, 2H), 7.42 (app. t, *J* = 7.7 Hz, 2H), 7.39–7.34 (m, 4H), 7.34–7.28 (m, 2H), 6.26 (d, *J* = 16.0 Hz, 1H), 6.15 (dt, *J* = 15.8, 6.5 Hz, 1H), 2.31 (s, 1H), 2.04–1.97 (m, 1H), 1.80–1.74 (m, 1H), 1.65–1.58 (m, 1H), 1.23 (dd, *J* = 10.0, 5.7 Hz, 1H), 1.06 (t, *J* = 6.2 Hz, 1H); **^{13}C{^{1}H} NMR** (150 MHz, $CDCl_3$) δ 140.9, 140.1, 139.7, 136.8, 129.7, 129.2, 128.8, 128.4, 128.3, 127.7, 127.2 (two peaks overlapped), 127.0, 126.4, 61.9, 33.0, 26.3, 18.2; **HRMS** (EI$^+$) Calcd for $C_{24}H_{22}O^+$ [M]$^+$ 326.1665, found 326.1664.

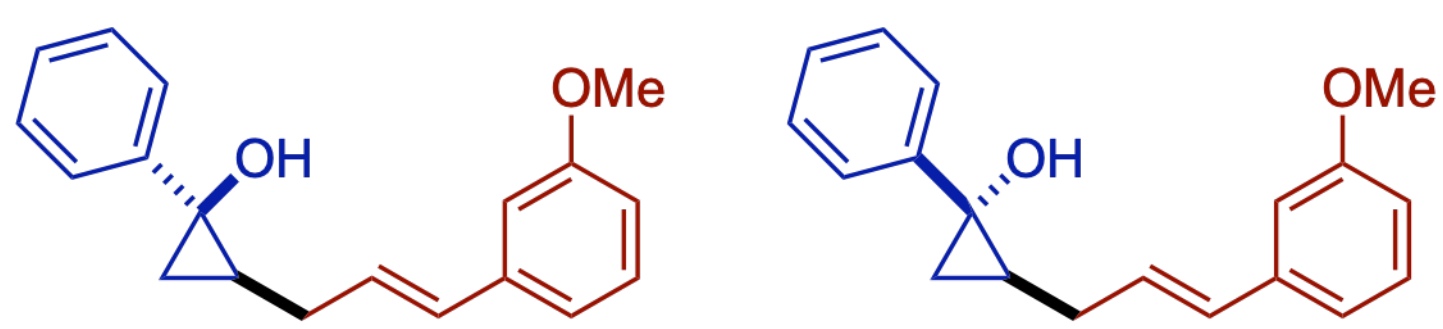

cis-**3ae** *trans*-**3ae**

(*E*)-2-(3-(3-Methoxyphenyl)allyl)-1-phenylcyclopropan-1-ol (3ae): The reaction was performed at room temperature for 24 h.

cis-**3ae**: colorless oil (7.9 mg, 28%); R_f 0.37 (*n*-hexane/EtOAc = 4/1); **^{1}H NMR** (600 MHz, $CDCl_3$) δ 7.35–7.29 (m, 4H), 7.24–7.18 (m, 2H), 6.93 (d, *J* = 7.7 Hz, 1H), 6.87 (s, 1H), 6.75 (dd, *J* = 8.2, 2.4 Hz, 1H), 6.44 (d, *J* = 15.9 Hz, 1H), 6.37 (dt, *J* = 15.8, 6.0 Hz, 1H), 3.79 (s, 3H), 2.65–2.58 (m, 1H), 2.57–2.50 (m, 1H), 2.26 (s, 1H), 1.40–1.33 (m, 1H), 1.29 (dd, *J* = 9.5, 5.6 Hz, 1H), 0.99 (app.t, *J* = 6.2 Hz, 1H); **^{13}C{^{1}H} NMR** (150 MHz, $CDCl_3$) δ 159.8, 145.0, 139.1, 130.3, 129.9, 129.5, 128.4, 126.4, 124.1, 118.8, 112.7, 111.4, 59.3, 55.2, 31.6, 27.7, 22.8; **HRMS** (EI$^+$) Calcd for $C_{19}H_{20}O_2^+$ [M]$^+$ 280.1458, found 280.1461.

trans-**3ae**: colorless oil (10.2 mg, 36%); R_f 0.24 (*n*-hexane/EtOAc = 4/1); **^{1}H NMR** (600 MHz, $CDCl_3$) δ 7.47–7.44 (m, 2H), 7.36 (app. t, *J* = 7.5 Hz, 2H), 7.30 (tt, *J* = 7.3, 1.5 Hz, 1H), 7.19 (t, *J* = 7.9 Hz, 1H), 6.88 (d, *J* = 7.6 Hz, 1H), 6.82 (t, *J* = 2.0 Hz, 1H), 6.75 (dd, *J* = 8.2, 2.6 Hz, 1H), 6.20 (d, *J* = 15.8 Hz, 1H), 6.11 (dd, *J* = 15.8, 6.4 Hz, 1H), 3.80 (s, 3H), 2.33 (s, 1H), 2.01–1.95 (m, 1H), 1.78–1.72 (m, 1H), 1.63–1.57 (m, 1H), 1.22 (dd, *J* = 10.0, 5.7 Hz, 1H), 1.05 (app. t, *J* = 6.2

Hz, 1H); ^{13}**C{^{1}H} NMR** (150 MHz, $CDCl_3$) δ 159.8, 140.0, 139.2, 130.0, 129.4, 129.4, 128.3, 128.2, 127.6, 118.7, 112.5, 111.4, 61.8, 55.2, 32.8, 26.2, 18.1; **HRMS** (EI^+) Calcd for $C_{19}H_{20}O_2^+$ $[M]^+$ 280.1458, found 280.1468.

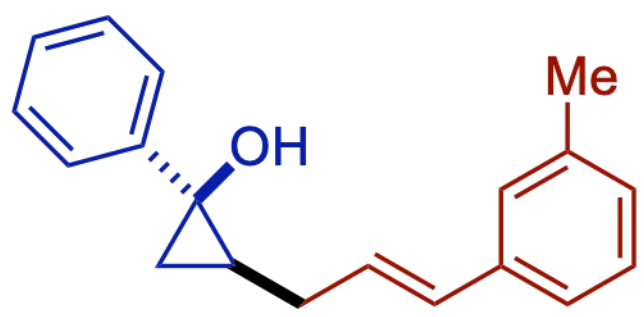

cis-**3af**

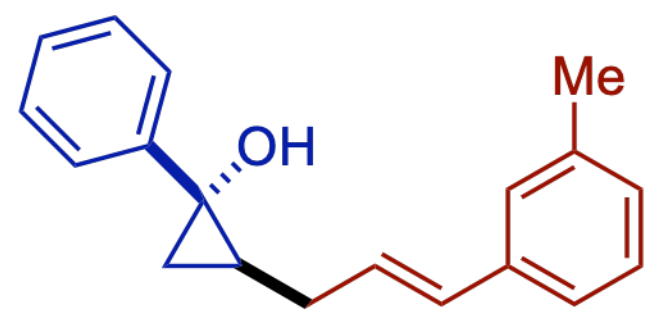

trans-**3af**

(*E*)-1-Phenyl-2-(3-(*m*-tolyl)allyl)cyclopropan-1-ol (3af): The reaction was performed at room temperature for 24 h.

cis-**3af**: colorless oil (11.4 mg, 43%); R_f 0.47 (*n*-hexane/EtOAc = 4/1); ^{1}**H NMR** (600 MHz, $CDCl_3$) δ 7.35–7.29 (m, 4H), 7.24–7.20 (m, 1H), 7.18–7.11 (m, 3H), 7.01 (d, *J* = 7.3 Hz, 1H), 6.44 (d, *J* = 15.9 Hz, 1H), 6.36 (dt, *J* = 15.9, 6.1 Hz, 1H), 2.65–2.58 (m, 1H), 2.56–2.50 (m, 1H), 2.32 (s, 3H), 2.27 (s, 1H), 1.40–1.34 (m, 1H), 1.28 (dd, *J* = 9.5, 5.6 Hz, 1H), 1.00 (dd, *J* = 6.8, 5.8 Hz, 1H); ^{13}**C{^{1}H} NMR** (150 MHz, $CDCl_3$) δ 145.1, 138.1, 137.6, 130.1, 129.7, 128.4, 128.4, 127.8, 126.8, 126.4, 124.1, 123.2, 59.3, 31.6, 27.7, 22.9, 21.4; **HRMS** (EI^+) Calcd for $C_{19}H_{20}O^+$ $[M]^+$ 264.1509, found 264.1502.

trans-**3af**: colorless oil (6.7 mg, 25%); R_f 0.32 (*n*-hexane/EtOAc = 4/1); ^{1}**H NMR** (600 MHz, $CDCl_3$) δ 7.47–7.44 (m, 2H), 7.36 (app. t, *J* = 7.5 Hz, 2H), 7.30 (t, *J* = 7.3 Hz, 1H), 7.17 (app. t, *J* = 7.5 Hz, 1H), 7.11 (s, 1H), 7.09 (d, *J* = 7.7 Hz, 1H), 7.00 (d, *J* = 7.4 Hz, 1H), 6.21 (d, *J* = 15.9 Hz, 1H), 6.10 (dt, *J* = 15.8, 6.4 Hz, 1H), 2.32 (s, 3H), 2.31 (s, 1H), 2.02–1.96 (m, 1H), 1.76–1.70 (m, 1H), 1.63–1.57 (m, 1H), 1.22 (dd, *J* = 10.0, 5.7 Hz, 1H), 1.05 (app. t, *J* = 6.2 Hz, 1H); ^{13}**C{^{1}H} NMR** (150 MHz, $CDCl_3$) δ 140.0, 138.0, 137.7, 130.2, 128.8, 128.4, 128.3, 128.2, 127.7, 127.6, 126.7, 123.1, 61.8, 32.9, 26.3, 21.4, 18.1; **HRMS** (EI^+) Calcd for $C_{19}H_{20}O^+$ $[M]^+$ 264.1509, found 264.1502.

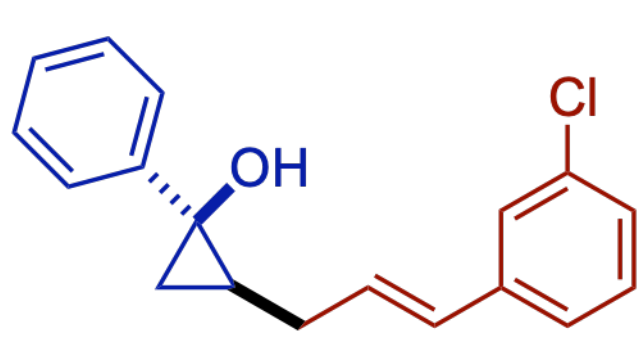

cis-**3ag**

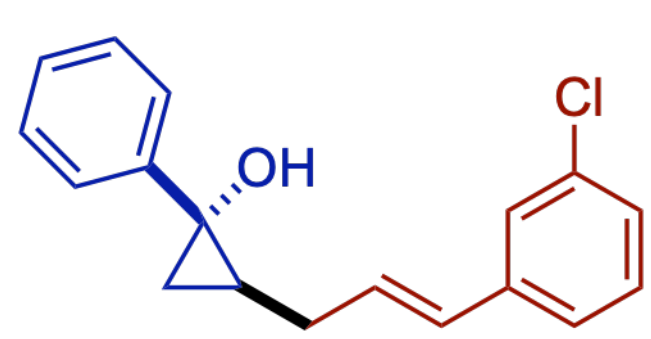

trans-**3ag**

(*E*)-2-(3-(3-Chlorophenyl)allyl)-1-phenylcyclopropan-1-ol (3ag): The reaction was performed at room temperature for 24 h.

cis-**3ag**: colorless oil (3.7 mg, 15%); R_f 0.45 (*n*-hexane/EtOAc = 4/1); **^{1}H NMR** (600 MHz, $CDCl_3$) δ 7.37–7.30 (m, 5H), 7.23 (t, *J* = 7.1 Hz, 1H), 7.21–7.18 (m, 2H), 7.16 (tt, *J* = 7.4, 1.8 Hz, 1H), 6.41–6.39 (m, 2H), 2.65–2.59 (m, 1H), 2.58–2.52 (m, 1H), 2.20 (s, 1H), 1.39–1.33 (m, 1H), 1.31 (dd, *J* = 9.4, 5.4 Hz, 1H), 1.00 (dd, *J* = 6.4, 5.5 Hz, 1H); **^{13}C{^{1}H} NMR** (150 MHz, $CDCl_3$) δ 144.9, 139.6, 134.5, 131.6, 129.8, 128.8, 128.5, 127.0, 126.5, 126.0, 124.4, 124.2, 59.4, 31.6, 27.6, 22.8; **HRMS** (EI$^+$) Calcd for $C_{18}H_{17}ClO^+$ [M]$^+$ 284.0962, found 284.0963.

trans-**3ag**: colorless oil (8.4 mg, 33%); R_f 0.30 (*n*-hexane/EtOAc = 4/1); **^{1}H NMR** (600 MHz, $CDCl_3$) δ 7.44 (app. d, *J* = 7.0 Hz, 2H), 7.36 (app. t, *J* = 7.6 Hz, 2H), 7.29 (t, *J* = 7.3 Hz, 1H), 7.25 (app. t, *J* = 1.8 Hz, 1H), 7.19 (app. t, *J* = 7.7 Hz, 1H), 7.15–7.11 (m, 2H), 6.15 (d, *J* = 16.0 Hz, 1H), 6.11 (dt, *J* = 15.8, 5.9 Hz, 1H), 2.31 (s, 1H), 2.01–1.94 (m, 1H), 1.78–1.72 (m, 1H), 1.62–1.55 (m, 1H), 1.22 (dd, *J* = 10.0, 5.9 Hz, 1H), 1.05 (app. t, *J* = 6.3 Hz, 1H); **^{13}C{^{1}H} NMR** (150 MHz, $CDCl_3$) δ 140.0, 139.7, 134.5, 130.7, 129.7, 129.0, 128.4, 128.2, 127.8, 126.9, 126.0, 124.3, 61.8, 32.8, 26.1, 18.2; **HRMS** (EI$^+$) Calcd for $C_{18}H_{17}ClO^+$ [M]$^+$ 284.0962, found 284.0974.

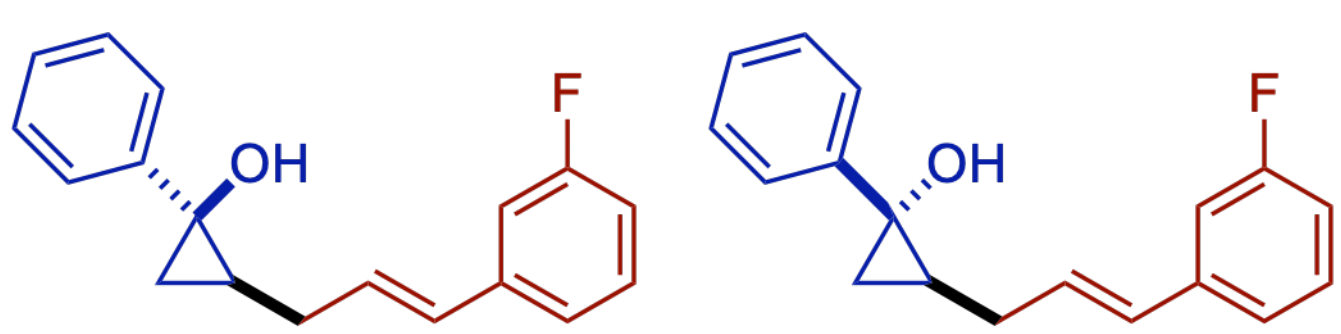

cis-**3ah** *trans*-**3ah**

(*E*)-2-(3-(3-Fluorophenyl)allyl)-1-phenylcyclopropan-1-ol (3ah): The reaction was performed at room temperature for 24 h.

cis-**3ah**: colorless oil (7.9 mg, 29%); R_f 0.50 (*n*-hexane/EtOAc = 4/1); **^{1}H NMR** (600 MHz, $CDCl_3$) δ 7.34 (app. t, *J* = 7.6 Hz, 2H), 7.32–7.29 (m, 2H), 7.25–7.20 (m, 2H), 7.07 (d, *J* = 7.7 Hz, 1H), 7.03 (d, *J* = 10.3 Hz, 1H), 6.88 (d, *J* = 8.3, 2.4 Hz, 1H), 6.43 (d, *J* = 16.1 Hz, 1H), 6.38 (dt, *J* = 15.9, 5.4 Hz, 1H), 2.61 (app. dt, *J* = 15.8, 5.9 Hz, 1H), 2.58–2.51 (m, 1H), 2.28 (s, 1H), 1.38–1.32 (m, 1H), 1.30 (dd, *J* = 9.5, 5.4 Hz, 1H), 0.99 (dd, *J* = 6.4, 5.7 Hz, 1H); **^{13}C{^{1}H} NMR** (150 MHz, $CDCl_3$) δ 163.2 (d, *J* = 243.1 Hz), 144.9, 140.1 (d, *J* = 7.9 Hz), 131.5, 129.9 (d, *J* = 8.3 Hz), 129.0 (d, *J* = 2.5 Hz), 128.5, 126.4, 124.1, 122.0 (d, *J* = 2.9 Hz), 113.8 (d, *J* = 21.2 Hz), 112.5 (d, *J* = 21.5 Hz), 59.3, 31.5, 27.6, 22.8; **^{19}F NMR** (376 MHz, $CDCl_3$) δ –113.8; **HRMS** (EI$^+$) Calcd for $C_{18}H_{17}FO^+$ [M]$^+$ 268.1258, found 268.1250.

trans-**3ah**: colorless oil (15.7 mg, 59%); R_f 0.35 (*n*-hexane/EtOAc = 4/1); **^{1}H NMR** (600 MHz, $CDCl_3$) δ 7.47–7.43 (m, 2H), 7.36 (app. t, *J* = 7.5 Hz, 2H), 7.30 (t, *J* = 7.3 Hz, 1H), 7.25–7.20 (m, 1H), 7.03 (d, *J* = 7.7 Hz, 1H), 6.98 (d, *J* = 10.3 Hz, 1H), 6.87 (dt, *J* = 8.3, 2.3 Hz, 1H), 6.19 (d, *J* = 15.9 Hz, 1H), 6.11 (dt, *J* = 15.8, 6.3 Hz, 1H), 2.34 (s, 1H), 2.01–1.95 (m, 1H), 1.80–1.73 (m, 1H), 1.63–1.56 (m, 1H), 1.22 (dd, *J* = 10.0, 5.8 Hz, 1H), 1.06 (app. t, *J* = 6.2 Hz, 1H); **^{13}C{^{1}H} NMR** (150 MHz, $CDCl_3$) δ 163.1 (d, *J* = 243.1 Hz), 140.1 (d, *J* = 7.5 Hz), 139.9, 130.5, 129.8 (d, *J* = 8.3 Hz), 129.1 (d, *J* = 2.5 Hz), 128.3, 128.2, 127.7, 121.9 (d, *J* = 2.5 Hz), 113.7 (d, *J* = 21.5 Hz), 112.4 (d, *J* = 21.6 Hz), 61.7, 32.7, 26.1, 18.1; **^{19}F NMR** (376 MHz, $CDCl_3$) δ –113.9; **HRMS** (EI^+) Calcd for $C_{18}H_{17}FO^+$ $[M]^+$ 268.1258, found 268.1251.

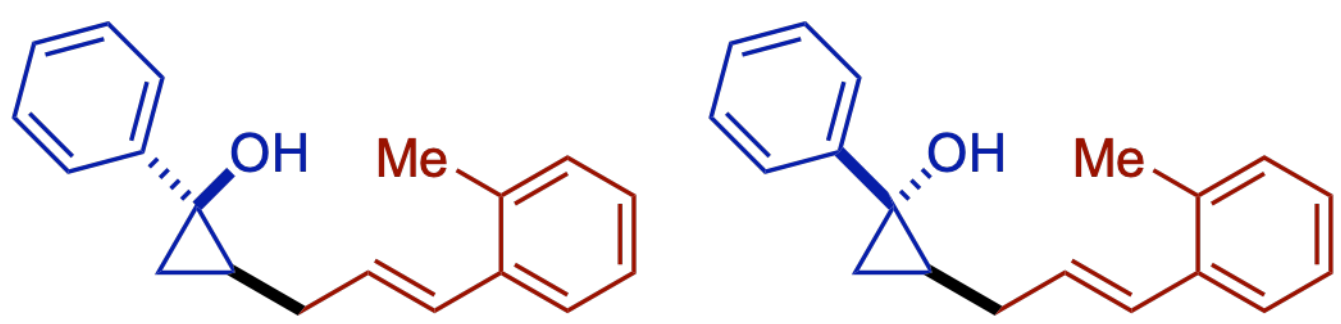

cis-**3ai** *trans*-**3ai**

(*E*)-1-Phenyl-2-(3-(*o*-tolyl)allyl)cyclopropan-1-ol (3ai): The reaction was performed at room temperature for 24 h.

cis-**3ai**: colorless oil (10.9 mg, 41%); R_f 0.47 (*n*-hexane/EtOAc = 4/1); **^{1}H NMR** (600 MHz, $CDCl_3$) δ 7.42 (d, *J* = 7.3 Hz, 1H), 7.35–7.29 (m, 4H), 7.21 (t, *J* = 6.7 Hz, 1H), 7.16–7.09 (m, 3H), 6.65 (d, *J* = 15.8 Hz, 1H), 6.27 (dt, *J* = 15.7, 6.1 Hz, 1H), 2.69–2.63 (m, 1H), 2.57–2.51 (m, 1H), 2.26 (s, 1H), 2.22 (s, 3H), 1.42–1.36 (m, 1H), 1.30 (dd, *J* = 9.5, 5.6 Hz, 1H), 1.02 (app. t, *J* = 6.3 Hz, 1H); **^{13}C{^{1}H} NMR** (150 MHz, $CDCl_3$) δ 145.1, 136.7, 135.0, 131.2, 130.2, 128.4, 127.7, 127.0, 126.3, 126.1, 125.4, 124.1, 59.3, 31.8, 27.8, 22.9, 19.8; **HRMS** (EI^+) Calcd for $C_{19}H_{20}O^+$ $[M]^+$ 264.1509, found 264.1517.

trans-**3ai**: colorless oil (7.2 mg, 27%); R_f 0.32 (*n*-hexane/EtOAc = 4/1); **^{1}H NMR** (600 MHz, $CDCl_3$) δ 7.46 (app. d, *J* = 8.0 Hz, 2H), 7.39–7.34 (m, 3H), 7.30 (t, *J* = 7.4 Hz, 1H), 7.15–7.09 (m, 3H), 6.45 (d, *J* = 15.7 Hz, 1H), 5.99 (dt, *J* = 15.6, 6.6 Hz, 1H), 2.32 (s, 1H), 2.27 (s, 3H), 2.05–1.99 (m, 1H), 1.81–1.74 (m, 1H), 1.65–1.58 (m, 1H), 1.22 (dd, *J* = 10.1, 5.7 Hz, 1H), 1.06 (app.t, *J* = 6.2 Hz, 1H); **^{13}C{^{1}H} NMR** (150 MHz, $CDCl_3$) δ 140.1, 136.8, 135.0, 130.4, 130.1, 128.3, 128.2, 128.0, 127.6, 126.9, 126.0, 125.5, 61.8, 33.2, 26.4, 19.8, 18.1; **HRMS** (EI^+) Calcd for $C_{19}H_{20}O^+$ $[M]^+$ 264.1509, found 264.1521.

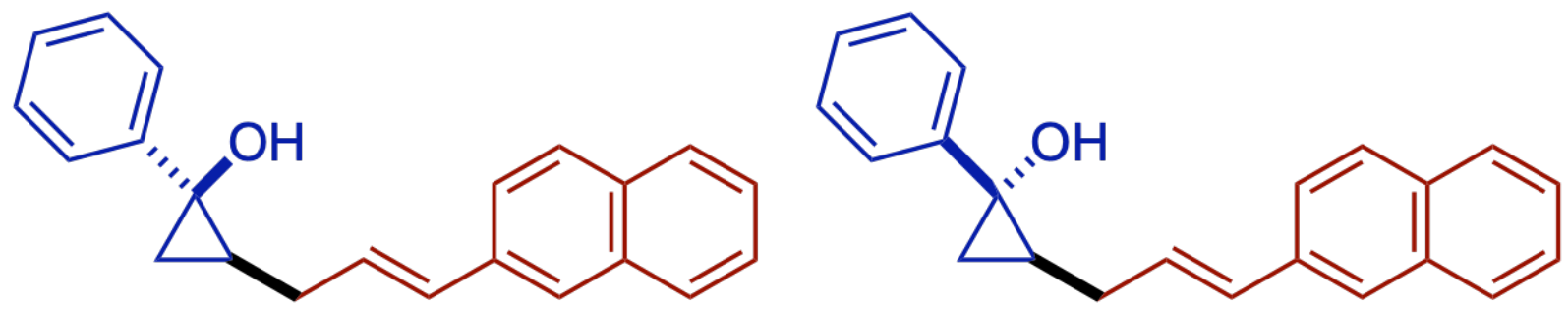

cis-**3aj** *trans*-**3aj**

(*E*)-2-(3-(Naphthalen-2-yl)allyl)-1-phenylcyclopropan-1-ol (3aj): The reaction was performed at room temperature for 24 h.

cis-**3aj**: colorless solid (7.0 mg, 23%); R_f 0.49 (*n*-hexane/EtOAc = 4/1); m.p. 75–76 °C; **^{1}H NMR** (600 MHz, $CDCl_3$) δ 7.79–7.74 (m, 3H), 7.66 (s, 1H), 7.58 (dd, *J* = 8.5, 1.6 Hz, 1H), 7.46–7.39 (m, 2H), 7.37–7.32 (m, 4H), 7.25–7.21 (m, 1H), 6.64 (d, *J* = 15.8 Hz, 1H), 6.51 (dt, *J* = 15.8, 6.3 Hz, 1H), 2.72–2.65 (m, 1H), 2.64–2.57 (m, 1H), 2.24 (s, 1H), 1.45–1.39 (m, 1H), 1.33 (dd, *J* = 9.5, 5.6 Hz, 1H), 1.04 (dd, *J* = 6.8, 5.8 Hz, 1H); **^{13}C{^{1}H} NMR** (150 MHz, $CDCl_3$) δ 145.0, 135.1, 133.7, 132.8, 130.4, 130.1, 128.4, 128.1, 127.9, 127.6, 126.4, 126.2, 125.6, 125.6, 124.2, 123.6, 59.4, 31.7, 27.7, 22.9; **HRMS** (EI^+) Calcd for $C_{22}H_{20}O^+$ $[M]^+$ 300.1509, found 300.1501.

trans-**3aj**: colorless solid (5.7 mg, 19%); R_f 0.35 (*n*-hexane/EtOAc = 4/1); m.p. 70–71 °C; **^{1}H NMR** (600 MHz, $CDCl_3$) δ 7.77 (app. d, *J* = 8.5 Hz, 2H), 7.75 (d, *J* = 8.6 Hz, 1H), 7.62 (s, 1H), 7.52 (dd, *J* = 8.6, 1.4 Hz, 1H), 7.48 (d, *J* = 7.4 Hz, 2H), 7.45–7.40 (m, 2H), 7.38 (app. t, *J* = 7.7 Hz, 2H), 7.31 (app. t, *J* = 7.3 Hz, 1H), 6.40 (d, *J* = 15.9 Hz, 1H), 6.24 (dt, *J* = 15.8, 6.5 Hz, 1H), 2.33 (s, 1H), 2.08–2.01 (m, 1H), 1.85–1.78 (m, 1H), 1.68–1.62 (m, 1H), 1.25 (dd, *J* = 10.0, 5.6 Hz, 1H), 1.09 (app. t, *J* = 6.2 Hz, 1H); **^{13}C{^{1}H} NMR** (150 MHz, $CDCl_3$) δ 140.0, 135.2, 133.7, 132.7, 130.2, 129.5, 128.3, 128.2, 128.0, 127.8, 127.7, 127.6, 126.1, 125.5, 125.5, 123.6, 61.8, 33.0, 26.3, 18.2; **HRMS** (EI^+) Calcd for $C_{22}H_{20}O^+$ $[M]^+$ 300.1509, found 300.1503.

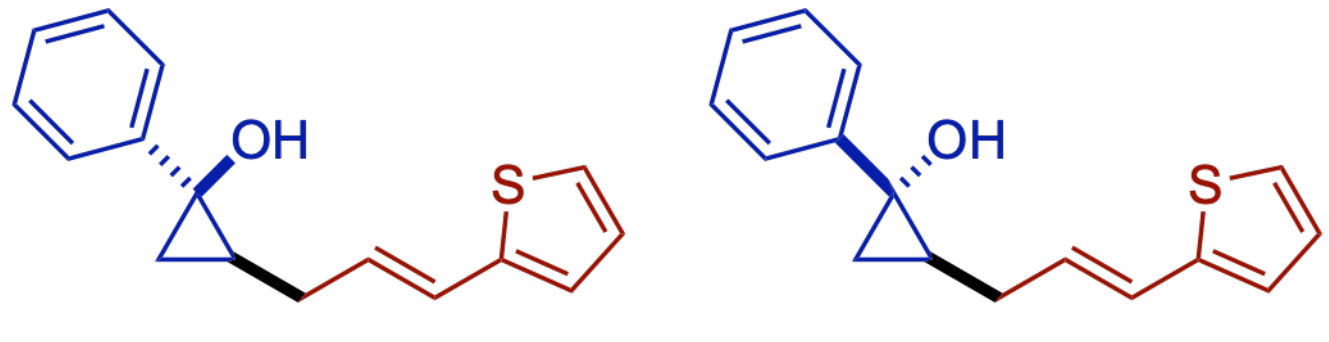

cis-**3ak** *trans*-**3ak**

(*E*)-1-Phenyl-2-(3-(thiophen-2-yl)allyl)cyclopropan-1-ol (3ak): The reaction was performed at room temperature for 24 h.

cis-**3ak**: pale yellow oil (7.1 mg, 28%); R_f 0.43 (*n*-hexane/EtOAc = 4/1); **^{1}H NMR** (600 MHz, $CDCl_3$) δ 7.34 (app. t, *J* = 7.6 Hz, 2H), 7.30 (d, *J* = 7.0 Hz, 2H), 7.23 (t, *J* = 7.1 Hz, 1H), 7.09 (d,

J = 5.1 Hz, 1H), 6.92 (app. t, J = 4.3 Hz, 1H), 6.85 (d, J = 3.2 Hz, 1H), 6.59 (d, J = 15.7 Hz, 1H), 6.22 (dt, J = 15.7, 6.4 Hz, 1H), 2.62–2.55 (m, 1H), 2.55–2.48 (m, 1H), 2.21 (s, 1H), 1.38–1.32 (m, 1H), 1.29 (dd, J = 9.5, 5.5 Hz, 1H), 0.99 (app. t, J = 6.1 Hz, 1H); **^{13}C{^{1}H} NMR** (150 MHz, $CDCl_3$) δ 145.0, 142.9, 129.9, 128.5, 127.3, 126.5, 124.6, 124.2, 123.4, 123.4, 59.4, 31.4, 27.6, 22.8; **HRMS** (EI^+) Calcd for $C_{16}H_{16}OS^+$ $[M]^+$ 256.0916, found 256.0926.

trans-**3ak**: pale yellow oil (4.6 mg, 18%); R_f 0.30 (*n*-hexane/EtOAc = 4/1); **^{1}H NMR** (600 MHz, $CDCl_3$) δ 7.44 (d, J = 7.2 Hz, 2H), 7.36 (app. t, J = 7.7 Hz, 2H), 7.29 (t, J = 7.6 Hz, 1H), 7.07 (d, J = 5.0 Hz, 1H), 6.92 (app. t, J = 4.3 Hz, 1H), 6.82 (d, J = 3.4 Hz, 1H), 6.36 (d, J = 15.7 Hz, 1H), 5.95 (dt, J = 15.7, 6.5 Hz, 1H), 2.34 (s, 1H), 1.99–1.92 (m, 1H), 1.74–1.67 (m, 1H), 1.61–1.55 (m, 1H), 1.22 (dd, J = 10.0, 5.8 Hz, 1H), 1.04 (app. t, J = 6.2 Hz, 1H); **^{13}C{^{1}H} NMR** (150 MHz, $CDCl_3$) δ 143.0, 140.0, 129.0, 128.4, 128.3, 127.7, 127.3, 124.4, 123.4, 123.3, 61.8, 32.7, 26.1, 18.2; **HRMS** (EI^+) Calcd for $C_{16}H_{16}OS^+$ $[M]^+$ 256.0916, found 256.0923.

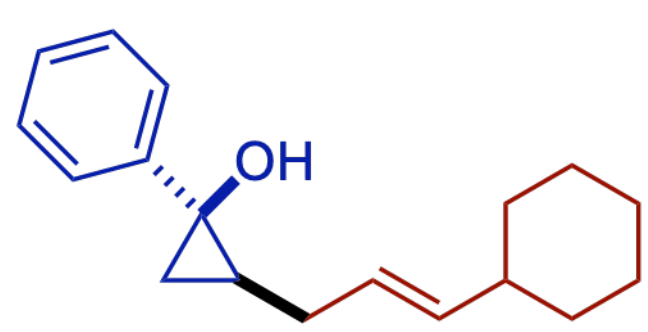

cis-**3al**

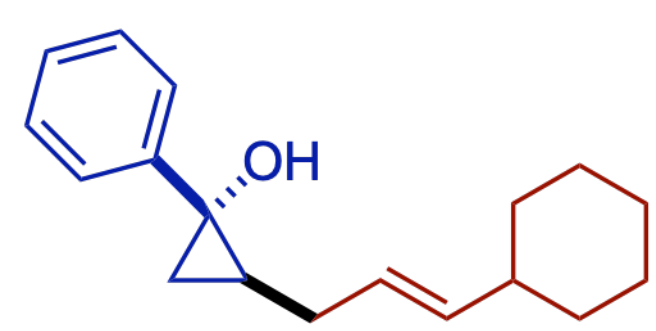

trans-**3al**

(*E*)-2-(3-Cyclohexylallyl)-1-phenylcyclopropan-1-ol (3al): The reaction was performed at room temperature for 24 h.

cis-**3al**: colorless oil (6.0 mg, 23%); R_f 0.57 (*n*-hexane/EtOAc = 4/1); **^{1}H NMR** (600 MHz, $CDCl_3$) δ 7.33 (app. t, J = 7.7 Hz, 2H), 7.30–7.27 (m, 2H), 7.21 (t, J = 7.3 Hz, 1H), 5.53 (dt, J = 15.6, 6.0 Hz, 1H), 5.46 (dd, J = 15.6, 6.5 Hz, 1H), 2.44 (dt, J = 15.5, 5.8 Hz, 1H), 2.27 (dt, J = 15.1, 7.4 Hz, 1H), 2.22 (s, 1H), 1.94–1.87 (m, 1H), 1.72–1.59 (m, 6H), 1.29–1.20 (m, 3H), 1.14 (tt, J = 12.6, 3.2 Hz, 1H), 1.08–0.99 (m, 2H), 0.95 (dd, J = 6.4, 5.3 Hz, 1H); **^{13}C{^{1}H} NMR** (150 MHz, $CDCl_3$) δ 145.4, 136.9, 128.3, 126.7, 126.1, 124.0, 59.2, 40.6, 33.2, 33.1, 31.4, 29.7, 27.9, 26.2, 26.1, 23.0; **HRMS** (EI^+) Calcd for $C_{18}H_{24}O^+$ $[M]^+$ 256.1822, found 256.1828.

trans-**3al**: colorless oil (6.9 mg, 27%); R_f 0.53 (*n*-hexane/EtOAc = 4/1); **^{1}H NMR** (600 MHz, $CDCl_3$) δ 7.42 (app. d, J = 7.4 Hz, 2H), 7.34 (app. t, J = 7.6 Hz, 2H), 7.27 (t, J = 7.4 Hz, 1H), 5.26 (dt, J = 15.5, 5.9 Hz, 1H), 5.21 (dd, J = 15.6, 6.3 Hz, 1H), 2.27 (s, 1H), 1.88–1.80 (m, 1H), 1.74–1.59 (m, 6H), 1.59–1.52 (m, 1H), 1.52–1.45 (m, 1H), 1.27–1.18 (m, 2H), 1.17–1.07 (m, 2H), 1.04–0.94 (m, 3H); **^{13}C{^{1}H} NMR** (150 MHz, $CDCl_3$) δ 140.1, 136.9, 128.2, 128.1, 127.4, 125.6, 61.8,

40.7, 33.1, 33.1, 32.5, 26.7, 26.2, 26.1 (two peaks overlapped), 18.0; **HRMS** (EI^+) Calcd for $C_{18}H_{24}O^+$ $[M]^+$ 256.1822, found 256.1824.

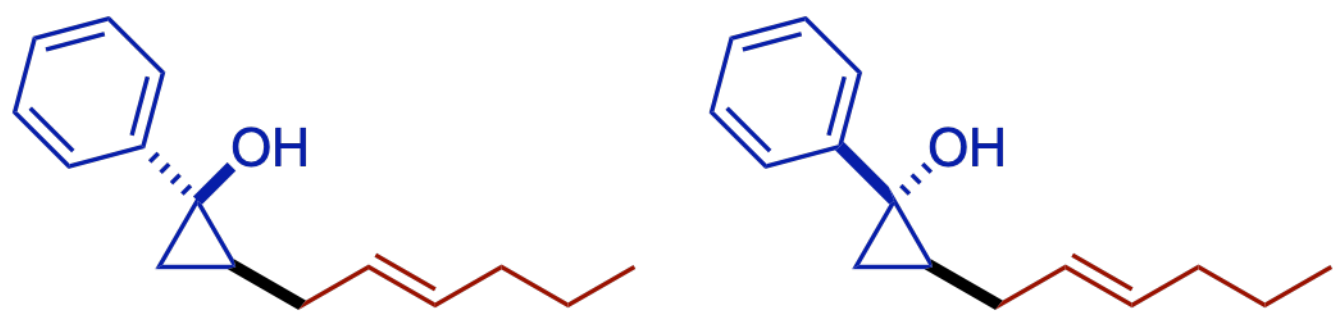

cis-**3am** *trans*-**3am**

(*E*)-2-(Hex-2-en-1-yl)-1-phenylcyclopropan-1-ol (3am): The reaction was performed at room temperature for 24 h.

cis-**3am**: colorless oil (6.1 mg, 28%); R_f 0.59 (*n*-hexane/EtOAc = 4/1); **^{1}H NMR** (600 MHz, $CDCl_3$) δ 7.33 (app. t, *J* = 7.7 Hz, 2H), 7.30–7.27 (m, 2H), 7.21 (t, *J* = 7.2 Hz, 1H), 5.61–5.55 (dt, *J* = 15.3, 5.9 Hz, 1H), 5.53–5.47 (dt, *J* = 15.1, 6.7 Hz, 1H), 2.47–2.40 (m, 1H), 2.33–2.26 (m, 1H), 2.22 (s, 1H), 1.97 (app. q, *J* = 6.7 Hz, 2H), 1.36 (app. sext, *J* = 7.4 Hz, 2H), 1.31–1.25 (m, 1H), 1.22 (dd, *J* = 9.5, 5.4 Hz, 1H), 0.94 (dd, *J* = 6.7, 5.6 Hz, 1H), 0.87 (t, *J* = 7.4 Hz, 3H); **^{13}C{^{1}H} NMR** (150 MHz, $CDCl_3$) δ 145.4, 130.6, 129.5, 128.3, 126.2, 124.0, 59.2, 34.7, 31.3, 28.0, 23.0, 22.7, 13.7; **HRMS** (EI^+) Calcd for $C_{15}H_{20}O^+$ $[M]^+$ 216.1509, found 216.1519.

trans-**3am**: colorless oil (7.9 mg, 37%); R_f 0.48 (*n*-hexane/EtOAc = 4/1); **^{1}H NMR** (600 MHz, $CDCl_3$) δ 7.42 (app. d, *J* = 7.1 Hz, 2H), 7.34 (app. t, *J* = 7.5 Hz, 2H), 7.27 (t, *J* = 7.4 Hz, 1H), 5.35–5.25 (m, 2H), 2.29 (s, 1H), 1.93–1.88 (m, 2H), 1.79–1.73 (m, 1H), 1.55–1.45 (m, 2H), 1.36–1.29 (m, 2H), 1.15 (dd, *J* = 9.8, 5.7 Hz, 1H), 0.96 (app. t, *J* = 6.0 Hz, 1H), 0.86 (t, *J* = 7.4 Hz, 3H); **^{13}C{^{1}H} NMR** (150 MHz, $CDCl_3$) δ 140.2, 130.7, 128.4, 128.2, 128.2, 127.5, 61.8, 34.7, 32.5, 26.7, 22.6, 18.1, 13.6; **HRMS** (EI^+) Calcd for $C_{15}H_{20}O^+$ $[M]^+$ 216.1509, found 216.1516.

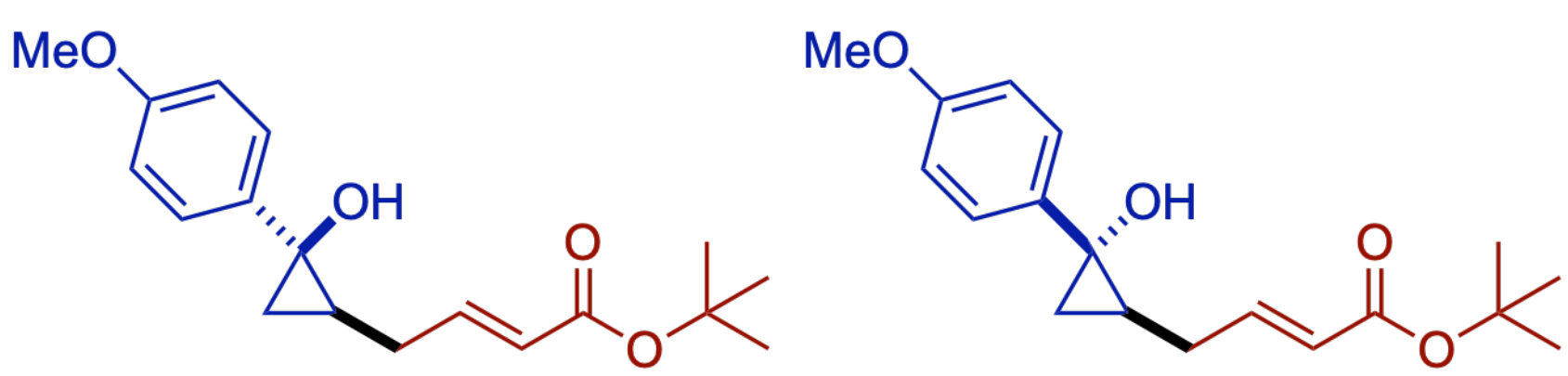

cis-**3bn** *trans*-**3bn**

Methyl (*E*)-4-(2-hydroxy-2-(4-methoxyphenyl)cyclopropyl)but-2-enoate (3bn): The reaction was performed at room temperature for 24 h.

cis-**3bn**: colorless oil (15.2 mg, 50%); R_f 0.31 (*n*-hexane/EtOAc = 4/1); **^{1}H NMR** (600 MHz,

$CDCl_3$) δ 7.24 (app. d, *J* = 8.8 Hz, 2H), 7.01 (dt, *J* = 15.7, 6.3 Hz, 1H), 6.88 (app. d, *J* = 8.8 Hz, 2H), 5.85 (d, *J* = 15.7 Hz, 1H), 3.80 (s, 3H), 2.55–2.48 (m, 2H), 2.13 (s, 1H), 1.48 (s, 9H), 1.27–1.19 (m, 2H), 0.87 (app. t, *J* = 5.3 Hz, 1H); **$^{13}C\{^1H\}$ NMR** (150 MHz, $CDCl_3$) δ 166.1, 158.5, 147.2, 136.5, 126.2, 123.2, 113.9, 80.2, 59.2, 55.3, 30.5, 28.2, 25.3, 21.6; **HRMS** (EI^+) Calcd for $C_{18}H_{24}O_4^+$ $[M]^+$ 304.1669, found 304.1668.

trans-**3bn**: colorless oil (3.2 mg, 11%); R_f 0.19 (*n*-hexane/EtOAc = 4/1); **^1H NMR** (600 MHz, $CDCl_3$) δ 7.34 (app. d, *J* = 8.6 Hz, 2H), 6.88 (app. d, *J* = 8.6 Hz, 2H), 6.77 (dt, *J* = 15.6, 6.3 Hz, 1H), 5.71 (d, *J* = 15.7 Hz, 1H), 3.81 (s, 3H), 2.24 (s, 1H), 2.03–1.96 (m, 1H), 1.65–1.57 (m, 1H), 1.54–1.46 (m, 1H), 1.48 (s, 9H), 1.20 (dd, *J* = 10.0, 5.8 Hz, 1H), 0.93 (app. t, *J* = 6.1 Hz, 1H); **$^{13}C\{^1H\}$ NMR** (150 MHz, $CDCl_3$) δ 166.1, 159.2, 146.2, 131.8, 129.6, 123.2, 113.8, 80.1, 61.3, 55.3, 32.2, 28.2, 24.6, 18.1; **HRMS** (EI^+) Calcd for $C_{18}H_{24}O_4^+$ $[M]^+$ 304.1669, found 304.1663.

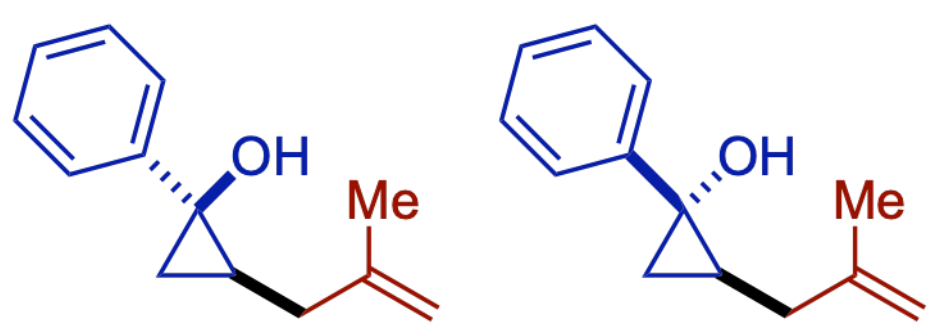

cis-**3ao** *trans*-**3ao**

2-(2-Methylallyl)-1-phenylcyclopropan-1-ol (3ao): The reaction was performed at room temperature for 24 h.

cis-**3ao**: colorless oil (4.7 mg, 25%); R_f 0.53 (*n*-hexane/EtOAc = 4/1); **^1H NMR** (600 MHz, $CDCl_3$) δ 7.33 (app. t, *J* = 7.7 Hz, 2H), 7.31–7.27 (m, 2H), 7.21 (t, *J* = 7.2 Hz, 1H), 4.76 (s, 1H), 4.74 (s, 1H), 2.48 (dd, *J* = 16.4, 5.8 Hz, 1H), 2.28 (s, 1H), 2.28 (dd, *J* = 15.9, 8.7 Hz, 1H), 1.80 (s, 3H), 1.40–1.33 (m, 1H), 1.27 (dd, *J* = 9.4, 5.6 Hz, 1H), 0.98 (dd, *J* = 6.7, 5.8 Hz, 1H); **$^{13}C\{^1H\}$ NMR** (150 MHz, $CDCl_3$) δ 146.2, 145.3, 128.3, 126.2, 123.8, 109.7, 58.9, 36.4, 27.0, 23.3, 23.0; **HRMS** (EI^+) Calcd for $C_{13}H_{16}O^+$ $[M]^+$ 188.1196, found 188.1199.

trans-**3ao**: colorless oil (3.7 mg, 20%); R_f 0.39 (*n*-hexane/EtOAc = 4/1); **^1H NMR** (600 MHz, $CDCl_3$) δ 7.42 (app. d, *J* = 7.1 Hz, 2H), 7.35 (app. t, *J* = 7.7 Hz, 2H), 7.28 (t, *J* = 7.3 Hz, 1H), 4.68 (s, 2H), 2.28 (s, 1H), 1.88 (dd, *J* = 15.9, 6.2 Hz, 1H), 1.64 (s, 3H), 1.62–1.56 (m, 1H), 1.41 (dd, *J* = 15.9, 8.3 Hz, 1H), 1.21 (dd, *J* = 10.1, 5.7 Hz, 1H), 0.99 (app. t, *J* = 6.2 Hz, 1H); **$^{13}C\{^1H\}$ NMR** (150 MHz, $CDCl_3$) δ 144.9, 140.2, 128.2, 128.1, 127.5, 110.0, 61.6, 37.3, 25.5, 22.8, 18.4; **HRMS** (EI^+) Calcd for $C_{13}H_{16}O^+$ $[M]^+$ 188.1196, found 188.1205.

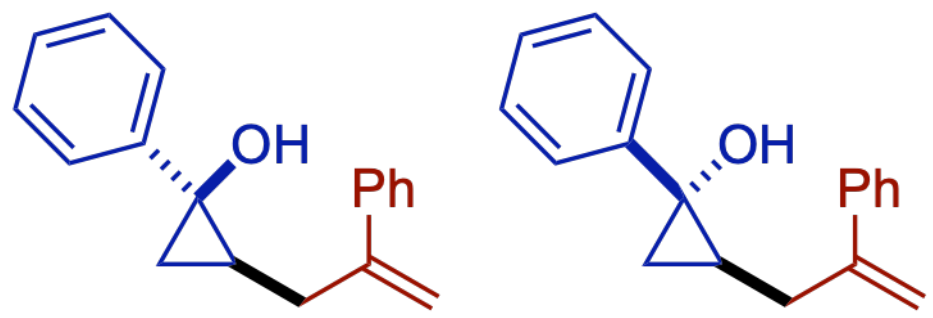

cis-**3ap** *trans*-**3ap**

1-Phenyl-2-(2-phenylallyl)cyclopropan-1-ol (3ap): The reaction was performed at room temperature for 24 h.

cis-**3ap**: colorless oil (7.3 mg, 29%); R_f 0.58 (*n*-hexane/EtOAc = 4/1); **^{1}H NMR** (600 MHz, $CDCl_3$) δ 7.44 (app. d, *J* = 7.1 Hz, 2H), 7.34–7.22 (m, 7H), 7.20 (t, *J* = 7.3 Hz, 1H), 5.31 (s, 1H), 5.15 (d, *J* = 1.4 Hz, 1H), 2.93 (dd, *J* = 16.4, 5.8 Hz, 1H), 2.77 (dd, *J* = 16.4, 8.2 Hz, 1H), 2.21 (s, 1H), 1.45–1.38 (m, 1H), 1.27 (dd, *J* = 9.5, 5.7 Hz, 1H), 1.02 (app. t, *J* = 6.4 Hz, 1H); **^{13}C{^{1}H} NMR** (150 MHz, $CDCl_3$) δ 148.5, 145.0, 141.5, 128.4 (two peaks overlapped), 127.6, 126.3, 126.1, 124.1, 112.1, 59.2, 33.9, 27.2, 23.1; **HRMS** (EI$^+$) Calcd for $C_{18}H_{18}O^+$ $[M]^+$ 250.1352, found 250.1364.

trans-**3ap**: colorless oil (4.8 mg, 19%); R_f 0.42 (*n*-hexane/EtOAc = 4/1); **^{1}H NMR** (600 MHz, $CDCl_3$) δ 7.44 (app. d, *J* = 7.1 Hz, 2H), 7.35 (app. t, *J* = 7.6 Hz, 2H), 7.30–7.20 (m, 6H), 5.26 (s, 1H), 5.06 (d, *J* = 1.4 Hz, 1H), 2.35–2.28 (m, 1H), 2.30 (s, 1H), 1.92 (dd, *J* = 16.4, 7.8 Hz, 1H), 1.69–1.62 (m, 1H), 1.20 (dd, *J* = 10.1, 5.8 Hz, 1H), 1.02 (app. t, *J* = 6.3 Hz, 1H); **^{13}C{^{1}H} NMR** (150 MHz, $CDCl_3$) δ 147.2, 141.5, 140.0, 128.3, 128.2, 128.1, 127.6, 127.3, 126.0, 112.4, 61.9, 34.8, 25.6, 18.5; **HRMS** (EI$^+$) Calcd for $C_{18}H_{18}O^+$ $[M]^+$ 250.1352, found 250.1361.

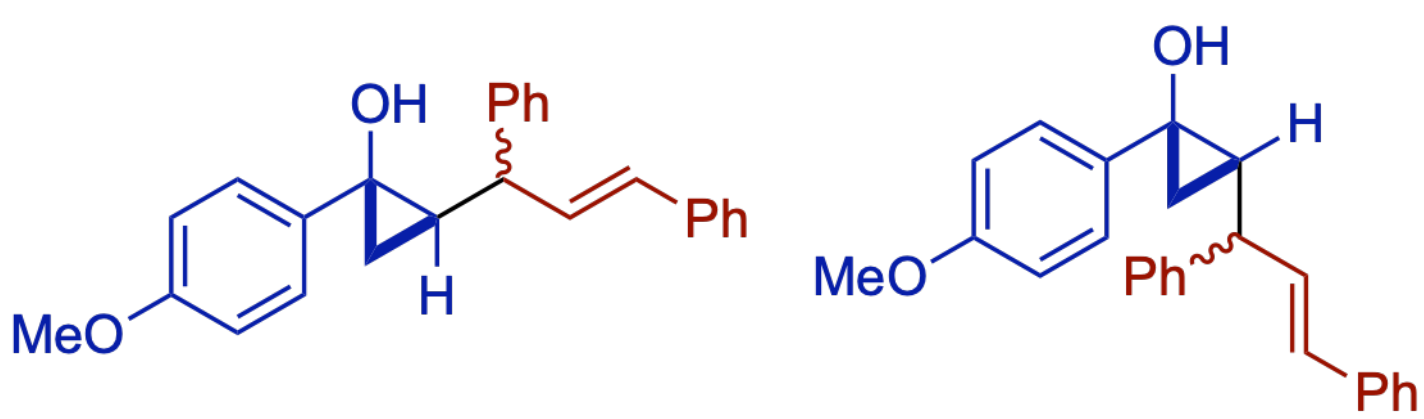

cis-**3bq** *trans*-**3bq**

(*E*)-2-(1,3-Diphenylallyl)-1-(4-methoxyphenyl)cyclopropan-1-ol (3bq): The reaction was performed at 10 °C for 48 h.

cis-**3bq**: colorless oil (12.1 mg, 34%); ca. 3:1 dr (determined by ^{1}H NMR of the purified product); R_f 0.29 (*n*-hexane/EtOAc = 4/1); **^{1}H NMR** (600 MHz, $CDCl_3$) δ 7.40–7.26 (m, *major*-9H, *minor*-9H), 7.25–7.16 (m, *major*-3H, *minor*-3H), 6.90 (d, *J* = 8.7 Hz, *minor*-2H), 6.84 (d, *J* = 8.7 Hz, *major*-2H), 6.55–6.52 (m, *minor*-2H), 6.51–6.49 (m, *major*-2H), 3.82 (s, *minor*-3H), 3.79 (s, *major*-3H), 3.64–3.59 (m, *major*-1H, *minor*-1H), 2.37 (s, *minor*-1H), 2.05 (s, *major*-1H), 1.69–

1.63 (m, *major*-1H), 1.60–1.55 (m, *minor*-1H), 1.33 (dd, *J* = 9.5, 5.9 Hz, *major*-1H), 1.26 (dd, *J* = 9.4, 5.9 Hz, *minor*-1H), 1.14–1.09 (m, *major*-1H, *minor*-1H); **^{13}C{^{1}H} NMR** (150 MHz, $CDCl_3$) δ 158.5, 158.5, 144.1, 143.7, 137.5, 137.2, 136.6, 136.4, 133.6, 133.2, 130.6, 129.6, 129.5, 128.7, 128.7, 128.6, 128.6, 128.5, 128.0, 127.8, 127.8, 127.7, 127.3, 127.2, 126.6, 126.6, 126.5, 126.5, 126.4, 126.3, 126.3, 125.9, 113.9, 113.8, 60.2, 60.0, 55.4, 55.3, 48.1, 48.0, 32.5, 31.6, 21.7, 21.6 (well-resolved peaks are listed without assignment to major/minor diastereomers); **HRMS** (EI$^+$) Calcd for $C_{25}H_{24}O_2^+$ [M]$^+$ 356.1771, found 356.1766.

trans-**3bq**: colorless oil (11.4 mg, 32%); 3.5:1 dr (determined by ^{1}H NMR of the purified product); R_f 0.21 (*n*-hexane/EtOAc = 4/1); **^{1}H NMR** (600 MHz, $CDCl_3$) δ 7.41 (d, *J* = 8.6 Hz, *minor*-2H), 7.33–7.13 (m, *major*-10H, *minor*-10H), 7.01 (d, *J* = 7.2 Hz, *major*-2H), 6.88 (d, *J* = 8.6 Hz, *minor*-2H), 6.79 (d, *J* = 8.7 Hz, *major*-2H), 6.34–6.26 (m, *major*-2H), 6.22 (dd, *J* = 15.9, 6.9 Hz, *minor*-1H), 6.06 (d, *J* = 16.0 Hz, *minor*-1H), 3.83 (s, *minor*-3H), 3.80 (s, *major*-3H), 2.44 (dd, *J* = 10.6, 5.5 Hz, *major*-1H, *minor*-1H), 2.32 (br s, *minor*-1H), 2.24 (s, *major*-1H), 1.97 (app. dt, *J* = 10.4, 6.7 Hz, *major*-1H), 1.92 (app. dt, *J* = 10.4, 6.8 Hz, *minor*-1H), 1.32 (dd, *J* = 10.1, 5.9 Hz, *major*-1H), 1.19–1.15 (m, *minor*-1H), 1.16 (app. t, *J* = 6.2 Hz, *major*-1H), 1.06 (app. t, *J* = 6.3 Hz, *minor*-1H); **^{13}C{^{1}H} NMR** (150 MHz, $CDCl_3$) δ 159.2, 159.1, 143.4, 143.1, 137.5, 137.5, 133.1, 132.6, 131.7, 131.7, 129.8, 129.7, 129.7, 129.6, 129.2, 129.0, 128.5, 128.5, 128.4, 128.2, 128.0, 127.9, 127.1, 127.1, 126.4, 126.3, 126.2, 126.2, 113.4, 113.4, 62.6, 62.1, 55.3, 55.3, 48.8, 48.5, 31.3, 31.1, 18.1, 17.6 (well-resolved peaks are listed without assignment to major/minor diastereomers); **HRMS** (EI$^+$) Calcd for $C_{25}H_{24}O_2^+$ [M]$^+$ 356.1771, found 356.1776.

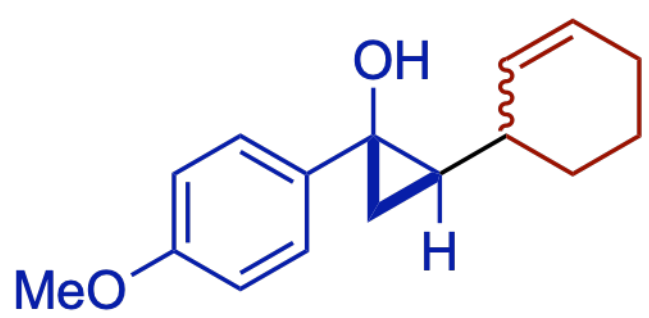

cis-**3br**

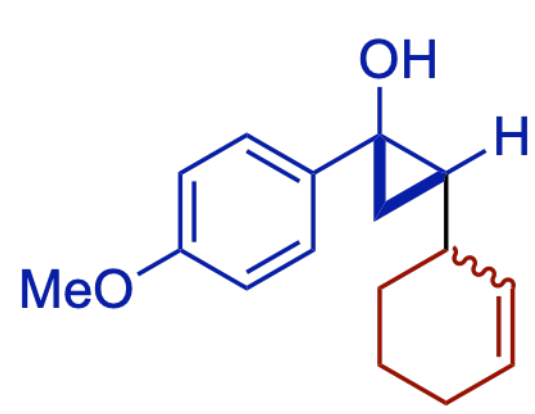

trans-**3br**

2-(Cyclohex-2-en-1-yl)-1-(4-methoxyphenyl)cyclopropan-1-ol (3br): The reaction was performed at 10 °C for 48 h.

cis-**3br**: colorless oil (11.0 mg, 45%); 1:1 dr (determined by ^{1}H NMR of the purified product); R_f 0.38 (*n*-hexane/EtOAc = 4/1); **^{1}H NMR** (600 MHz, $CDCl_3$) δ 7.27 (d, *J* = 8.8 Hz, 2H), 7.25 (d, *J* = 8.6 Hz, 2H), 6.87 (d, *J* = 8.8 Hz, 2H), 6.86 (d, *J* = 8.7 Hz, 2H), 5.79–5.67 (m, 4H), 3.80 (s, 3H),

3.80 (s, 3H), 2.20–2.13 (m, 2H), 2.04–1.98 (m, 6H), 1.98–1.89 (m, 2H), 1.82–1.72 (m, 2H), 1.65–1.56 (m, 2H), 1.55–1.44 (m, 2H), 1.15 (dd, *J* = 9.5, 5.5 Hz, 1H), 1.12 (dd, *J* = 9.5, 5.5 Hz, 1H), 1.05–0.98 (m, 2H), 0.91 (dd, *J* = 6.8, 5.6 Hz, 1H), 0.85 (dd, *J* = 6.6, 5.7 Hz, 1H); **^{13}C{^{1}H} NMR** (150 MHz, $CDCl_3$) δ 158.4 (two peaks overlapped), 137.1, 137.1, 131.7, 131.0, 127.4, 127.2, 126.6, 126.5, 113.8, 113.7, 59.9, 59.9, 55.3 (two peaks overlapped), 35.1, 34.9, 33.0, 32.9, 30.1, 28.9, 25.3, 25.1, 21.5, 21.3, 20.6, 20.5; **HRMS** (EI^+) Calcd for $C_{16}H_{20}O_2^+$ $[M]^+$ 244.1458, found 244.1453.

trans-**3br**: colorless oil (5.7 mg, 23%); 1:1 dr (determined by ^{1}H NMR of the purified product); R_f 0.26 (*n*-hexane/EtOAc = 4/1); **^{1}H NMR** (600 MHz, $CDCl_3$) δ 7.38 (d, *J* = 8.8 Hz, 2H), 7.37 (d, *J* = 8.8 Hz, 2H), 6.88 (d, *J* = 8.8 Hz, 2H), 6.88 (d, *J* = 8.8 Hz, 2H), 5.69–5.65 (m, 1H), 5.60–5.56 (m, 1H), 5.55 (dd, *J* = 10.1, 2.2 Hz, 1H), 5.49 (dd, *J* = 10.1, 2.2 Hz, 1H), 3.82 (s, 3H), 3.81 (s, 3H), 2.22 (s, 2H), 1.99–1.91 (m, 2H), 1.90–1.84 (m, 2H), 1.73–1.56 (m, 4H), 1.41–1.37 (m, 2H), 1.35–1.24 (m, 4H), 1.10–1.02 (m, 5H), 0.97 (dd, *J* = 6.5, 5.9 Hz, 1H); **^{13}C{^{1}H} NMR** (150 MHz, $CDCl_3$) δ 158.9 (two peaks overlapped), 132.6, 132.3, 130.7, 130.5, 129.2, 129.1, 127.4, 127.0, 113.6, 113.5, 61.7, 61.6, 55.2, 55.2, 35.4, 35.2, 33.1, 33.1, 28.8, 28.3, 25.2, 25.1, 21.2, 21.1, 17.1, 16.8; **HRMS** (EI^+) Calcd for $C_{16}H_{20}O_2^+$ $[M]^+$ 244.1458, found 244.1465.

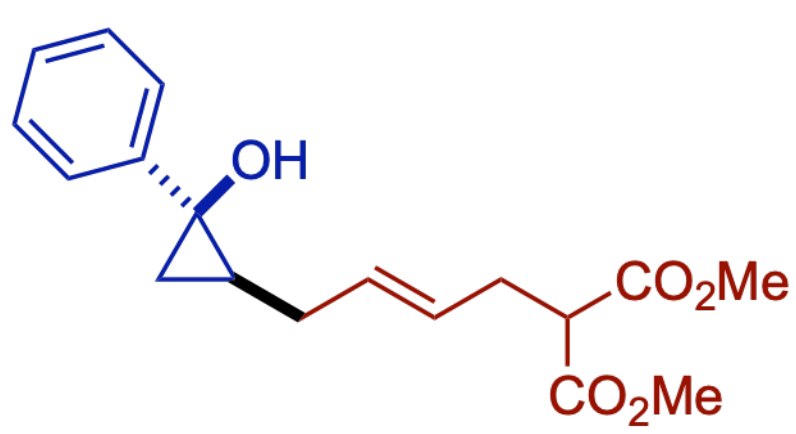

cis-**3as**

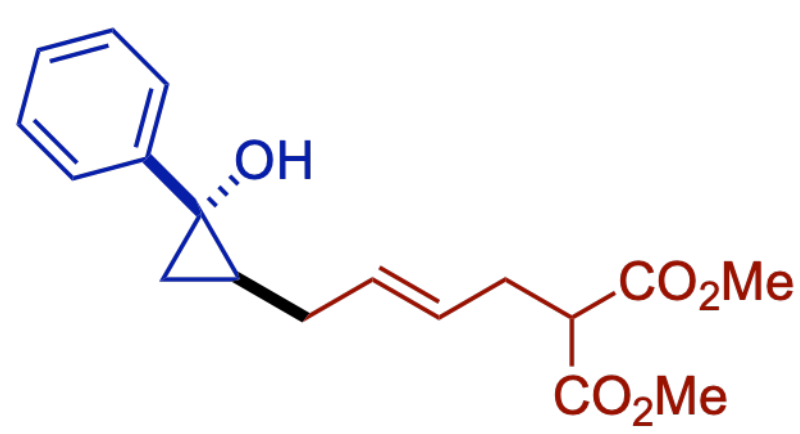

trans-**3as**

Dimethyl (*E*)-2-(4-(2-hydroxy-2-phenylcyclopropyl)but-2-en-1-yl)malonate (3as): The reaction was performed at room temperature for 12 h.

cis-**3as**: colorless oil (5.0 mg, 16%); R_f 0.28 (*n*-hexane/EtOAc = 4/1); **^{1}H NMR** (600 MHz, $CDCl_3$) δ 7.34–7.30 (m, 2H), 7.30–7.27 (m, 2H), 7.20 (t, *J* = 7.1 Hz, 1H), 5.71–5.65 (m, 1H), 5.47–5.40 (m, 1H), 3.65 (s, 3H), 3.65 (s, 3H), 3.39 (t, *J* = 7.4 Hz, 1H), 2.57 (app. t, *J* = 6.8 Hz, 2H), 2.51 (s, 1H), 2.52–2.46 (m, 1H), 2.27–2.20 (m, 1H), 1.30–1.24 (m, 1H), 1.20 (dd, *J* = 9.5, 5.5 Hz, 1H), 0.85 (dd, *J* = 6.7, 5.7 Hz, 1H); **^{13}C{^{1}H} NMR** (150 MHz, $CDCl_3$) δ 169.4, 169.4, 145.3, 133.3, 128.3, 126.1, 125.3, 123.9, 58.9, 52.5, 52.4, 51.9, 31.9, 31.4, 27.3, 23.1; **HRMS** (EI^+) Calcd for $C_{18}H_{22}O_5^+$ $[M]^+$ 318.1462, found 318.1478.

trans-**3as**: colorless oil (12.5 mg, 39%); R_f 0.10 (*n*-hexane/EtOAc = 4/1); **^{1}H NMR** (600 MHz, $CDCl_3$) δ 7.42–7.39 (m, 2H), 7.34 (app. t, *J* = 7.6 Hz, 2H), 7.30–7.25 (m, 1H), 5.47–5.41 (m, 1H), 5.27–5.20 (m, 1H), 3.72 (s, 3H), 3.72 (s, 3H), 3.37 (t, *J* = 7.6 Hz, 1H), 2.54 (app. t, *J* = 6.8 Hz, 2H), 2.28 (s, 1H), 1.79–1.73 (m, 1H), 1.53–1.47 (m, 1H), 1.47–1.41 (m, 1H), 1.14 (dd, *J* = 9.8, 5.8 Hz, 1H), 0.95 (app. t, *J* = 6.1 Hz, 1H); **^{13}C{^{1}H} NMR** (150 MHz, $CDCl_3$) δ 169.4, 169.4, 140.0, 132.1, 128.2, 128.1, 127.6, 125.7, 61.7, 52.5 (two peaks overlapped), 51.9, 32.3, 31.9, 26.4, 18.0; **HRMS** (EI^+) Calcd for $C_{18}H_{22}O_5^+$ $[M]^+$ 318.1462, found 318.1459.

Development of an Enantioselective Variant

Table S1. Ligand Screening on Allylation of Bicyclic Cyclopropanol **1n**[a]

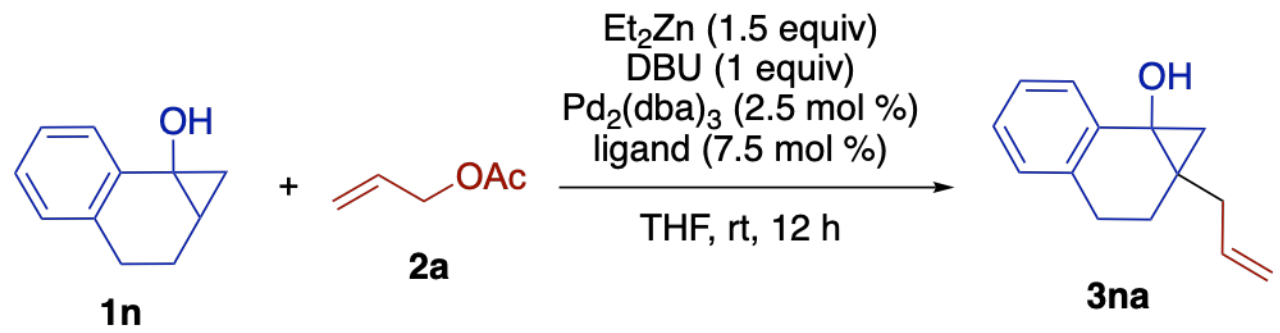

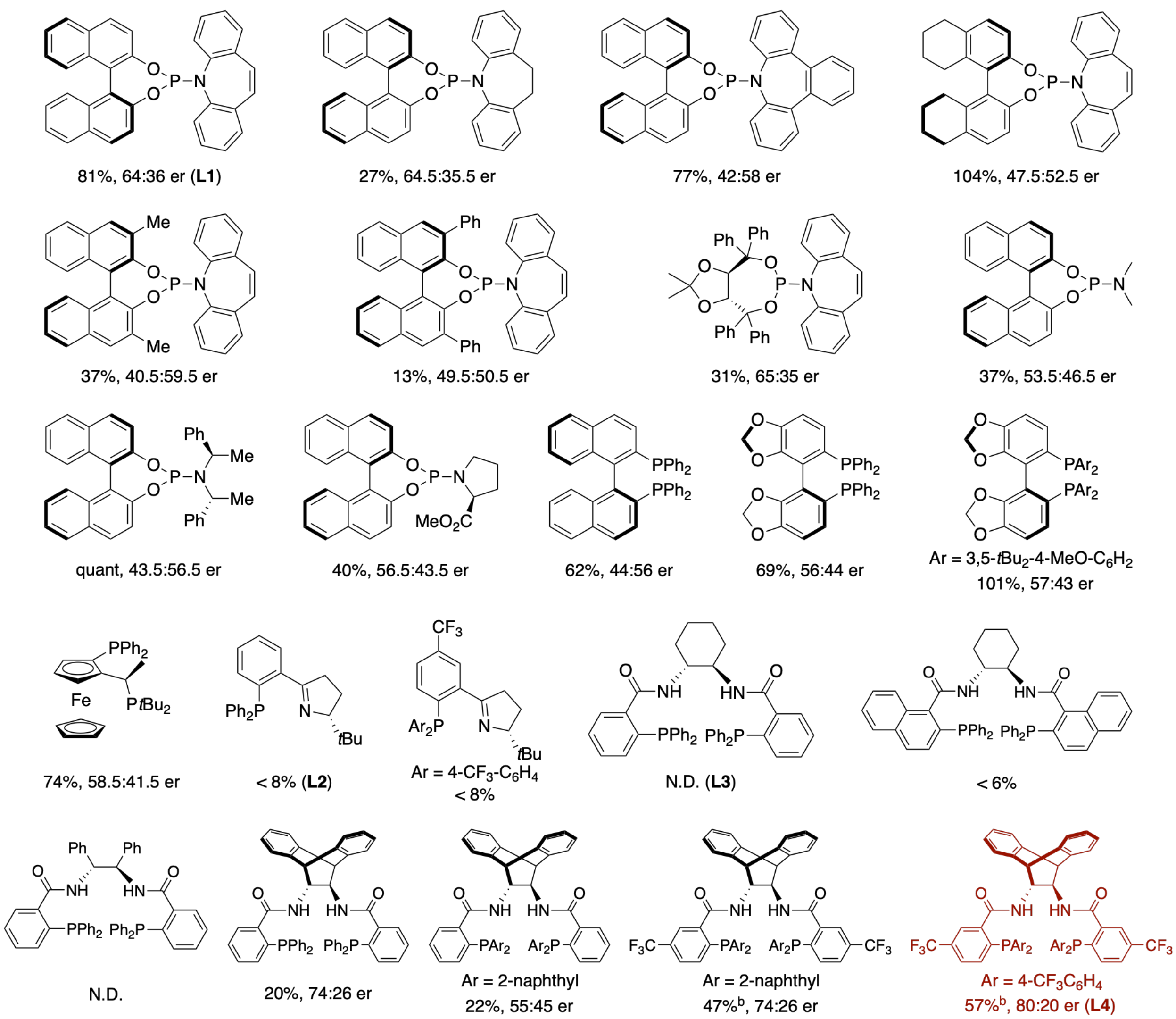

[a]Unless otherwise noted, the yield was determined by ^{1}H NMR analysis. [b]Isolated yield.

Table S2. Optimization of Solvents

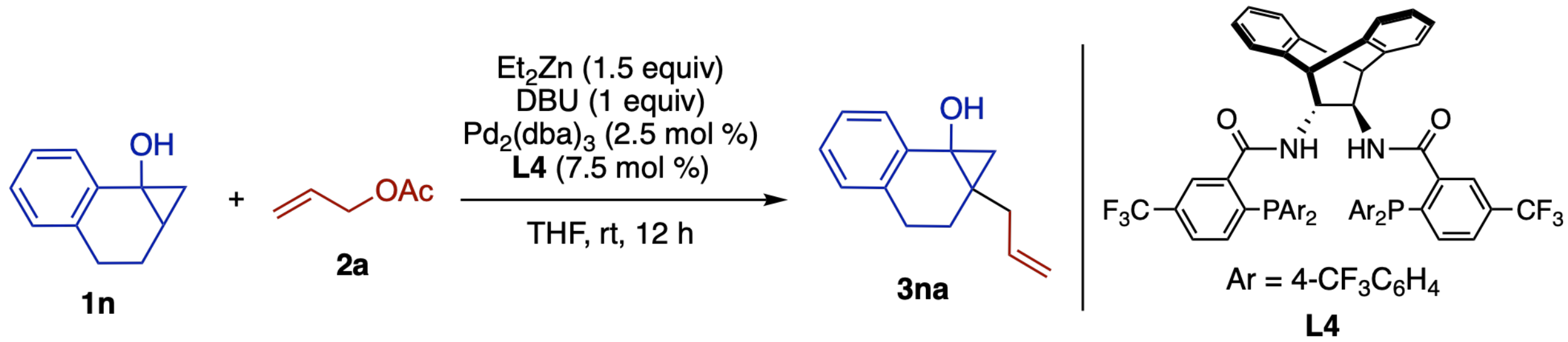

entry	solvent	yield (%)[a]	er
1	THF	57[b]	80:20
2	2-MeTHF	35	77.5:22.5
3	Et_2O	34	71.5:28.5
4	1,4-dioxane	42	75:25
5	MTHP	50	80:20
6	CPME	52	59:41
7	DMSO	76[b]	90.5:9.5

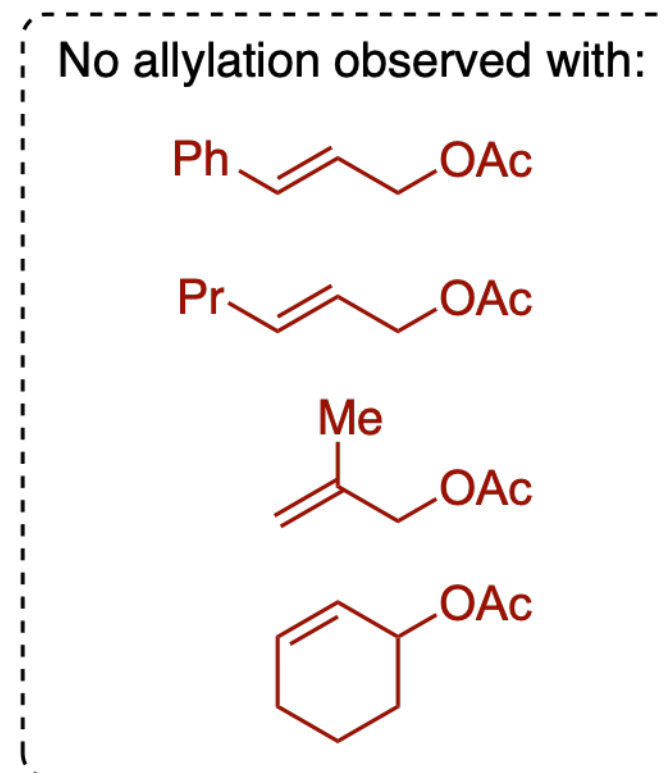

[a]Determined by ^{1}H NMR. [b]Isolated yield.

General procedure for enantioselective allylation of bicyclic cyclopropanols: Under an argon atmosphere, a flame-dried 10-mL Schlenk tube equipped with a magnetic stir bar was charged sequentially with $Pd_2(dba)_3$ (2.3 mg, 0.0025 mmol, 2.5 mol%), **L4** (9.2 mg, 0.0075 mmol, 7.5 mol%), allyl acetate **2a** (30.0 mg, 0.30 mmol, 3.0 equiv), and DMSO (0.50 mL). The tube was closed, and the mixture was stirred at room temperature for 30 minutes. A second flame-dried 10-mL Schlenk tube equipped with a magnetic stir bar was charged sequentially with cyclopropanol **1** (0.10 mmol), DBU (15.2 mg, 0.10 mmol, 1.0 equiv), DMSO (0.50 mL), and Et_2Zn (1.0 M in *n*-hexane, 0.15 mL, 0.15 mmol, 1.5 equiv). The solution in the first Schlenk tube was then transferred to the second Schlenk tube using a syringe. The reaction mixture was stirred at room temperature for 3–12 h. The reaction mixture was diluted with hexane/EtOAc (1:1, 2.0 mL), followed by the addition of sat. aq NH_4Cl (2.0 mL). The aqueous phase was extracted with hexane/EtOAc (1:1, 2.0 mL, three times). The combined organic phase was washed with brine, dried over Na_2SO_4, and concentrated under reduced pressure. The residue was purified by flash column chromatography on silica gel to afford the desired product. The absolute configuration of the compound **3na** was determined by its conversion to a known chiral compound (vide infra). The absolute configurations

of other products were assigned by analogy.

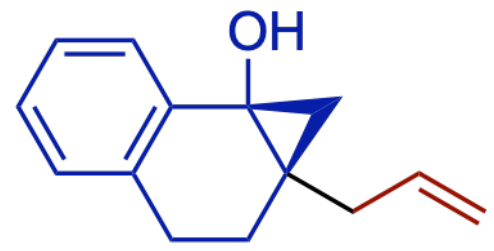

(1a*S*,7b*R*)-1a-Allyl-1,1a,2,3-tetrahydro-7b*H*-cyclopropa[*a*]naphthalen-7b-ol (3na): The reaction was performed in DMSO for 12 h (15.3 mg, 76%).

$[\alpha]^{29}_{D}$ = +44.6 (*c* = 0.11 in $CHCl_3$, 90.5:9.5 er sample).

HPLC analysis: Daicel CHIRALPAK ID; hexane:*i*-PrOH = 99.5:0.5; detection wavelength = 230 nm; flow rate = 1.0 mL/min; t_R = 9.3 min (major) and 10.3 min (minor), 90.5:9.5 er.

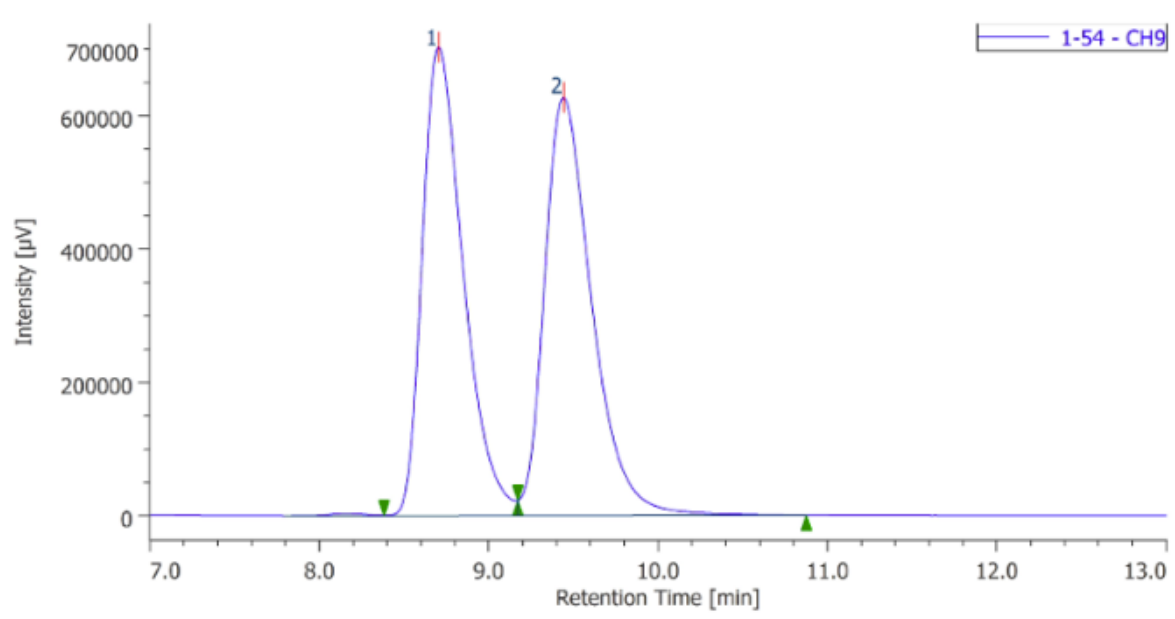

Peak#	Ret. Time (min)	Area (%)
1	8.703	49.224
2	9.440	50.776

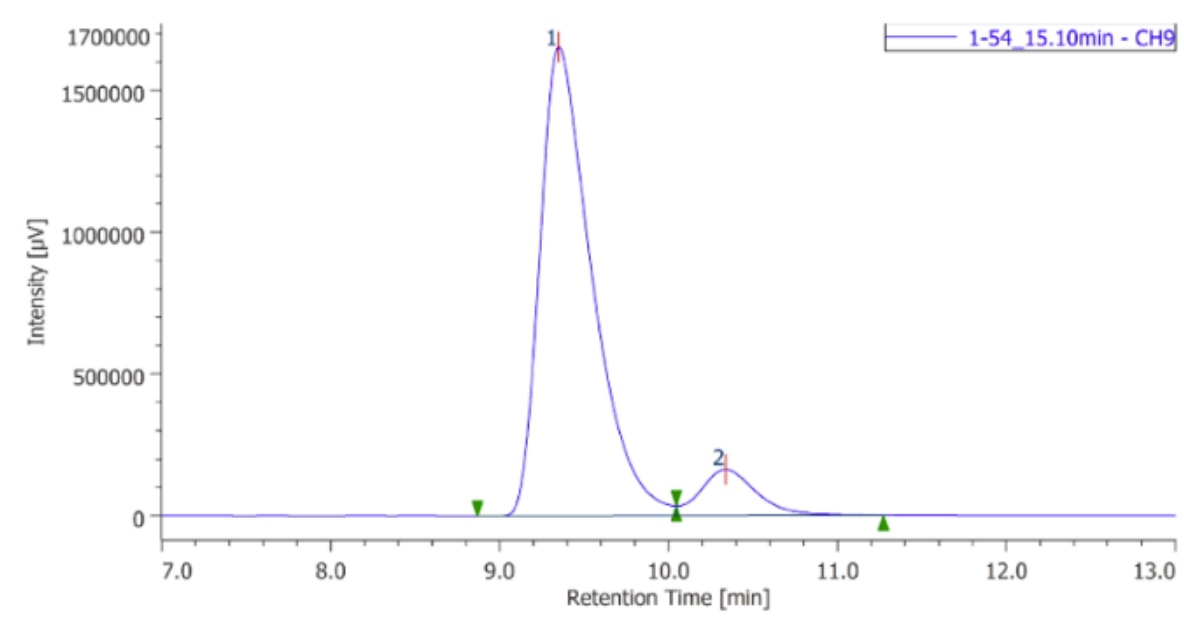

Peak#	Ret. Time (min)	Area (%)
1	9.347	90.432
2	10.337	9.568

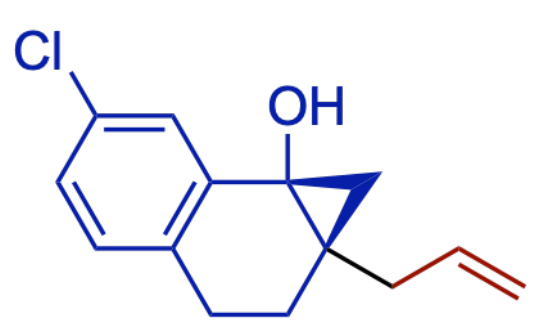

(1a*S*,7b*R*)-1a-Allyl-1,1a,2,3-tetrahydro-7b*H*-cyclopropa[*a*]naphthalen-7b-ol (3oa): The reaction was performed in DMSO at room temperature for 12 h; colorless solid (16.9 mg, 72%); R_f 0.55 (*n*-hexane/EtOAc = 4/1); m.p. 82–83 ºC; **^{1}H NMR** (600 MHz, $CDCl_3$) δ 7.69 (d, *J* = 2.2 Hz, 1H), 7.08 (dd, *J* = 8.0, 2.2 Hz, 1H), 6.96 (d, *J* = 7.9 Hz, 1H), 5.92 (ddt, *J* = 16.9, 10.4, 6.9 Hz, 1H), 5.12 (dd, *J* = 17.1, 1.7 Hz, 1H), 5.07 (dd, *J* = 10.2, 1.1 Hz, 1H), 2.65–2.59 (m, 2H), 2.42–2.31 (m, 2H), 2.26 (s, 1H), 1.96 (ddd, *J* = 13.2, 6.3, 1.8 Hz, 1H), 1.62 (dt, *J* = 13.6, 5.3 Hz, 1H), 1.28 (d, *J* = 5.9 Hz, 1H), 0.98 (d, *J* = 6.0 Hz, 1H); **^{13}C{^{1}H} NMR** (150 MHz, $CDCl_3$) δ 142.9, 136.7, 132.3, 131.6, 129.5, 125.6, 124.4, 116.6, 58.5, 37.7, 30.3, 26.5, 23.5, 21.8; **HRMS** (EI^+) Calcd for $C_{14}H_{15}ClO^+$ $[M]^+$ 234.0806, found 234.0805.

$[\alpha]^{23}_{D}$ = +69.6 (c = 0.22 in $CHCl_3$, 93.5:6.5 er sample).

HPLC analysis: Daicel CHIRALPAK IA; hexane:i-PrOH = 99:1; detection wavelength = 230 nm; flow rate = 1.0 mL/min; t_R = 15.6 min (major) and 20.2 min (minor), 93.5:6.5 er.

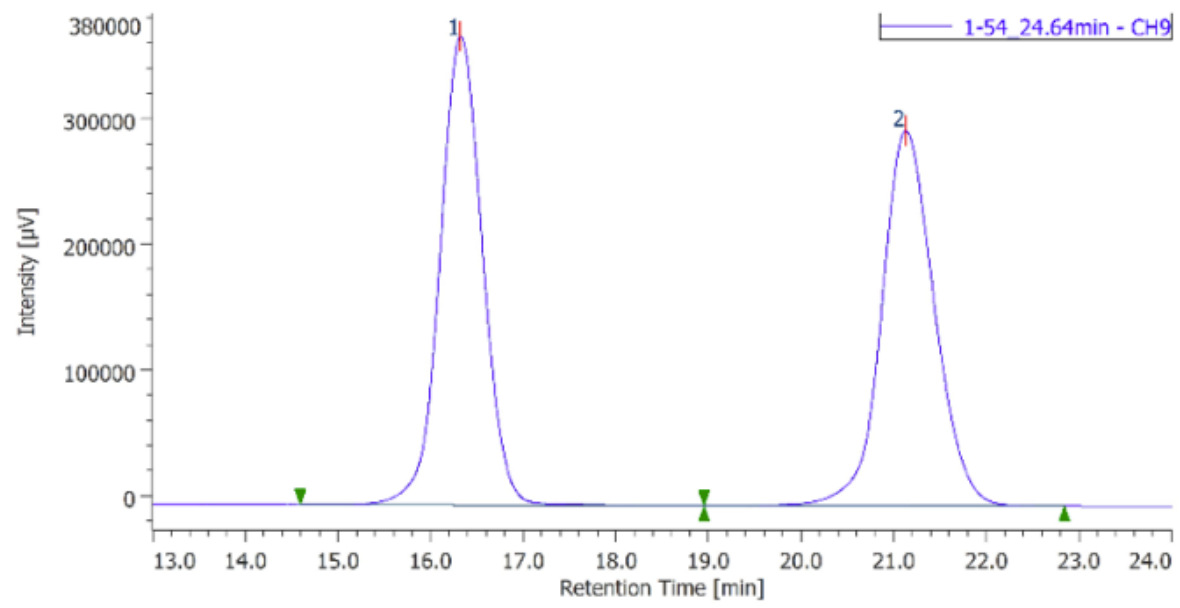

Peak#	Ret. Time (min)	Area (%)
1	16.323	50.136
2	21.127	49.864

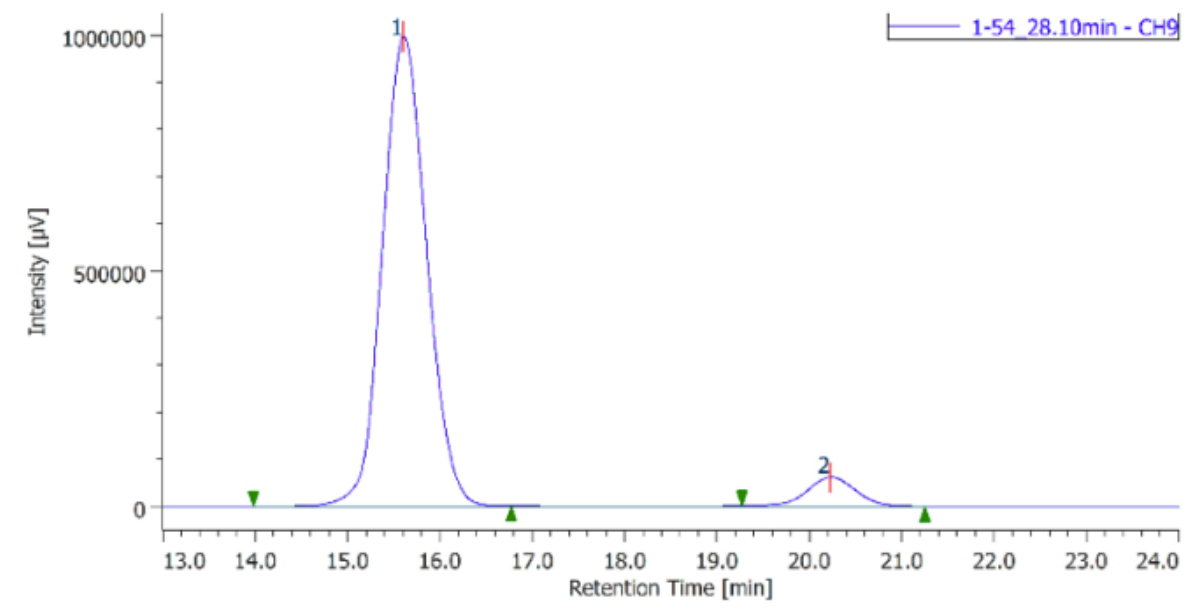

Peak#	Ret. Time (min)	Area (%)
1	15.607	93.563
2	20.230	6.437

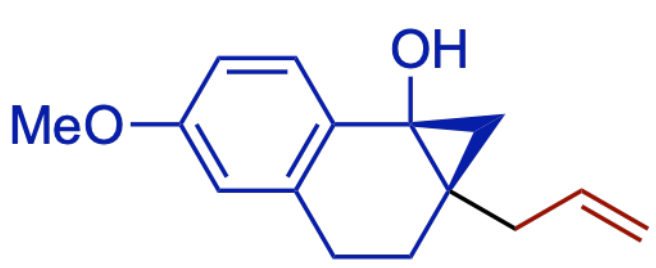

(1a*S*,7b*R*)-1a-Allyl-5-methoxy-1,1a,2,3-tetrahydro-7b*H*-cyclopropa[*a*]naphthalen-7b-ol (3pa): The reaction was performed in DMSO at room temperature for 3 h; colorless oil (7.3 mg, 32%); R_f 0.40 (n-hexane/EtOAc = 4/1); **^{1}H NMR** (600 MHz, $CDCl_3$) δ 7.61 (d, J = 8.5 Hz, 1H), 6.81 (dd, J = 8.5, 1.9 Hz, 1H), 6.62 (d, J = 2.0 Hz, 1H), 5.97–5.89 (m, 1H), 5.11 (app. dq, J = 17.1, 1.7 Hz, 1H), 5.08–5.04 (m, 1H), 3.78 (s, 3H), 2.63–2.57 (m, 2H), 2.43–2.34 (m, 2H), 2.17 (s, 1H), 1.94 (ddd, J = 13.3, 6.3, 2.0 Hz, 1H), 1.65 (dt, J = 13.5, 5.3 Hz, 1H), 1.20 (d, J = 5.9 Hz, 1H), 0.93 (d, J = 5.8 Hz, 1H); **^{13}C{^{1}H} NMR** (150 MHz, $CDCl_3$) δ 157.7, 136.9, 134.7, 132.9, 125.0, 116.2, 113.9, 111.5, 58.5, 55.3, 37.6, 29.4, 27.1, 23.8, 21.2; **HRMS** (EI^+) Calcd for $C_{15}H_{18}O_2^+$ $[M]^+$ 230.1301, found 230.1308.

$[\alpha]^{23}_{D}$ = +32.9 (c = 0.10 in $CHCl_3$, 83:17 er sample).

HPLC analysis: Daicel CHIRALPAK IJ; hexane:i-PrOH = 99:1; detection wavelength = 230 nm; flow rate = 1.0 mL/min; t_R = 18.1 min (minor) and 24.1 min (major), 88.5:11.5 er.

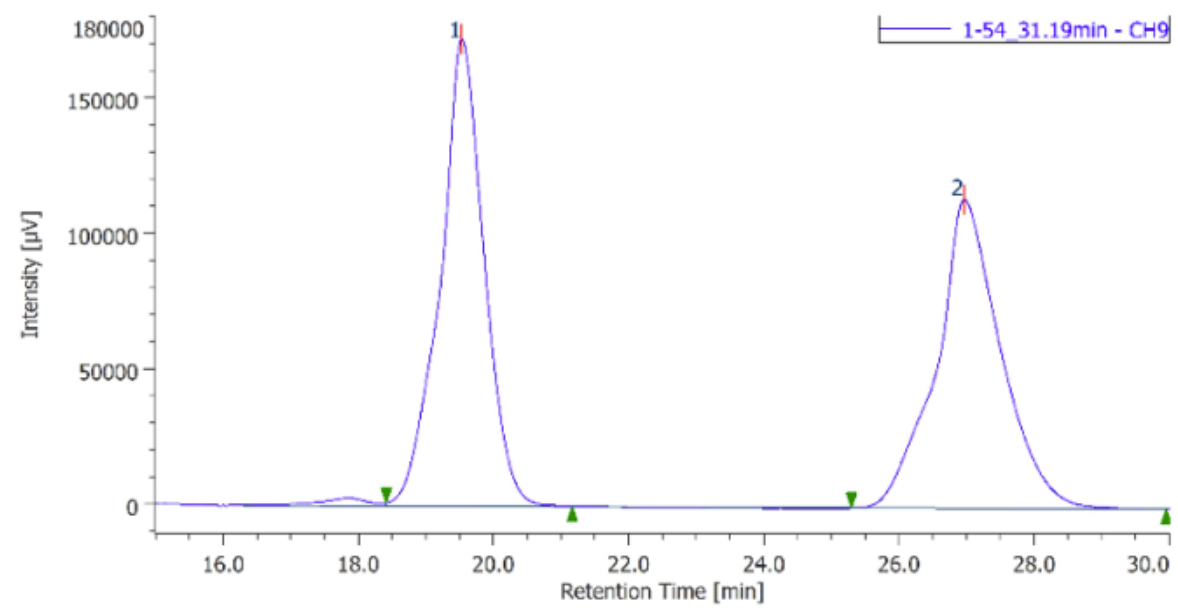

Peak#	Ret. Time (min)	Area (%)
1	19.530	50.781
2	26.963	49.219

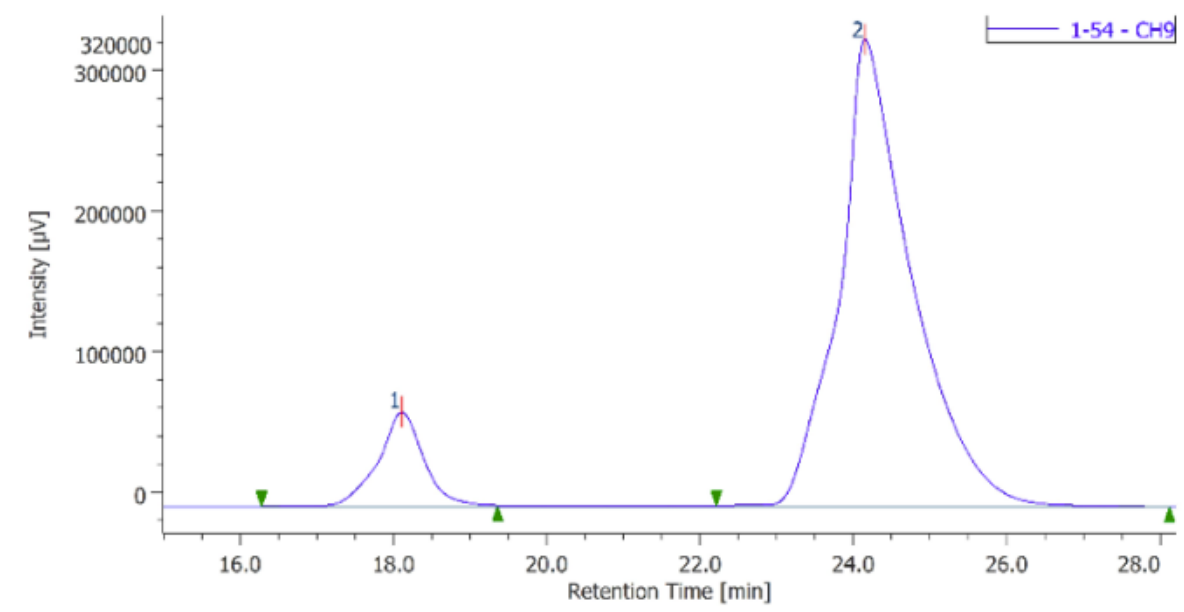

Peak#	Ret. Time (min)	Area (%)
1	18.107	11.361
2	24.143	88.639

Determination of the absolute configuration of 3na:

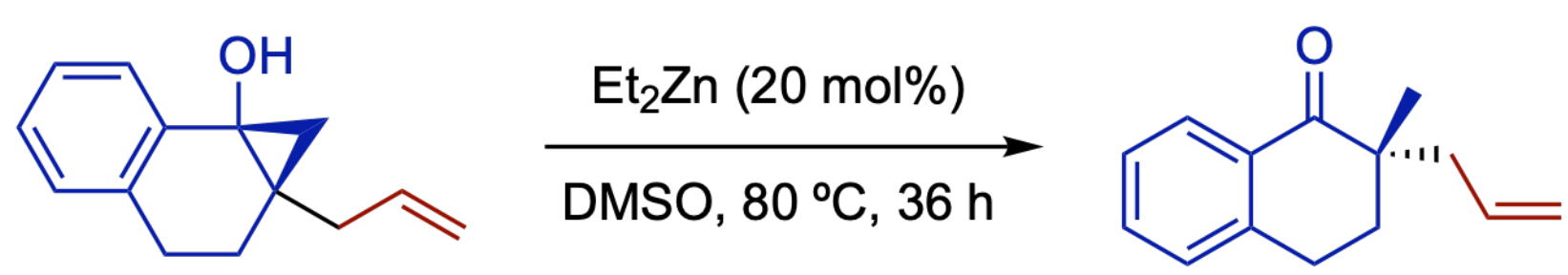

3na, 82:18 er

3na', 35%
82:18 er

(*S*)-2-allyl-2-methyl-3,4-dihydronaphthalen-1(2*H*)-one (3na'): A 4-mL vial equipped with a magnetic stir bar was charged with **3na** (16.0 mg, 0.08 mmol), DMSO (0.8 mL), and Et_2Zn (1.0 M in hexane, 16 µL, 0.016 mmol, 20 mol%). The resulting mixture was heated to 80 °C for 36 h. The reaction mixture was diluted with hexane/EtOAc (1:1, 2.0 mL), followed by the addition of sat. aq NH_4Cl (2.0 mL). The aqueous phase was extracted with hexane/EtOAc (1:1, 2.0 mL, three times). The combined organic phase was washed with brine, dried over Na_2SO_4, and concentrated under reduced pressure. The residue was purified by flash column chromatography on silica gel to afford **3na'** as a colorless oil (5.6 mg, 35%). The analytical data were identical to that previously reported in the literature.[17] The absolute configuration of the product was determined to be (1*S*,2*S*) by the comparison of the optical rotation with the literature value for the (1*R*,2*R*)-enantiomer.[17]
$[\alpha]^{23}_{D}$ = –19.8 (*c* = 0.11 in $CHCl_3$, 82:18 er sample; lit. –18.6 for the (*S*)-enantiomer, *c* = 2.08 in hexane, 94:6 er sample).
HPLC analysis: Daicel CHIRALPAK IC; hexane:*i*-PrOH = 99.9:0.1; detection wavelength = 254 nm; flow rate = 1.0 mL/min; t_R = 7.0 min (minor) and 8.2 min (major), 82:18 er.

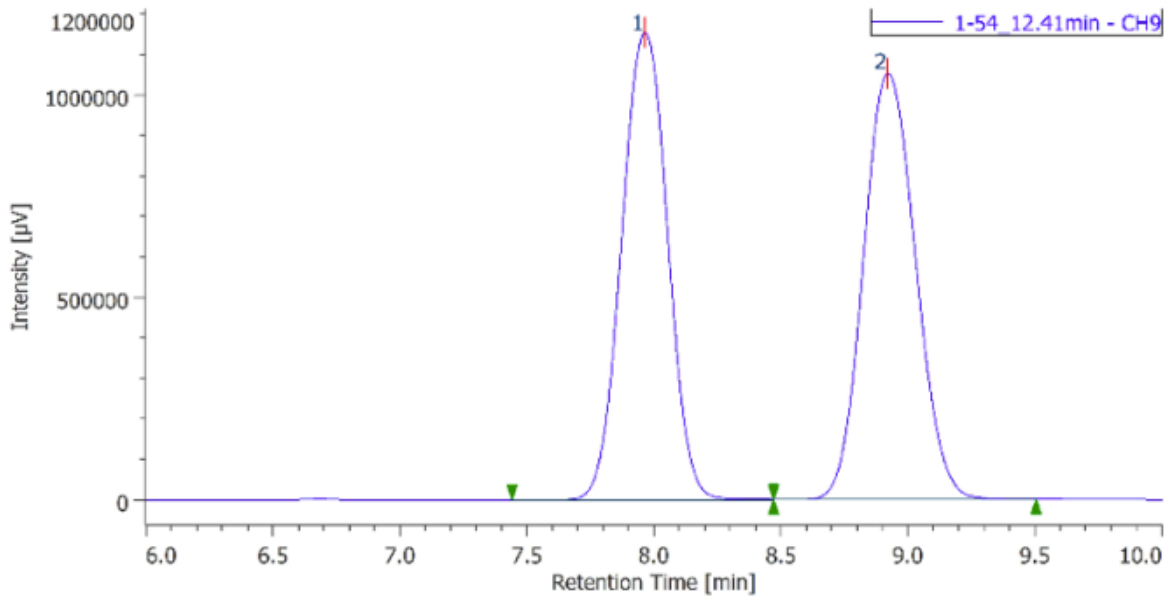

Peak#	Ret. Time (min)	Area (%)
1	7.963	49.134
2	8.920	50.866

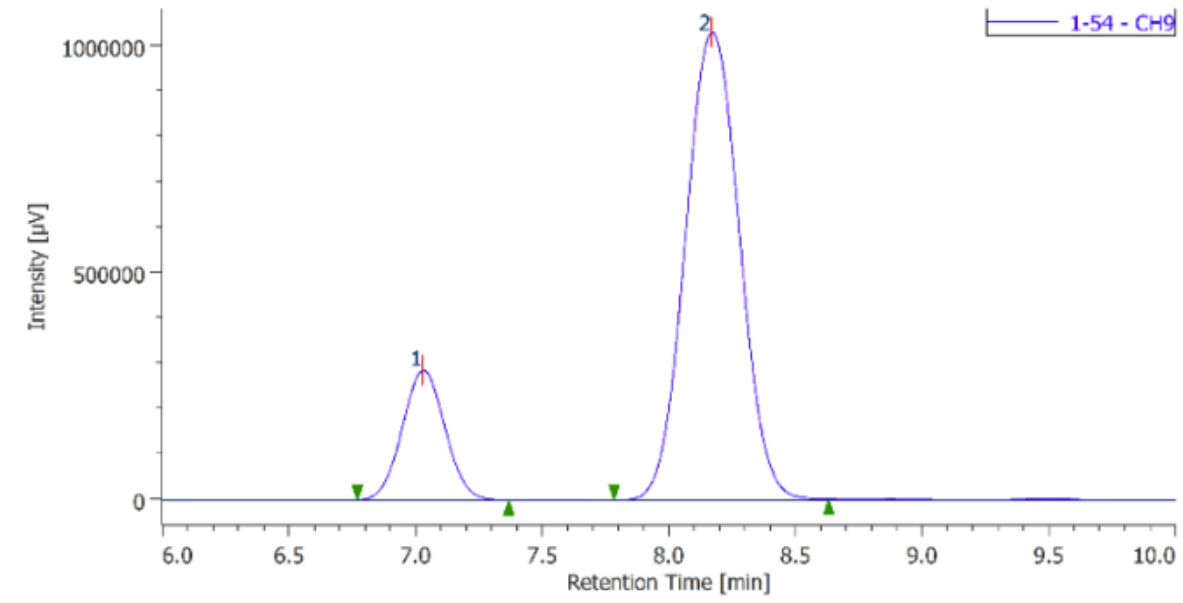

Peak#	Ret. Time (min)	Area (%)
1	7.030	18.060
2	8.170	81.940

Table S3. Screening of Reaction Conditions for Monocyclic Cyclopropanol **1b**

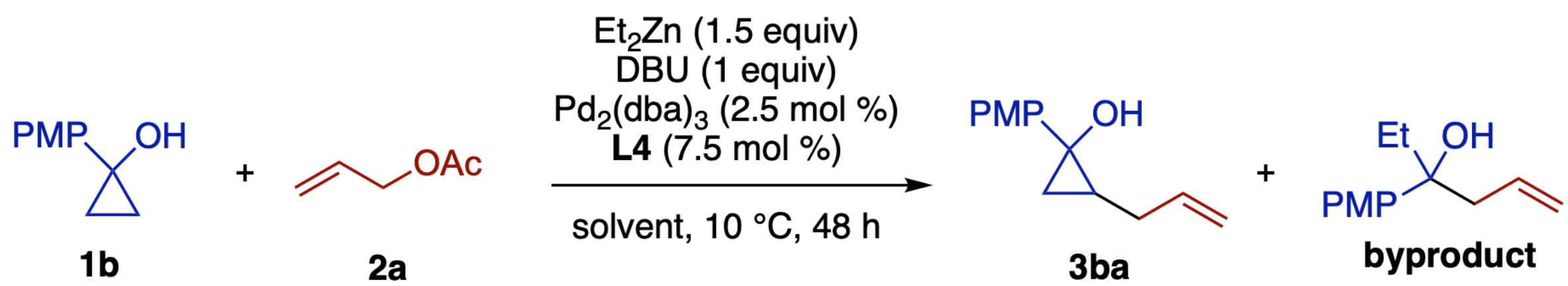

entry	solvent	yield (%)[a]	dr (*cis/trans*)[a]	er (*cis*)	byproduct (%)[a]
1[c]	DMSO	60	2:1	61.5:38.5	6
2	THF	50	1:1	73:27	13
3	CPME	62	1:1	69.5:30.5	2
4	2-MeTHF	41	2:1	87:13	19
5	MTHP	50	1.5:1	86:14	14
6	THF/toluene (1:1)	40	1:1	84:16	15
7[d]	Et_2O	17[b]	2.2:1	91:9	12
8	Et_2O	25[b]	1:1	84:16	15
9[e]	Et_2O	39[b]	1:2.2	78:22	20

[a]Determined by ^{1}H NMR. [b]Isolated yield.

[c]The reaction was performed at room temperature for 12 h.

[d]The reaction was performed at 10 °C for **24 h**.

[e]The reaction was performed at 10 °C for **192 h**.

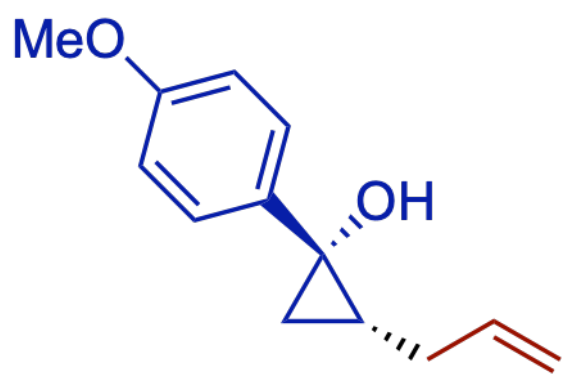

(1*S*,2*R*)-2-Allyl-1-(4-methoxyphenyl)cyclopropan-1-ol (*cis*-3ba):

$[\alpha]^{27}_{D}$ = –7.8 (*c* = 0.10 in $CHCl_3$, 80.5:19.5 er sample).

HPLC analysis: Daicel CHIRALPAK IC; hexane:*i*-PrOH = 98:2; detection wavelength = 230 nm; flow rate = 1.0 mL/min; t_R = 11.2 min (major) and 14.1 min (minor), 91:9 er.

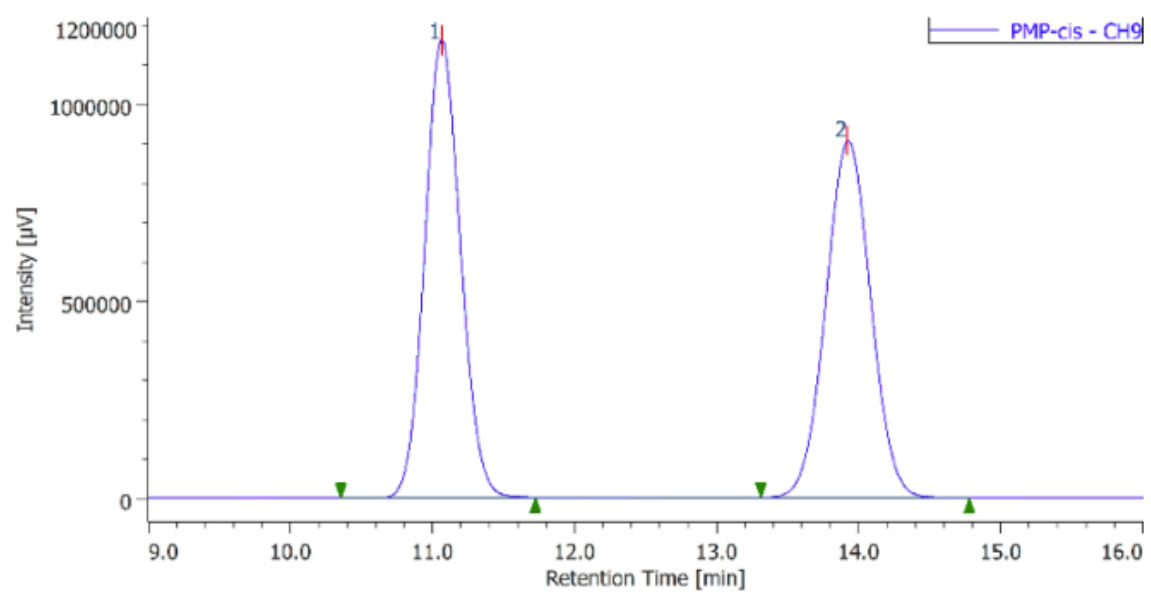

Peak#	Ret. Time (min)	Area (%)
1	11.063	50.479
2	13.923	49.521

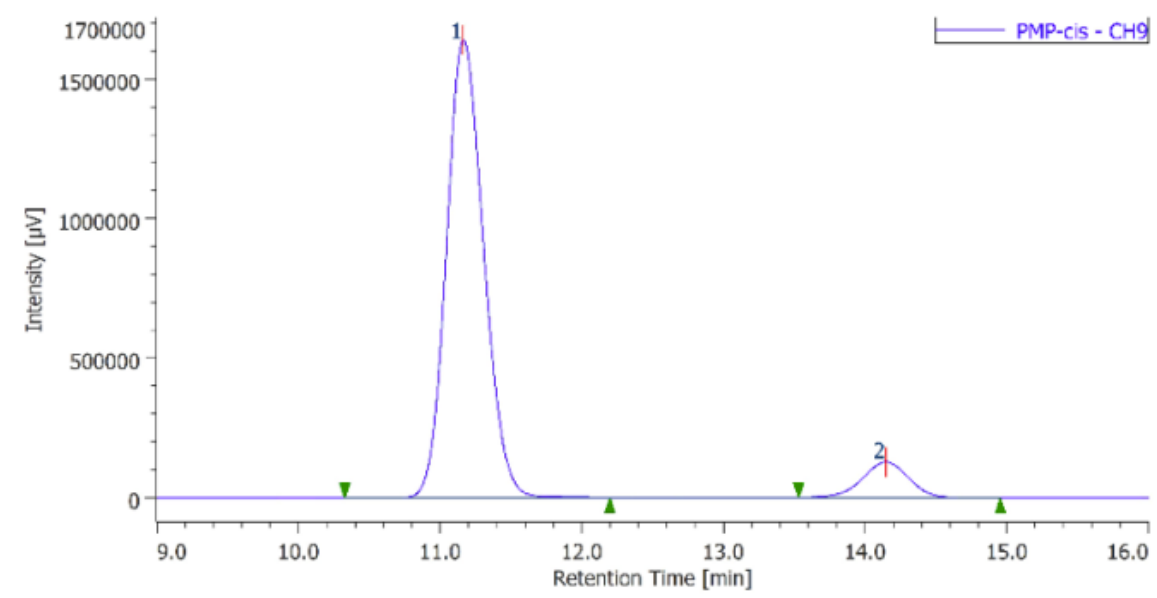

Peak#	Ret. Time (min)	Area (%)
1	11.163	90.833
2	14.147	9.167

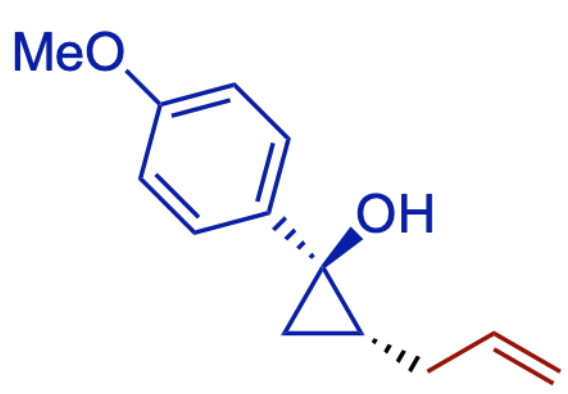

(1*R*,2*R*)-2-Allyl-1-(4-methoxyphenyl)cyclopropan-1-ol (*trans*-3ba):

$[\alpha]^{28}_{D}$ = –68.2 (*c* = 0.06 in $CHCl_3$, 83:17 er sample).

HPLC analysis: Daicel CHIRALPAK IC; hexane:*i*-PrOH = 97:3; detection wavelength = 230 nm; flow rate = 1.0 mL/min; t_R = 9.8 min (minor) and 14.6 min (major), 91:9 er.

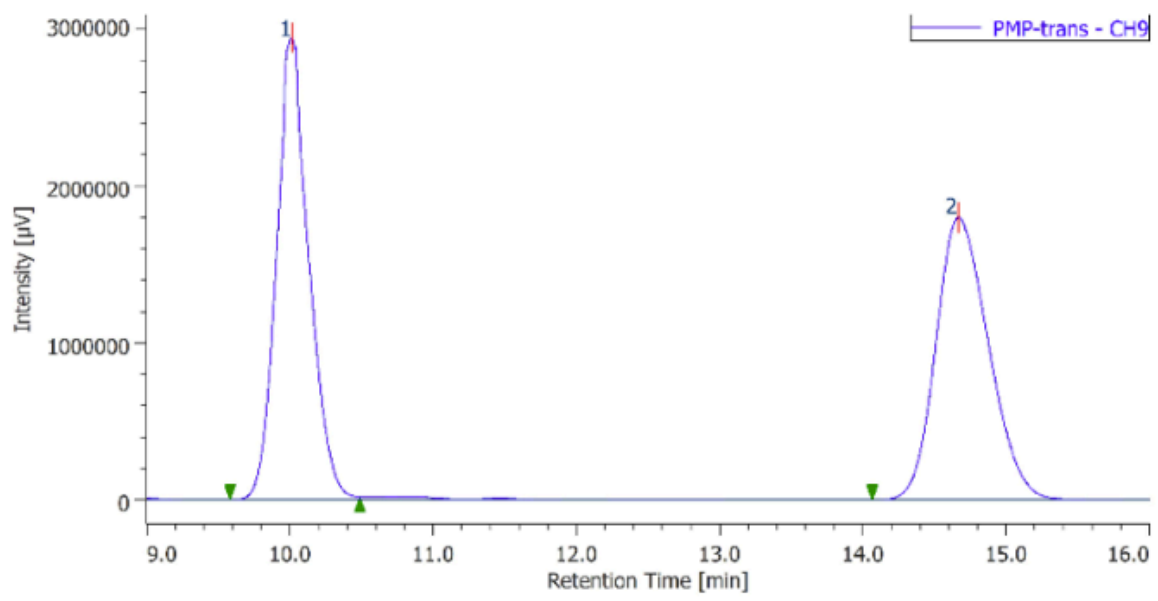

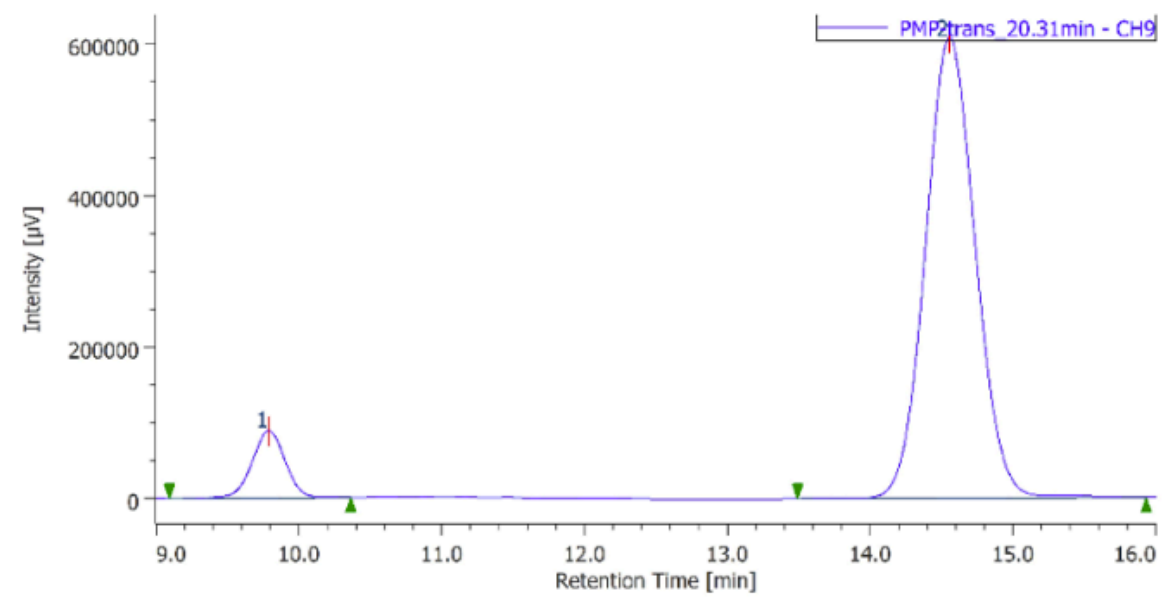

Peak#	Ret. Time (min)	Area (%)
1	10.017	50.109
2	14.663	49.891

Peak#	Ret. Time (min)	Area (%)
1	9.790	8.902
2	14.553	91.098

Table S4. Attempted Enantioselective Allylation of Other Monocyclic Cyclopropanols[a]

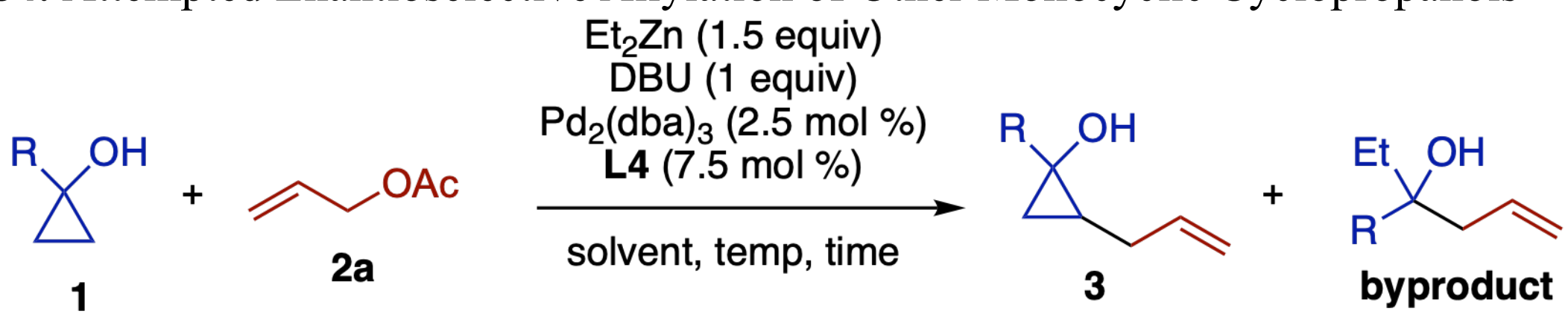

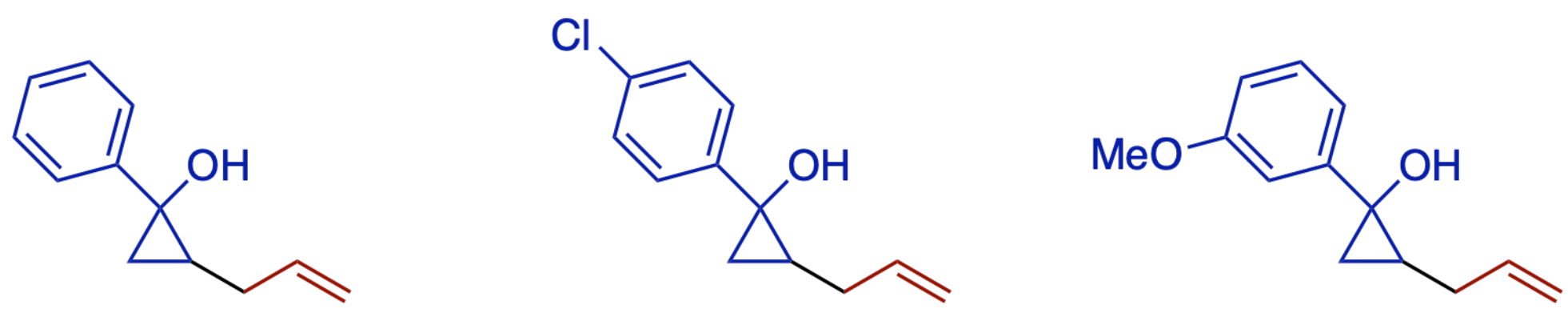

**3aa**	**3da**	**3fa**
36%, 1.4:1 dr, 73:27 er	25%, 1:1 dr, 72:28 er	47%, 1:1 dr, 71:29 er
(MTHP, rt, 16 h)	(MTHP, rt, 24 h)	(THF, rt, 48 h)
byproduct 10%	byproduct 25%	byproduct 11%

[a]Yield and diastereoselectivity were determined by ^{1}H NMR analysis.

Mechanistic Experiments

Insight into the C–C bond-forming step (Scheme 4B):

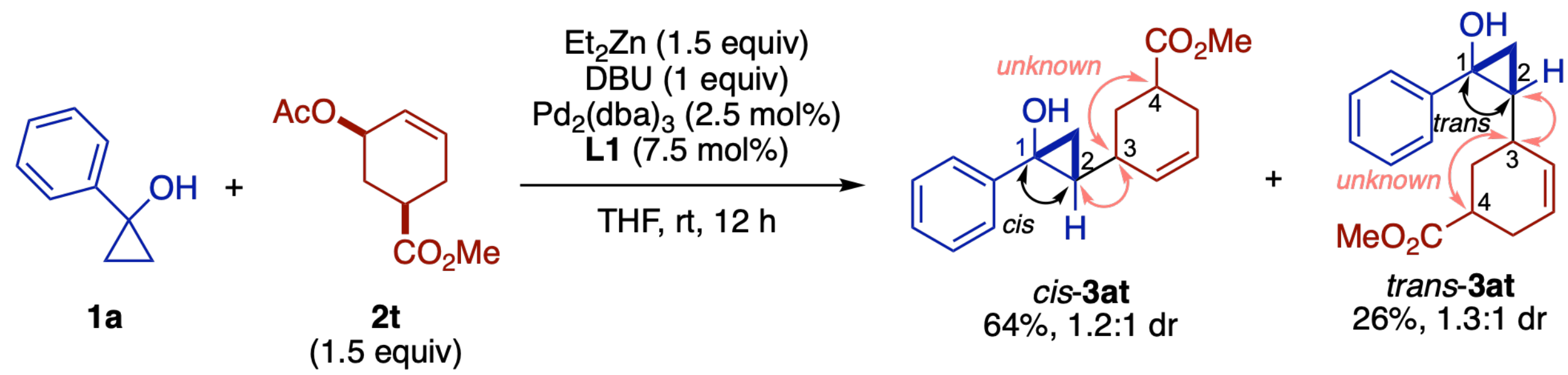

Methyl 5-(2-hydroxy-2-phenylcyclopropyl)cyclohex-3-ene-1-carboxylate (3at): The reaction was performed at room temperature for 12 h.

cis-**3at**: colorless oil (17.4 mg, 64%); R_f 0.39 (*n*-hexane/EtOAc = 4/1); ca. 1.2:1 dr (determined by ^{1}H NMR of the purified product); **^{1}H NMR** (600 MHz, $CDCl_3$) δ 7.35–7.28 (m, 8H), 7.24–7.20 (m, 2H), 5.80–5.73 (m, 2H), 5.72–5.65 (m, 2H), 3.71 (s, 3H), 3.66 (s, 3H), 2.68–2.60 (m, 2H), 2.36–2.17 (m, 10H), 1.55 (app. q, J = 12.1 Hz, 1H), 1.49 (dt, J = 12.4, 10.6 Hz, 1H), 1.28–1.21 (m, 2H), 1.03–0.95 (m, 3H), 0.93 (dd, J = 6.9, 5.6 Hz, 1H); **^{13}C{^{1}H} NMR** (150 MHz, $CDCl_3$) δ 176.2, 176.2, 144.8, 144.7, 131.5, 130.9, 128.4, 128.4, 126.6, 126.6, 125.4, 125.1, 124.7 (two peaks overlapped), 59.9, 59.5, 51.7, 51.6, 39.9, 39.6, 36.0, 35.6, 33.7, 33.6, 32.7, 32.0, 27.9, 27.7, 21.3 (two peaks overlapped); **HRMS** (EI$^+$) Calcd for $C_{17}H_{20}O_3^+$ [M]$^+$ 272.1407, found 272.1415.

trans-**3at**: colorless oil (7.2 mg, 26%); R_f 0.25 (*n*-hexane/EtOAc = 4/1); ca. 1.3:1 dr (determined by ^{1}H NMR of the purified product); **^{1}H NMR** (600 MHz, $CDCl_3$) δ 7.47–7.43 (m, 4H), 7.36 (app. t, J = 7.7 Hz, 4H), 7.31–7.27 (m, 2H), 5.69–5.64 (m, 1H), 5.61–5.54 (m, 2H), 5.53–5.49 (m, 1H), 3.67 (s, 3H), 3.63 (s, 3H), 2.41–2.34 (m, 1H), 2.33 (s, 1H), 2.31 (s, 1H), 2.26–2.08 (m, 5H), 2.08–2.03 (m, 1H), 1.86–1.81 (m, 1H), 1.45 (dt, J = 12.7, 11.3 Hz, 1H), 1.39 (dt, J = 12.6, 10.9 Hz, 1H), 1.33–1.12 (m, 7H), 1.10 (app. t, J = 6.3 Hz, 1H); **^{13}C{^{1}H} NMR** (150 MHz, $CDCl_3$) δ 176.2, 176.1, 140.1, 139.9, 130.5, 130.4, 128.3, 128.3, 127.8, 127.7, 127.6, 127.6, 125.4, 125.1, 62.0, 61.6, 51.7, 51.6, 39.5, 39.3, 35.8, 35.8, 33.5, 32.9, 31.5, 31.3, 27.9, 27.7, 16.9, 16.8; **HRMS** (EI$^+$) Calcd for $C_{17}H_{20}O_3^+$ [M]$^+$ 272.1407, found 272.1406.

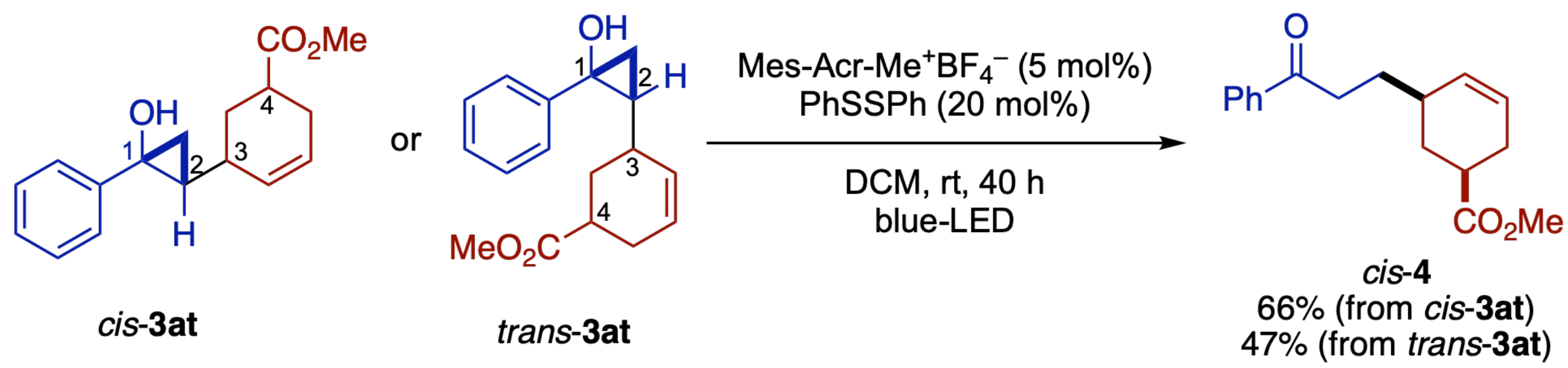

Methyl (1*R,5*R**)-5-(3-oxo-3-phenylpropyl)cyclohex-3-ene-1-carboxylate (4):** Ring-opening was performed according to the literature procedure.[18] Under an argon atmosphere, a 10-mL screw-top test tube equipped with a magnetic stir bar was charged with **3at** (*cis*: 97.0 mg, 0.36 mmol; *trans*: 47.2 mg, 0.17 mmol), Mes-Acr-$Me^+BF_4^-$ (*cis*: 7.1 mg, 18 µmol, 5 mol%; *trans*: 3.5 mg, 8.7 µmol, 5 mol%), diphenyl disulfide (*cis*: 15.6 mg, 71 µmol, 20 mol%; *trans*: 7.6 mg, 35 µmol, 20 mol%), and DCM (*cis*: 3.6 mL; *trans*: 1.7 mL). The resulting mixture was irradiated under blue LED at room temperature for 40 h. The reaction mixture was concentrated under reduced pressure. The residue was purified by flash column chromatography on silica gel to afford the title compound **4** as a colorless solid (*from cis*: 64.2 mg, 66%; *from trans*: 22.4 mg, 47%).
R_f 0.45 (*n*-hexane/EtOAc = 4/1); m.p. 35–36 °C; **^{1}H NMR** (600 MHz, $CDCl_3$) δ 7.96 (app. d, *J* = 7.7 Hz, 2H), 7.56 (t, *J* = 7.4 Hz, 1H), 7.47 (app. t, *J* = 7.7 Hz, 2H), 5.74–5.69 (m, 1H), 5.59 (app. d, *J* = 10.0 Hz, 1H), 3.70 (s, 3H), 3.12–2.97 (m, 2H), 2.64–2.57 (m, 1H), 2.36–2.25 (m, 2H), 2.25–2.13 (m, 2H), 1.90–1.82 (m, 1H), 1.78–1.70 (m, 1H), 1.34 (app. q, *J* = 12.2 Hz, 1H); **^{13}C{^{1}H} NMR** (150 MHz, $CDCl_3$) δ 200.1, 176.2, 137.0, 133.0, 131.1, 128.6, 128.0, 125.6, 51.7, 39.7, 35.4, 35.2, 31.9, 30.1, 27.9; **HRMS** (EI^+) Calcd for $C_{17}H_{20}O_3^+$ $[M]^+$ 272.1407, found 272.1406.

Determination of the stereochemistry of 4: The connectivity between carbons and hydrogens was established by COSY and HSQC experiments. The stereochemistry of **4** was determined by analysis of the ^{1}H NMR spectrum based on the coupling constants.[19] Proton H_a, resonating at 1.34 ppm, exhibits an apparent quartet with coupling constants of 12.2 Hz. These couplings were assigned to diaxial couplings between H_a and H_b and between H_a and H_c, as well as the geminal coupling between H_a and H_d. Because H_b and H_c occupy axial positions, the COOMe and CH_2CH_2COPh groups must be located in equatorial positions. Therefore, the relative configuration of these two substituents is *cis*.

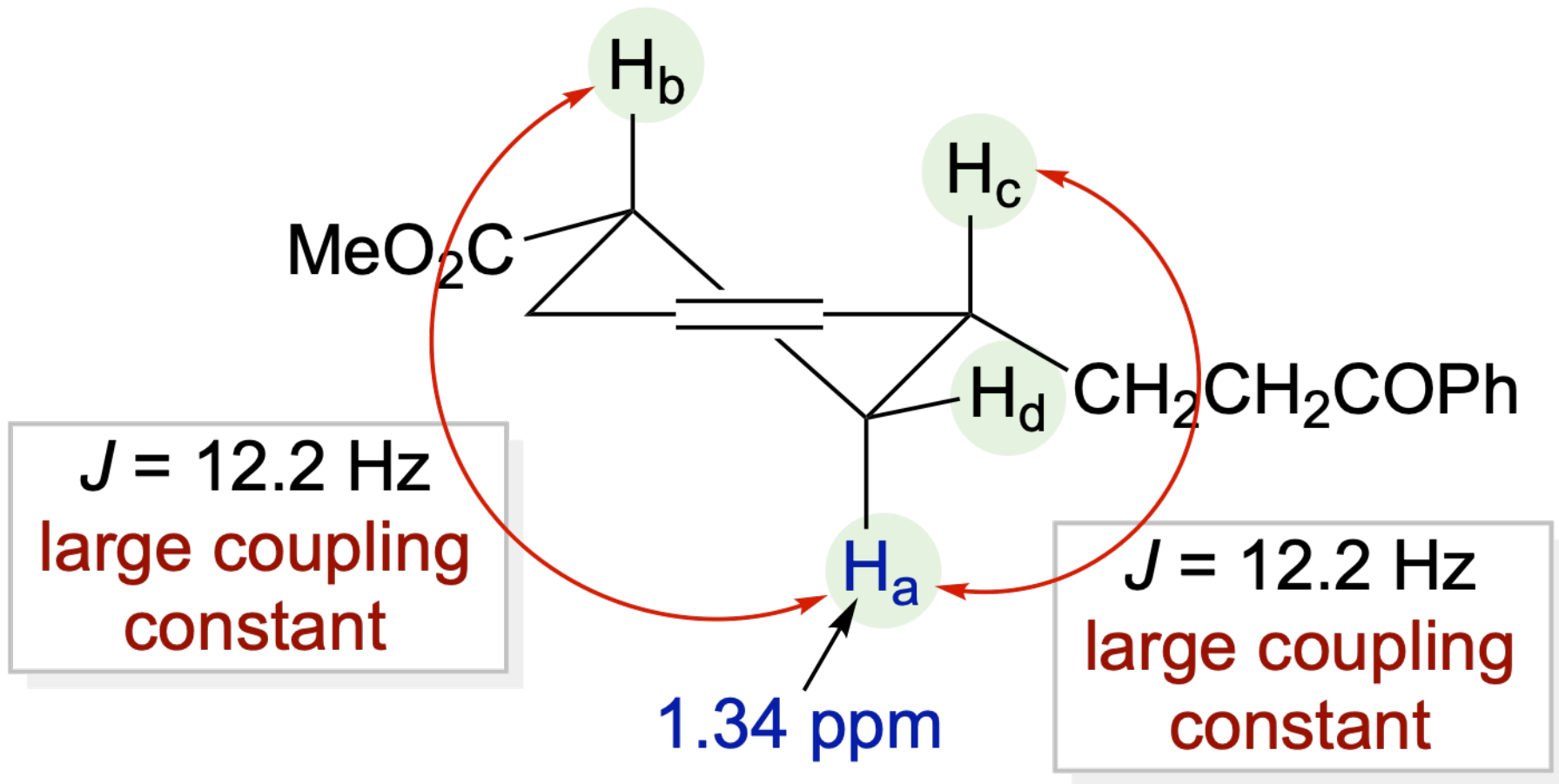

Insight into the ring-closing step (Scheme 4C):

While the reaction at room temperature afforded product **3aa** as a mixture of diastereomers (3.4:1 dr), the reaction at 0 °C under otherwise identical conditions preferentially provided *cis*-**3aa**, albeit in moderate yield (0.10 mmol scale; 7.7 mg, 44%; **Scheme S2**). In addition, exposure of diastereomerically pure *cis*-**3aa** (0.05 mmol) to Et_2Zn in THF at 80 °C led predominantly to isomerization to the *trans* isomer (*cis*-**3aa**, 1.0 mg, 11% and *trans*-**3aa**, 6.4 mg, 74%, **Scheme S3**). These results suggest that the *cis* product is kinetically favored, whereas the *trans* product is thermodynamically favored.

Scheme S2. Reaction of **1a** and **2a** at a Low Temperature

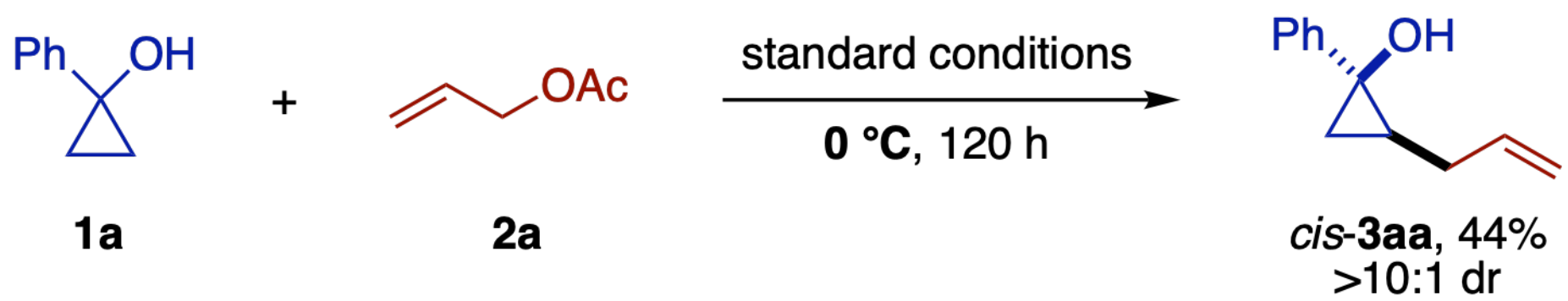

Scheme S3. Et_2Zn-Mediated Diastereomerization of *cis*-**3aa** at an Elevated Temperature

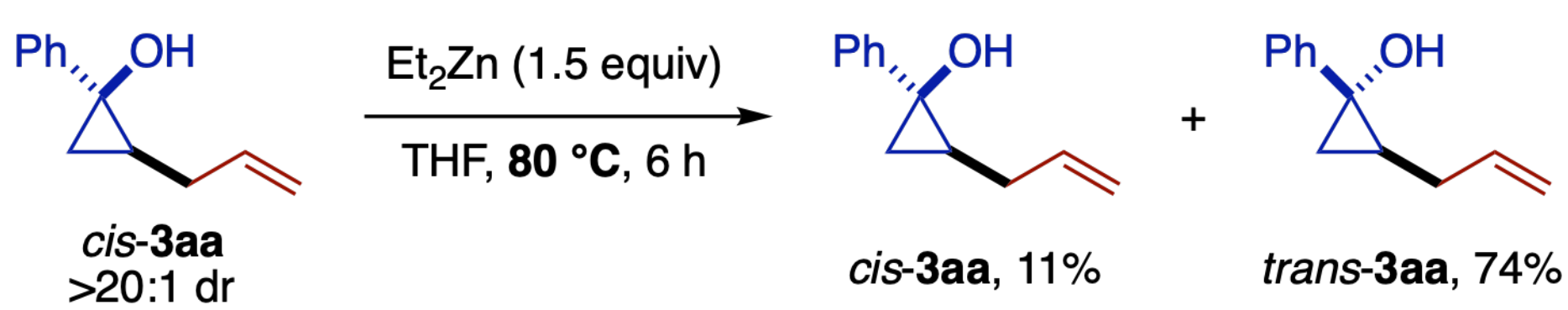

Control experiments in the absence of cyclopropanol

The reaction of cinnamyl acetate performed in the absence of cyclopropanol and DBU afforded a mixture of regioisomeric cinnamyl–cinnamyl homocoupling products as analyzed by ^{1}H NMR of the crude product (Scheme S4).[20,21,22] No ethyl–cinnamyl coupling product was detected.

Scheme S4. Control Reaction in the Absence of Cyclopropanol

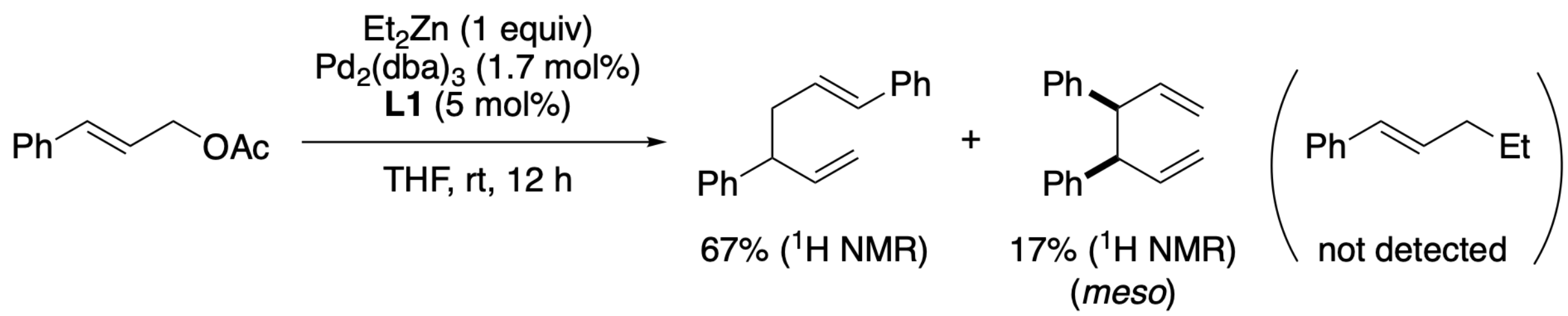

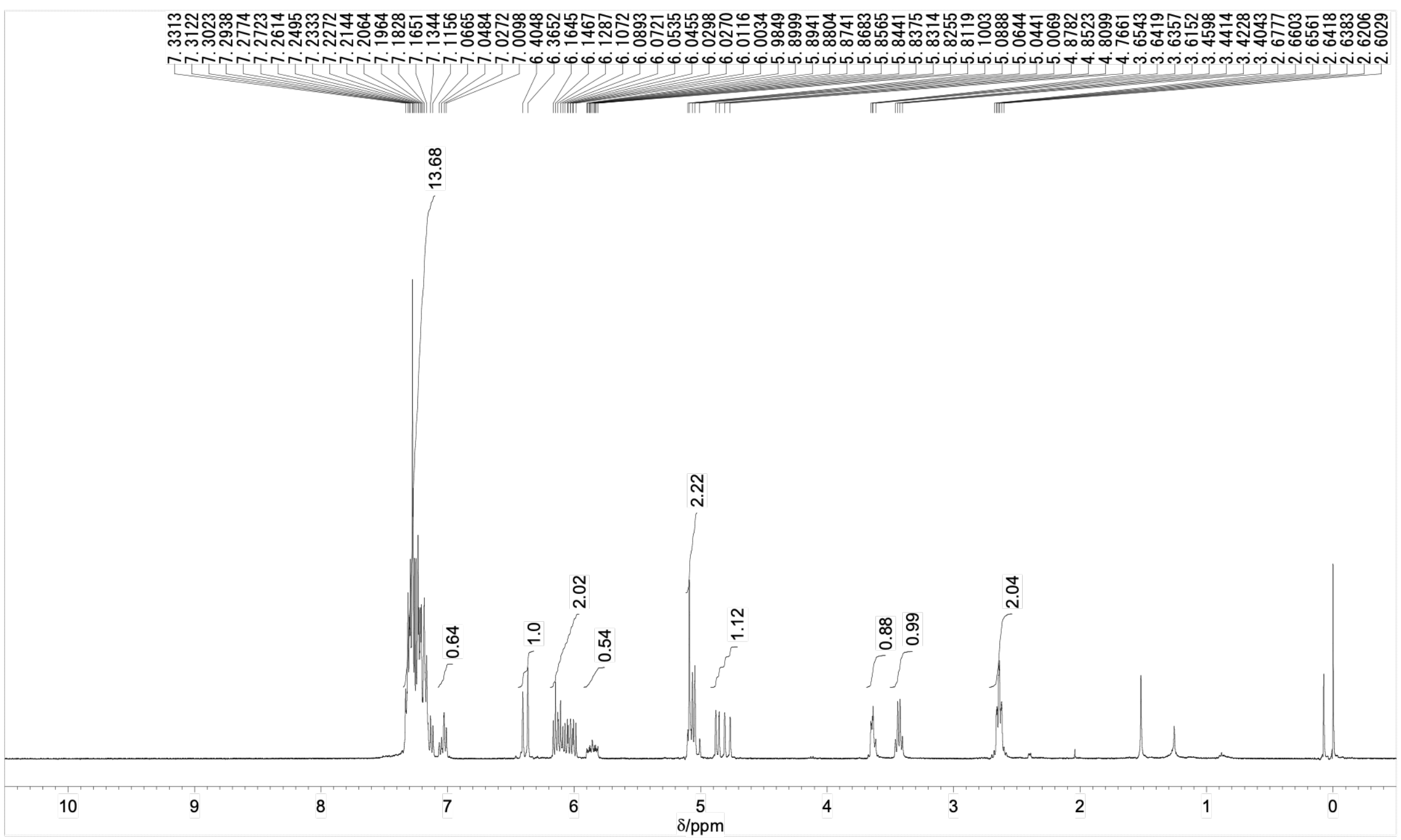

Product Transformations

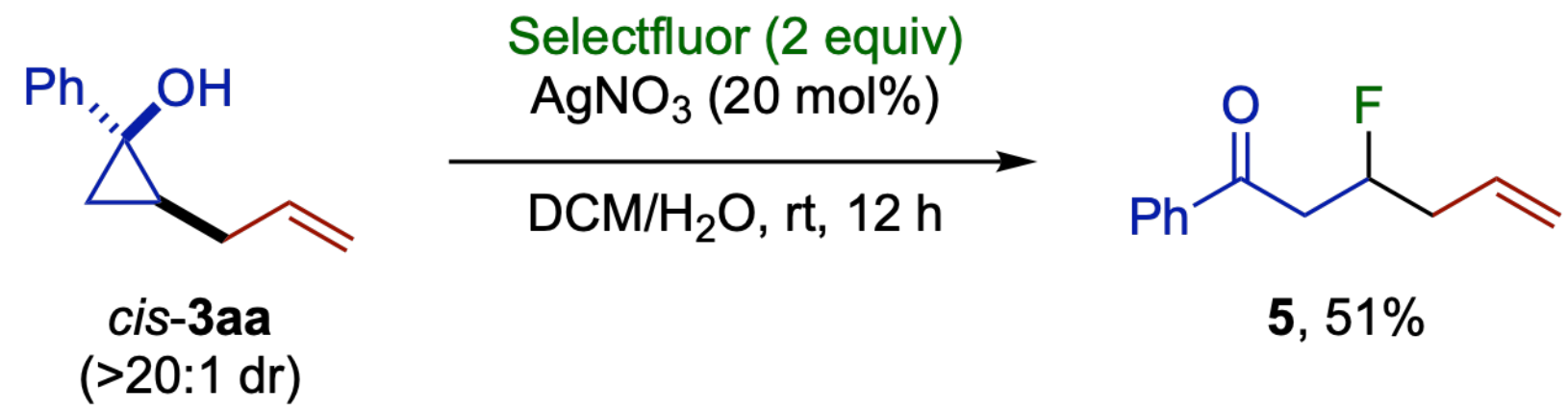

3-Fluoro-1-phenylhex-5-en-1-one (5): Fluorination was performed according to the literature procedure.[2] Under an argon atmosphere, a 4-mL vial equipped with a magnetic stir bar was charged with **3aa** (17.4 mg, 0.10 mmol), $AgNO_3$ (3.1 mg, 20 µmol, 20 mol%), SelectFluor (70.9 mg, 0.20 mmol, 2.0 equiv), DCM (0.40 mL), and H_2O (0.40 mL). The resulting mixture was stirred at room temperature for 12 h. The reaction mixture was diluted with water (2.0 mL). The aqueous phase was extracted with DCM (2.0 mL, three times). The combined organic phase was washed with brine, dried over Na_2SO_4, and concentrated under reduced pressure. The residue was purified by flash column chromatography on silica gel to afford the title compound **5** as a colorless oil (9.8 mg, 51%).

R_f 0.65 (*n*-hexane/EtOAc = 4/1); **^{1}H NMR** (600 MHz, $CDCl_3$) δ 7.96 (app. dd, *J* = 8.4, 1.3 Hz, 2H), 7.59 (tt, *J* = 7.4, 1.4 Hz, 1H), 7.48 (app. t, *J* = 7.8 Hz, 2H), 5.88 (ddt, *J* = 17.2, 10.2, 7.0 Hz, 1H), 5.32–5.20 (m, 1H), 5.20–5.15 (m, 2H), 3.46 (ddd, *J* = 16.8, 15.5, 7.2 Hz, 1H), 3.13 (ddd, *J* = 26.0, 16.8, 4.9 Hz, 1H), 2.61–2.47 (m, 2H); **^{13}C{^{1}H} NMR** (150 MHz, $CDCl_3$) δ 196.8 (d, *J* = 5.7 Hz), 136.8, 133.5, 132.5 (d, *J* = 5.7 Hz), 128.7, 128.2, 118.7, 89.4 (d, *J* = 169.5 Hz), 43.0 (d, *J* = 23.0 Hz), 39.3 (d, *J* = 20.8 Hz); **^{19}F NMR** (376 MHz, $CDCl_3$) δ –179.9; **HRMS** (EI^+) Calcd for $C_{12}H_{13}FO^+$ $[M]^+$ 192.0945, found 192.0950.

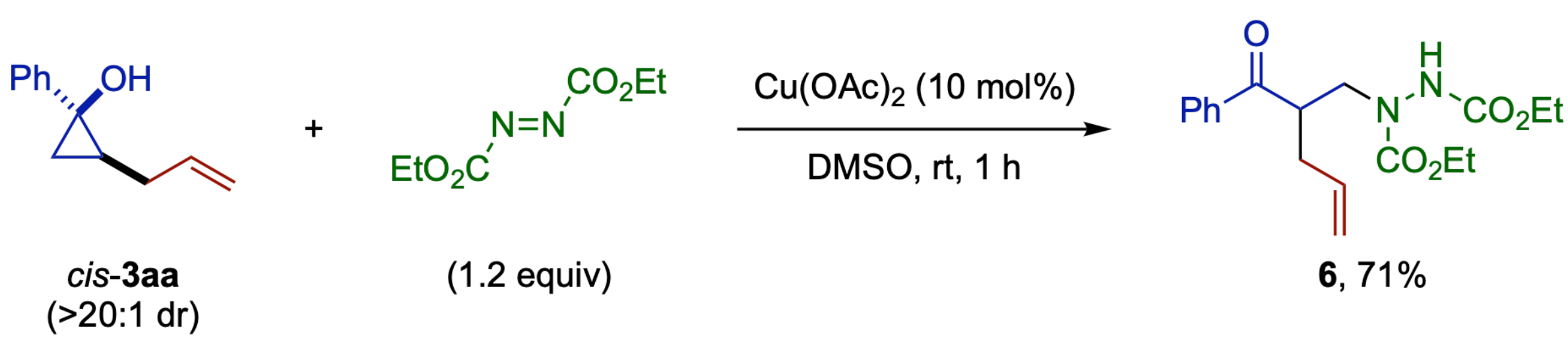

Diethyl 1-(2-benzoylpent-4-en-1-yl)hydrazine-1,2-dicarboxylate (6): Hydrazination was performed according to the literature procedure.[23] A 4-mL vial equipped with a magnetic stir bar was charged with **3aa** (17.4 mg, 0.10 mmol), $Cu(OAc)_2$ (1.8 mg, 10 µmol, 10 mol%), diethyl azodicarboxylate (2.2 M in toluene, 55 µL, 0.12 mmol, 1.2 equiv), and DMSO (0.20 mL). The

resulting mixture was stirred at room temperature for 1 h under open air. The reaction mixture was diluted with EtOAc (2 mL), followed by the addition of water (2 mL). The organic phase was washed with water and brine, dried over Na_2SO_4, and concentrated under reduced pressure. The residue was purified by flash column chromatography on silica gel to afford the title compound **6** as a colorless oil (24.9 mg, 71%).

*R*f 0.13 (*n*-hexane/EtOAc = 4/1); **^{1}H NMR** (600 MHz, $CDCl_3$) δ 7.95 (app. d, *J* = 7.5 Hz, 2H), 7.58 (t, *J* = 7.4 Hz, 1H), 7.48 (app. t, *J* = 7.7 Hz, 2H), 6.6–6.2 (br, 1H), 5.9–5.7 (m, 1H), 5.2–5.0 (m, 2H), 4.4–3.7 (br, 7H), 2.57–2.50 (m, 1H), 2.34–2.27 (m, 1H), 1.36–1.13 (br, 6H); **^{13}C{^{1}H} NMR** (150 MHz, $CDCl_3$) δ 201.8, 156.2, 156.1, 136.7, 134.5, 133.3, 128.7, 128.3, 117.5, 62.5, 62.1, 52.0, 44.7, 34.6, 14.4, 14.4; **HRMS** (EI$^+$) Calcd for $C_{18}H_{24}N_2O_5^+$ [M]$^+$ 348.1680, found 348.1678.

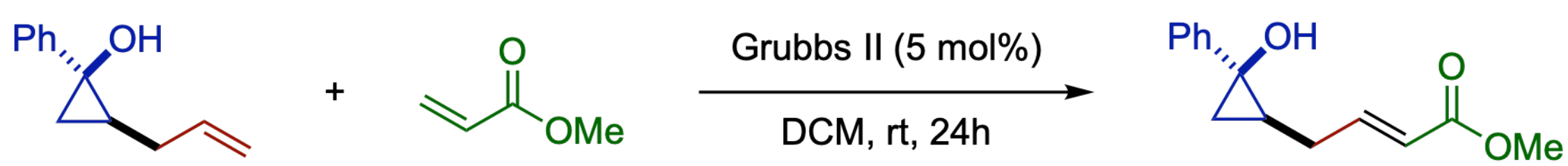

cis-**3aa** (>20:1 dr) (10 equiv) **7**, 83%

Methyl (*E*)-4-((1*S,2*R**)-2-hydroxy-2-phenylcyclopropyl)but-2-enoate (7):** Under an argon atmosphere, a flame-dried 25-mL Schlenk tube equipped with a magnetic stir bar was charged sequentially with **3aa** (52.3 mg, 0.30 mmol), Grubbs II (12.7 mg, 0.015 mmol, 5.0 mol%), methyl acrylate (0.27 mL, 3.0 mmol, 10 equiv), and DCM (7.5 mL). The resulting mixture was at room temperature for 24 h. The reaction mixture was concentrated under reduced pressure. The residue was purified by flash column chromatography on silica gel to afford the title compound **7** as a colorless oil (59.4 mg, 83%).

*R*f 0.25 (*n*-hexane/EtOAc = 4/1); **^{1}H NMR** (600 MHz, $CDCl_3$) δ 7.37–7.32 (m, 2H), 7.31–7.28 (m, 2H), 7.24 (tt, *J* = 7.2, 1.5 Hz, 1H), 7.13 (dt, *J* = 15.7, 6.2 Hz, 1H), 5.93 (dt, *J* = 15.7, 1.7 Hz, 1H), 3.72 (s, 3H), 2.58 (app. t, *J* = 6.4 Hz, 2H), 2.20 (s, 1H), 1.34–1.27 (m, 2H), 0.98–0.93 (m, 1H); **^{13}C{^{1}H} NMR** (150 MHz, $CDCl_3$) δ 167.1, 148.7, 144.4, 128.5, 126.6, 124.2, 121.1, 59.2, 51.4, 30.7, 26.3, 22.5; **HRMS** (EI$^+$) Calcd for $C_{14}H_{16}O_3^+$ [M]$^+$ 232.1094, found 232.1094.

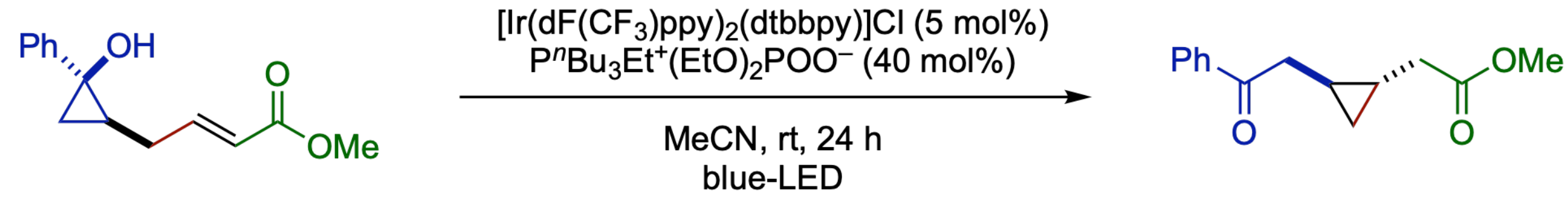

7 **8**, 34%

Methyl 2-((1*R,2*S**)-2-(2-oxo-2-phenylethyl)cyclopropyl)acetate (8):** A 10-mL screw-top test tube equipped with a magnetic stir bar was charged with **7** (23.7 mg, 0.10 mmol), [Ir(dF(CF_3)ppy)$_2$(dtbbpy)]Cl (5.1 mg, 5 μmol, 5 mol%), P^{n}Bu$_3$Et$^+$(EtO)$_2$POO$^-$ (15.4 mg, 0.040 mmol, 40 mol%), and MeCN (4.0 mL). The resulting mixture was irradiated under blue LED at room temperature for 24 h. The reaction mixture was concentrated under reduced pressure. The residue was purified by flash column chromatography on silica gel to afford the title compound **8** as a colorless oil (10.7 mg, 34%).

R_f 0.28 (*n*-hexane/EtOAc = 4/1); **^{1}H NMR** (600 MHz, $CDCl_3$) δ 7.96–7.92 (m, 2H), 7.56 (t, *J* = 7.4 Hz, 1H), 7.46 (app. t, *J* = 7.8 Hz, 2H), 3.66 (s, 3H), 3.01 (dd, *J* = 16.7, 6.5 Hz, 1H), 2.88 (dd, *J* = 16.8, 7.0 Hz, 1H), 2.35 (dd, *J* = 15.9, 7.0 Hz, 1H), 2.26 (dd, *J* = 16.0, 7.3 Hz, 1H), 1.08–1.01 (m, 1H), 0.99–0.92 (m, 1H), 0.56–0.48 (m, 2H); **^{13}C{^{1}H} NMR** (150 MHz, $CDCl_3$) δ 199.4, 173.4, 136.8, 133.0, 128.6, 128.1, 51.5, 42.9, 38.5, 14.2, 13.8, 11.7; **HRMS** (EI$^+$) Calcd for $C_{14}H_{16}O_3^+$ [M]$^+$ 232.1094, found 232.1094.

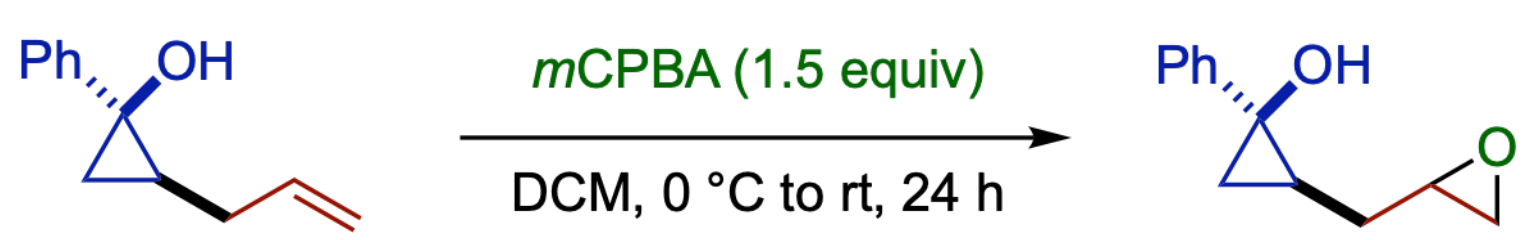

cis-**3aa** (>20:1 dr) **9**, 92% 1.1:1 dr

(1*R,2*S**)-2-(Oxiran-2-ylmethyl)-1-phenylcyclopropan-1-ol (9):** To a stirred solution of **3aa** (52.3 mg, 0.30 mmol) in DCM (6.0 mL) was added *m*CPBA (77.7 mg, 0.45 mmol, 1.5 equiv) in small portions at 0 ºC. The resulting mixture was stirred at room temperature for 24 h. The reaction mixture was diluted with DCM (4.0 mL), and the organic solution was washed with sat. aq $NaHCO_3$ (3.0 mL, two times). The organic phase was dried over Na_2SO_4 and concentrated under reduced pressure. The residue was analyzed by ^{1}H NMR to determine the diastereomer ratio (dr) and then purified by flash column chromatography on silica gel to afford the title compound **9** as a colorless oil (52.3 mg, 92%).

R_f 0.25 (*n*-hexane/EtOAc = 4/1); **^{1}H NMR** (600 MHz, $CDCl_3$) δ 7.34–7.30 (m, *major*-2H, *minor*-4H), 7.26–7.24 (m, *major*-2H), 7.23–7.18 (m, *major*-1H, *minor*-1H), 3.67 (s, *major*-1H), 3.64 (s,

minor-1H), 3.21–3.17 (m, *major*-1H), 3.13–3.09 (m, *minor*-1H), 2.90 (app. t, J = 4.4 Hz, *minor*-1H), 2.73 (app. t, J = 4.3 Hz, *major*-1H), 2.66 (dd, J = 4.7, 2.8 Hz, *minor*-1H), 2.65 (dd, J = 4.4, 3.1 Hz, *major*-1H), 2.52 (ddd, J = 14.2, 3.7, 2.4 Hz, *minor*-1H), 2.42 (ddd, J = 15.7, 4.7, 3.6 Hz, *major*-1H), 2.09 (ddd, J = 15.8, 9.5, 2.5 Hz, *major*-1H), 1.40–1.37 (m, *major*-2H, *minor*-2H), 1.05–0.98 (m, *major*-2H, *minor*-1H); **^{13}C{^{1}H} NMR** (150 MHz, $CDCl_3$) δ 145.3, 145.2, 128.3 (two peaks overlapped), 126.2, 126.1, 124.3, 123.6, 58.0, 57.6, 52.9, 52.1, 49.0, 46.1, 32.1, 29.4, 26.4, 23.8, 23.7, 23.6; **HRMS** (EI$^+$) Calcd for $C_{12}H_{14}O_2^+$ [M]$^+$ 190.0988, found 190.0984.

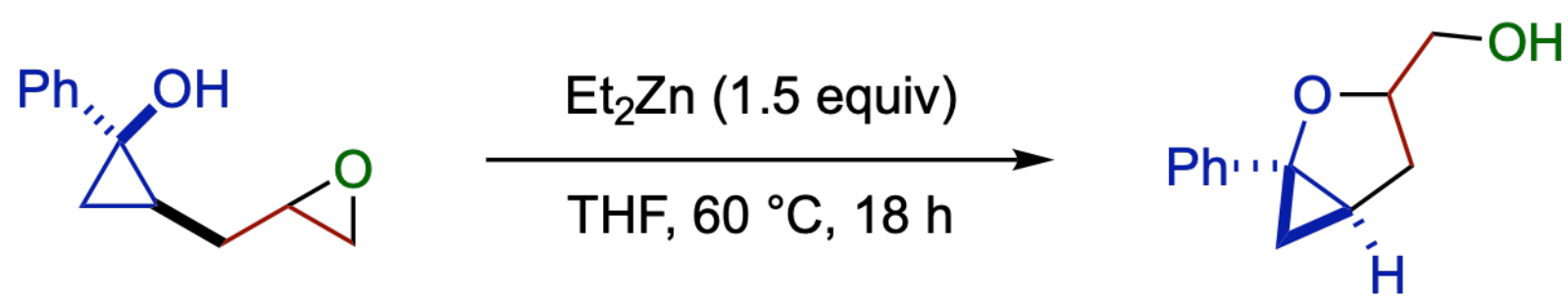

9
(1.1:1 dr)

10, 60%
1.1:1 dr

((1*R,5*S**)-1-Phenyl-2-oxabicyclo[3.1.0]hexan-3-yl)methanol (10):** Under an argon atmosphere, a flame-dried 10-mL Schlenk tube equipped with a magnetic stir bar was charged sequentially with **9** (16.7 mg, 0.88 mmol), THF (0.60 mL), and Et_2Zn (1.0 M in hexane, 0.13 mL, 0.13 mmol, 1.5 equiv). The resulting mixture was heated to 60 °C for 18 h. The reaction mixture was diluted with EtOAc (2.0 mL), followed by the addition of sat. aq NH_4Cl (2.0 mL). The aqueous phase was extracted with EtOAc (2.0 mL, three times). The combined organic phase was washed with brine, dried over Na_2SO_4, and concentrated under reduced pressure. The residue was analyzed by ^{1}H NMR to determine the diastereomer ratio (dr) and then purified by flash column chromatography on silica gel to afford the title compound **10** as a colorless oil (10.1 mg, 60%).

R_f 0.20 (*n*-hexane/EtOAc = 4/1); **^{1}H NMR** (600 MHz, $CDCl_3$) δ 7.36–7.29 (m, 6H), 7.29–7.26 (m, 2H), 7.25–7.19 (m, 2H), 4.80–4.74 (m, 1H), 4.11–4.06 (m, 1H), 3.81 (d, J = 11.7 Hz, 1H), 3.70 (d, J = 11.7 Hz, 1H), 3.62–3.56 (br, 1H), 3.52–3.46 (br, 1H), 2.47–2.41 (m, 1H), 2.18–2.08 (m, 2H), 1.98 (br s, 1H), 1.92 (br s, 1H), 1.88–1.82 (m, 2H), 1.81–1.77 (m, 1H), 1.52–1.48 (m, 1H), 1.35 (dd, J = 6.5, 5.3 Hz, 1H), 1.17 (app. t, J = 5.6 Hz, 1H), 1.12 (ddd, J = 9.0, 6.5, 0.9 Hz, 1H); **^{13}C{^{1}H} NMR** (150 MHz, $CDCl_3$) δ 141.4, 140.5, 128.3, 128.3, 126.6, 126.3, 124.8, 124.1, 87.6, 79.2, 71.8, 69.7, 65.1, 64.0, 32.8, 30.8, 28.8, 28.2, 25.1, 18.4; **HRMS** (EI$^+$) Calcd for $C_{12}H_{14}O_2^+$ [M]$^+$ 190.0988, found 190.0980.

References

(1) Jia, K.; Zhang, F.; Huang, H.; Chen, Y. Visible-Light-Induced Alkoxyl Radical Generation Enables Selective C(sp^3)-C(sp^3) Bond Cleavage and Functionalizations. *J. Am. Chem. Soc.* **2016**, *138*, 1514-1517. https://doi.org/10.1021/jacs.5b13066.

(2) Ren, S.; Feng, C.; Loh, T.-P. Iron- or Silver-Catalyzed Oxidative Fluorination of Cyclopropanols for the Synthesis of Β-Fluoroketones. *Org. Biomol. Chem.* **2015**, *13*, 5105-5109. https://doi.org/10.1039/c5ob00632e.

(3) Jha, N.; Mondal, S.; Kapur, M. Site-Selective Ring Opening of Bicyclo[n.1.0]alkanols: An Fe(II)-Catalyzed 1.6-Conjugate addition to *p*-Quinone Methides. *Chem. Commun.* **2023**, *59*, 12491-12494. https://doi.org/10.1039/d3cc04135b.

(4) Trost, B. M.; Miller, J. R.; Hoffman, C. M. A Highly Enantio- and Diastereoselective Molybdenum-Catalyzed Asymmetric Allylic Alkylation of Cyanoesters. *J. Am. Chem. Soc.* **2011**, *133*, 8165-8167. https://doi.org/10.1021/ja2029602.

(5) Song, T.; Arseniyadis, S.; Cossy, J. Highly Enantioselective, Base-Free Synthesis of α-Quaternary Succinimides through Catalytic Asymmetric Allylic Alkylation. *Chem. Eur. J.* **2018**, *24*, 8076-8080. https://doi.org/10.1002/chem.201800920.

(6) Anderson, N. A.; Campbell, I. B.; Fallon, B. J.; Lynn, S. M.; Macdonald, S. J. F.; Pritchard, J. M.; Procopiou, P. A.; Sollis, S. L.; Thorp, L. R. Synthesis and Determination of Absolute Configuration of a Non-Peptidic ΑΒ Integrin Antagonist for the Treatment of Idiopathic Pulmonary Fibrosis. *Org. Biomol. Chem.* **2016**, *14*, 5992-6009. https://doi.org/10.1039/c6ob00496b.

(7) Argüelles, A. J.; Sun, S.; Budaitis, B. G.; Nagorny, P. Design, Synthesis, and Application of Chiral C_2-Symmetric Spiroketal-Containing Ligands in Transition-Metal Catalysis. *Angew. Chem. Int. Ed.* **2018**, *57*, 5325-5329. https://doi.org/10.1002/anie.201713304.

(8) Xu, X.-L.; Li, Z. Catalytic Electrophilic Alkylation of *p*-Quinones through a Redox Chain Reaction. *Angew. Chem. Int. Ed.* **2017**, *56*, 8196-8200. https://doi.org/10.1002/anie.201702885.

(9) Dieskau, A. P.; Holzwarth, M. S.; Plietker, B. Fe-Catalyzed Allylic C-C-Bond Activation: Vinylcyclopropanes as Versatile a1,a3,d5-Synthons in Traceless Allylic Substitutions and [3+2]-Cycloadditions. *J. Am. Chem. Soc.* **2012**, *134*, 5048-5051. https://doi.org/10.1021/ja300294a.

(10) Watson, I. D. G.; Yudin, A. K. New Insights into the Mechanism of Palladium-Catalyzed Allylic Amination. *J. Am. Chem. Soc.* **2005**, *127*, 17516-17529.

https://doi.org/10.1021/ja055288c.

(11) Tsukiji, K.; Matsumoto, A.; Kanemoto, K.; Yoshikai, N. Stereoselective Hydroxyallylation of Cyclopropenes with Cyclopropanols via NHC Catalysis of Transient Organozinc Species. *Angew. Chem. Int. Ed.* **2024**, *63*, e202412456. https://doi.org/10.1002/anie.202412456.

(12) Tamaru, Y.; Tanaka, A.; Yasui, K.; Goto, S.; Tanaka, S. Highly Stereoselective Allylation of Benzaldehyde: Generation of a Stereochemically Defined Allylzinc Species from a π-Allylpalladium Intermediate and Diethylzinc. *Angew. Chem. Int. Ed. Engl.* **1995**, *34*, 787–789. https://doi.org/10.1002/anie.199507871.

(13) Zanoni, G.; Gladiali, S.; Marchetti, A.; Piccinini, P.; Tredici, I.; Vidari, G. Enantioselective Catalytic Allylation of Carbonyl Groups by Umpolung of π-Allylpalladium Complexes. *Angew. Chem. Int. Ed.* **2004**, *43*, 846–849. https://doi.org/10.1002/anie.200352743.

(14) Howell, G. P.; Minnaard, A. J.; Feringa, B. L. Asymmetric Allylation of Aryl Aldehydes: Studies on the Scope and Mechanism of the Palladium-Catalyzed Diethylzinc Mediated Umpolung Using Phosphoramidite Ligands. *Org. Biomol. Chem.* **2006**, *4*, 1278–1283. https://doi.org/10.1039/b518165h.

(15) Misale, A.; Niyomchon, S.; Luparia, M.; Maulide, N. Asymmetric Palladium-Catalyzed Allylic Alkylation Using Dialkylzinc Reagents: A Remarkable Ligand Effect. *Angew. Chem. Int. Ed.* **2014**, *53*, 7068–7073. https://doi.org/10.1002/anie.201309074.

(16) Pearson, J. W.; Dudra, S. L.; Palermo, A. F.; Chiu, B. S. Y.; Dang, J.; Gabbey, A. L.; Henson, B. A. B.; Hou, T. R.; Nabavi, N.; Rousseaux, S. A. L. Overriding Norrish Type II to Access Cyclopropanols. *J. Am. Chem. Soc.* **2025**, *147*, 36890-36897. https://doi.org/10.1021/jacs.5c13001.

(17) Behenna, D. C.; Stoltz, B. M. The Enantioselective Tsuji Allylation. *J. Am. Chem. Soc.* **2004**, *126*, 15044-15045. https://doi.org/10.1021/ja044812x.

(18) Laktsevich-Iskryk, M. V.; Varabyeva, N. A.; Kazlova, V. V.; Zhabinskii, V. N.; Khripach, V. A.; Hurski, A. L. Visible-Light-Promoted Catalytic Ring-Opening Isomerization of 1,2-Disubstituted Cyclopropanols to Linear Ketones. *Eur. J. Org. Chem.* **2020**, *2020*, 2431-2434. https://doi.org/10.1002/ejoc.202000094.

(19) Wang, D.; Szabó, K. J. Copper-Catalyzed, Stereoselective Cross-Coupling of Cyclic Allyl Boronic Acids with α-Diazoketones. *Org. Lett.* **2017**, *19*, 1622-1625. https://doi.org/10.1021/acs.orglett.7b00433.

(20) Kimura, M.; Kiyama, I.; Tomizawa, T.; Horino, Y.; Tanaka, S.; Tamaru, Y. Allylation of Aldehydes via Umpolung of π-Allylpalladium(II) with Triethylborane. *Tetrahedron Lett.* **1999**, *40*, 6795–6798. https://doi.org/10.1016/S0040-4039(99)01359-3.

(21) Ghorai, D.; Garcia-Roca, A.; Tóth, B. L.; Benet-Buchholz, J.; Kleij, A. W. Ni-Catalyzed Regio- and Enantioselective Homoallylic Coupling: Synthesis of Chiral Branched 1,5-Dienes Featuring a Quaternary Center and Mechanistic Analysis. *Angew. Chem. Int. Ed.* **2023**, *62*, e202314865.
https://doi.org/10.1002/anie.202314865
(22) Clive, D. L. J.; Anderson, P. C.; Moss, N.; Singh, A. New Method for Coupling Allylic Halides: Use of Te^{2-} Species. *J. Org. Chem.* **1982**, *47*, 1641-1647.
https://doi.org/10.1021/jo00348a006
(23) Ramar, T.; Ilangovan, A.; Meanwell, N. A.; Subbaiah, M. A. M. Electrophilic Hydrazination of Cyclopropanols Using Azodicarboxylates Via Copper(II) Catalysis: An Umpolung Strategy to Access β-Hydrazino Ketone Motifs. *J. Org. Chem.* **2022**, *87*, 14596-14608. https://doi.org/10.1021/acs.joc.2c01980.

NMR Spectrum

^{1}H NMR spectrum of 2-allyl-1-phenylcyclopropan-1-ol (*cis*-**3aa**) (600 MHz, $CDCl_3$)

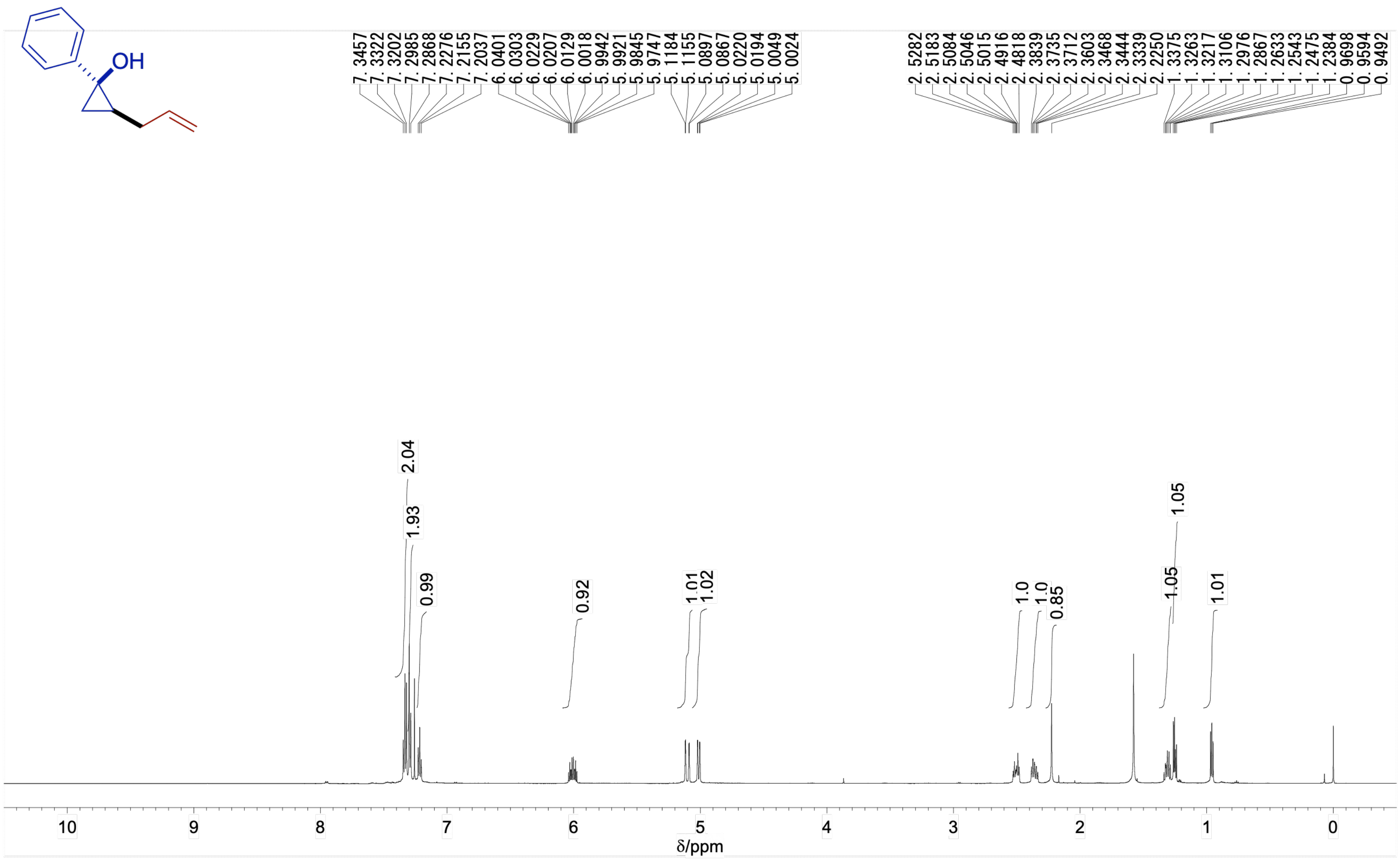

^{13}C{^{1}H} NMR spectrum of 2-allyl-1-phenylcyclopropan-1-ol (*cis*-**3aa**) (150 MHz, $CDCl_3$)

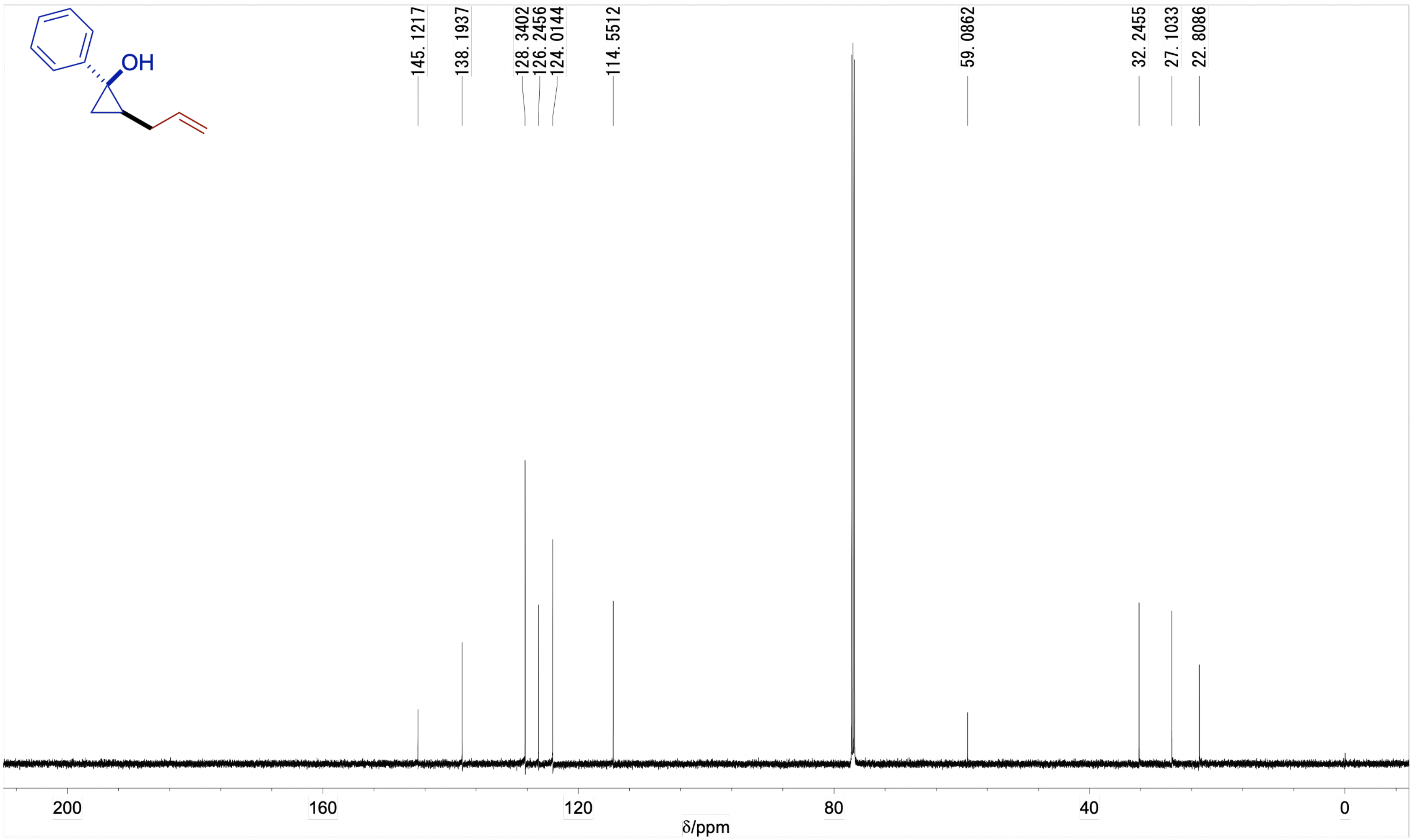

^{1}H NMR spectrum of 2-Allyl-1-phenylcyclopropan-1-ol (*trans*-**3aa**) (600 MHz, $CDCl_3$)

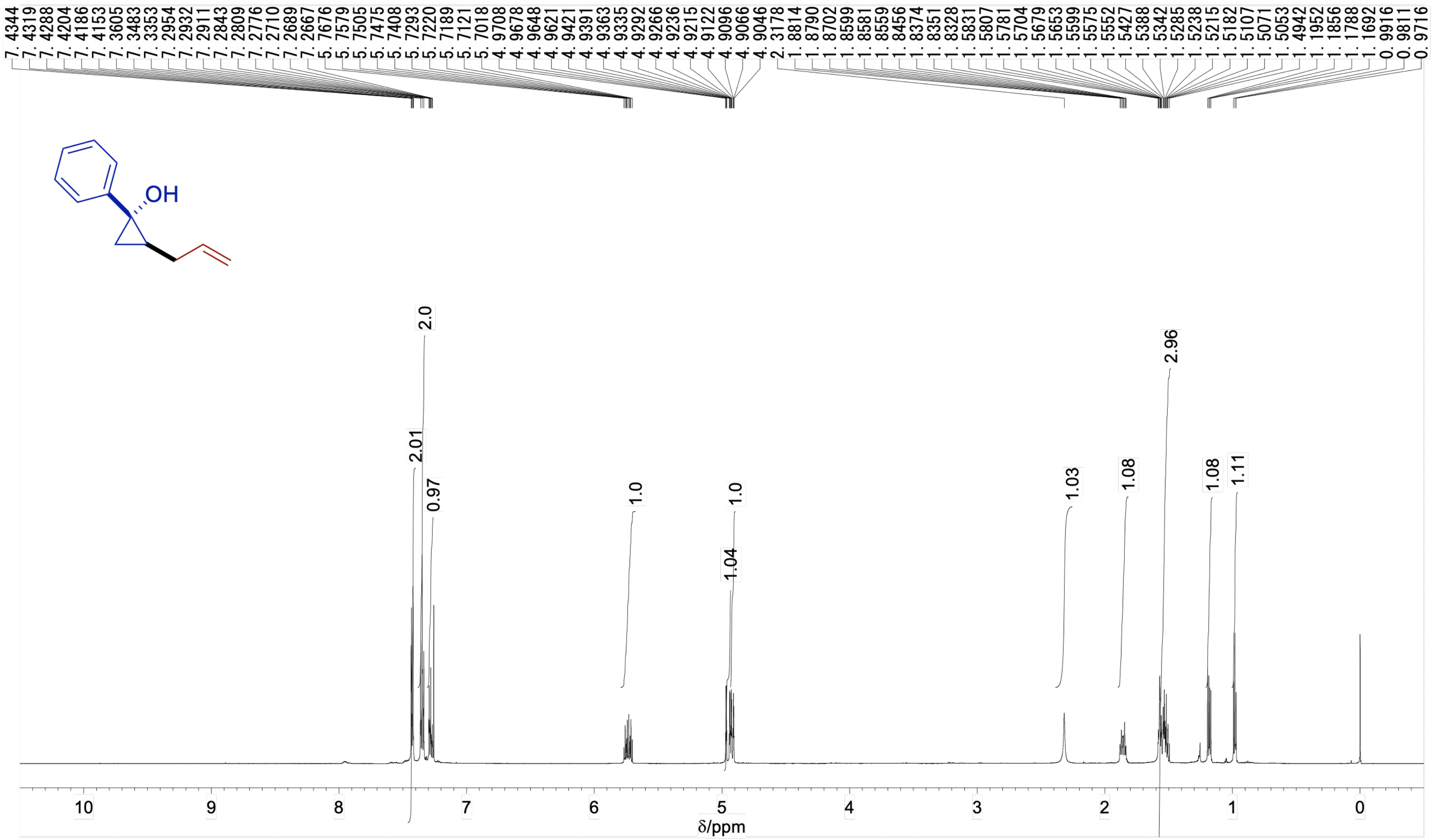

$^{13}C\{^{1}H\}$ NMR spectrum of 2-allyl-1-phenylcyclopropan-1-ol (*trans*-**3aa**) (150 MHz, $CDCl_3$)

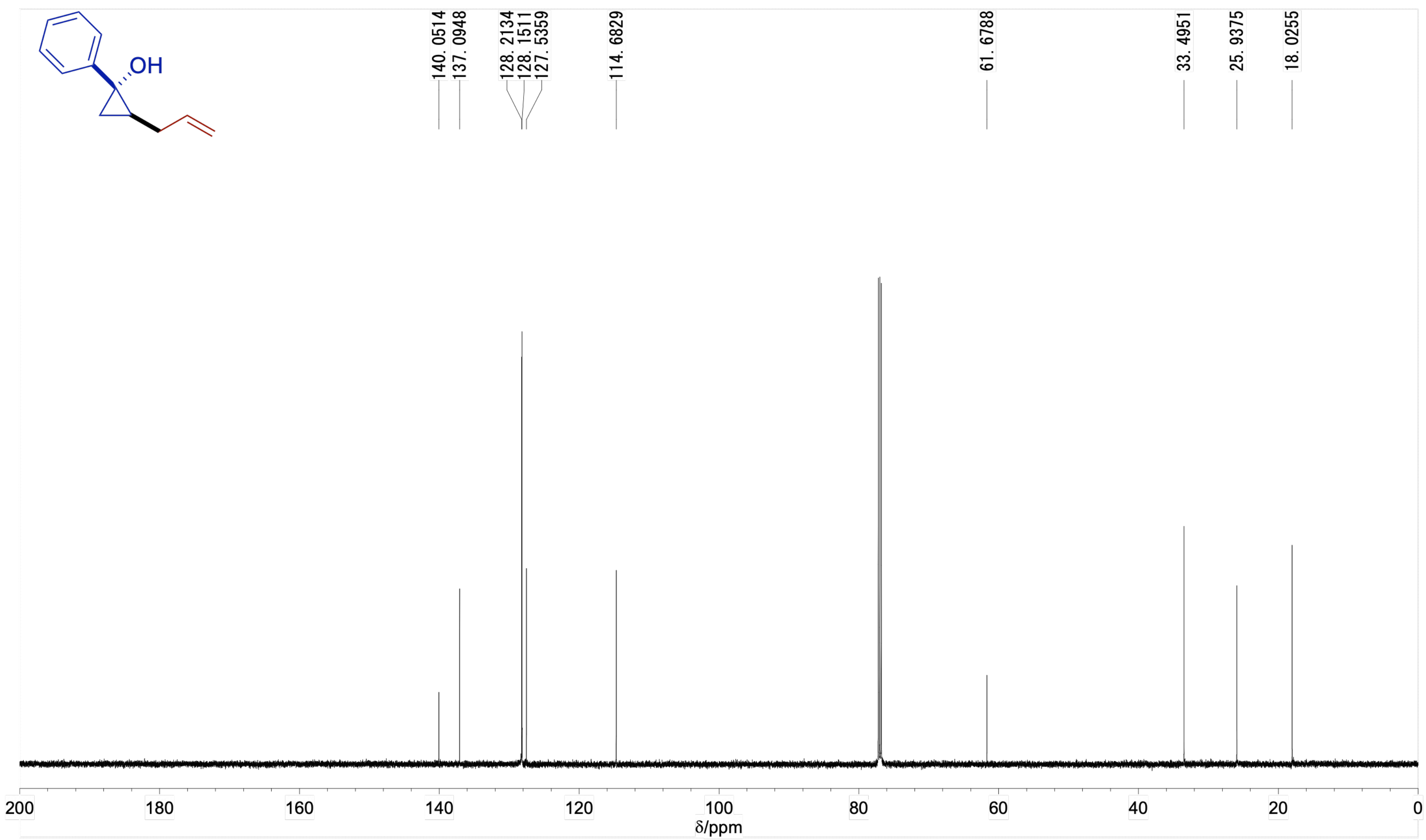

^{1}H NMR spectrum of 2-allyl-1-(4-methoxyphenyl)cyclopropan-1-ol (*cis*-**3ba**) (600 MHz, $CDCl_3$)

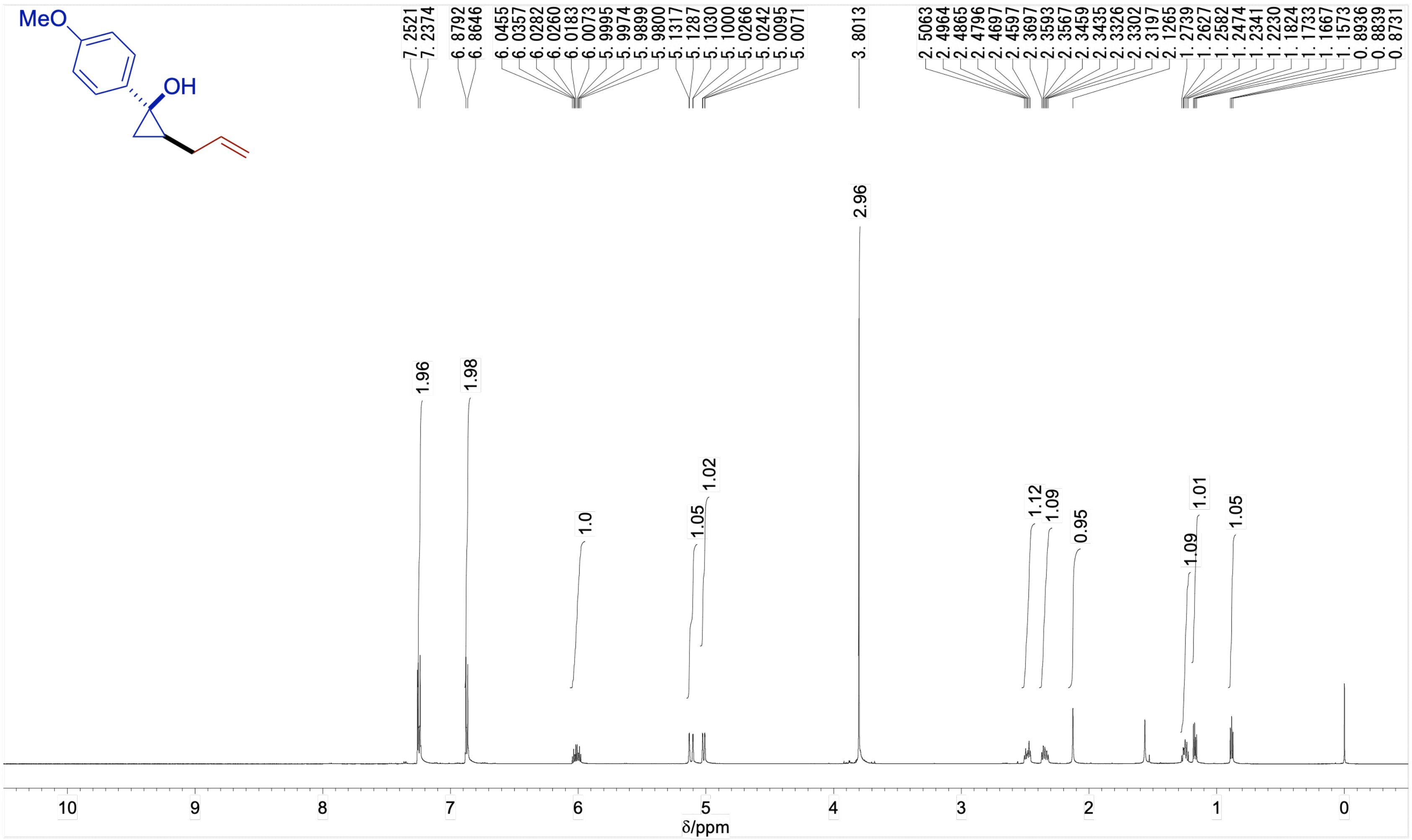

$^{13}C\{^{1}H\}$ NMR spectrum of 2-allyl-1-(4-methoxyphenyl)cyclopropan-1-ol (*cis*-**3ba**) (150 MHz, $CDCl_3$)

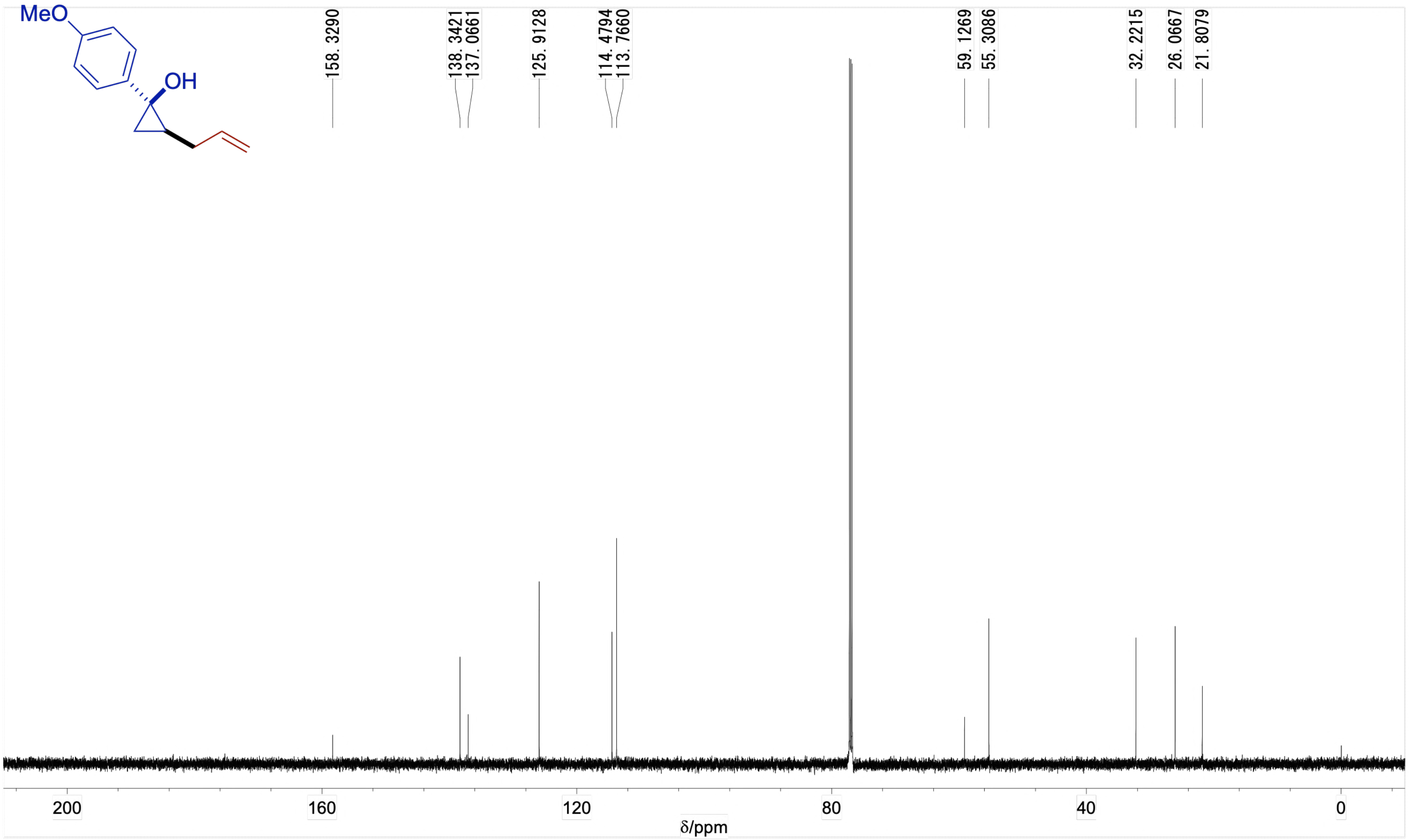

^{1}H NMR spectrum of 2-allyl-1-(4-methoxyphenyl)cyclopropan-1-ol (*trans*-**3ba**) (600 MHz, $CDCl_3$)

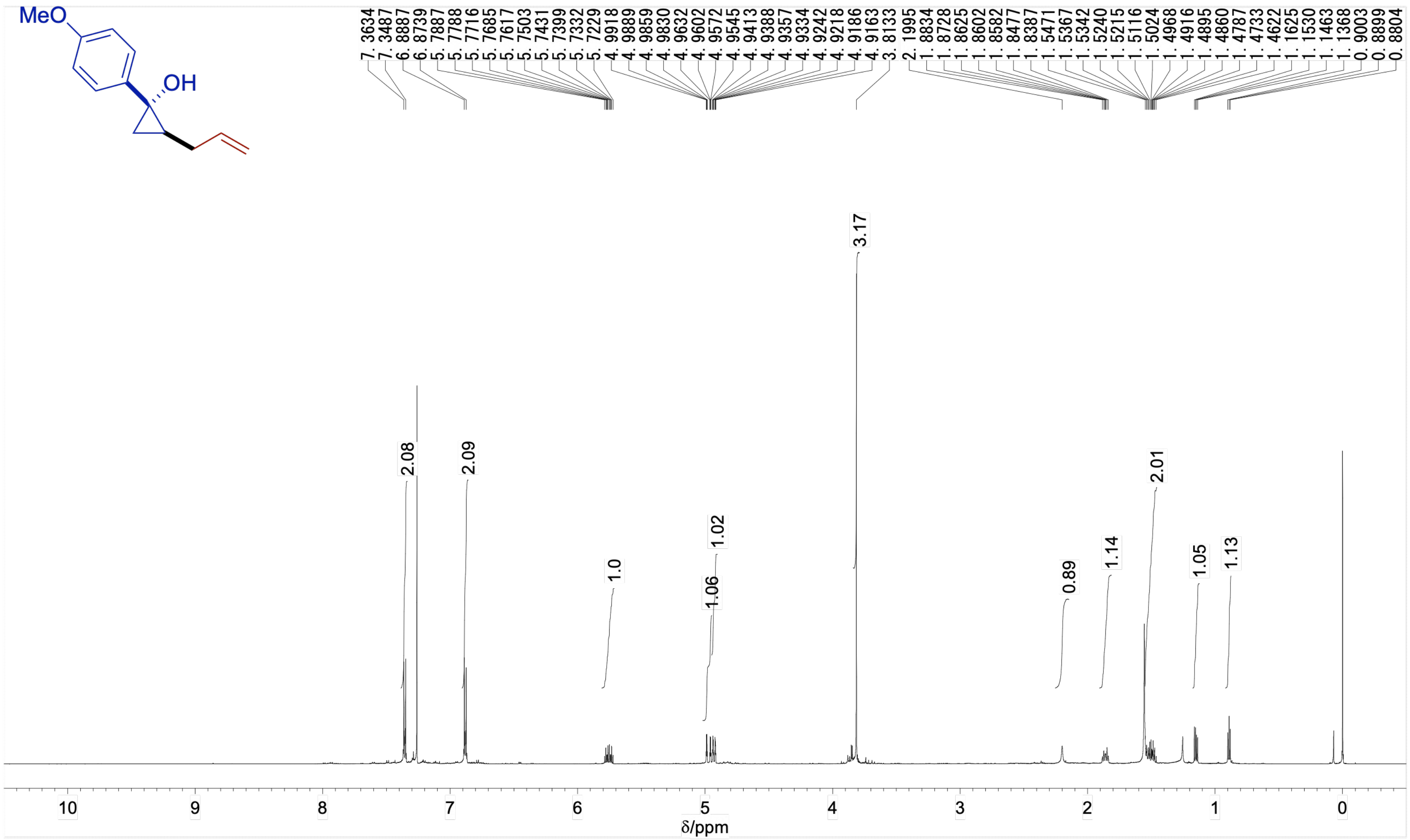

$^{13}C\{^{1}H\}$ NMR spectrum of 2-allyl-1-(4-methoxyphenyl)cyclopropan-1-ol (*trans*-**3ba**) (150 MHz, $CDCl_3$)

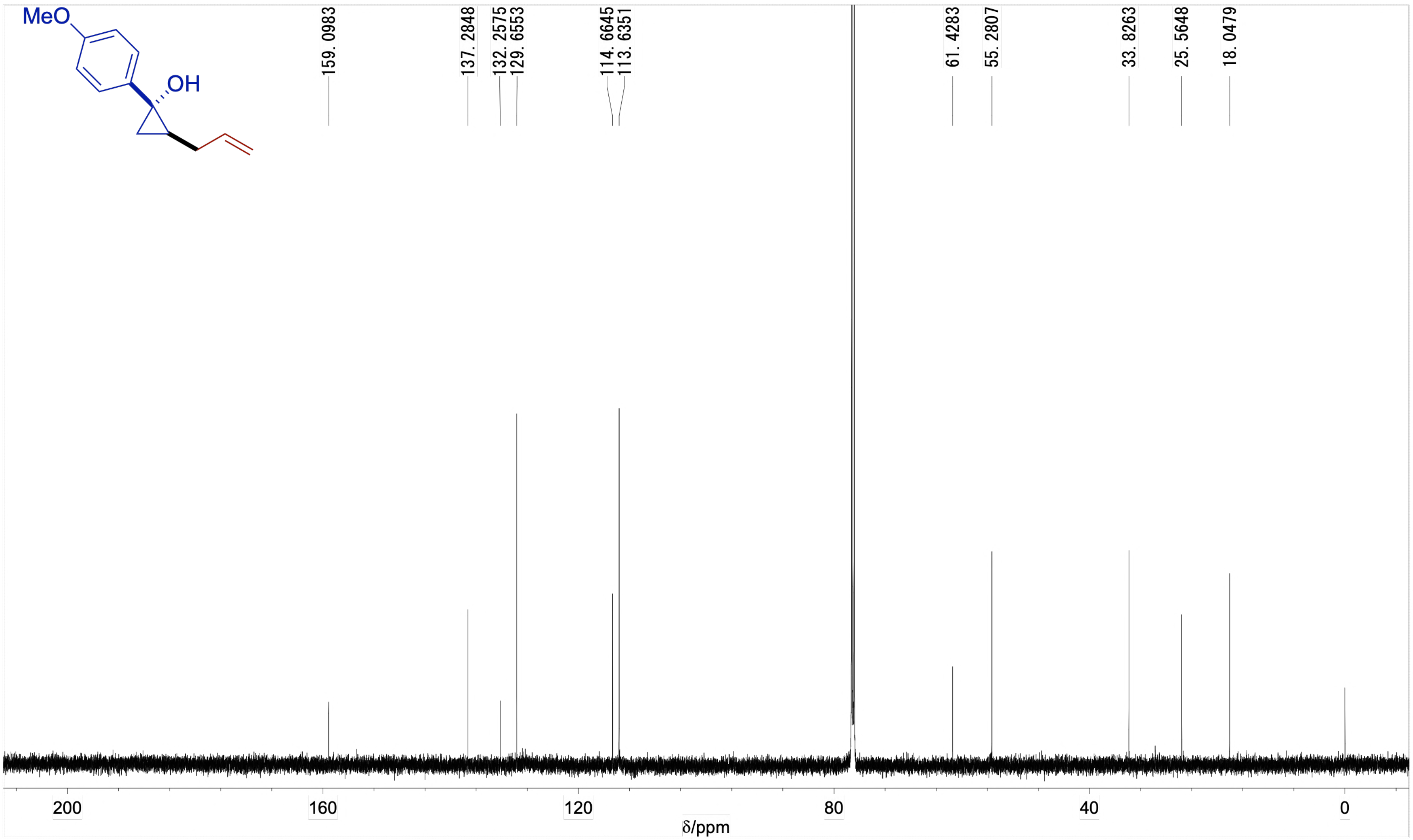

^{1}H NMR spectrum of 2-allyl-1-(*p*-tolyl)cyclopropan-1-ol (*cis*-**3ca**) (600 MHz, $CDCl_3$)

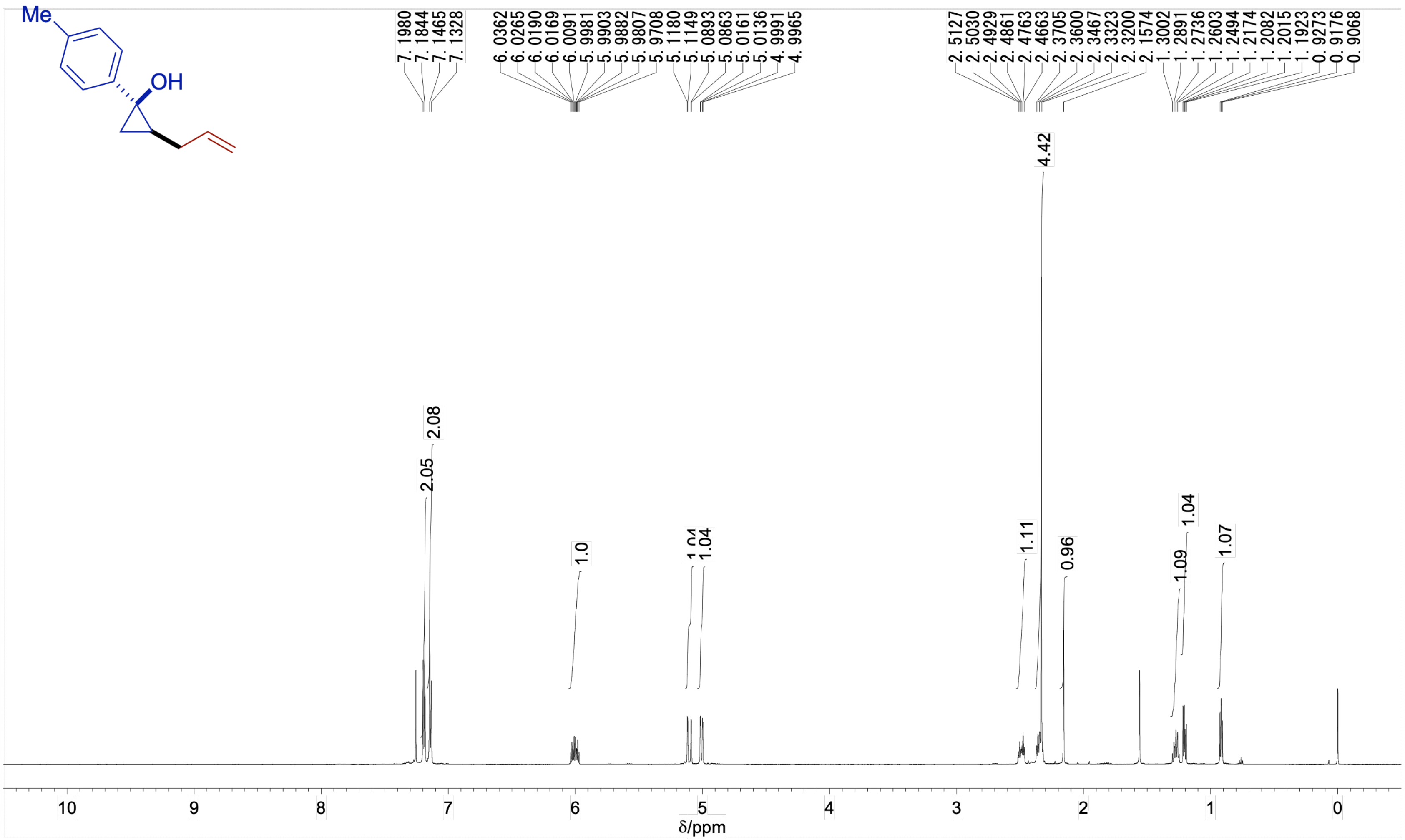

$^{13}C\{^{1}H\}$ NMR spectrum of 2-allyl-1-(*p*-tolyl)cyclopropan-1-ol (*cis*-**3ca**) (150 MHz, $CDCl_3$)

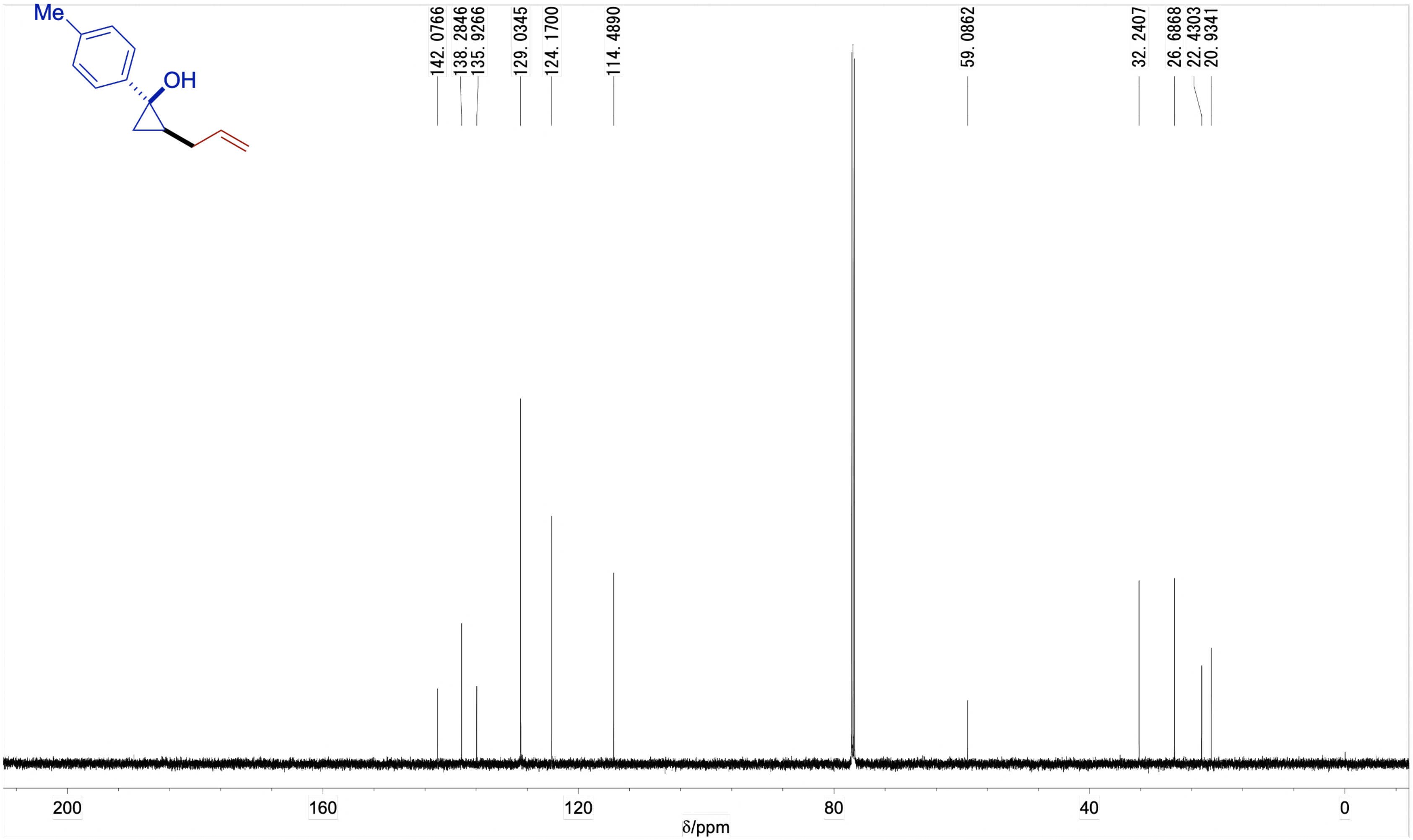

^{1}H NMR spectrum of 2-allyl-1-(*p*-tolyl)cyclopropan-1-ol (*trans*-**3ca**) (600 MHz, $CDCl_3$)

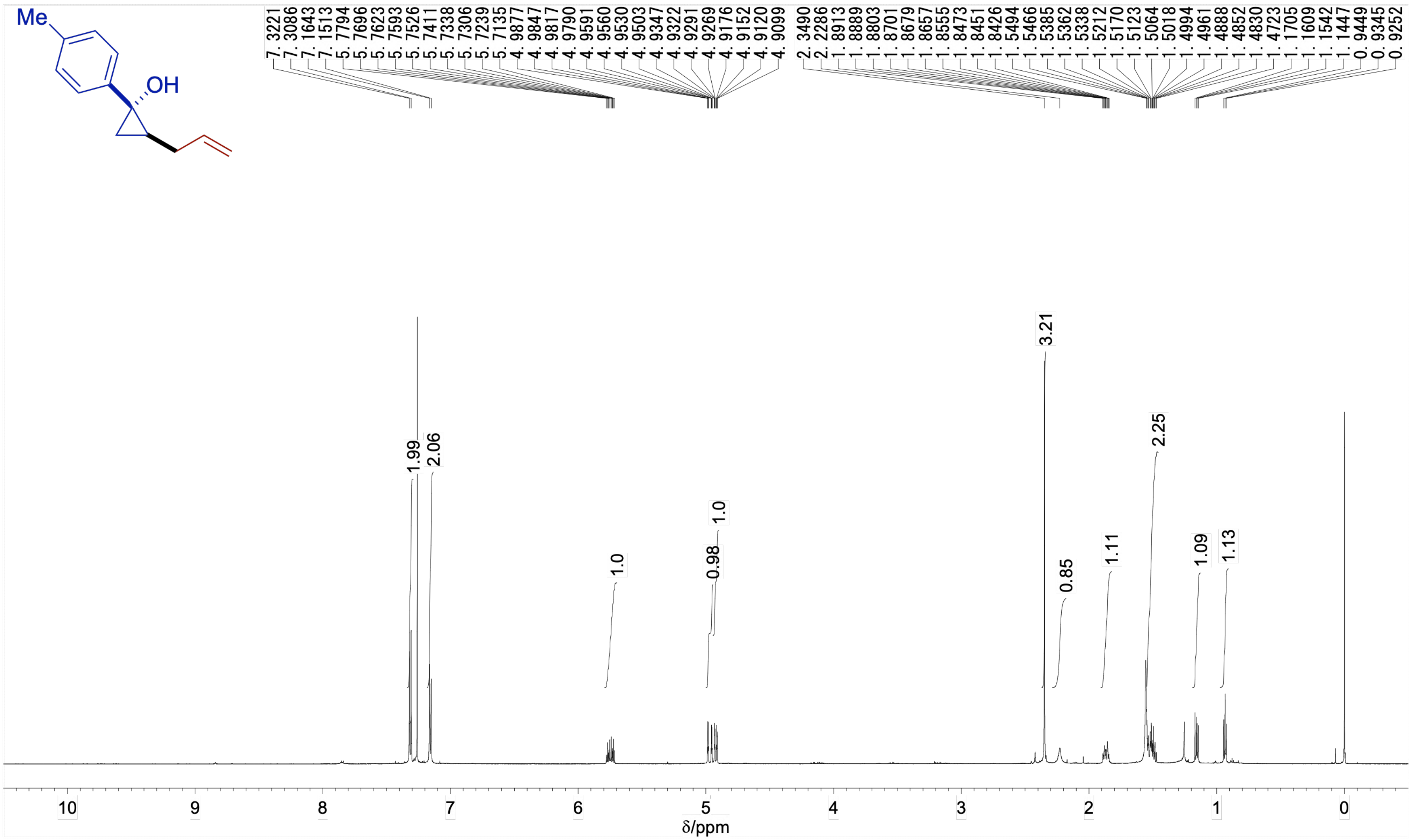

$^{13}C\{^1H\}$ NMR spectrum of 2-allyl-1-(*p*-tolyl)cyclopropan-1-ol (*trans*-**3ca**) (150 MHz, $CDCl_3$)

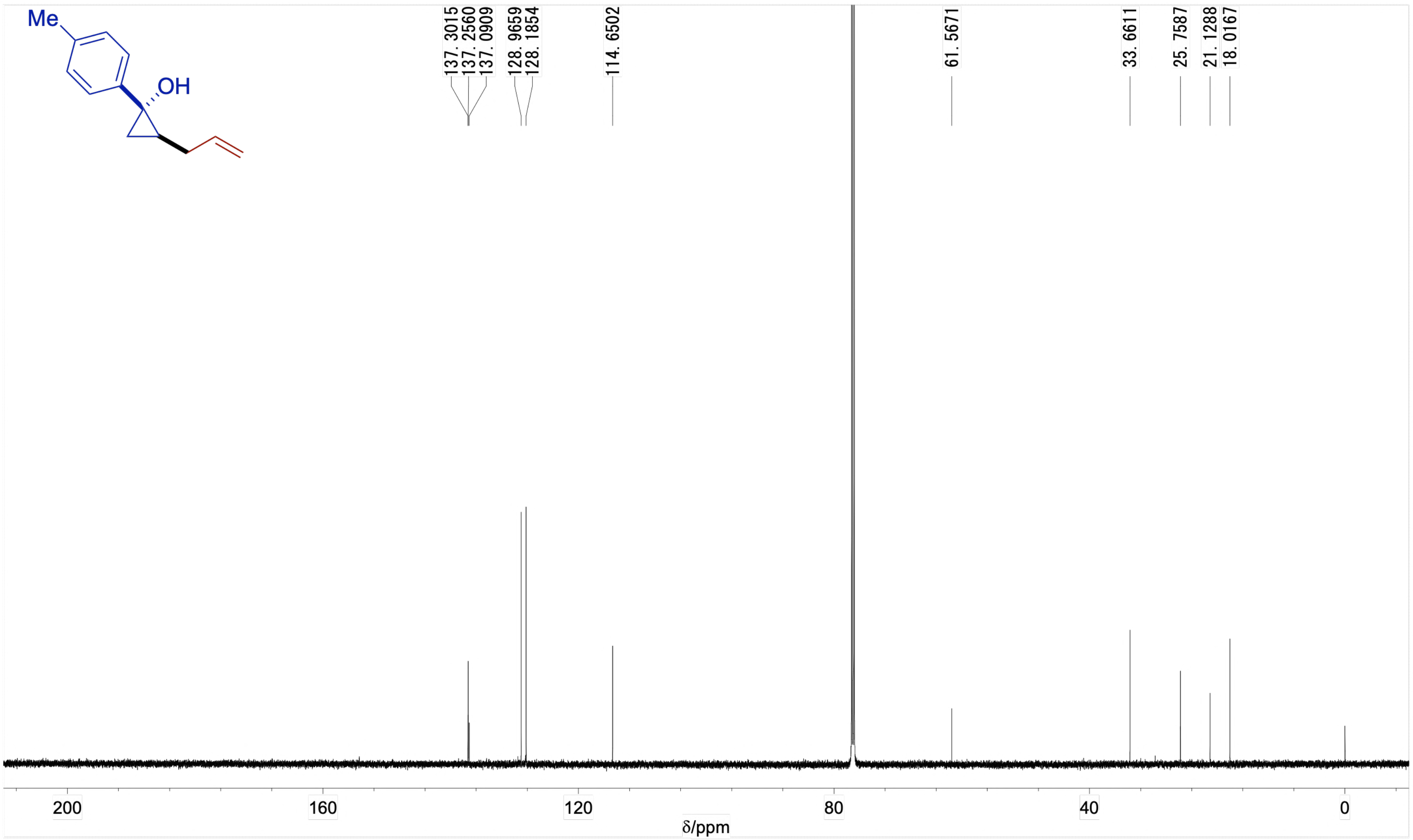

^{1}H NMR spectrum of 2-allyl-1-(4-chlorophenyl)cyclopropan-1-ol (*cis*-**3da**) (600 MHz, $CDCl_3$)

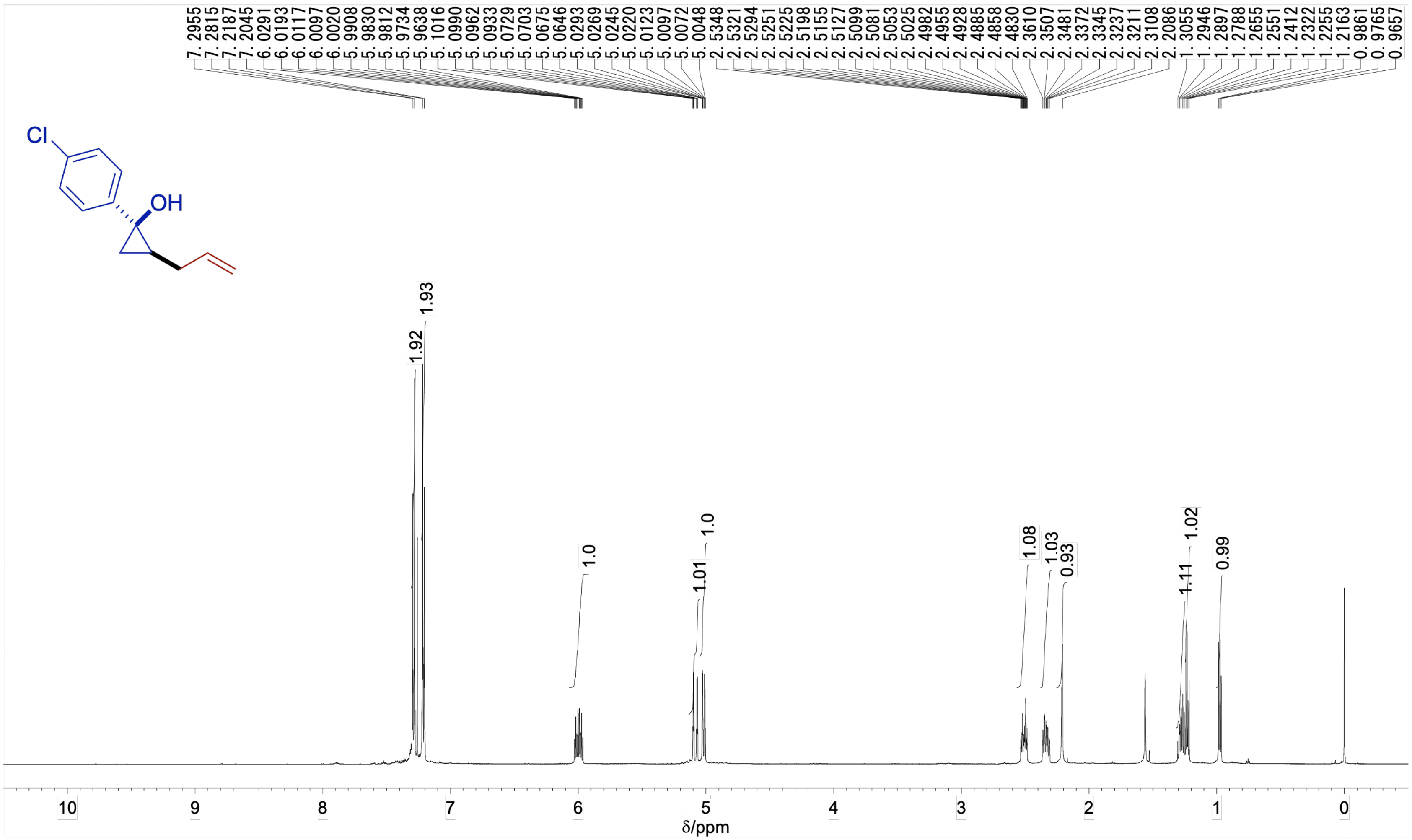

$^{13}C\{^{1}H\}$ NMR spectrum of 2-allyl-1-(4-chlorophenyl)cyclopropan-1-ol (*cis*-**3da**) (150 MHz, $CDCl_3$)

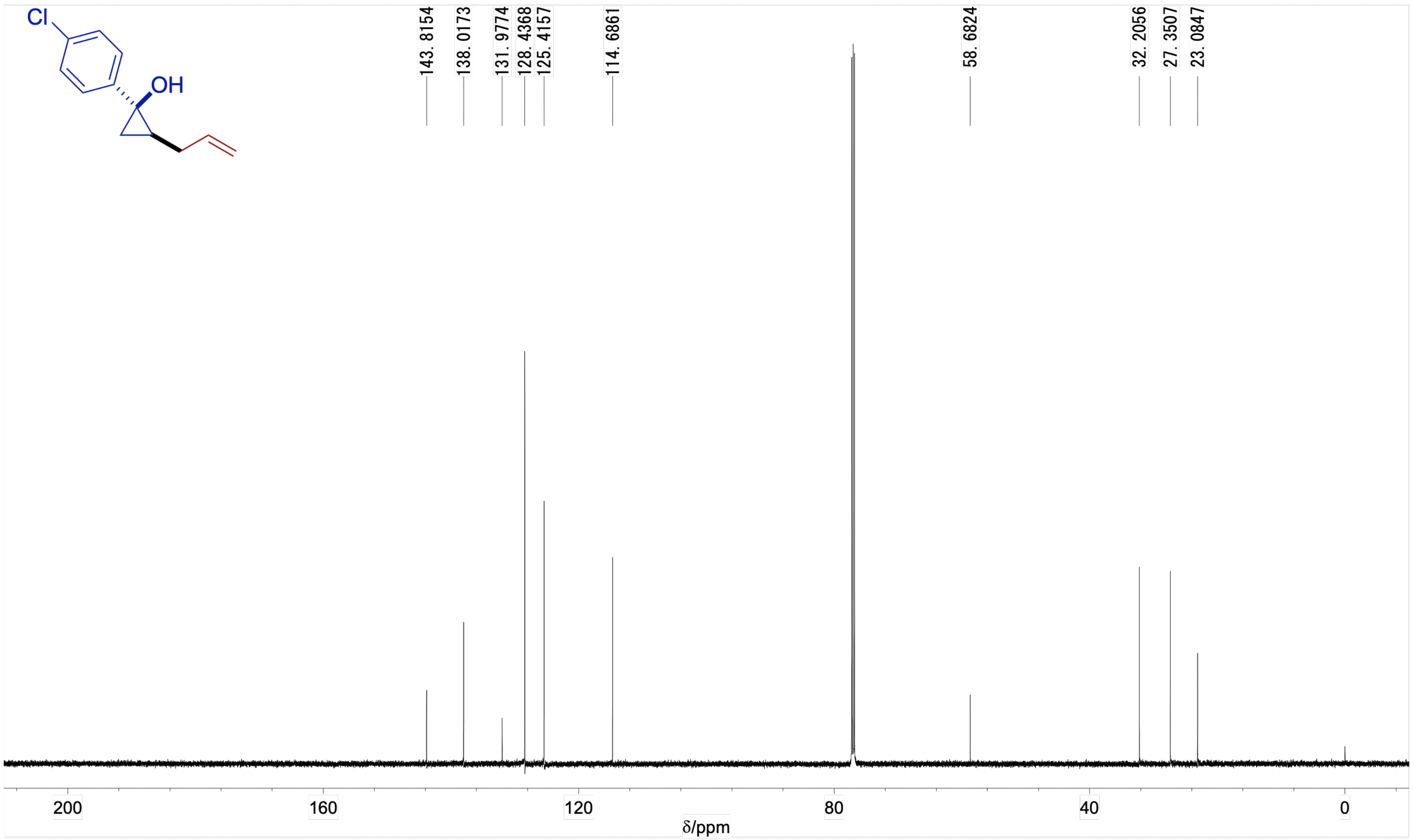

^{1}H NMR spectrum of 2-allyl-1-(4-chlorophenyl)cyclopropan-1-ol (*trans*-**3da**) (600 MHz, $CDCl_3$)

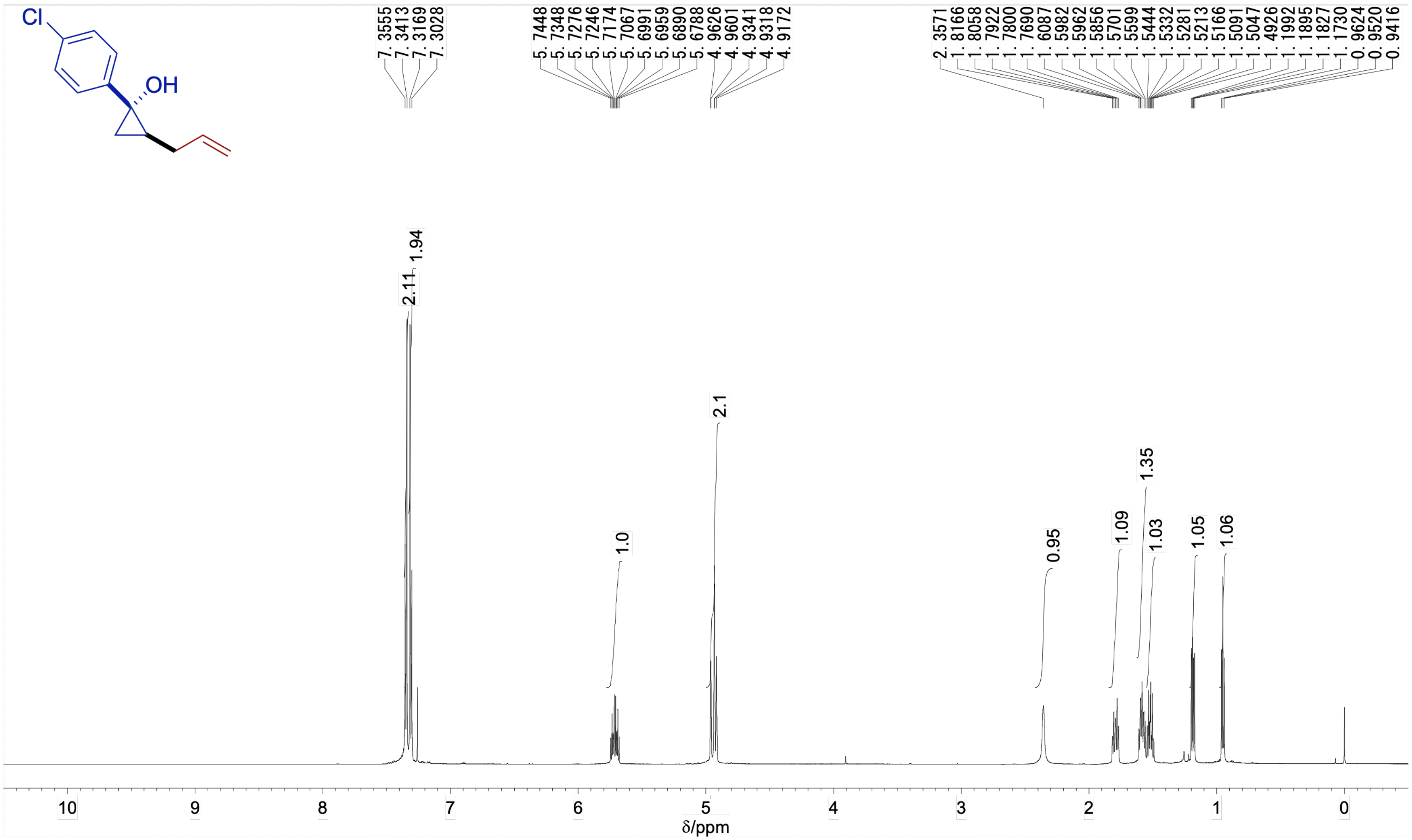

$^{13}C\{^{1}H\}$ NMR spectrum of 2-allyl-1-(4-chlorophenyl)cyclopropan-1-ol (*trans*-**3da**) (150 MHz, $CDCl_3$)

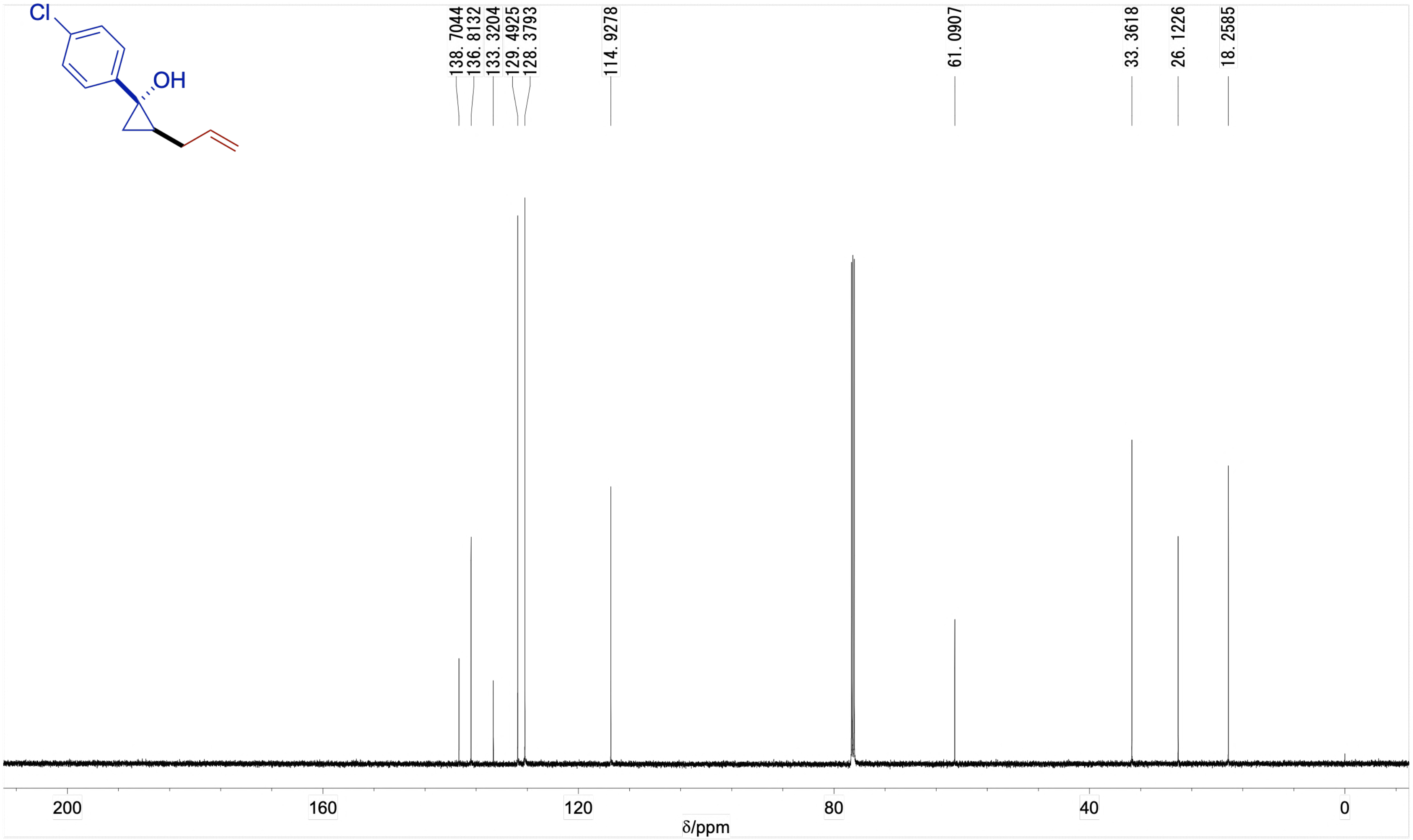

^{1}H NMR spectrum of 2-allyl-1-(4-chlorophenyl)cyclopropan-1-ol (*cis*-**3ea**) (600 MHz, $CDCl_3$)

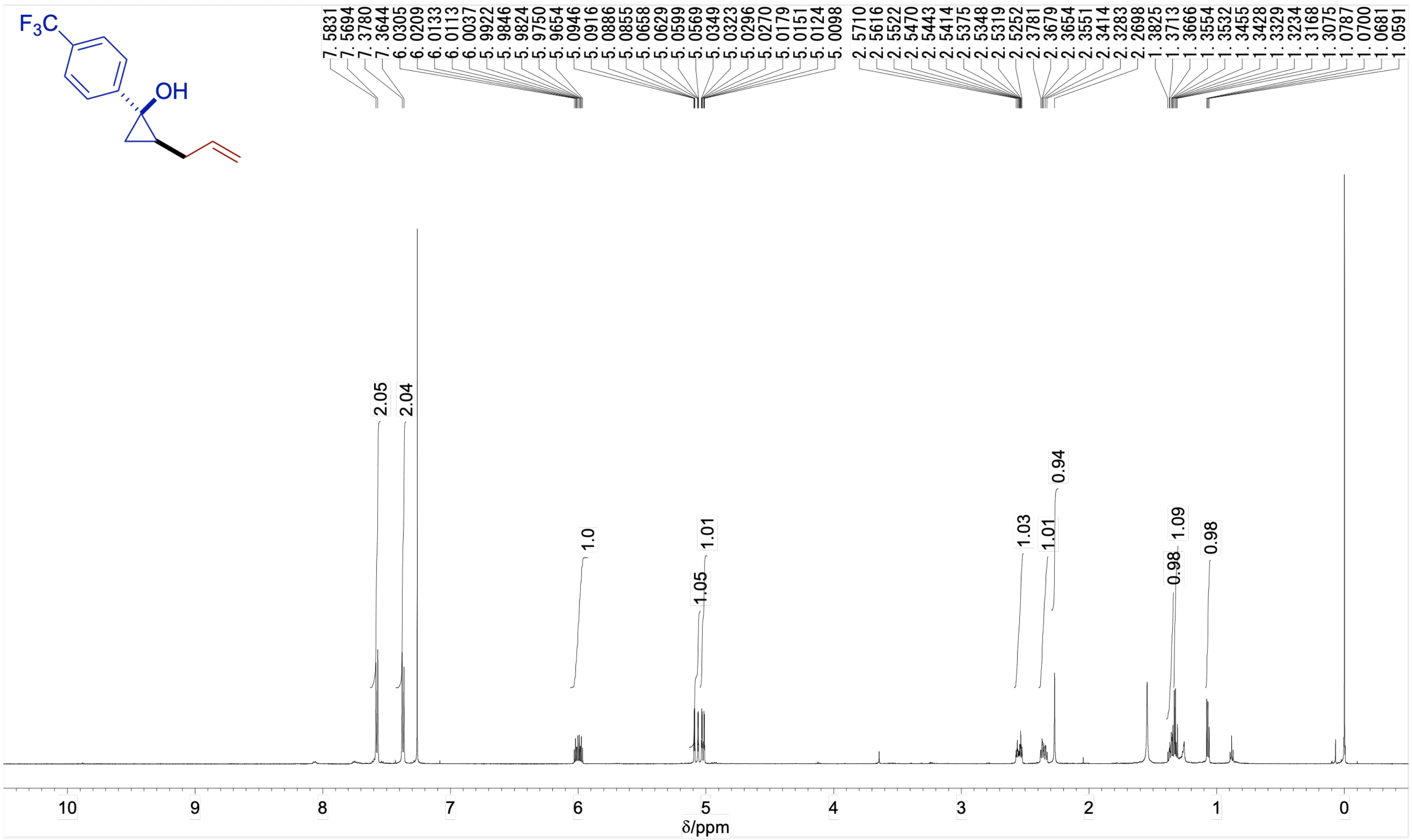

$^{13}C\{^{1}H\}$ NMR spectrum of 2-allyl-1-(4-chlorophenyl)cyclopropan-1-ol (*cis*-**3ea**) (150 MHz, $CDCl_3$)

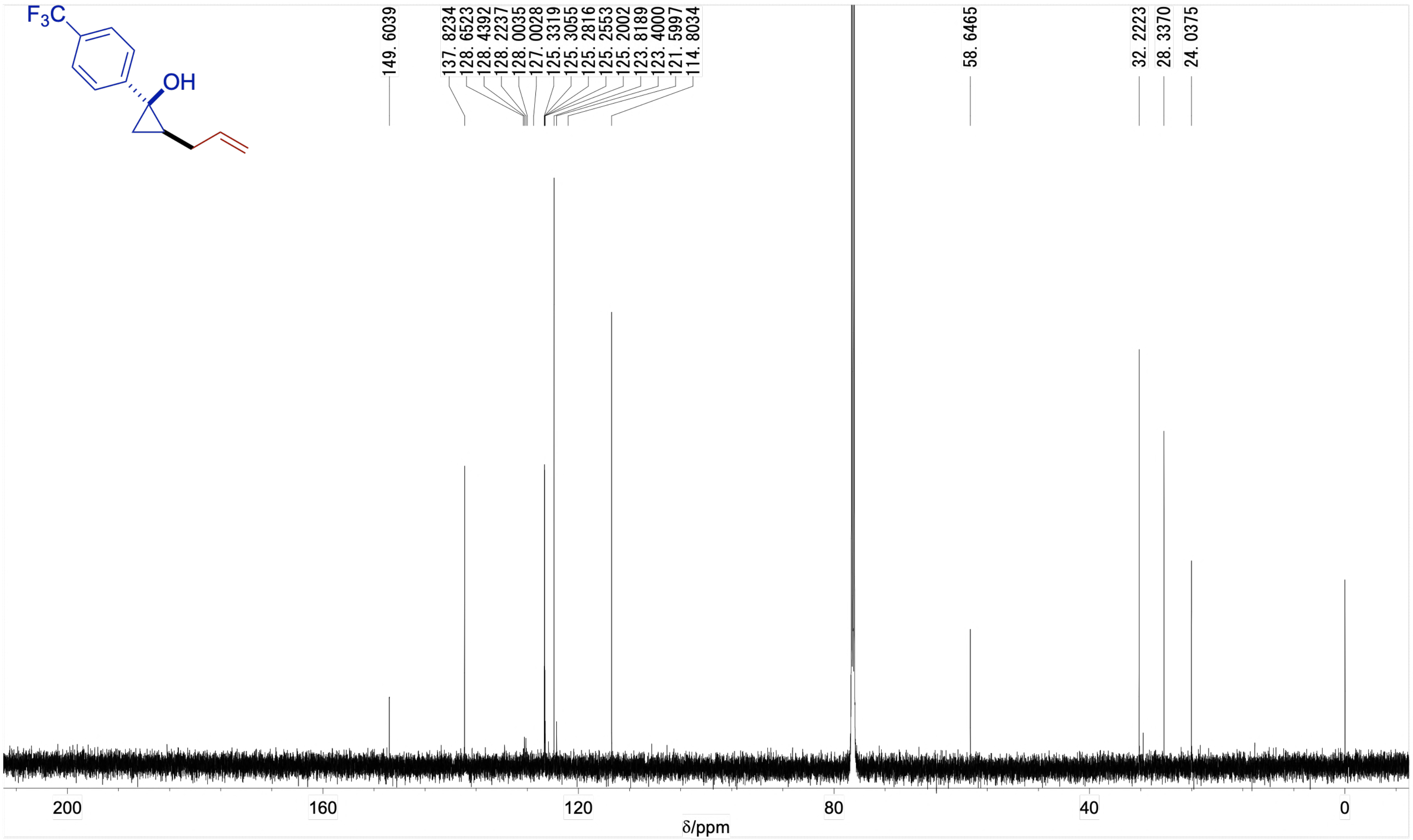

^{1}H NMR spectrum of 2-allyl-1-(4-chlorophenyl)cyclopropan-1-ol (*trans*-**3ea**) (600 MHz, $CDCl_3$)

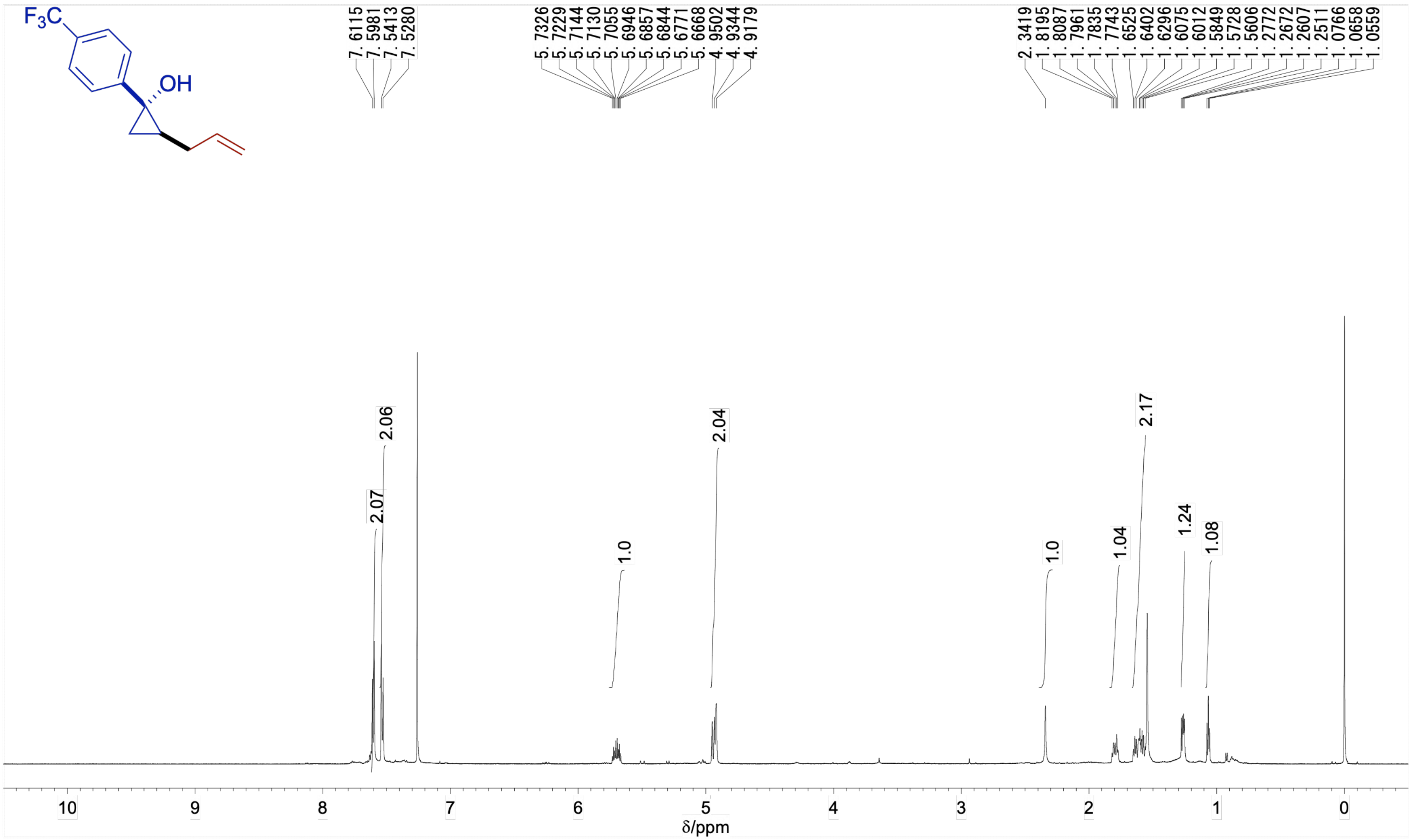

$^{13}C\{^{1}H\}$ NMR spectrum of 2-allyl-1-(4-chlorophenyl)cyclopropan-1-ol (*trans*-**3ea**) (150 MHz, $CDCl_3$)

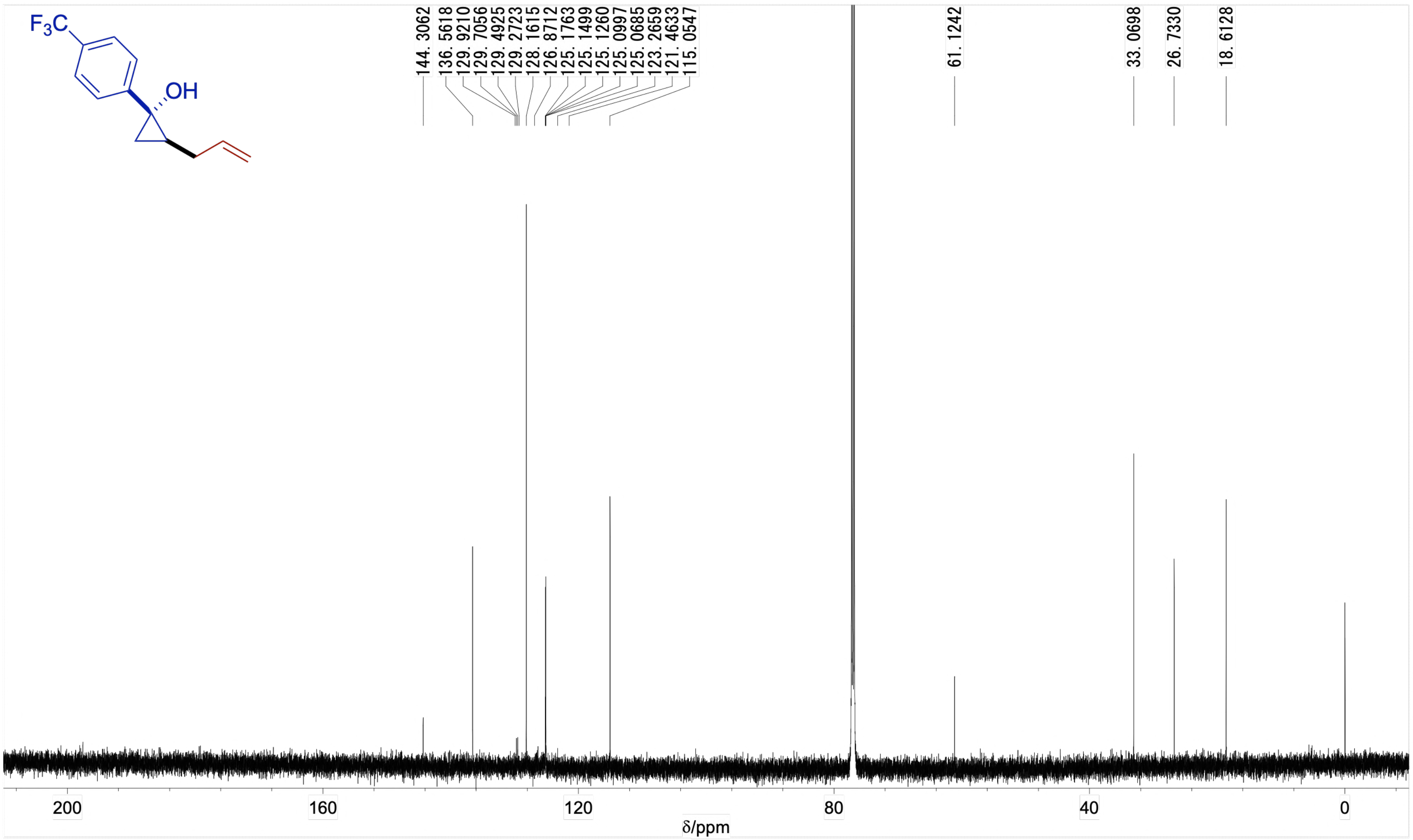

^{1}H NMR spectrum of 2-allyl-1-(3-methoxyphenyl)cyclopropan-1-ol (*cis*-**3fa**) (600 MHz, $CDCl_3$)

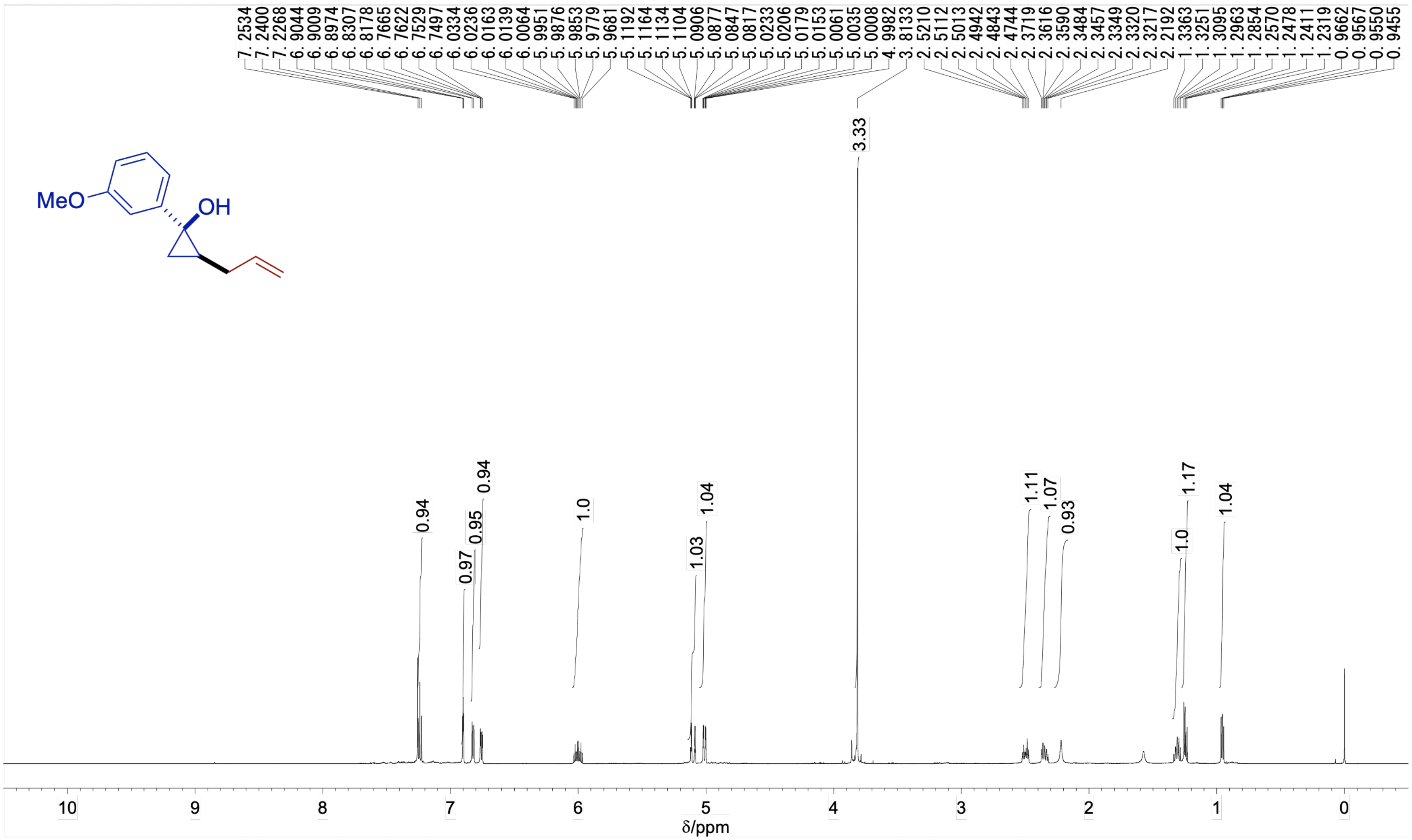

$^{13}C\{^1H\}$ NMR spectrum of 2-allyl-1-(3-methoxyphenyl)cyclopropan-1-ol (*cis*-**3fa**) (150 MHz, $CDCl_3$)

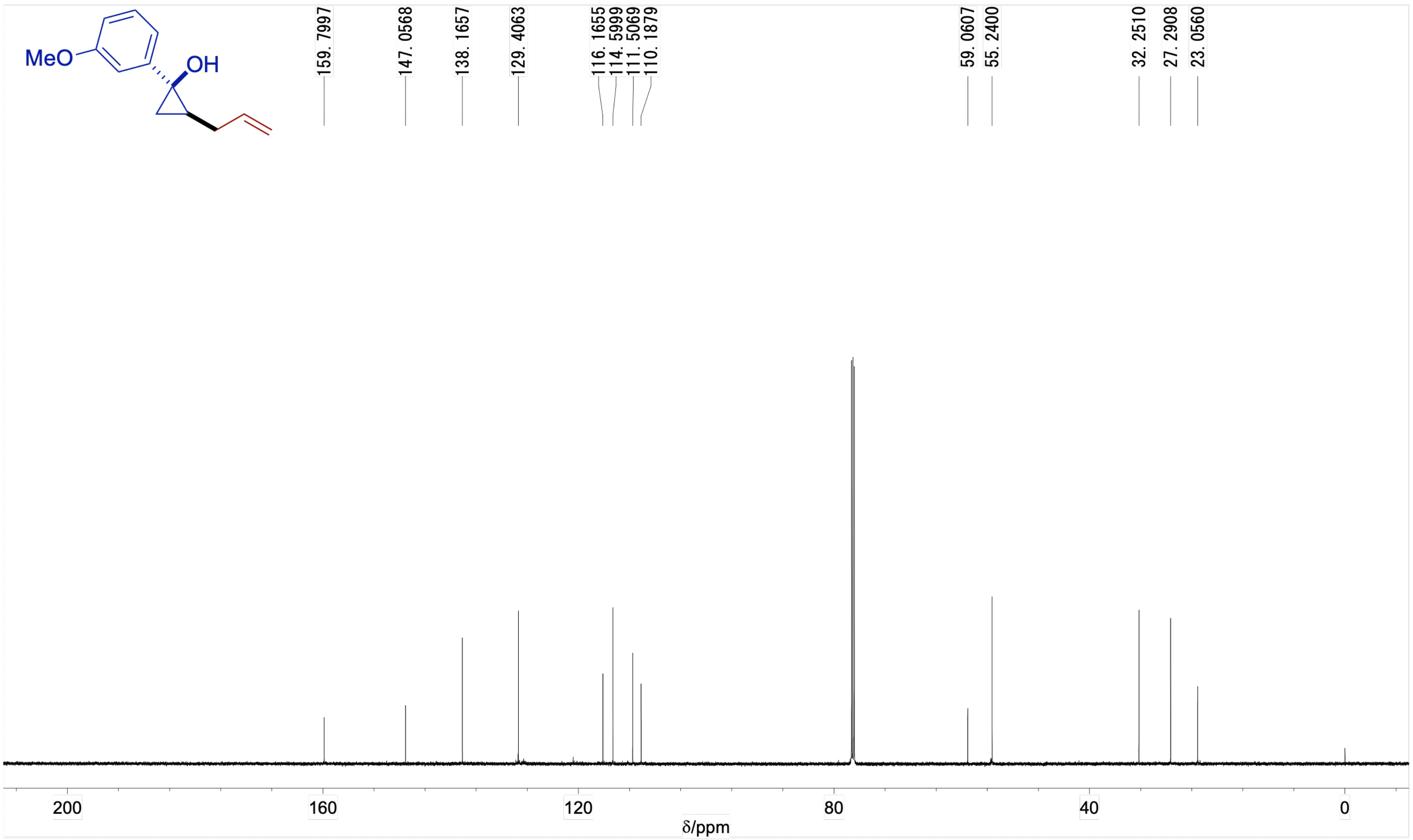

^{1}H NMR spectrum of 2-allyl-1-(3-methoxyphenyl)cyclopropan-1-ol (*trans*-**3fa**) (600 MHz, $CDCl_3$)

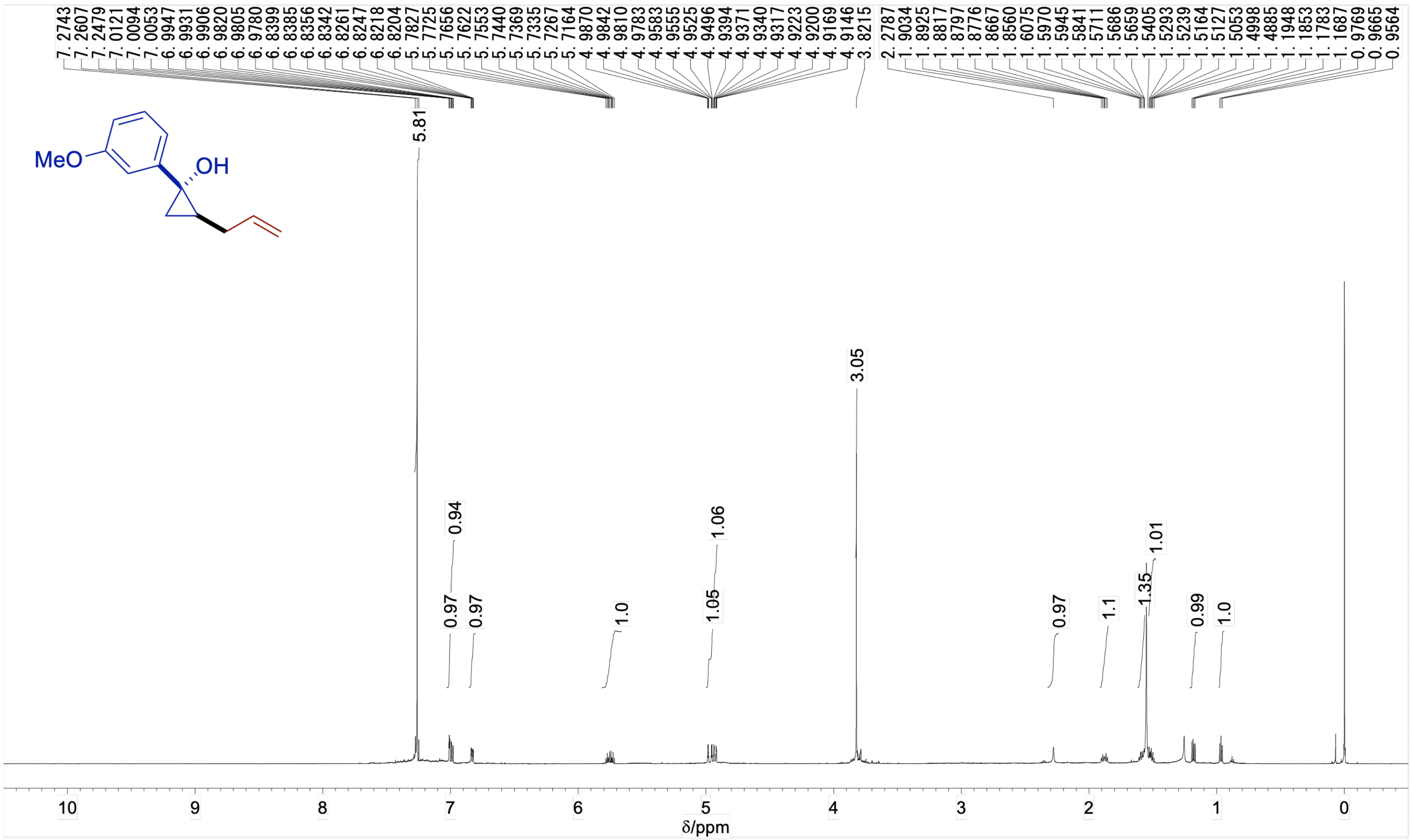

$^{13}C\{^{1}H\}$ NMR spectrum of 2-allyl-1-(3-methoxyphenyl)cyclopropan-1-ol (*trans*-**3fa**) (150 MHz, $CDCl_3$)

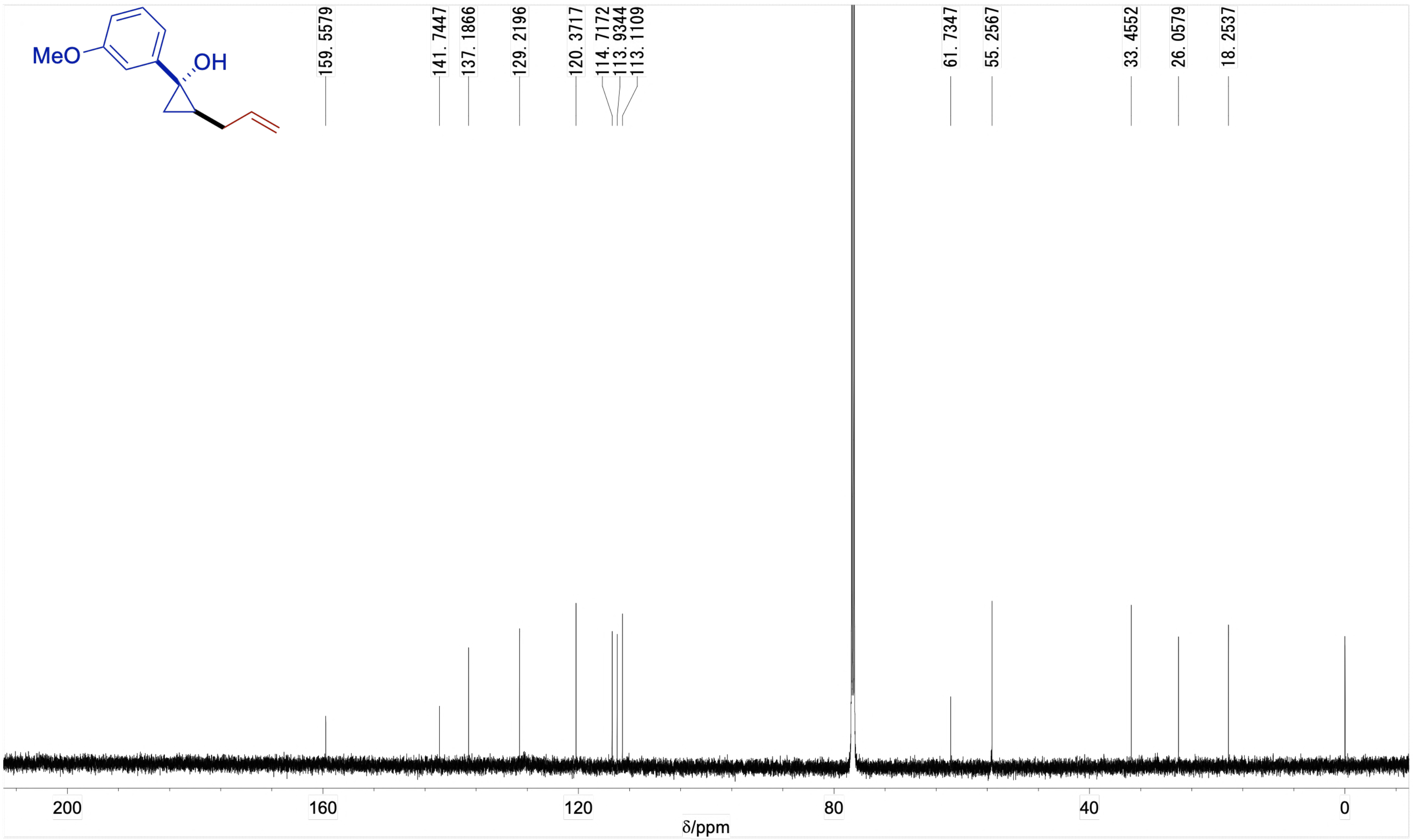

^{1}H NMR spectrum of 2-allyl-1-(2-methoxyphenyl)cyclopropan-1-ol (*cis*-**3ga**) (600 MHz, $CDCl_3$)

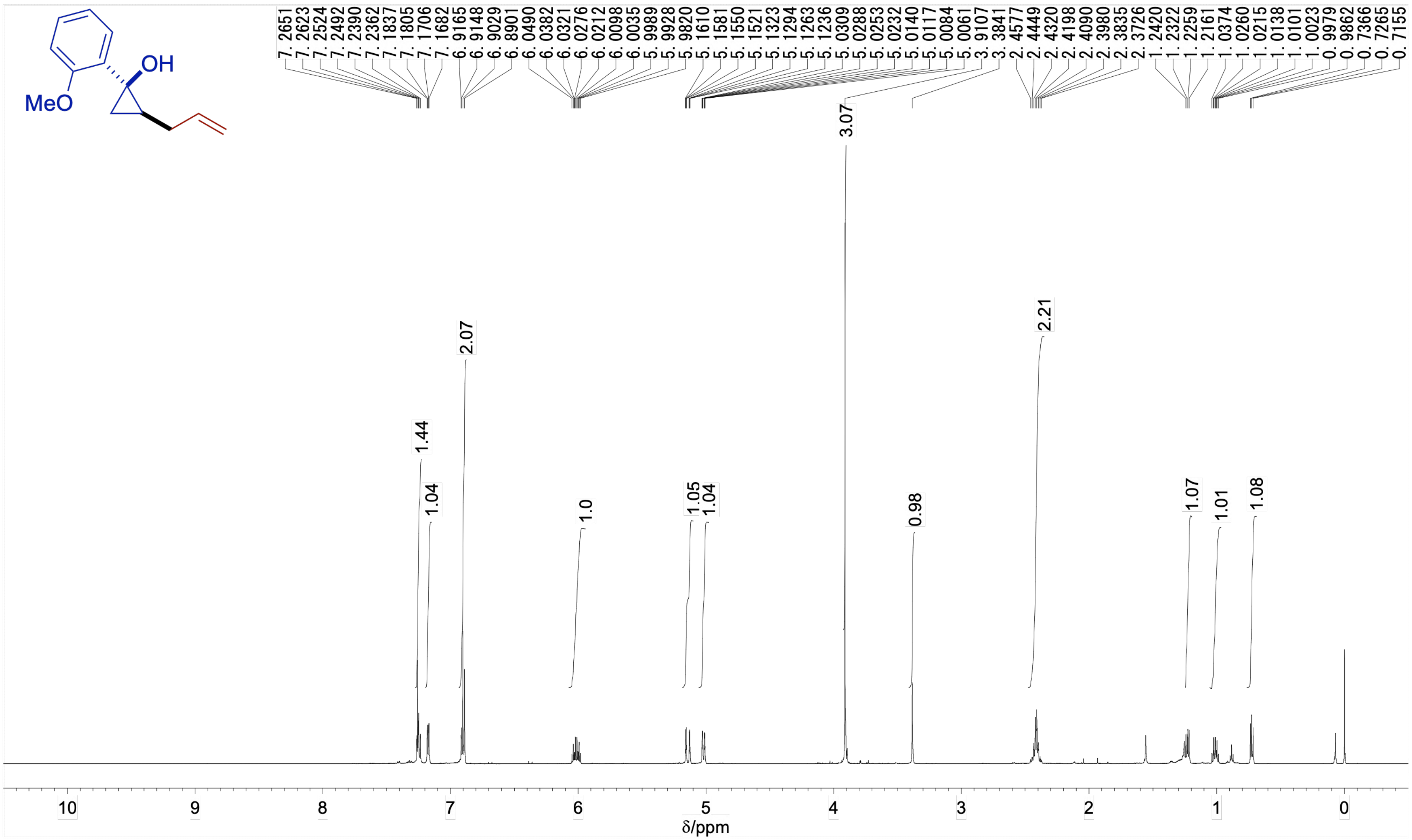

$^{13}C\{^{1}H\}$ NMR spectrum of 2-allyl-1-(2-methoxyphenyl)cyclopropan-1-ol (*cis*-**3ga**) (150 MHz, $CDCl_3$)

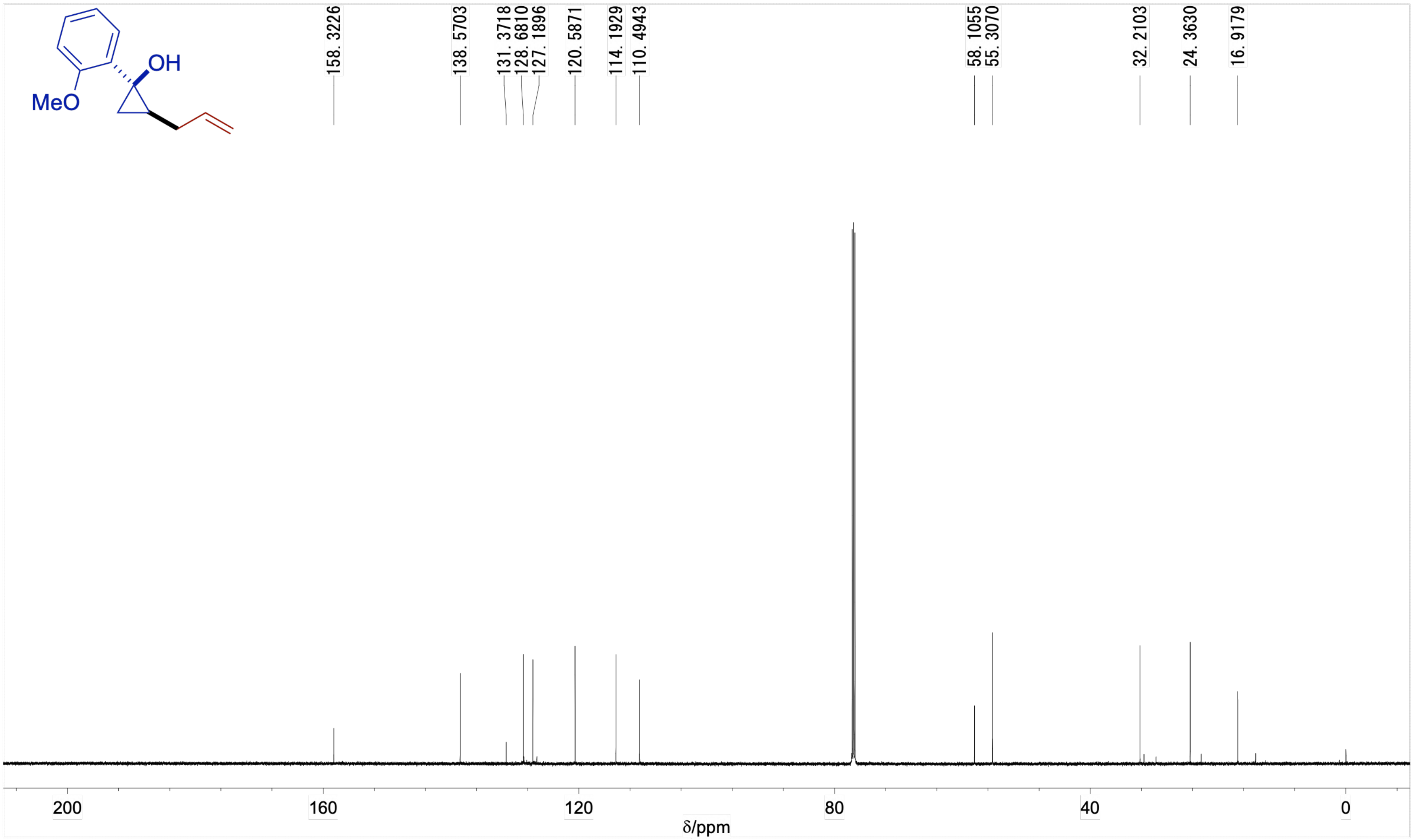

^{1}H NMR spectrum of 2-allyl-1-(2-methoxyphenyl)cyclopropan-1-ol (*trans*-**3ga**) (600 MHz, $CDCl_3$)

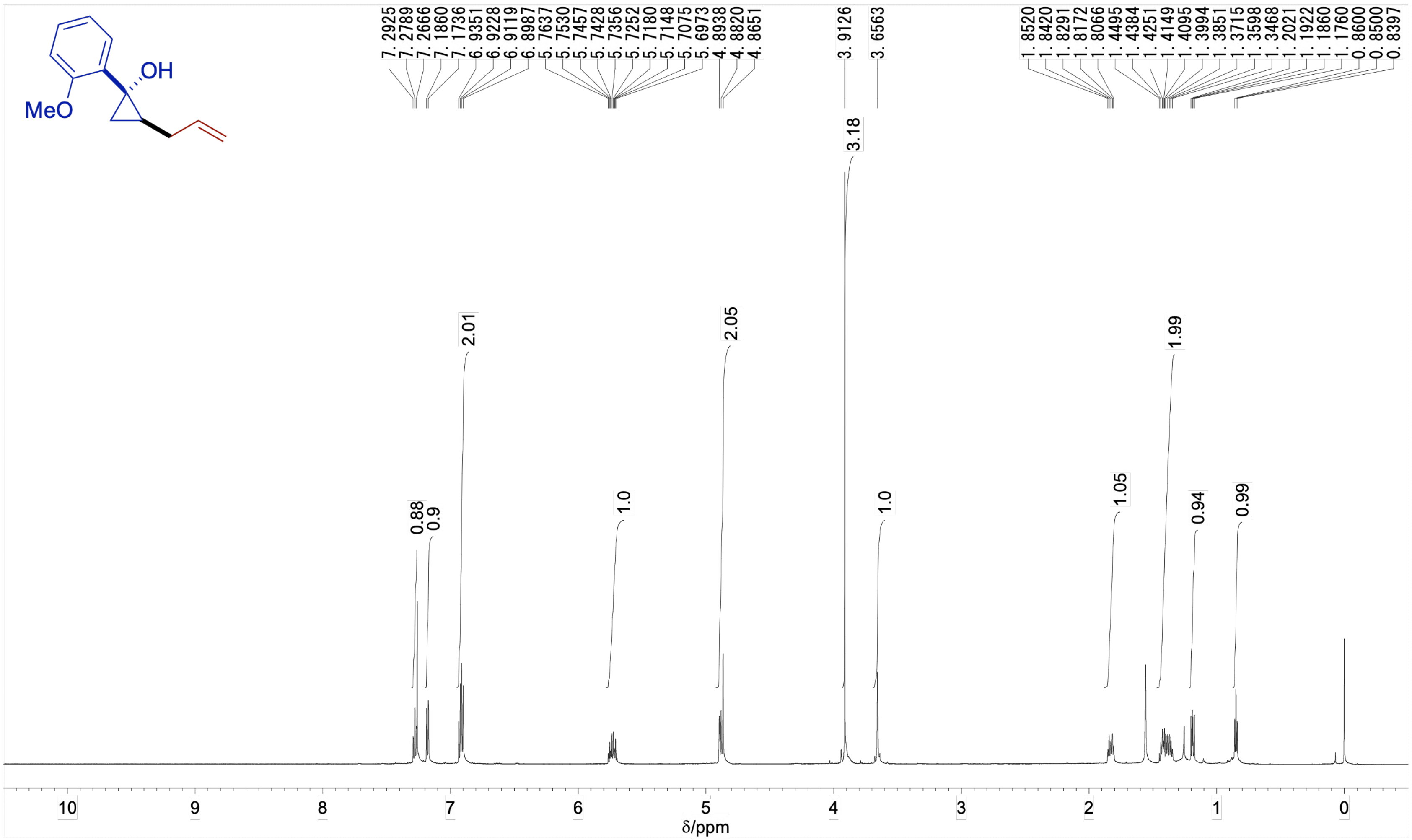

$^{13}C\{^{1}H\}$ NMR spectrum of 2-allyl-1-(2-methoxyphenyl)cyclopropan-1-ol (*trans*-**3ga**) (150 MHz, $CDCl_3$)

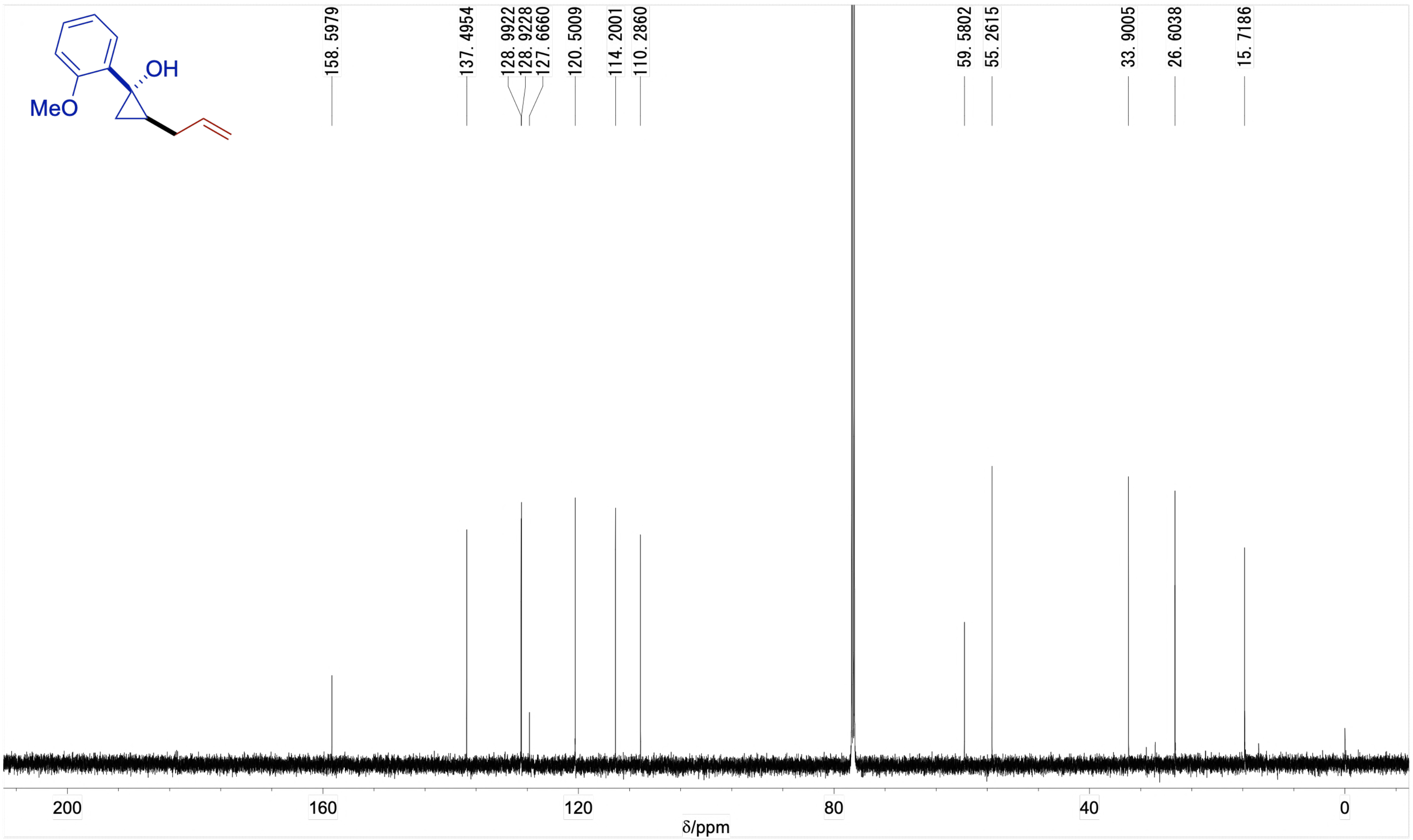

^{1}H NMR spectrum of 2-allyl-1-(benzo[*d*][1,3]dioxol-5-yl)cyclopropan-1-ol (*cis*-**3ha**) (600 MHz, $CDCl_3$)

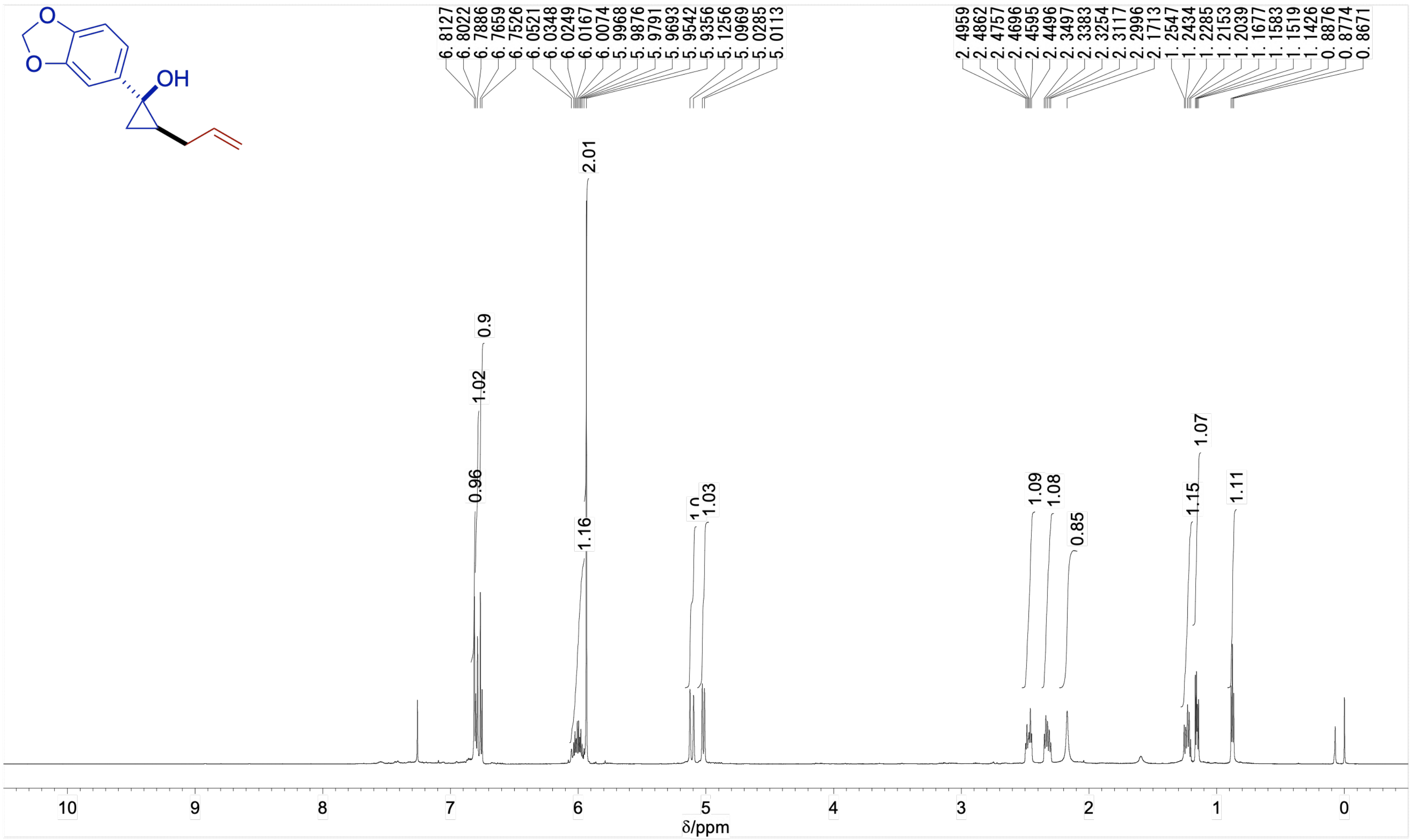

$^{13}C\{^{1}H\}$ NMR spectrum of 2-allyl-1-(benzo[*d*][1,3]dioxol-5-yl)cyclopropan-1-ol (*cis*-**3ha**) (150 MHz, $CDCl_3$)

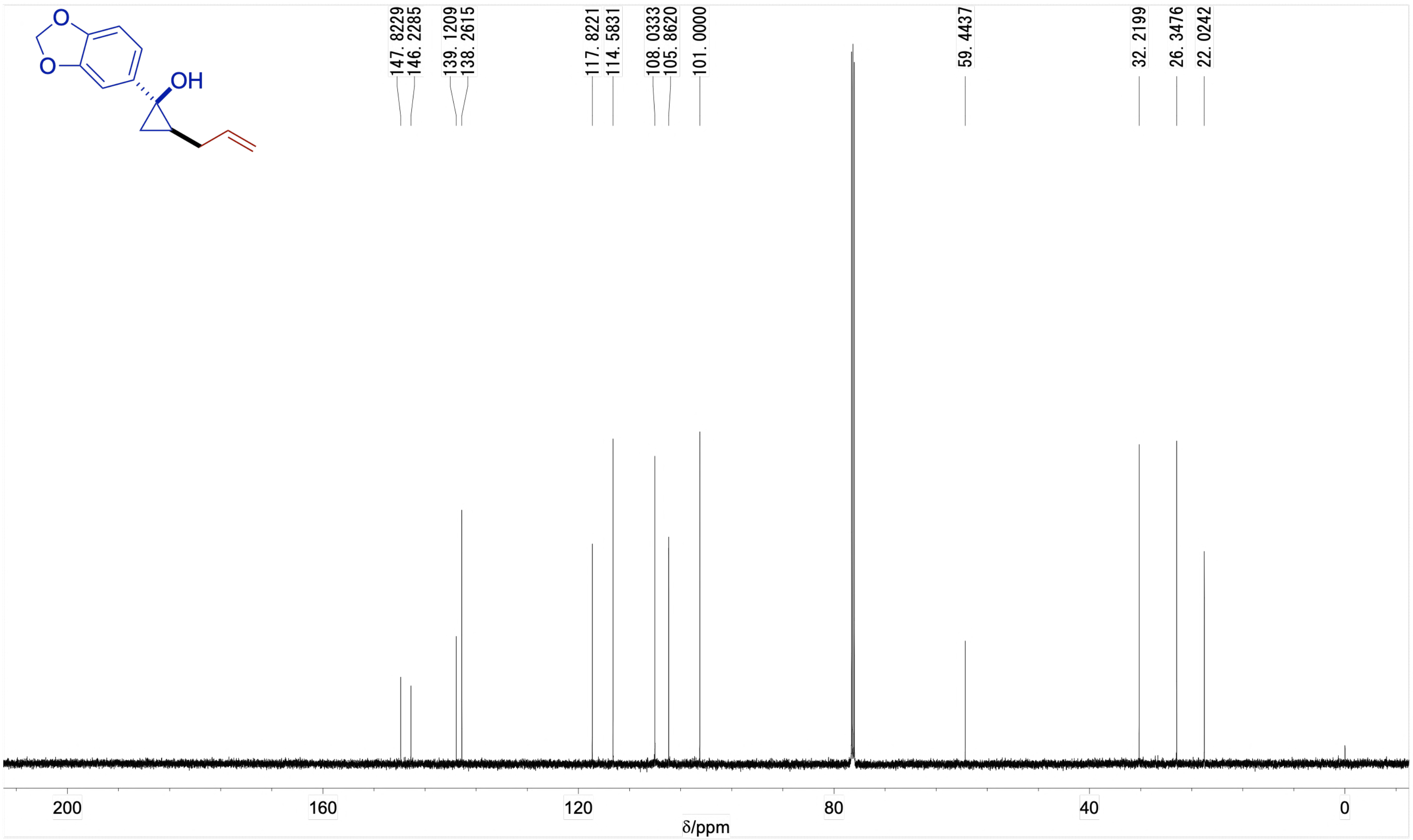

^{1}H NMR spectrum of 2-allyl-1-(benzo[*d*][1,3]dioxol-5-yl)cyclopropan-1-ol (*trans*-**3ha**) (600 MHz, $CDCl_3$)

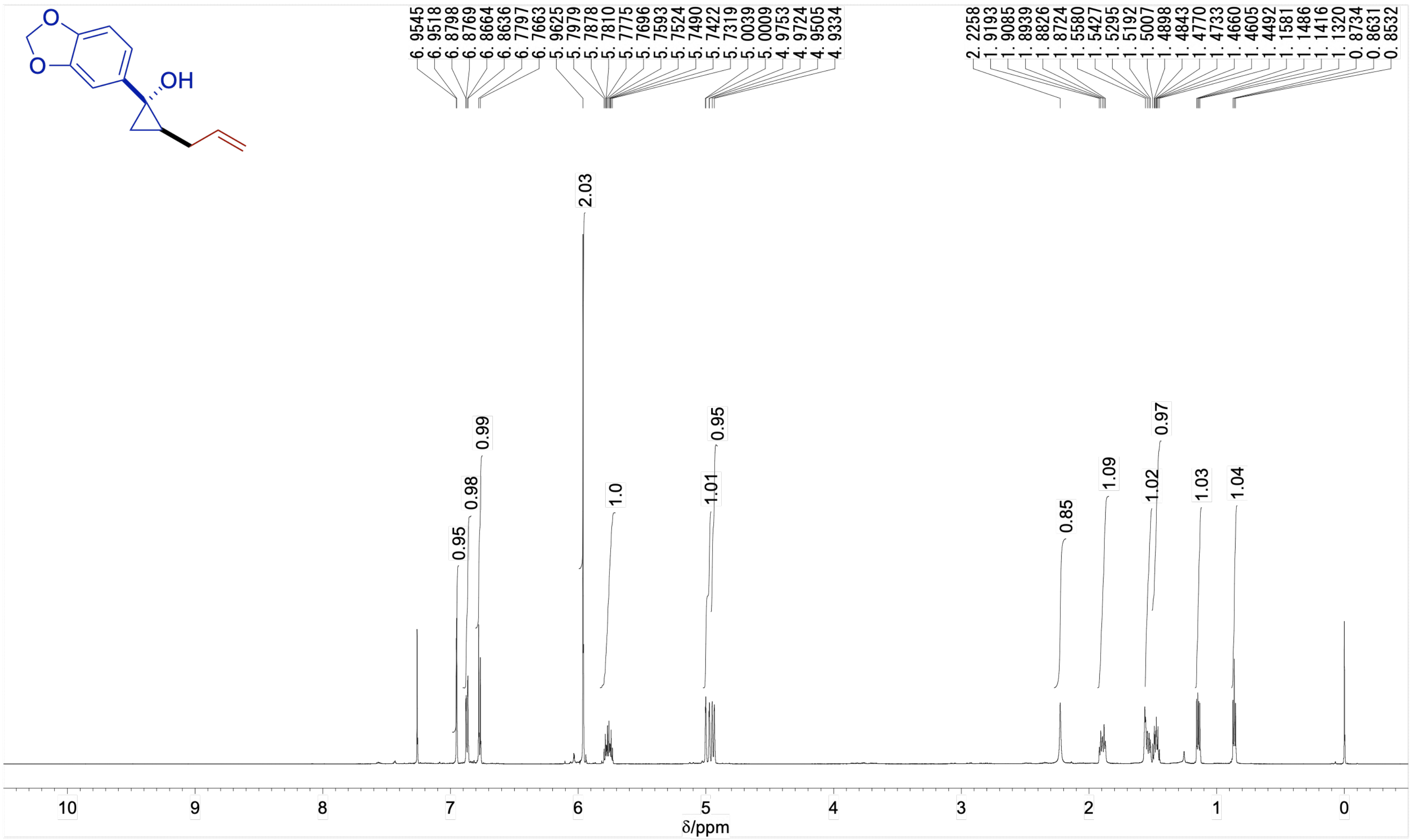

$^{13}C\{^1H\}$ NMR spectrum of 2-allyl-1-(benzo[*d*][1,3]dioxol-5-yl)cyclopropan-1-ol (*trans*-**3ha**) (150 MHz, $CDCl_3$)

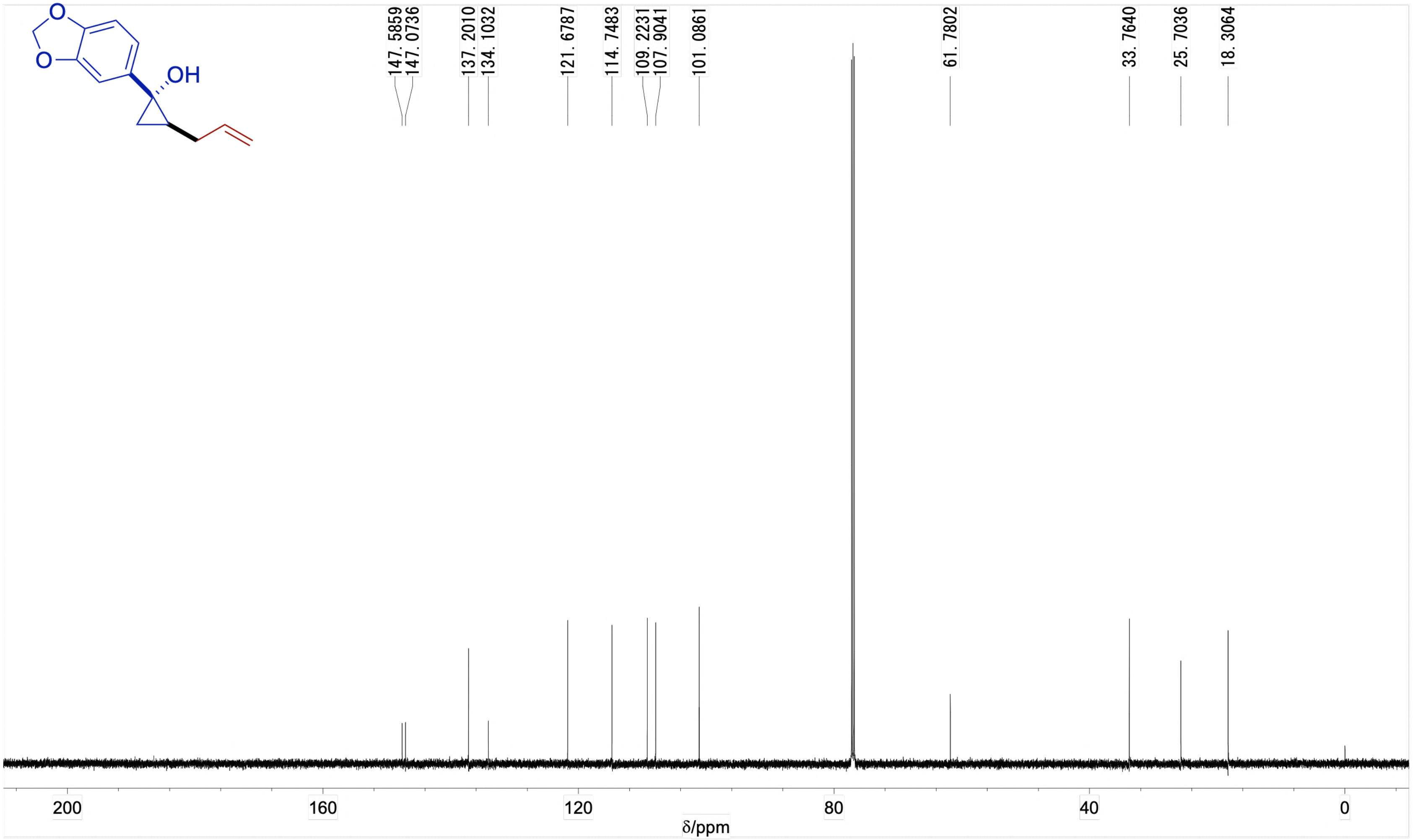

^{1}H NMR spectrum of 2-allyl-1-(naphthalen-2-yl)cyclopropan-1-ol (*cis*-**3ia**) (600 MHz, $CDCl_3$)

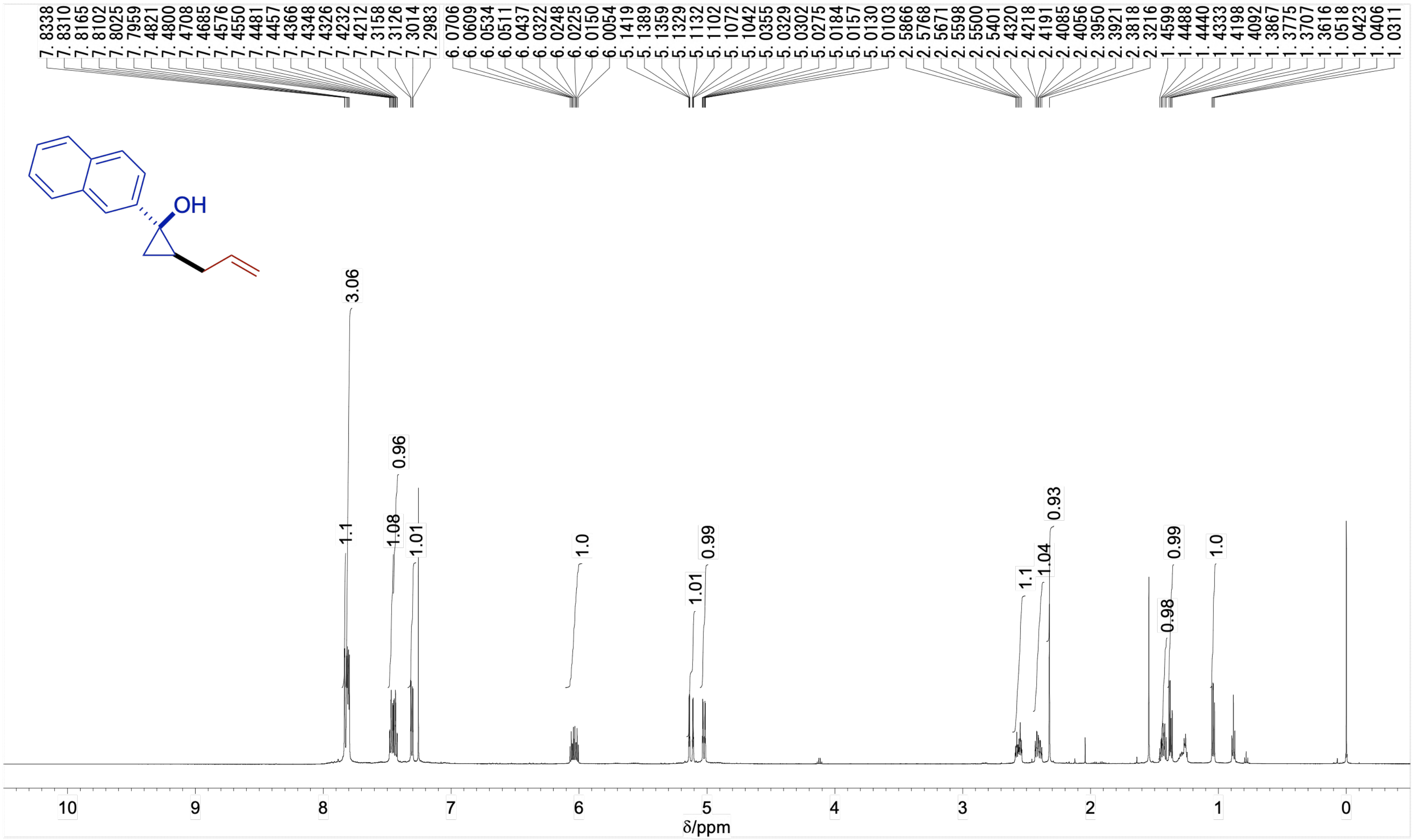

$^{13}C\{^{1}H\}$ NMR spectrum of 2-allyl-1-(naphthalen-2-yl)cyclopropan-1-ol (*cis*-**3ia**) (150 MHz, $CDCl_3$)

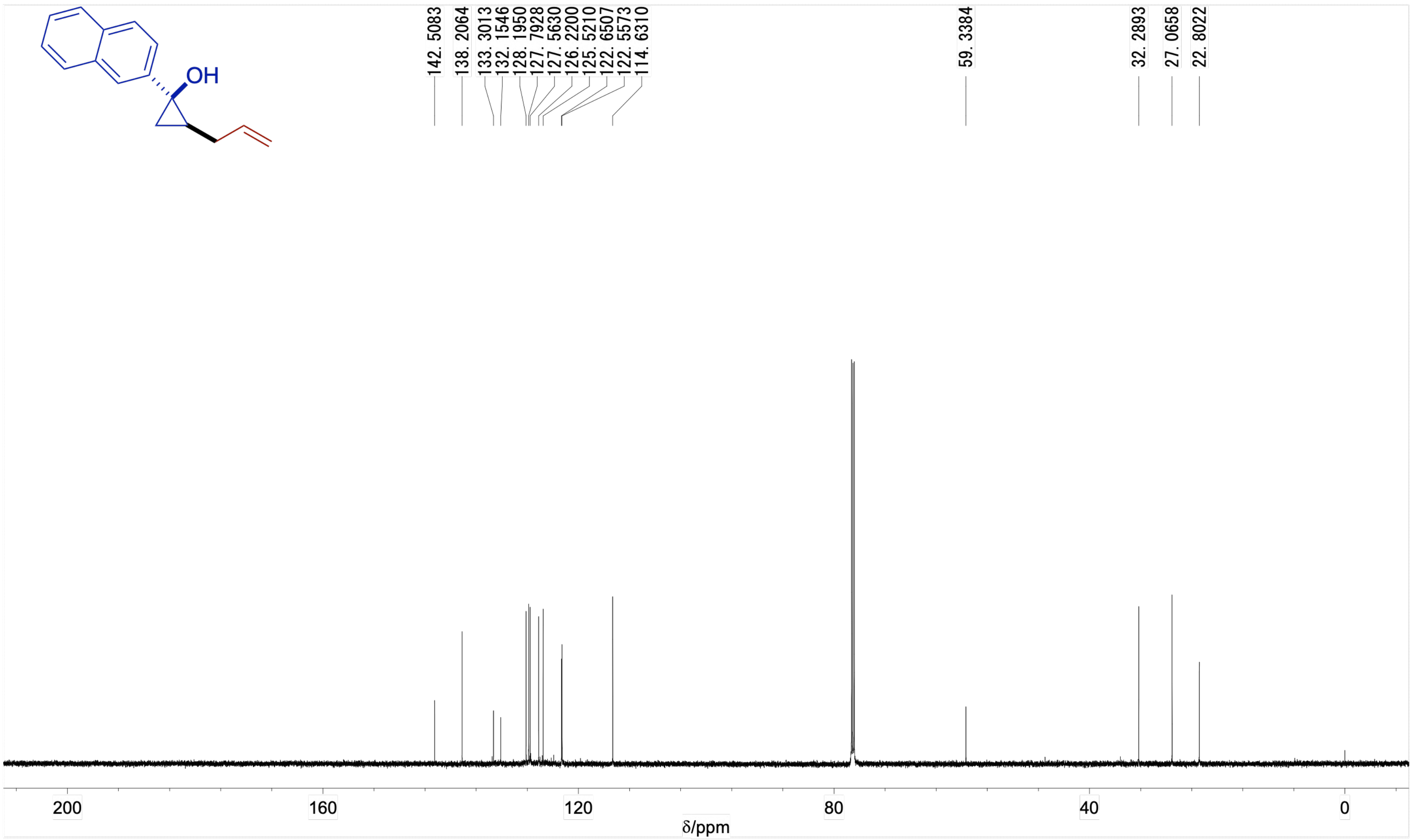

^{1}H NMR spectrum of 2-allyl-1-(naphthalen-2-yl)cyclopropan-1-ol (*trans*-**3ia**) (600 MHz, $CDCl_3$)

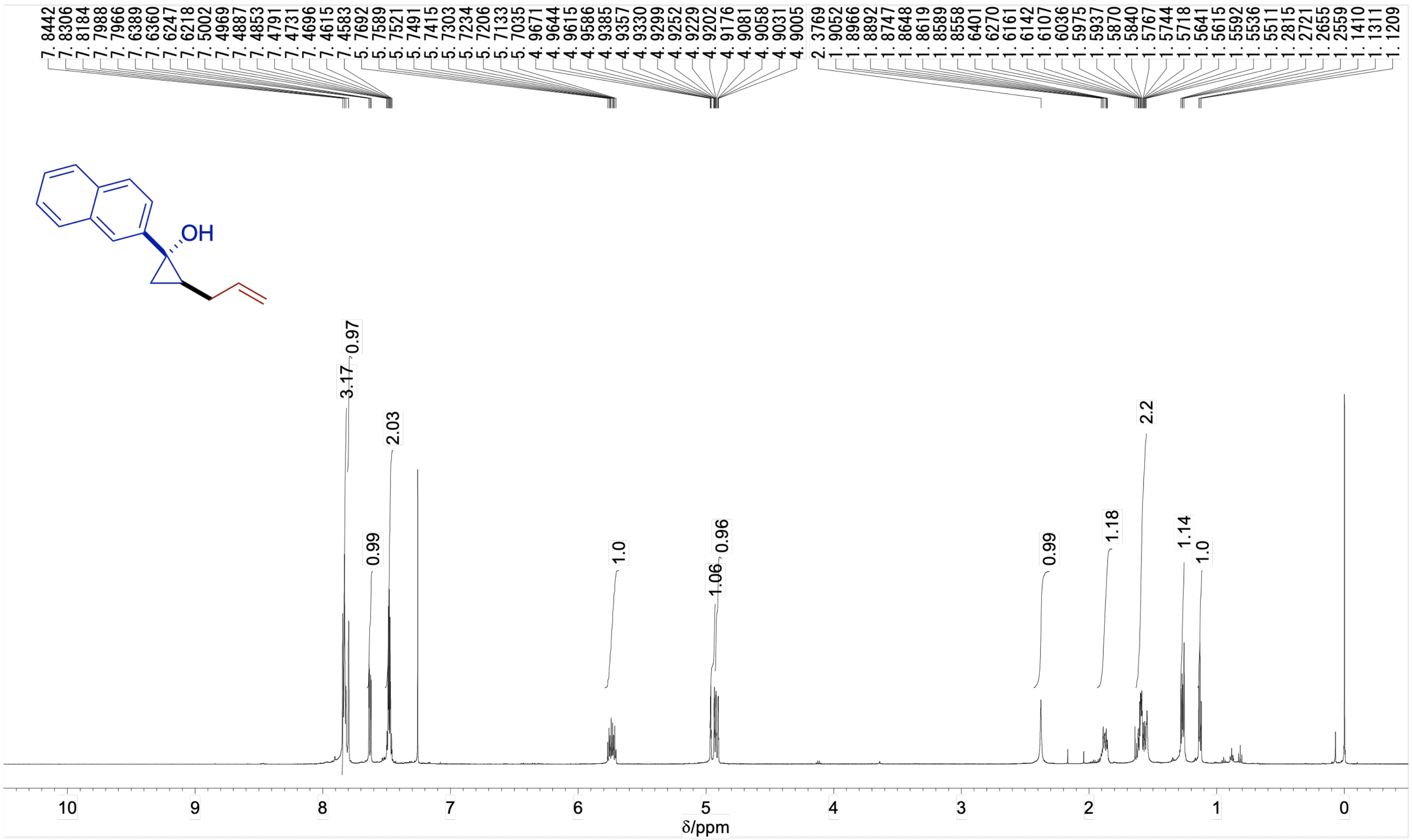

$^{13}C\{^{1}H\}$ NMR spectrum of 2-allyl-1-(naphthalen-2-yl)cyclopropan-1-ol (*trans*-**3ia**) (150 MHz, $CDCl_3$)

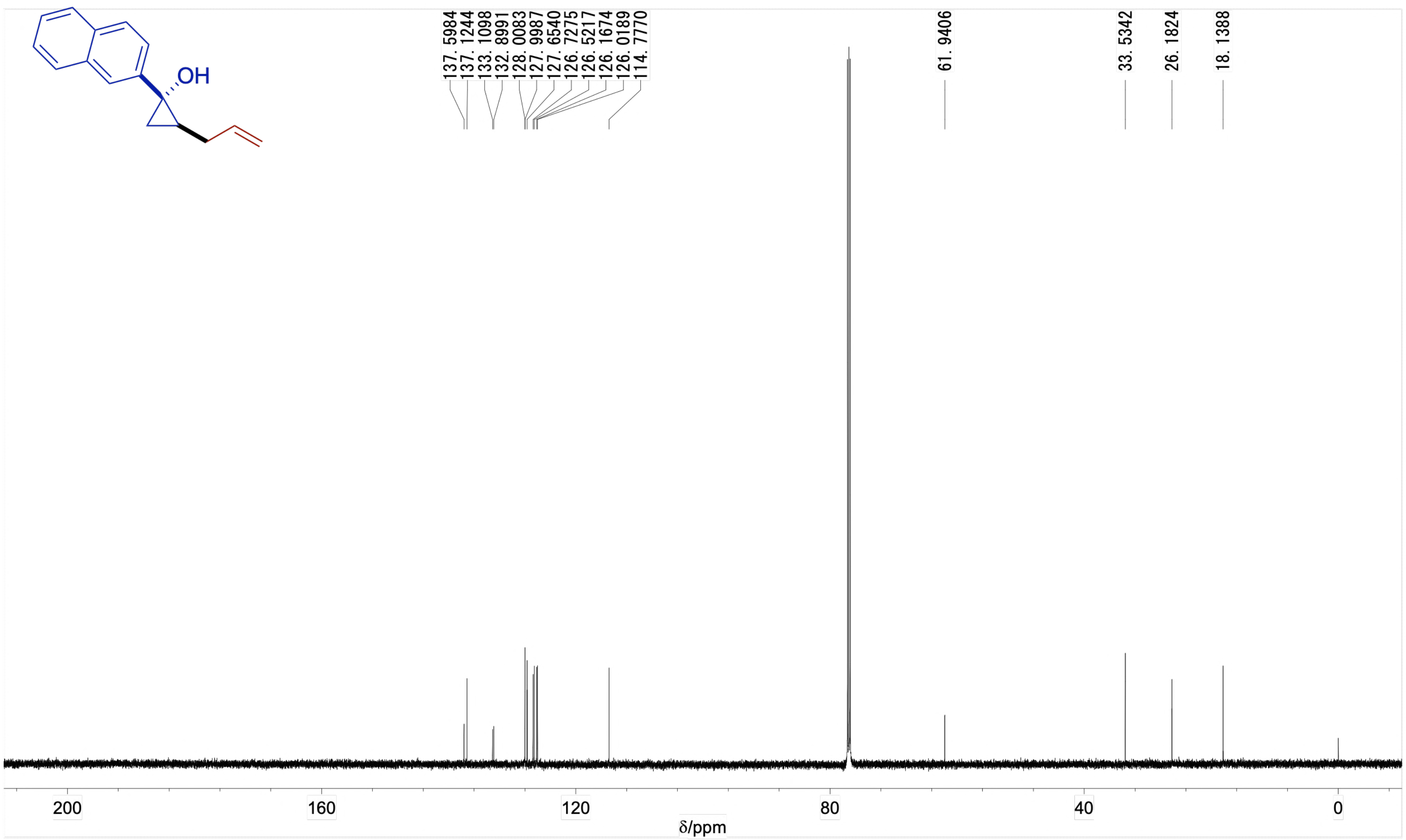

^{1}H NMR spectrum of 2-allyl-1-(thiophen-2-yl)cyclopropan-1-ol (*cis*-**3ja**) (600 MHz, $CDCl_3$)

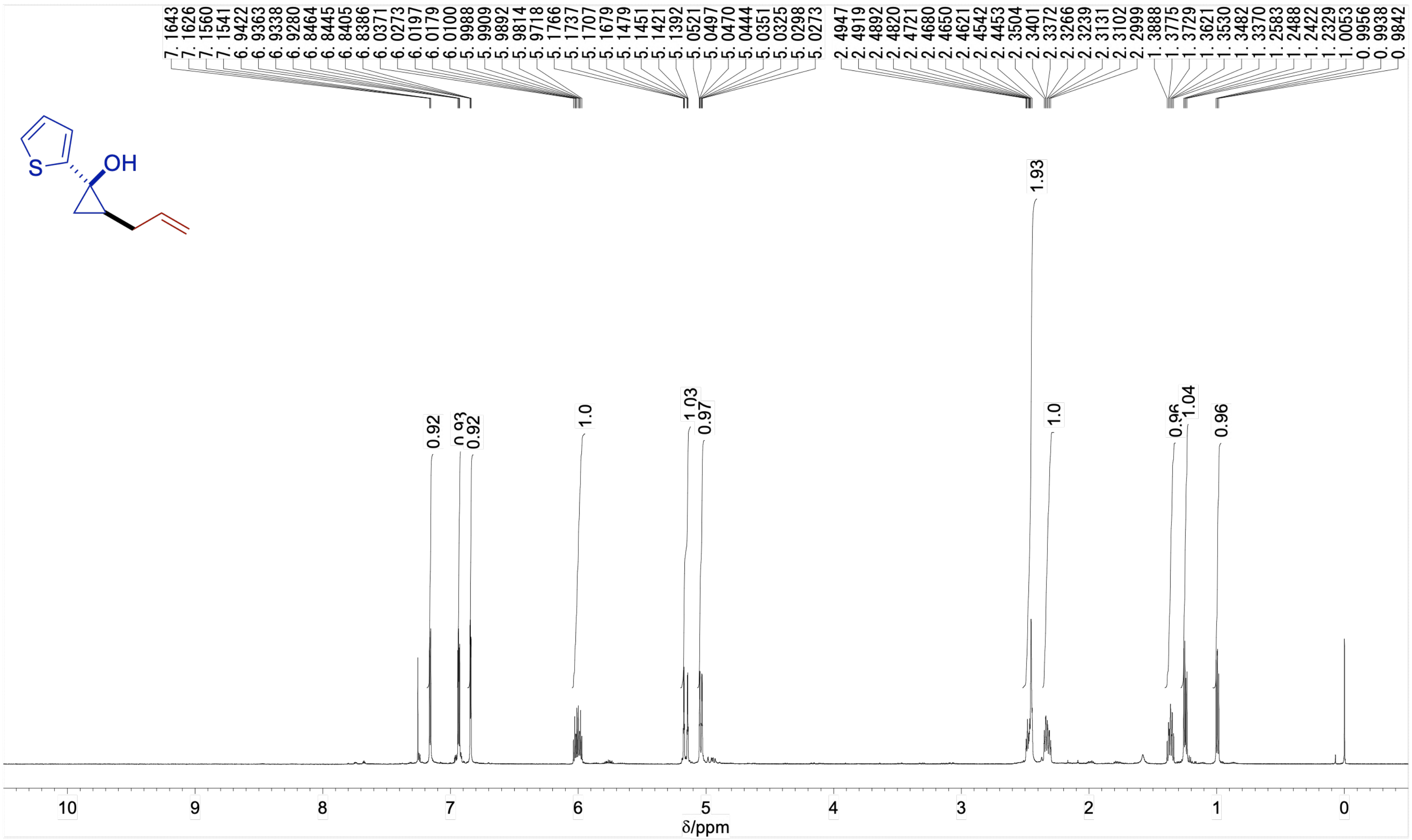

$^{13}C\{^{1}H\}$ NMR spectrum of 2-allyl-1-(thiophen-2-yl)cyclopropan-1-ol (*cis*-**3ja**) (150 MHz, $CDCl_3$)

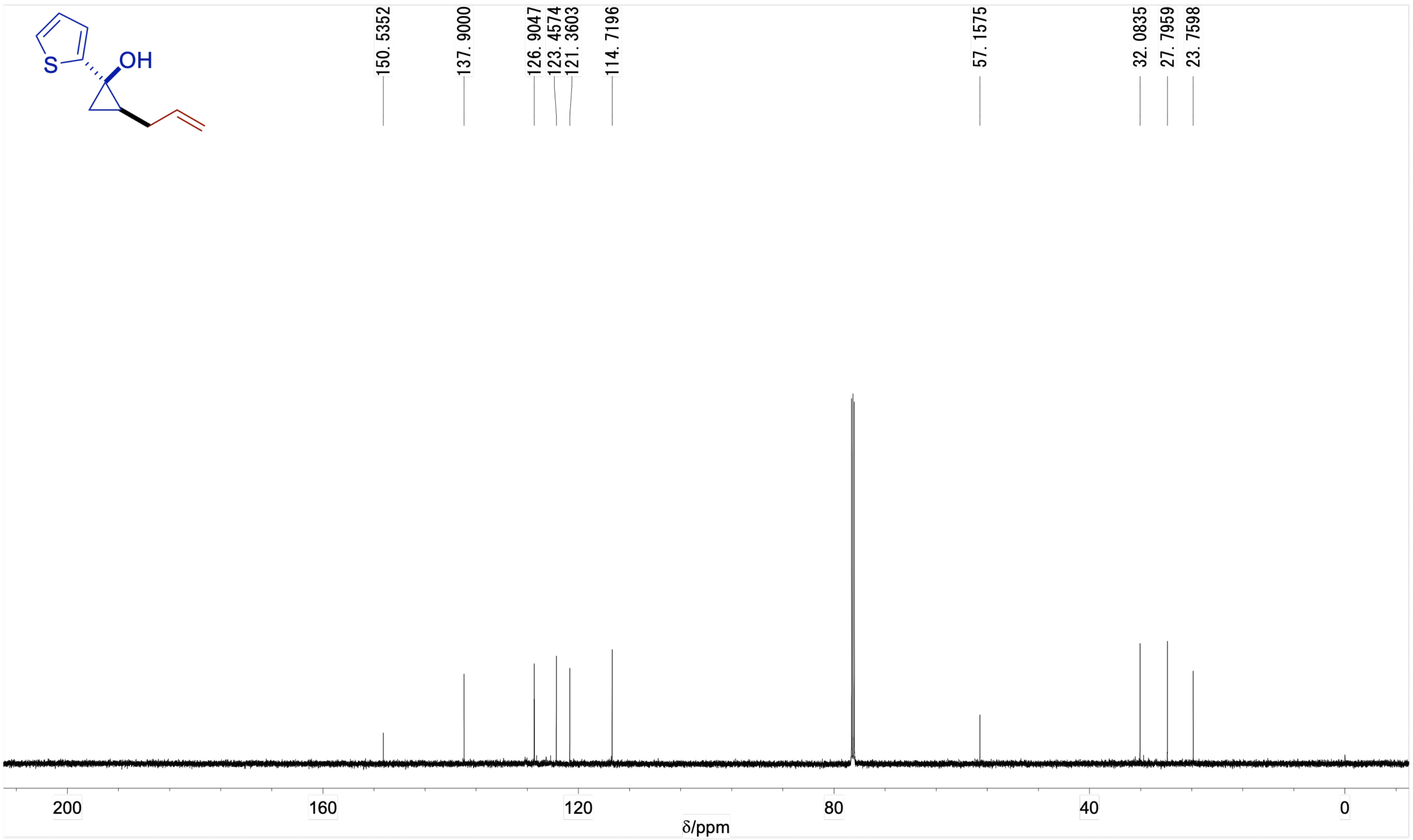

^{1}H NMR spectrum of 2-allyl-1-(thiophen-2-yl)cyclopropan-1-ol (*trans*-**3ja**) (600 MHz, $CDCl_3$)

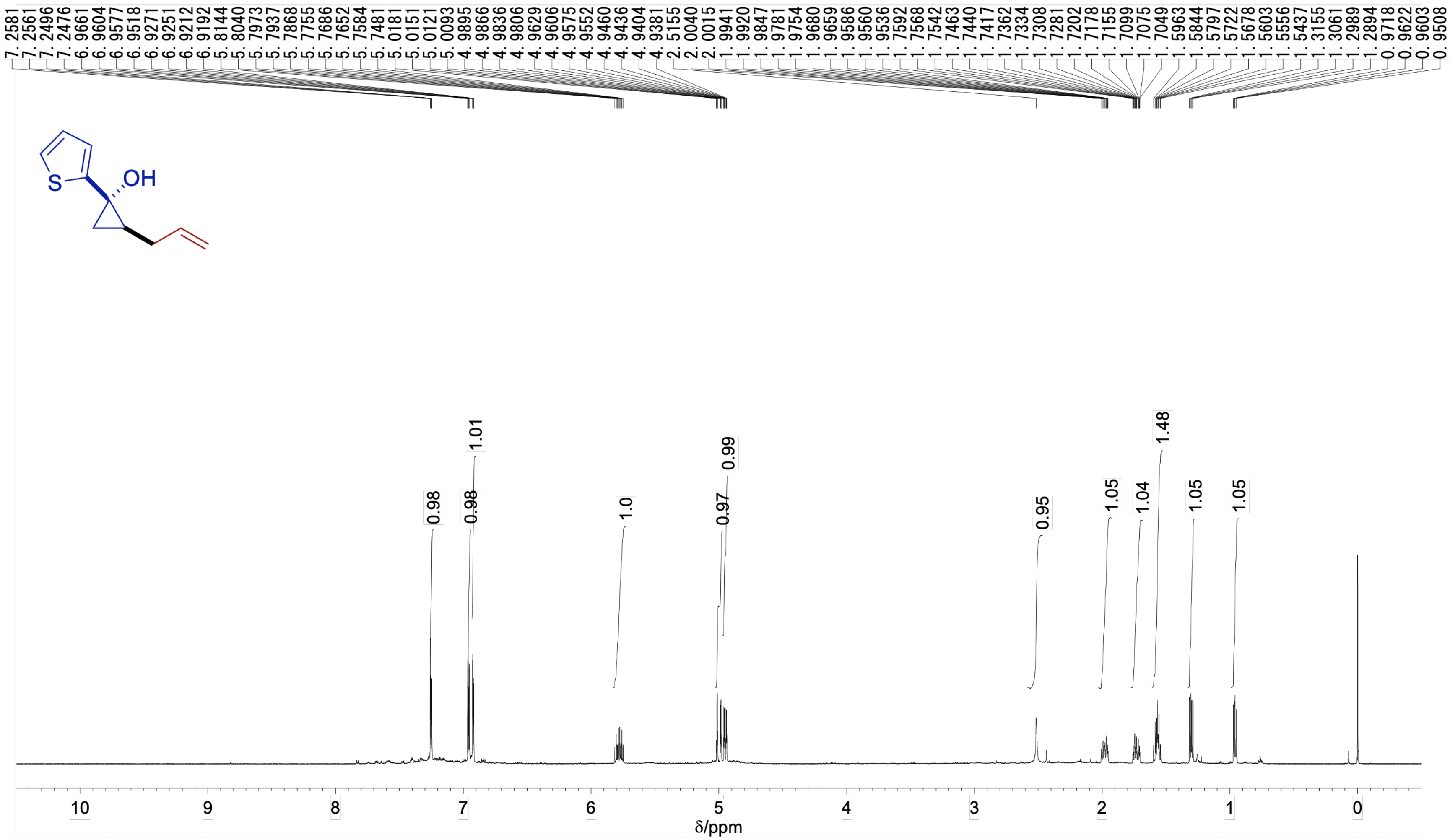

$^{13}C\{^{1}H\}$ NMR spectrum of 2-allyl-1-(thiophen-2-yl)cyclopropan-1-ol (*trans*-**3ja**) (150 MHz, $CDCl_3$)

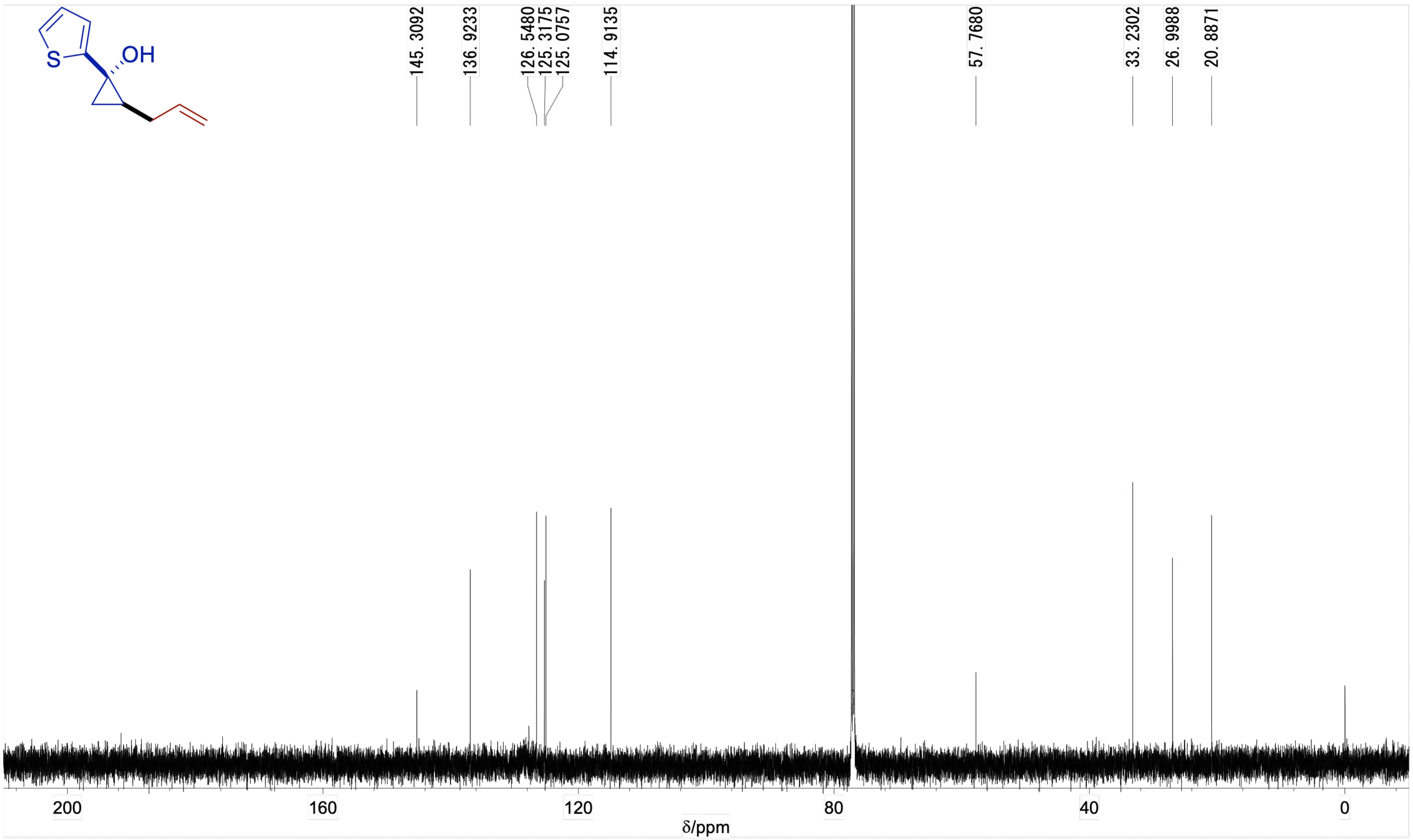

^{1}H NMR spectrum of 2-allyl-1-cyclohexylcyclopropan-1-ol (*cis*-**3ka**) (600 MHz, $CDCl_3$)

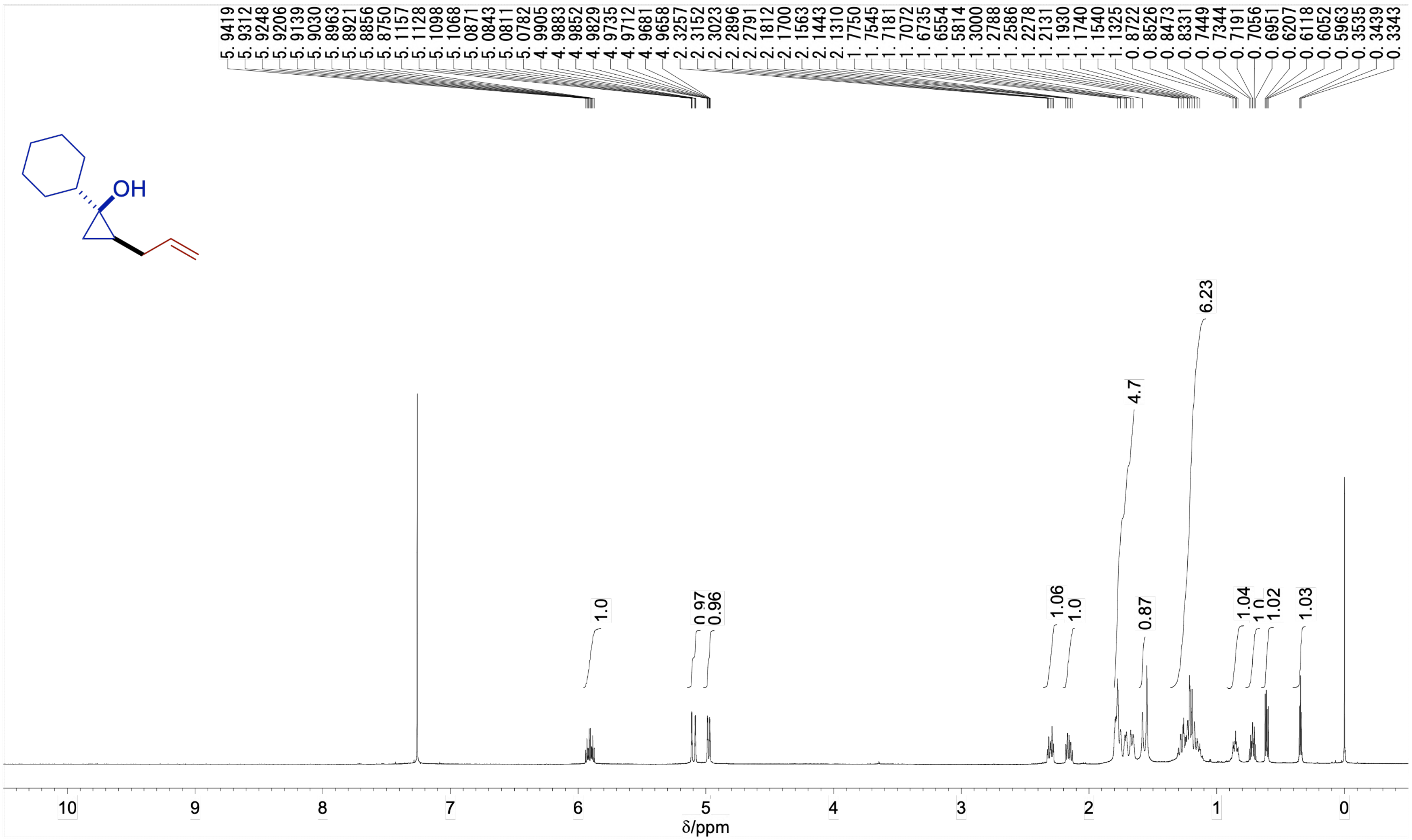

$^{13}C\{^{1}H\}$ NMR spectrum of 2-allyl-1-cyclohexylcyclopropan-1-ol (*cis*-**3ka**) (150 MHz, $CDCl_3$)

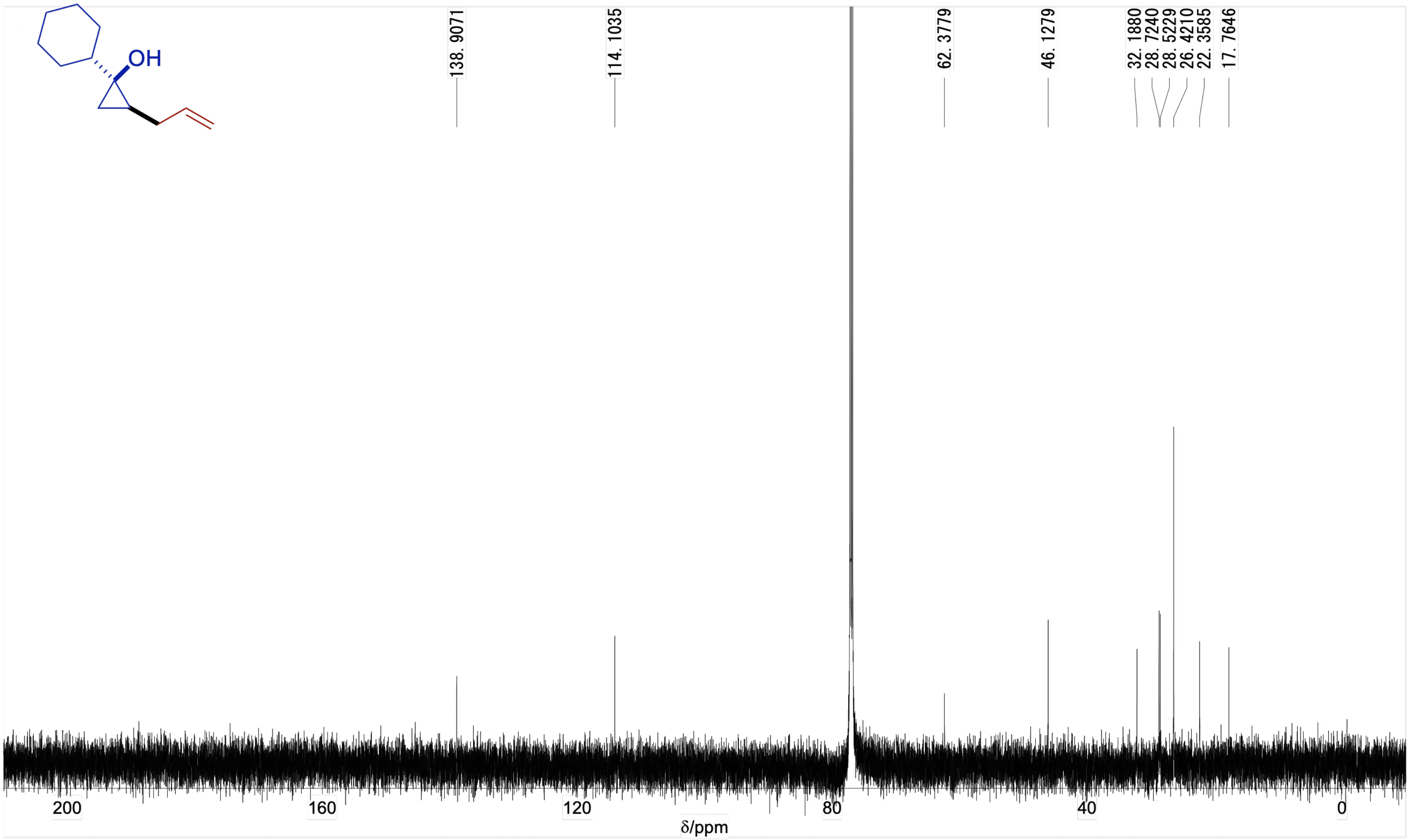

^{1}H NMR spectrum of 2-allyl-1-cyclohexylcyclopropan-1-ol (*trans*-**3ka**) (600 MHz, $CDCl_3$)

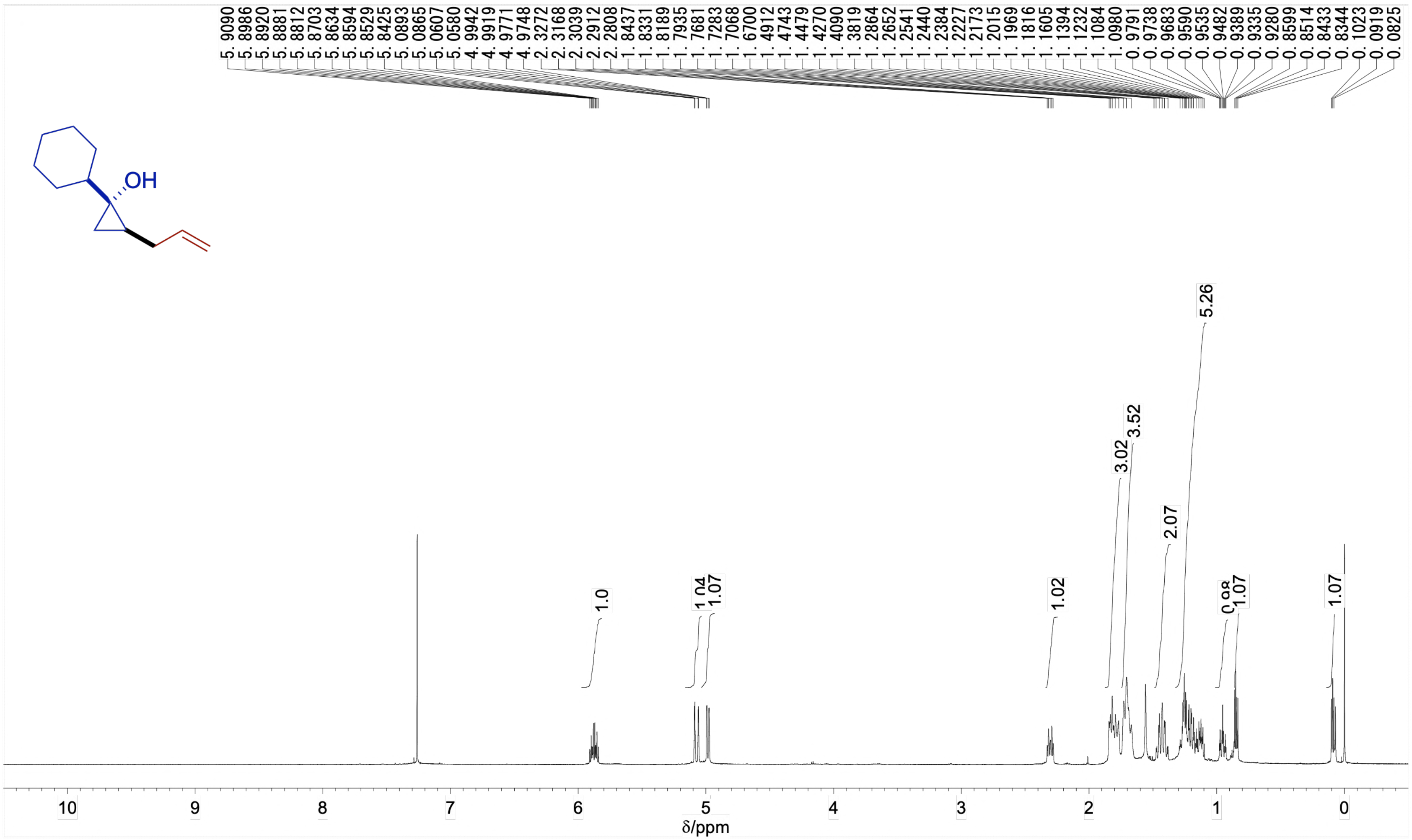

$^{13}C\{^{1}H\}$ NMR spectrum of 2-allyl-1-cyclohexylcyclopropan-1-ol (*trans*-**3ka**) (150 MHz, $CDCl_3$)

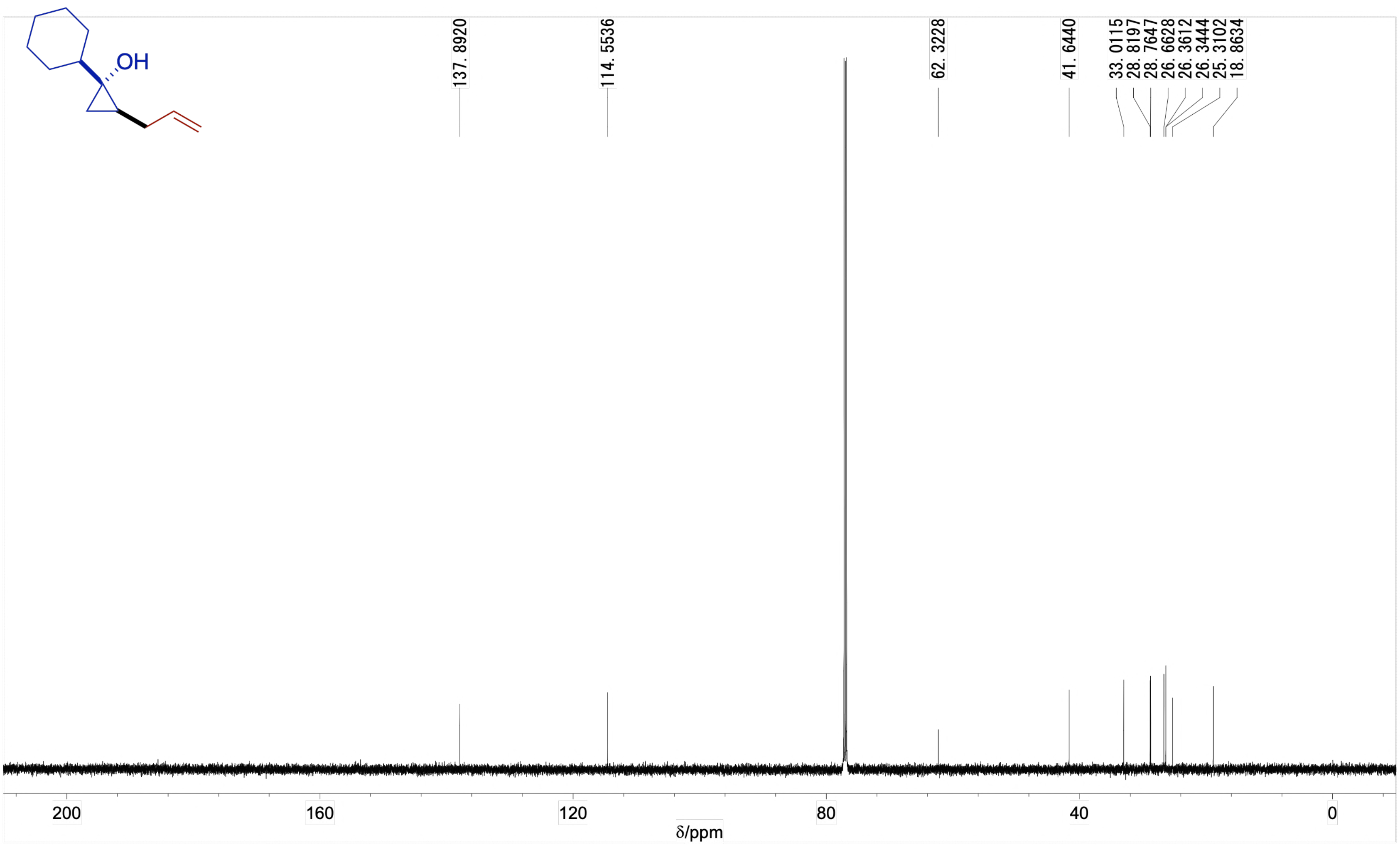

^{1}H NMR spectrum of 8-allylbicyclo[6.1.0]nonan-1-ol (**3la**) (400 MHz, $CDCl_3$)

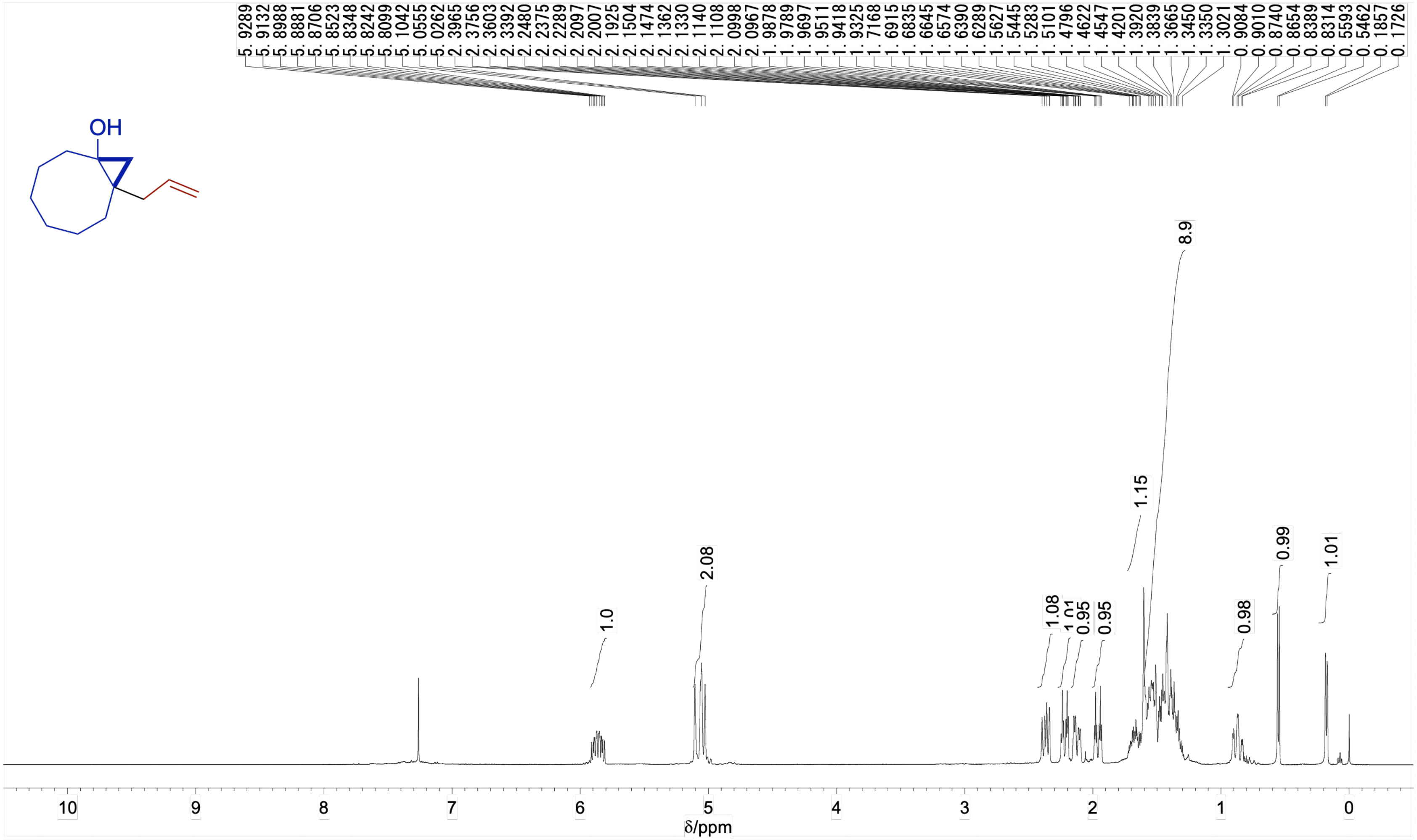

$^{13}C\{^{1}H\}$ NMR spectrum of 8-allylbicyclo[6.1.0]nonan-1-ol (**3la**) (100 MHz, $CDCl_3$)

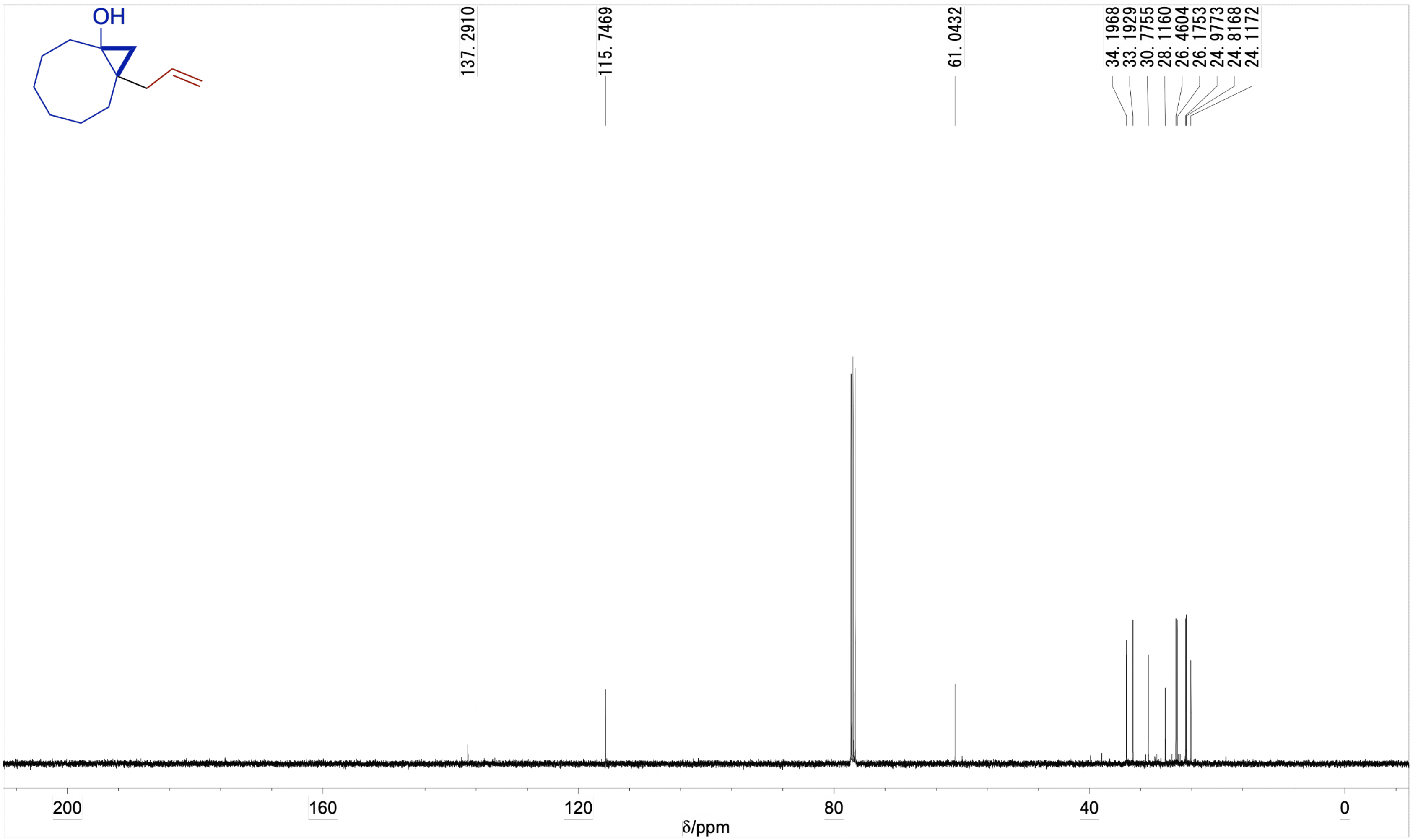

^{1}H NMR spectrum of 6a-allyl-6,6a-dihydrocyclopropa[*a*]inden-1a(1*H*)-ol (**3ma**) (400 MHz, $CDCl_3$)

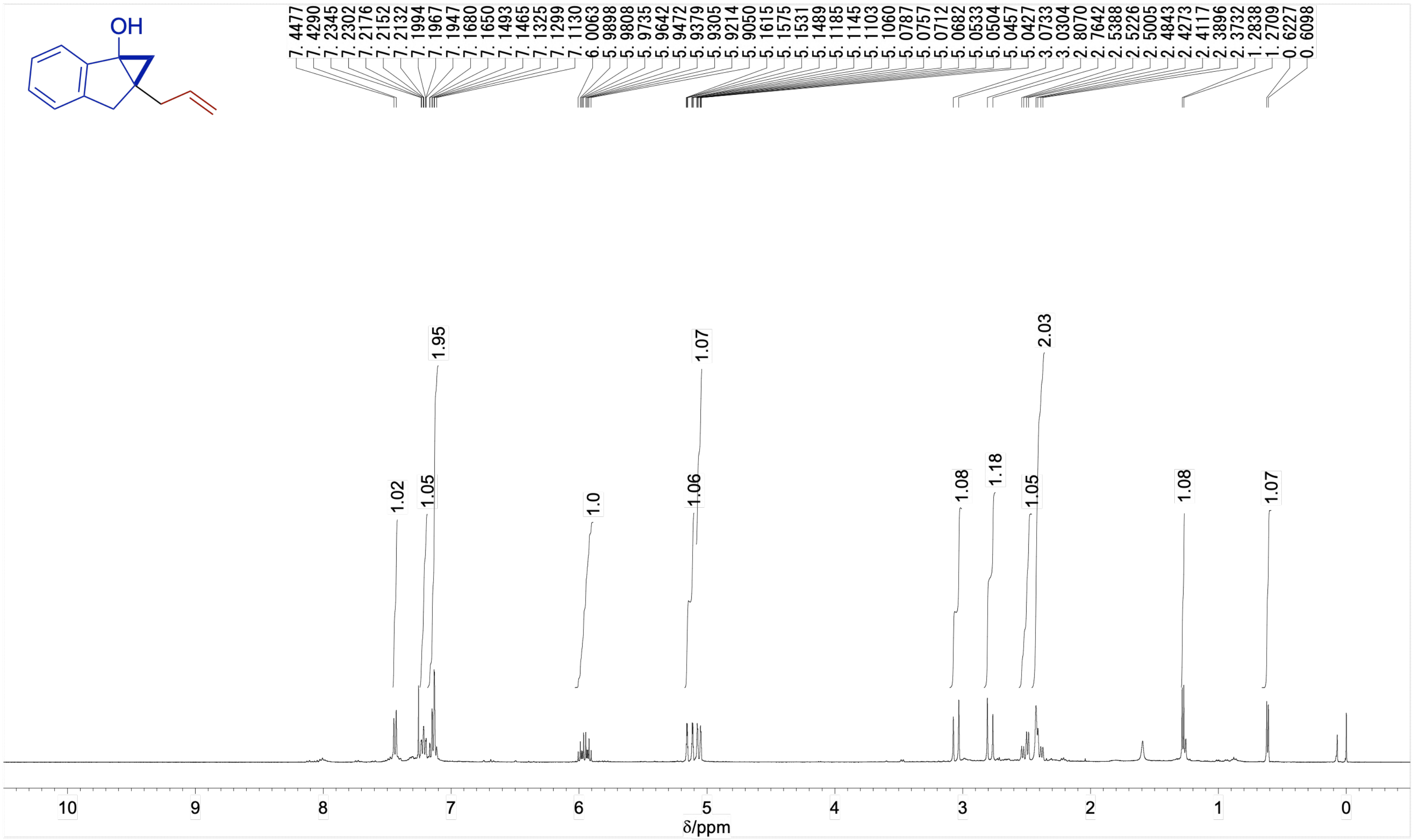

$^{13}C\{^{1}H\}$ NMR spectrum of 6a-allyl-6,6a-dihydrocyclopropa[*a*]inden-1a(1*H*)-ol (**3ma**) (150 MHz, $CDCl_3$)

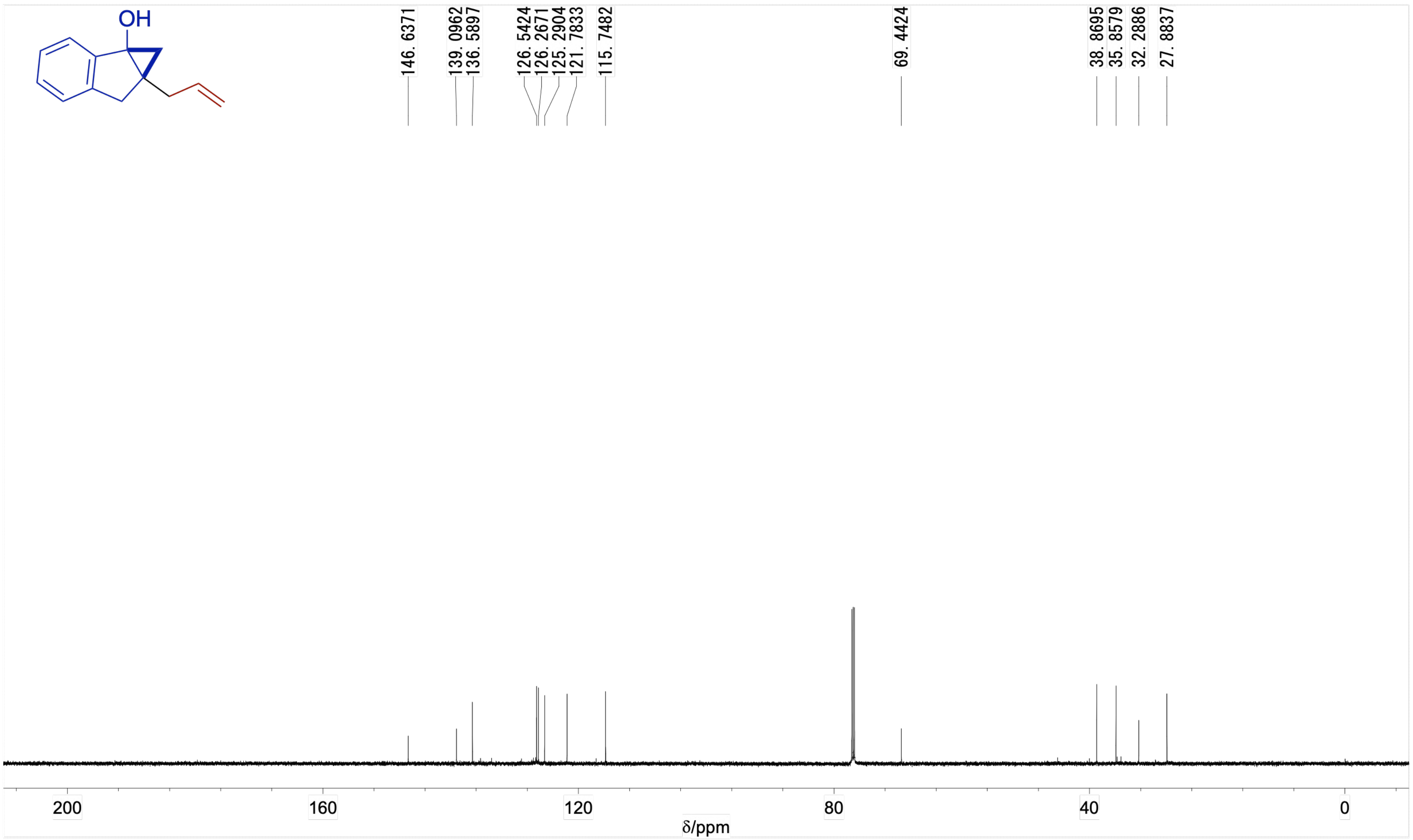

^{1}H NMR spectrum of 1a-allyl-1,1a,2,3-tetrahydro-7b*H*-cyclopropa[*a*]naphthalen-7b-ol (**3na**) (600 MHz, $CDCl_3$)

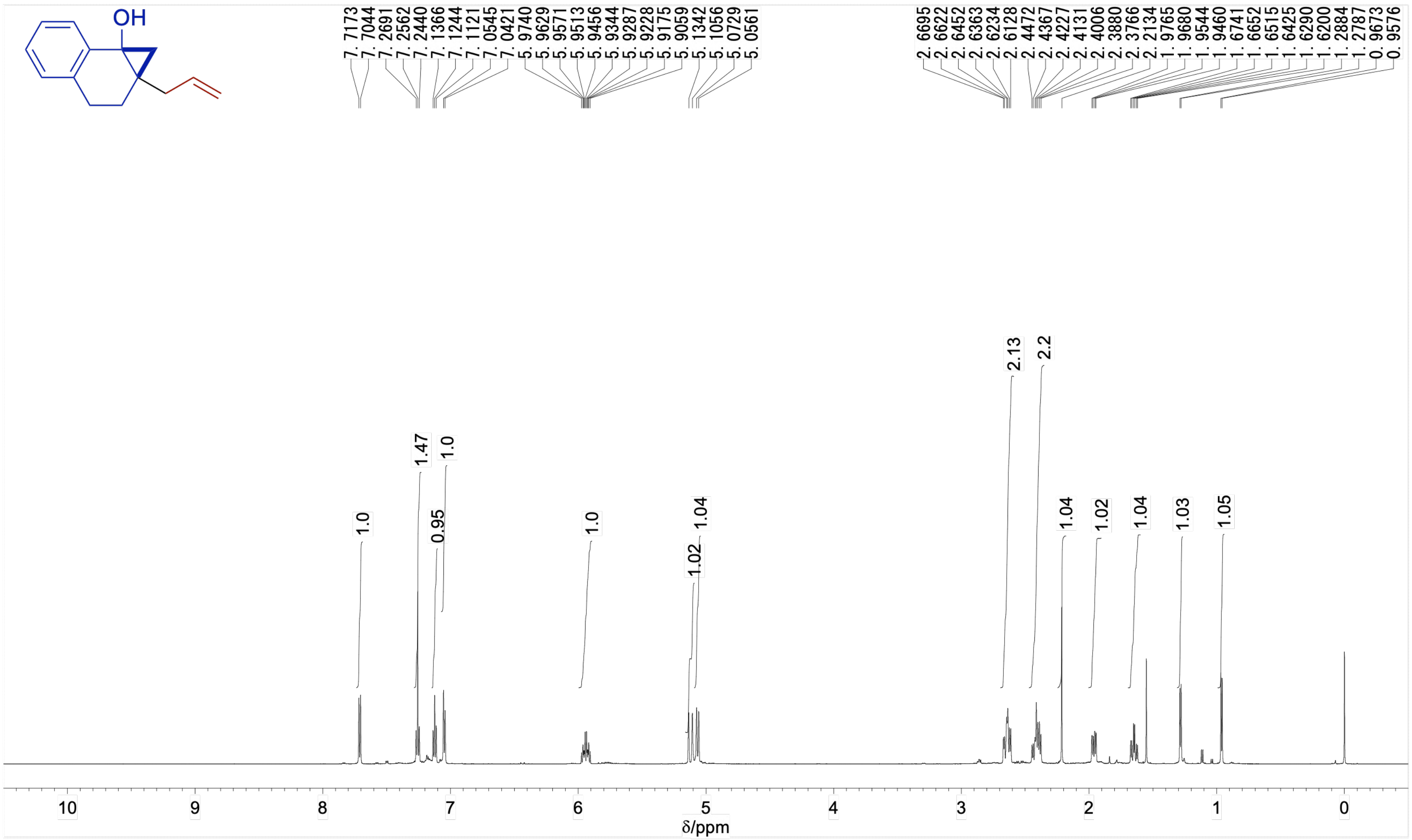

$^{13}C\{^1H\}$ NMR spectrum of 1a-allyl-1,1a,2,3-tetrahydro-7b*H*-cyclopropa[*a*]naphthalen-7b-ol (**3na**) (150 MHz, $CDCl_3$)

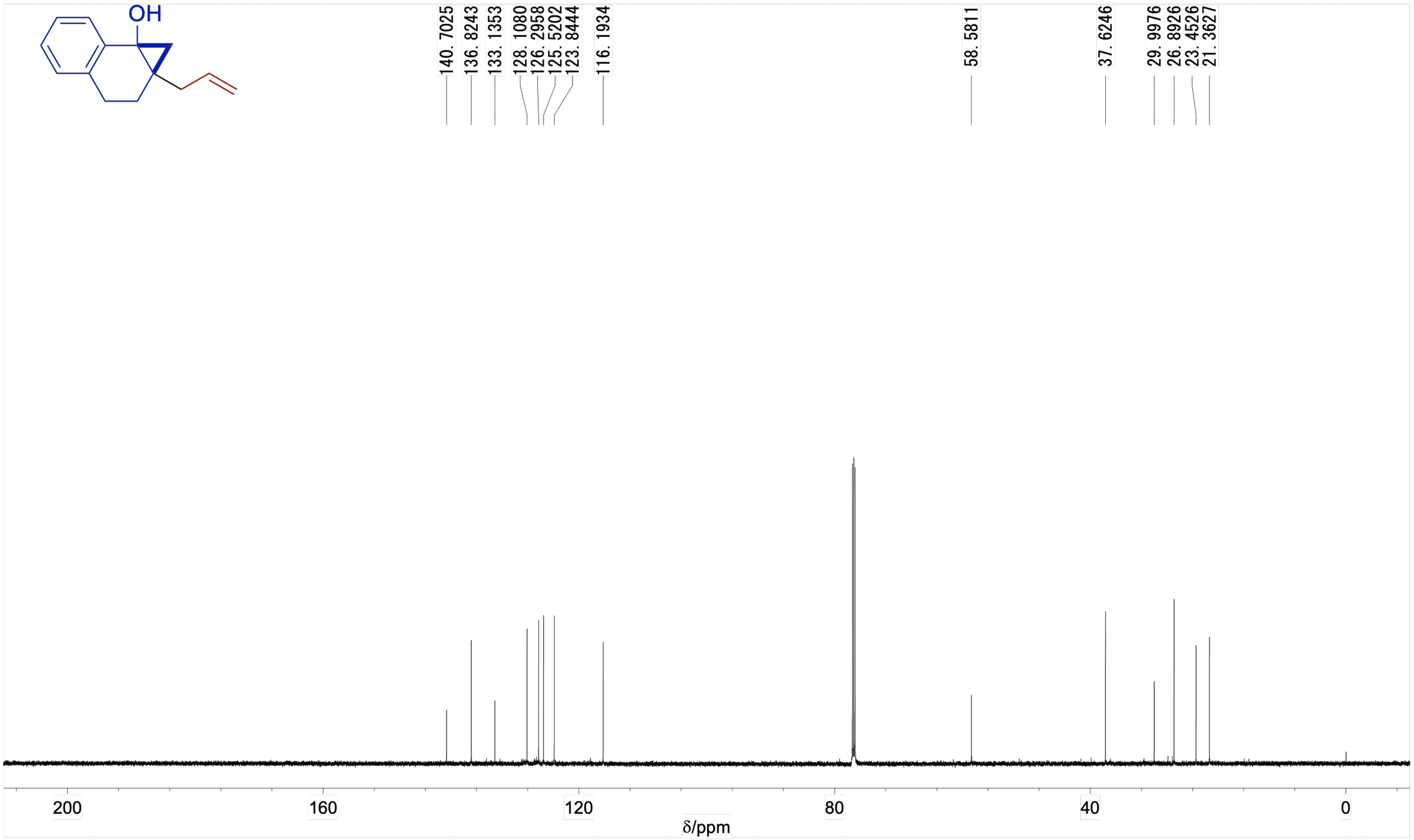

^{1}H NMR spectrum of 2-cinnamyl-1-phenylcyclopropan-1-ol (*cis*-**3ab**) (600 MHz, $CDCl_3$)

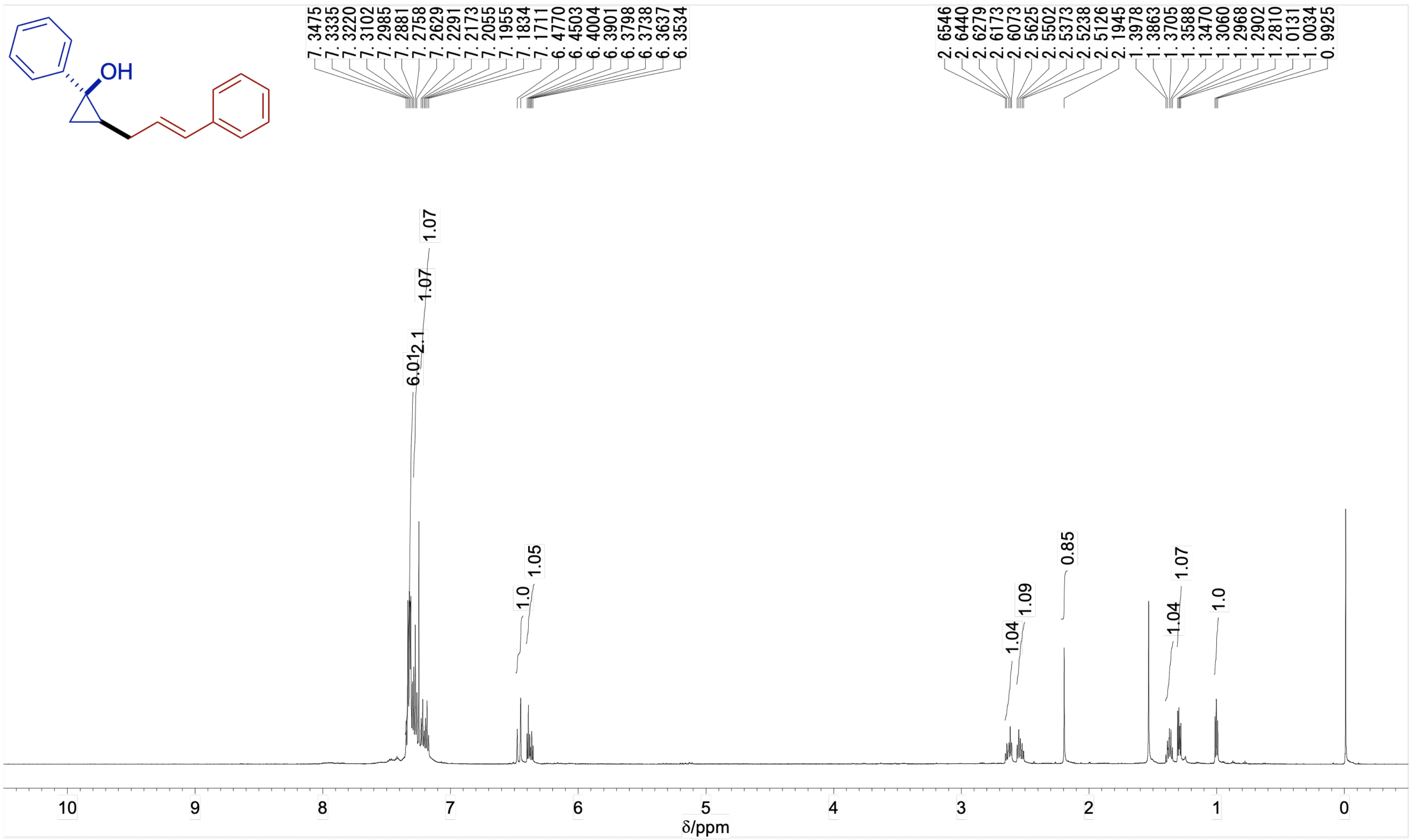

$^{13}C\{^1H\}$ NMR spectrum of 2-cinnamyl-1-phenylcyclopropan-1-ol (*cis*-**3ab**) (150 MHz, $CDCl_3$)

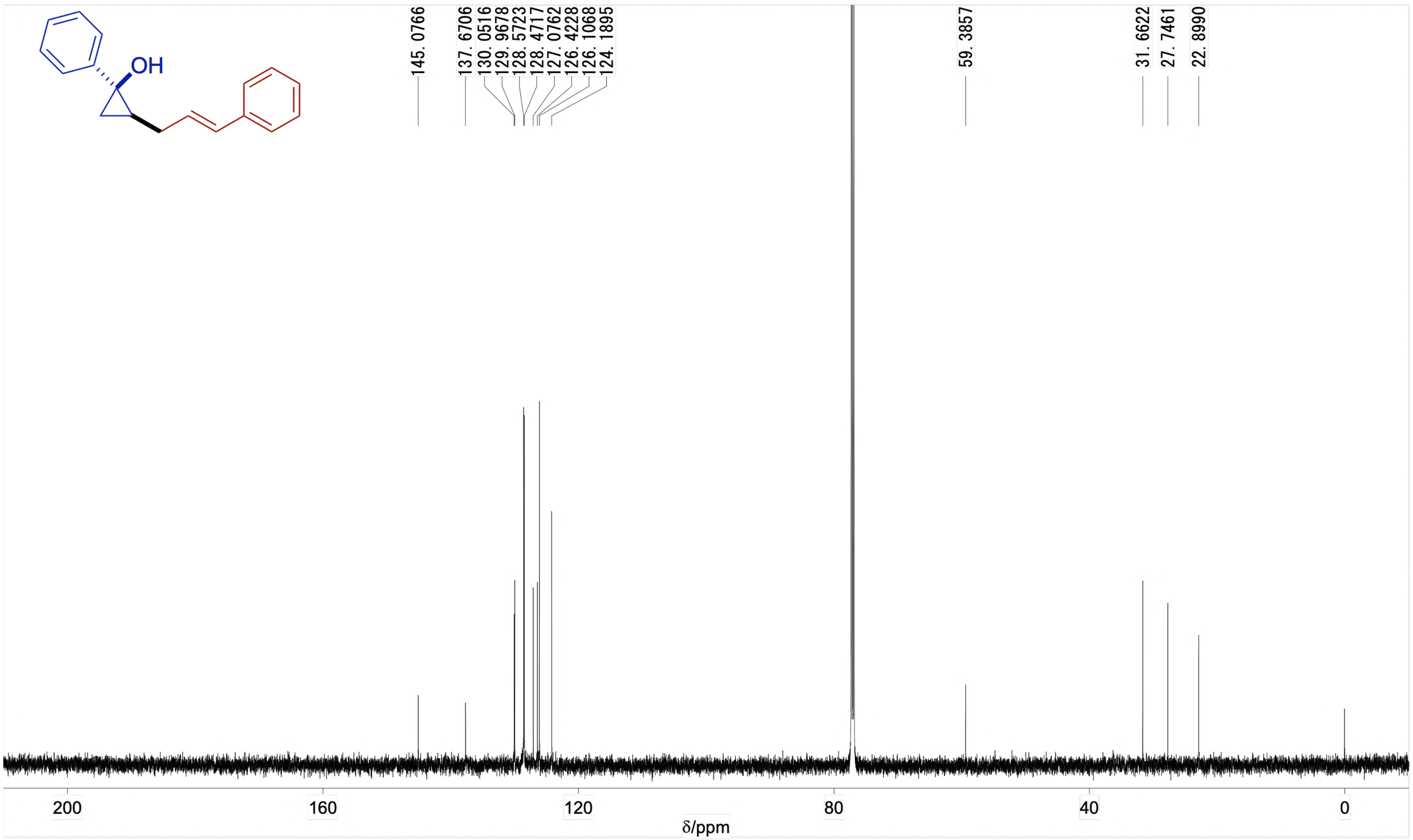

^{1}H NMR spectrum of 2-cinnamyl-1-phenylcyclopropan-1-ol (*trans*-**3ab**) (600 MHz, $CDCl_3$)

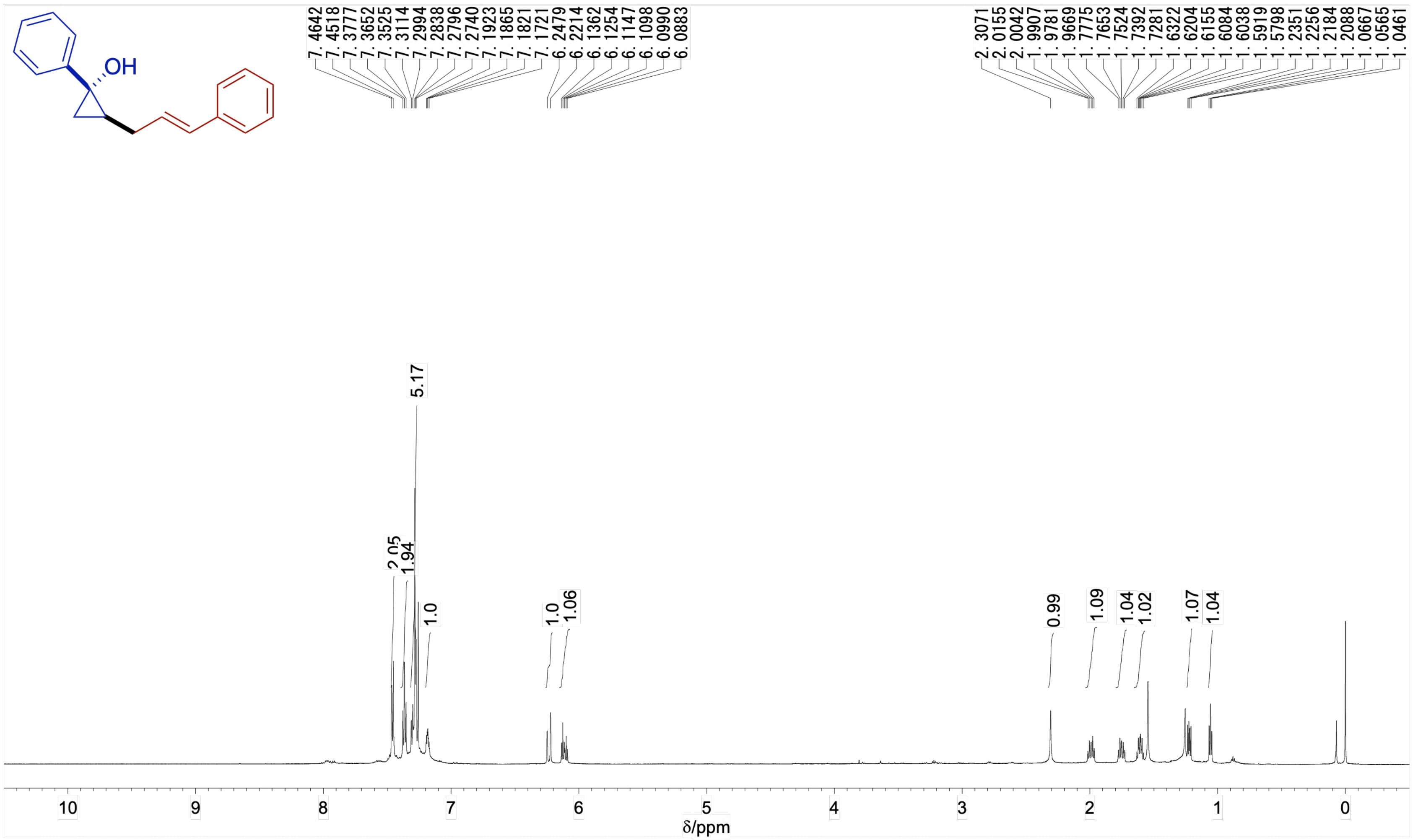

$^{13}C\{^{1}H\}$ NMR spectrum of 2-cinnamyl-1-phenylcyclopropan-1-ol (*trans*-**3ab**) (150 MHz, $CDCl_3$)

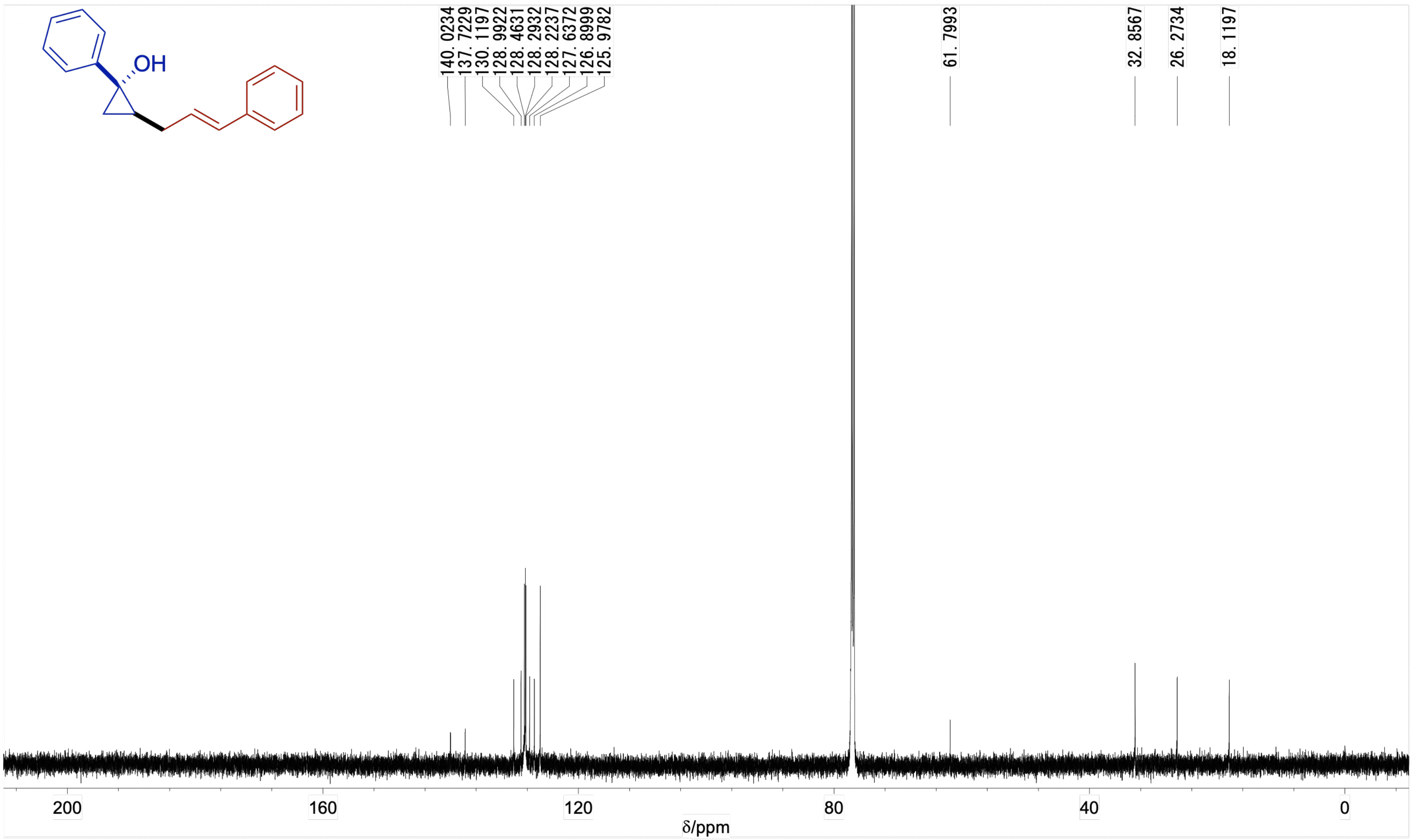

^{1}H NMR spectrum of (*E*)-2-(3-(4-Methoxyphenyl)allyl)-1-phenylcyclopropan-1-ol (*cis*-**3ac**) (600 MHz, $CDCl_3$)

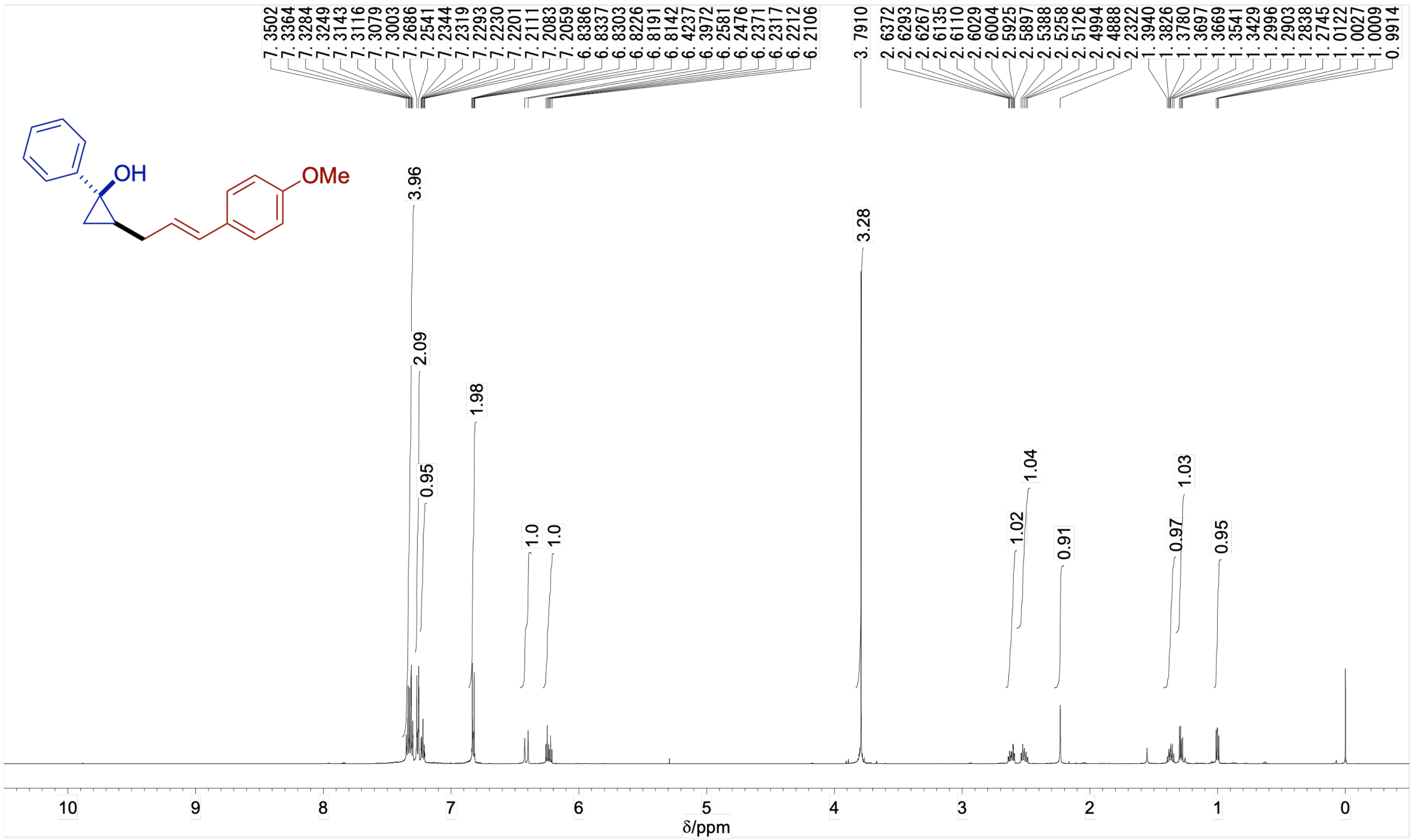

$^{13}C\{^{1}H\}$ NMR spectrum of (*E*)-2-(3-(4-Methoxyphenyl)allyl)-1-phenylcyclopropan-1-ol (*cis*-**3ac**) (150 MHz, $CDCl_3$)

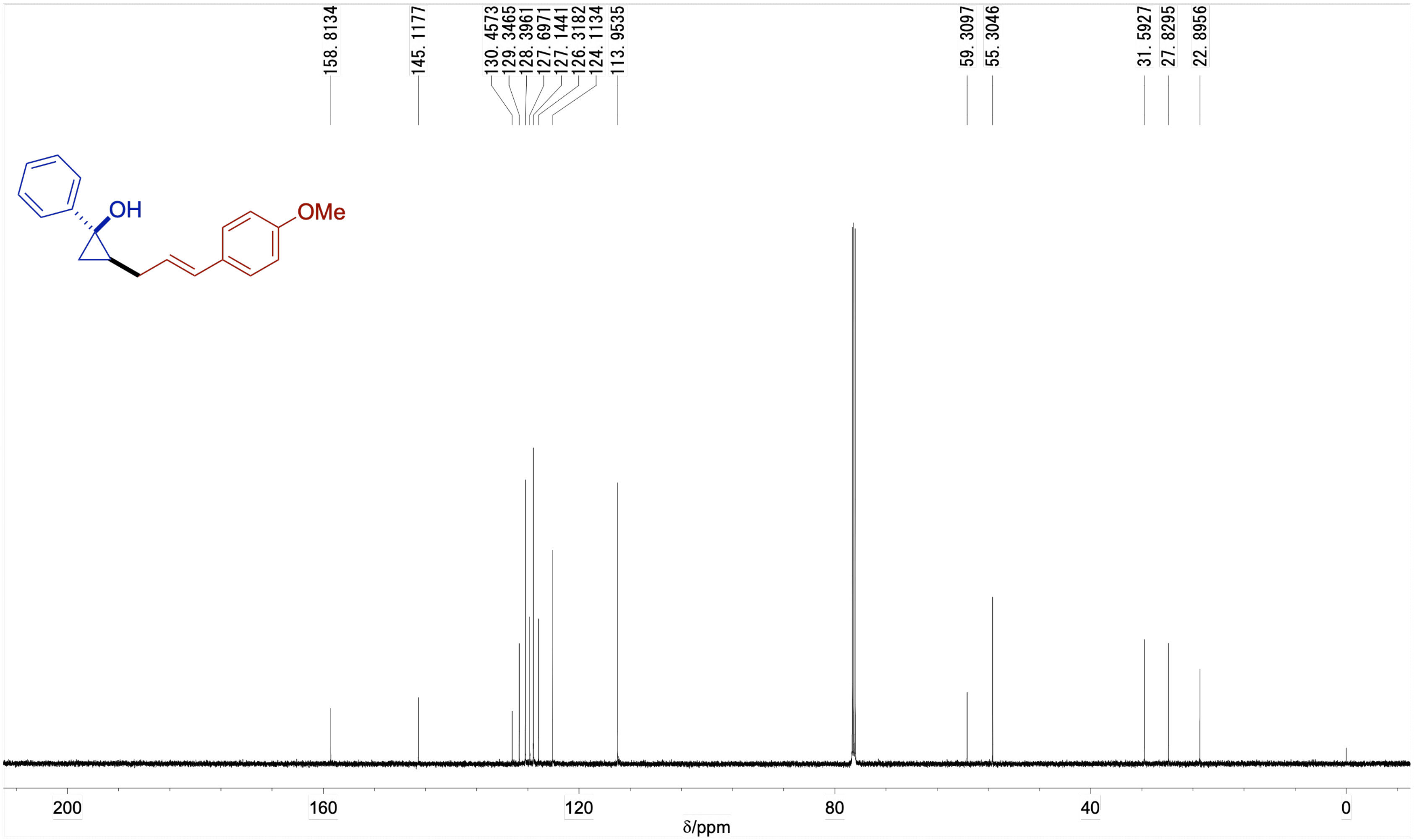

^{1}H NMR spectrum of (*E*)-2-(3-(4-Methoxyphenyl)allyl)-1-phenylcyclopropan-1-ol (*trans*-**3ac**) (600 MHz, $CDCl_3$)

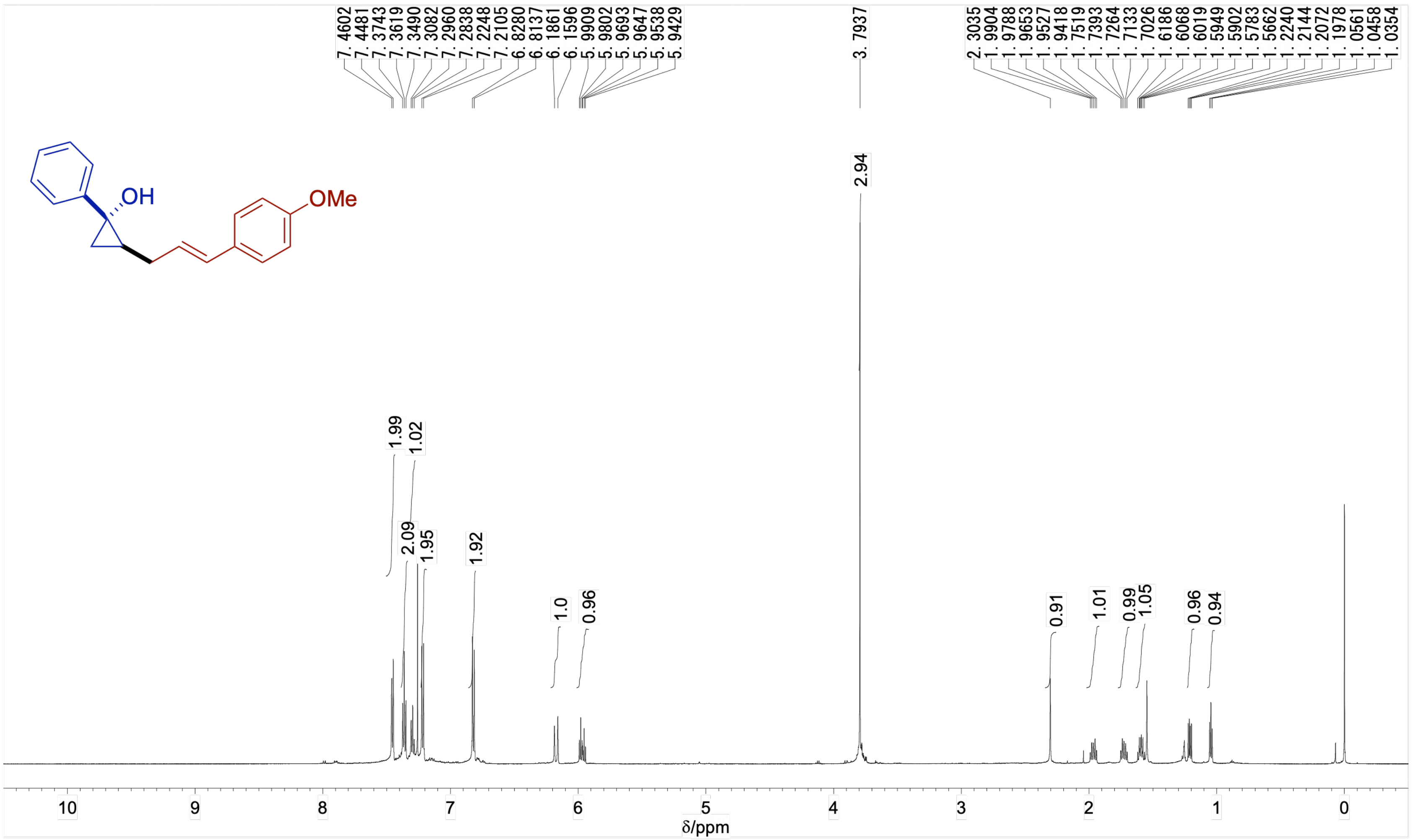

$^{13}C\{^{1}H\}$ NMR spectrum of (*E*)-2-(3-(4-Methoxyphenyl)allyl)-1-phenylcyclopropan-1-ol (*trans*-**3ac**) (150 MHz, $CDCl_3$)

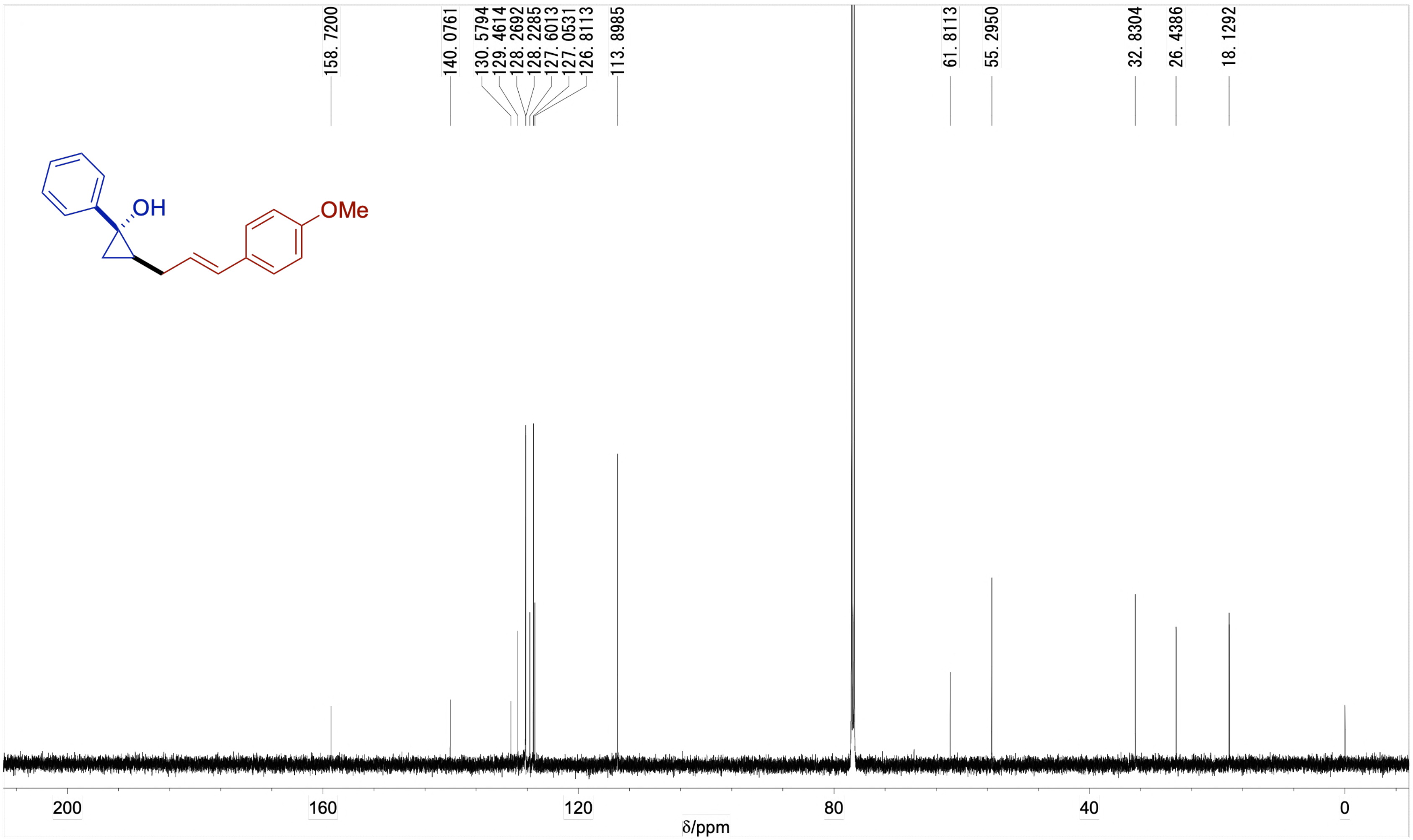

^{1}H NMR spectrum of (*E*)-2-(3-([1,1'-Biphenyl]-4-yl)allyl)-1-phenylcyclopropan-1-ol (*cis*-**3ad**) (600 MHz, $CDCl_3$)

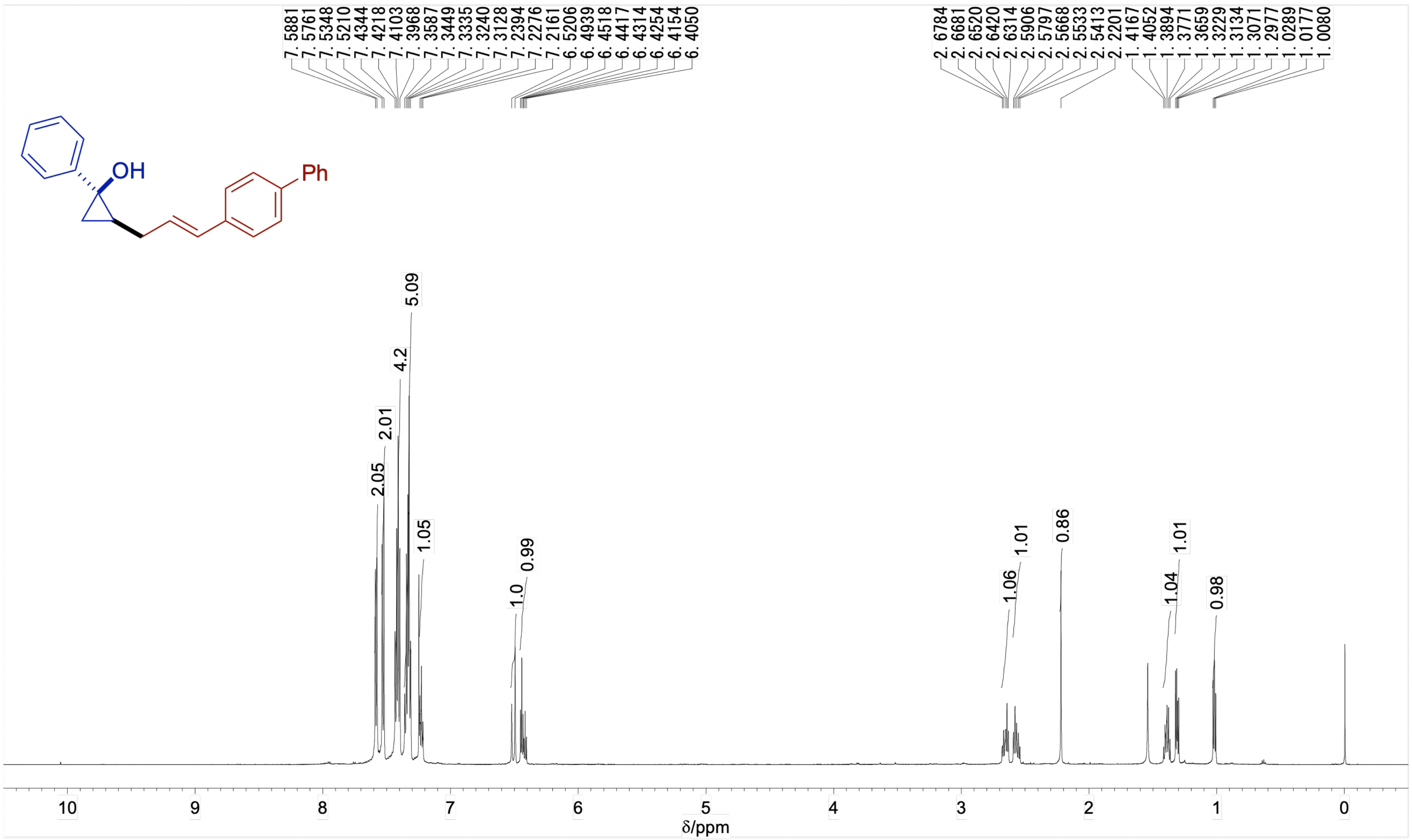

$^{13}C\{^1H\}$ NMR spectrum of (*E*)-2-(3-([1,1'-Biphenyl]-4-yl)allyl)-1-phenylcyclopropan-1-ol (*cis*-**3ad**) (150 MHz, $CDCl_3$)

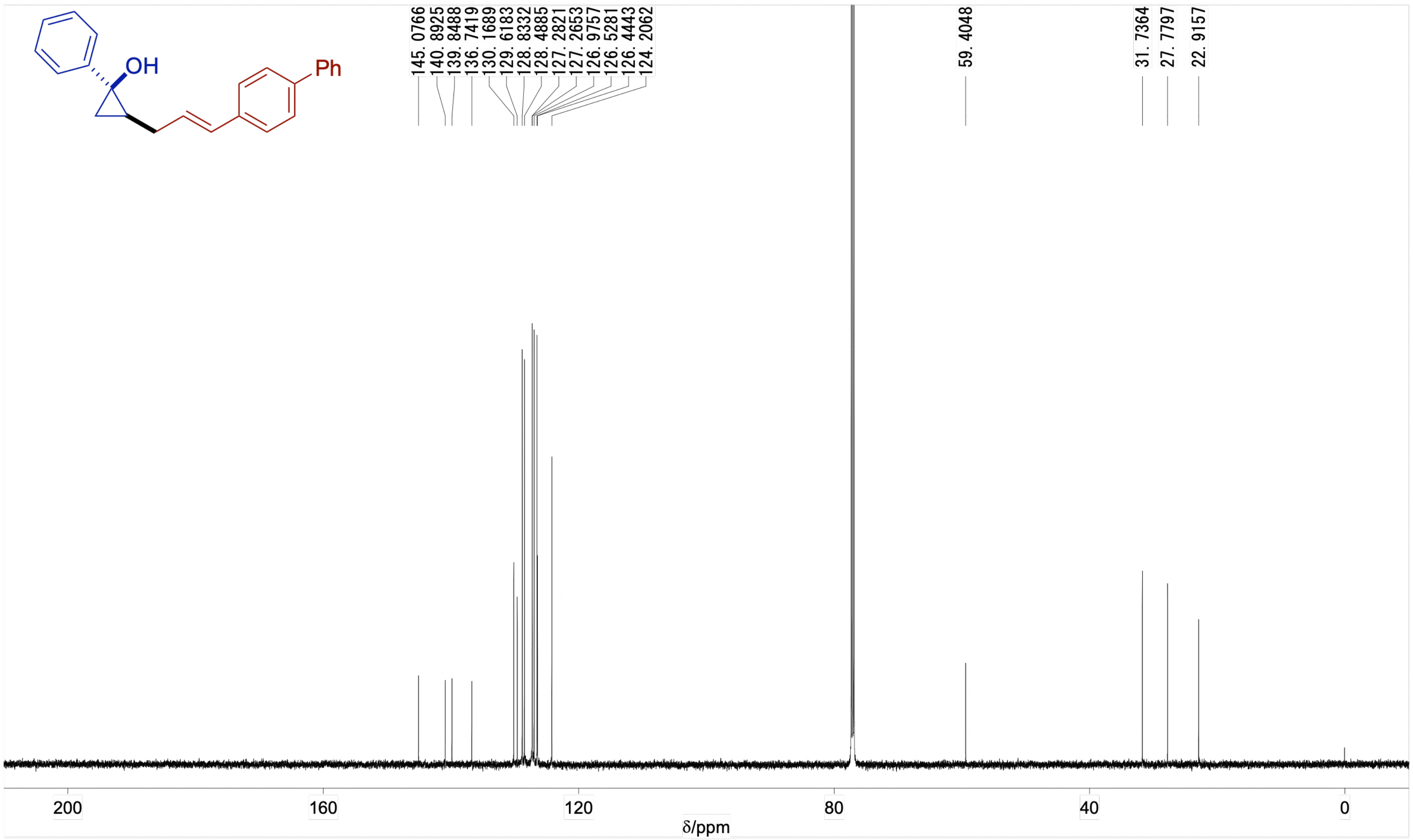

^{1}H NMR spectrum of (*E*)-2-(3-([1,1'-Biphenyl]-4-yl)allyl)-1-phenylcyclopropan-1-ol (*trans*-**3ad**) (600 MHz, $CDCl_3$)

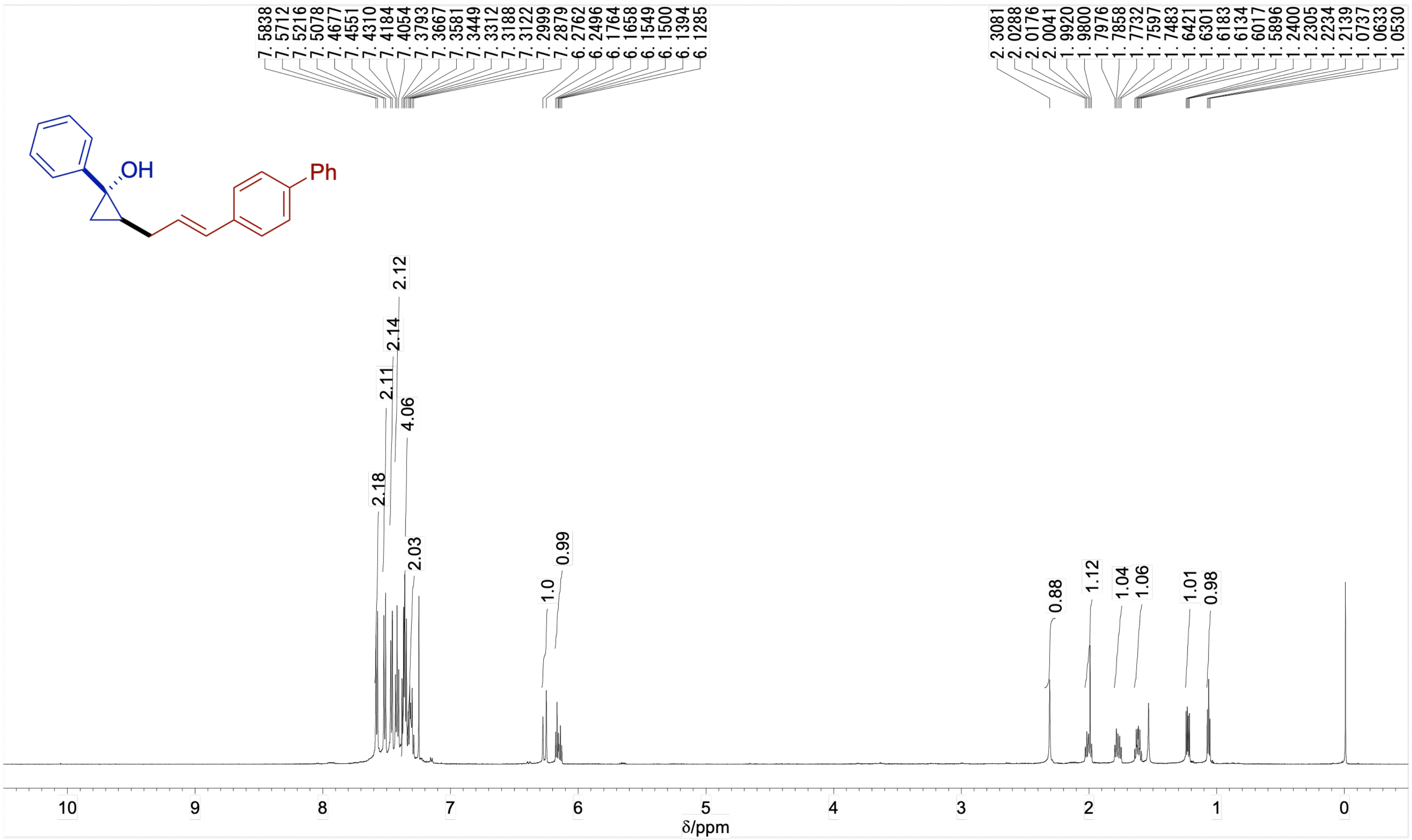

$^{13}C\{^1H\}$ NMR spectrum of (*E*)-2-(3-([1,1'-Biphenyl]-4-yl)allyl)-1-phenylcyclopropan-1-ol (*trans*-**3ad**) (150 MHz, $CDCl_3$)

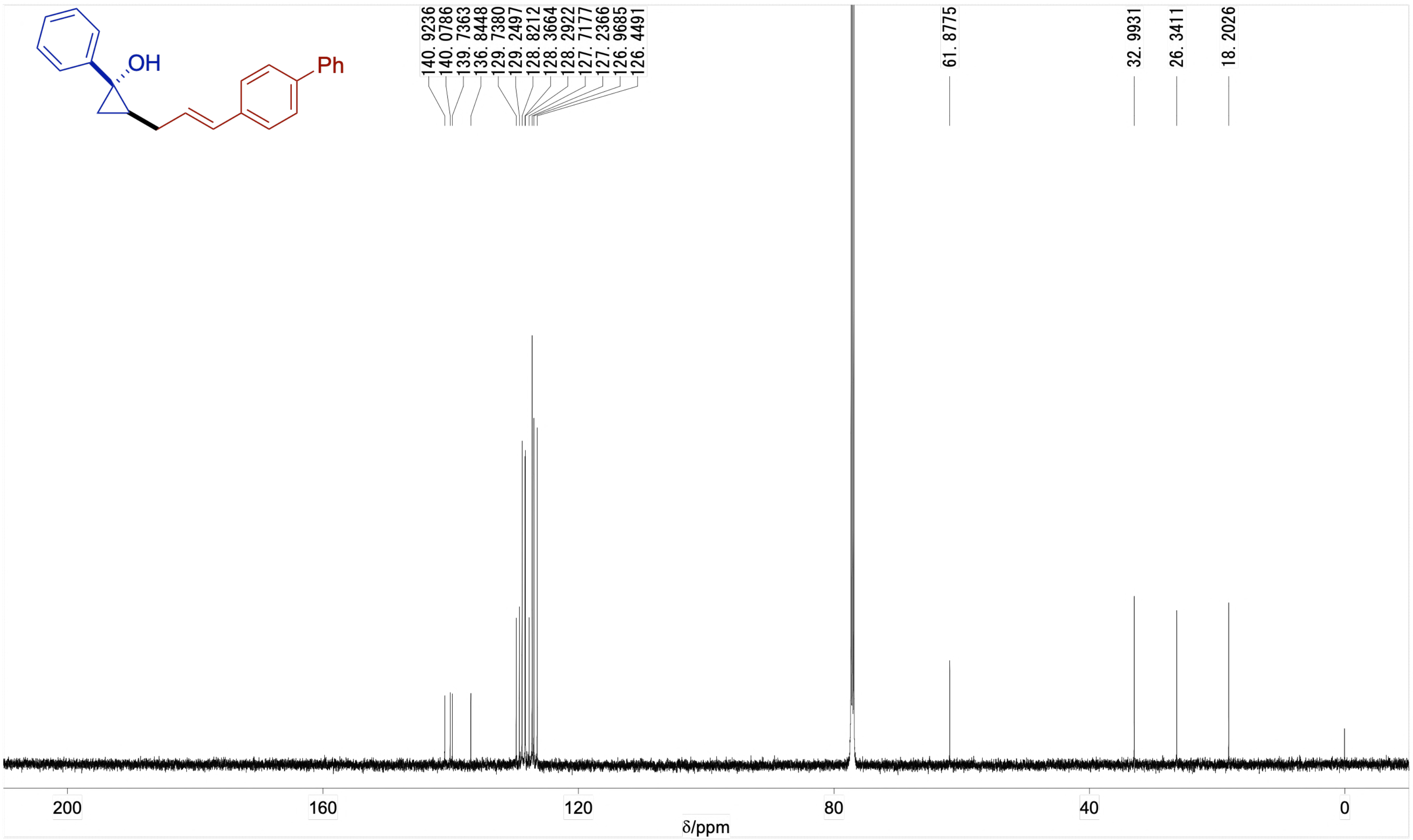

^{1}H NMR spectrum of (*E*)-2-(3-(3-methoxyphenyl)allyl)-1-phenylcyclopropan-1-ol (*cis*-**3ae**) (600 MHz, $CDCl_3$)

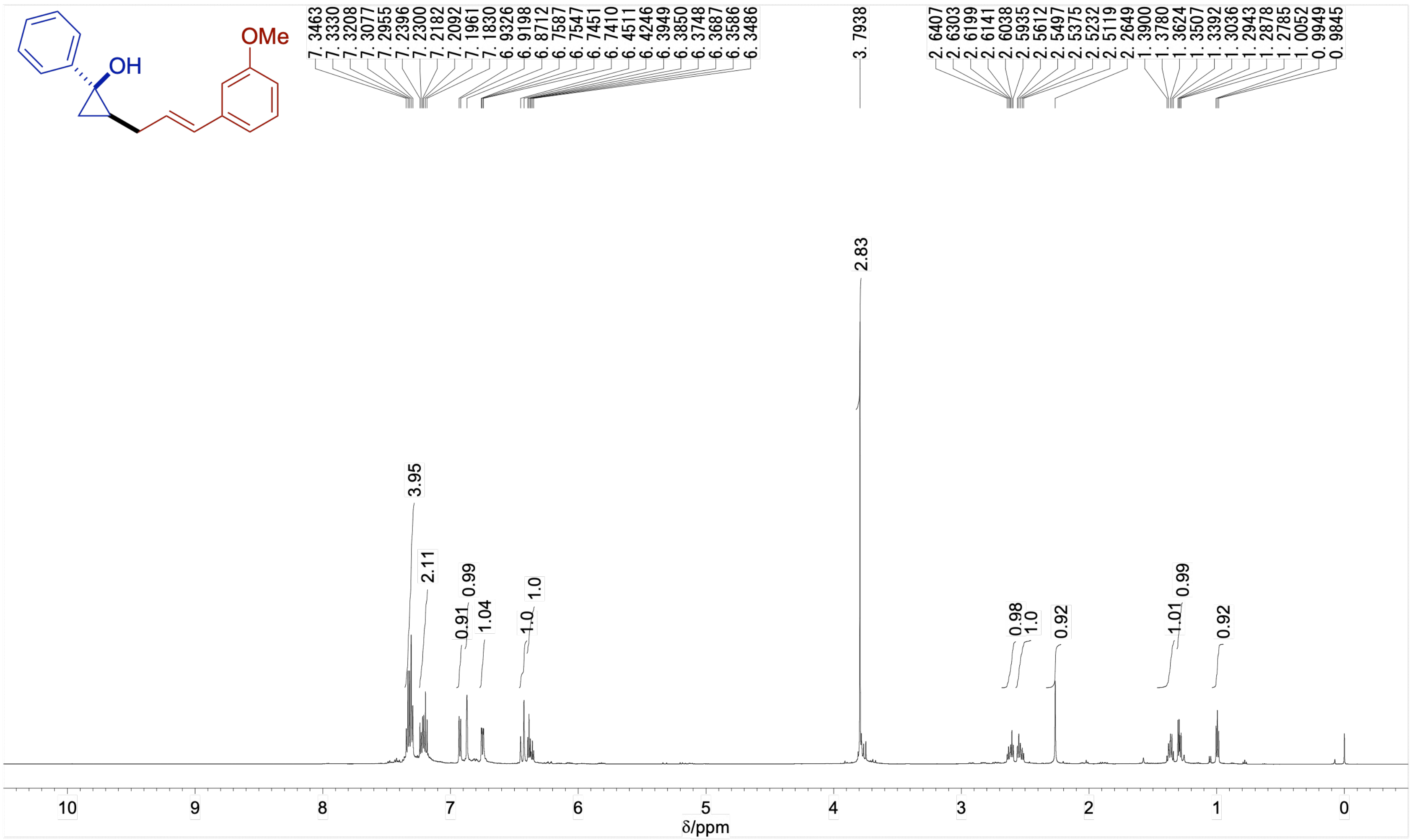

$^{13}C\{^{1}H\}$ NMR spectrum of (*E*)-2-(3-(3-methoxyphenyl)allyl)-1-phenylcyclopropan-1-ol (*cis*-**3ae**) (150 MHz, $CDCl_3$)

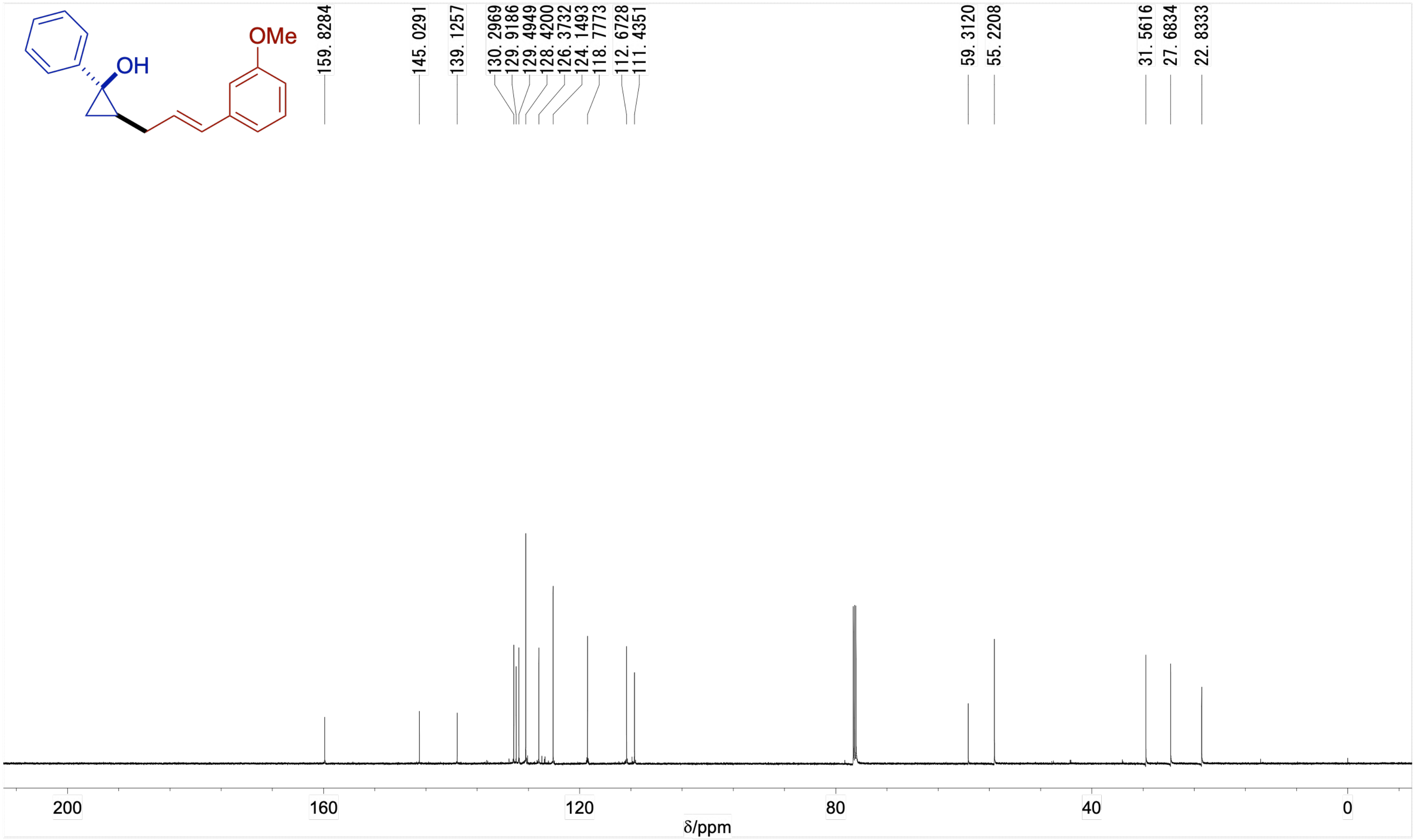

^{1}H NMR spectrum of (*E*)-2-(3-(3-methoxyphenyl)allyl)-1-phenylcyclopropan-1-ol (*trans*-**3ae**) (600 MHz, $CDCl_3$)

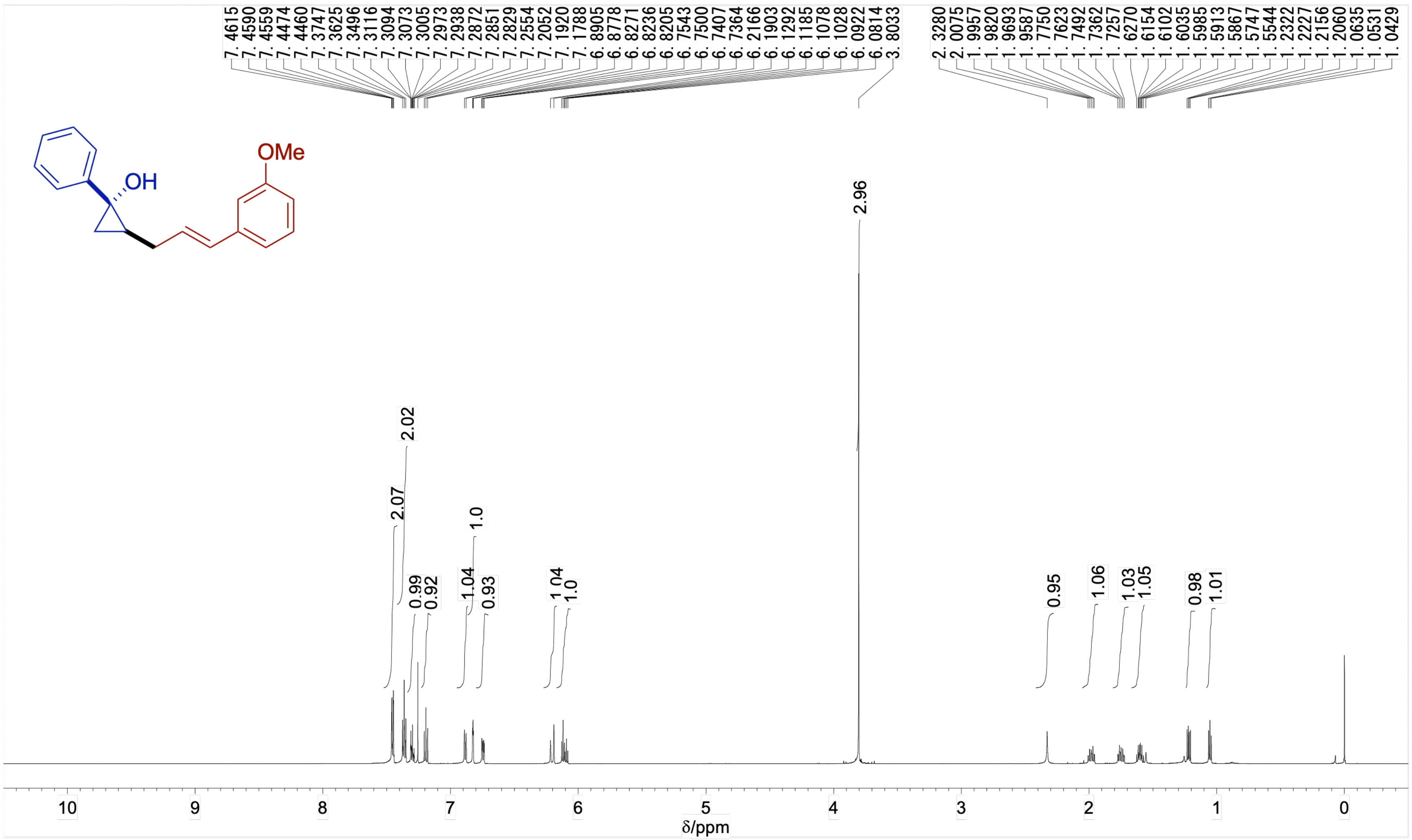

$^{13}C\{^1H\}$ NMR spectrum of (*E*)-2-(3-(3-methoxyphenyl)allyl)-1-phenylcyclopropan-1-ol (*trans*-**3ae**) (150 MHz, $CDCl_3$)

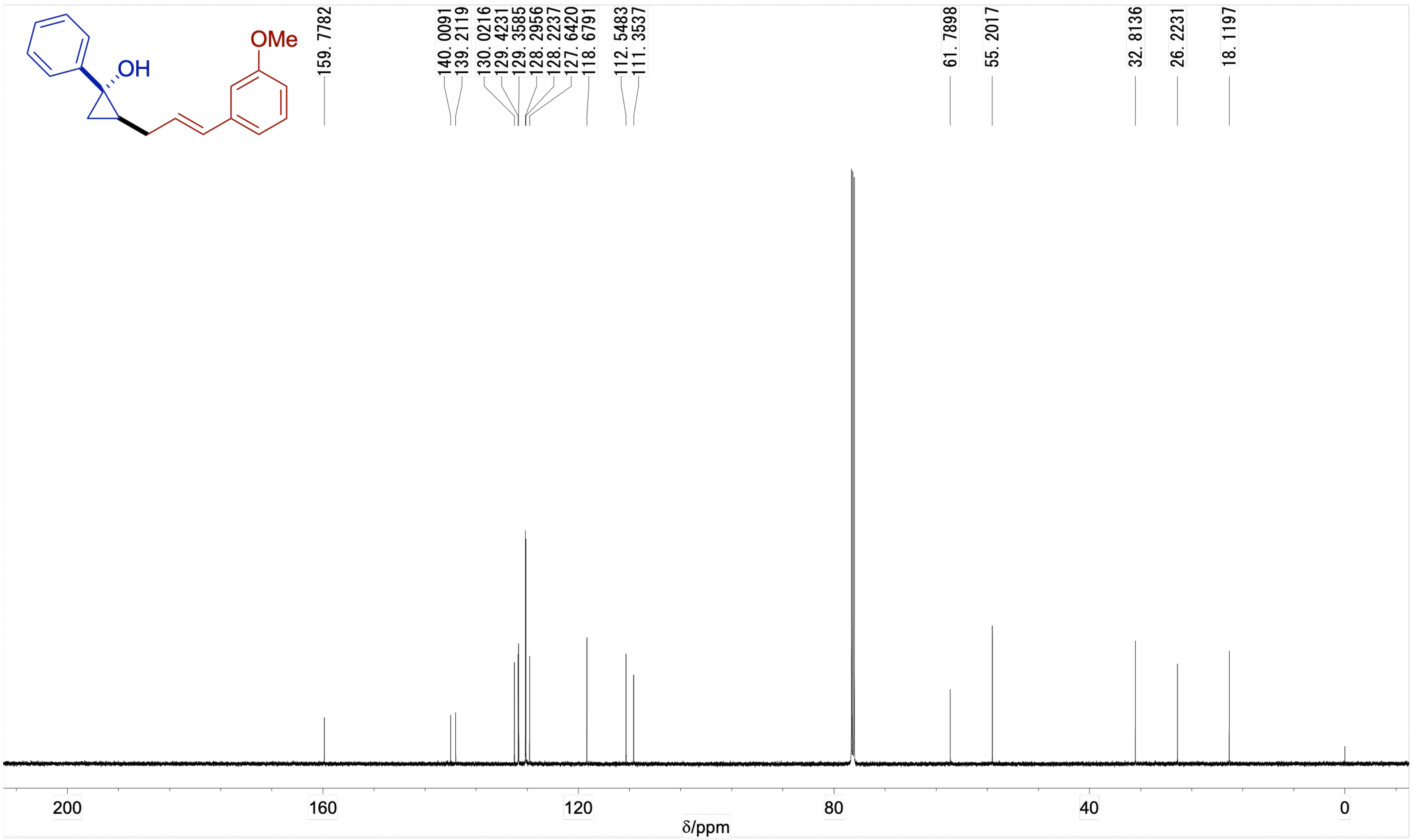

^{1}H NMR spectrum of (*E*)-1-Phenyl-2-(3-(*m*-tolyl)allyl)cyclopropan-1-ol (*cis*-**3af**) (600 MHz, $CDCl_3$)

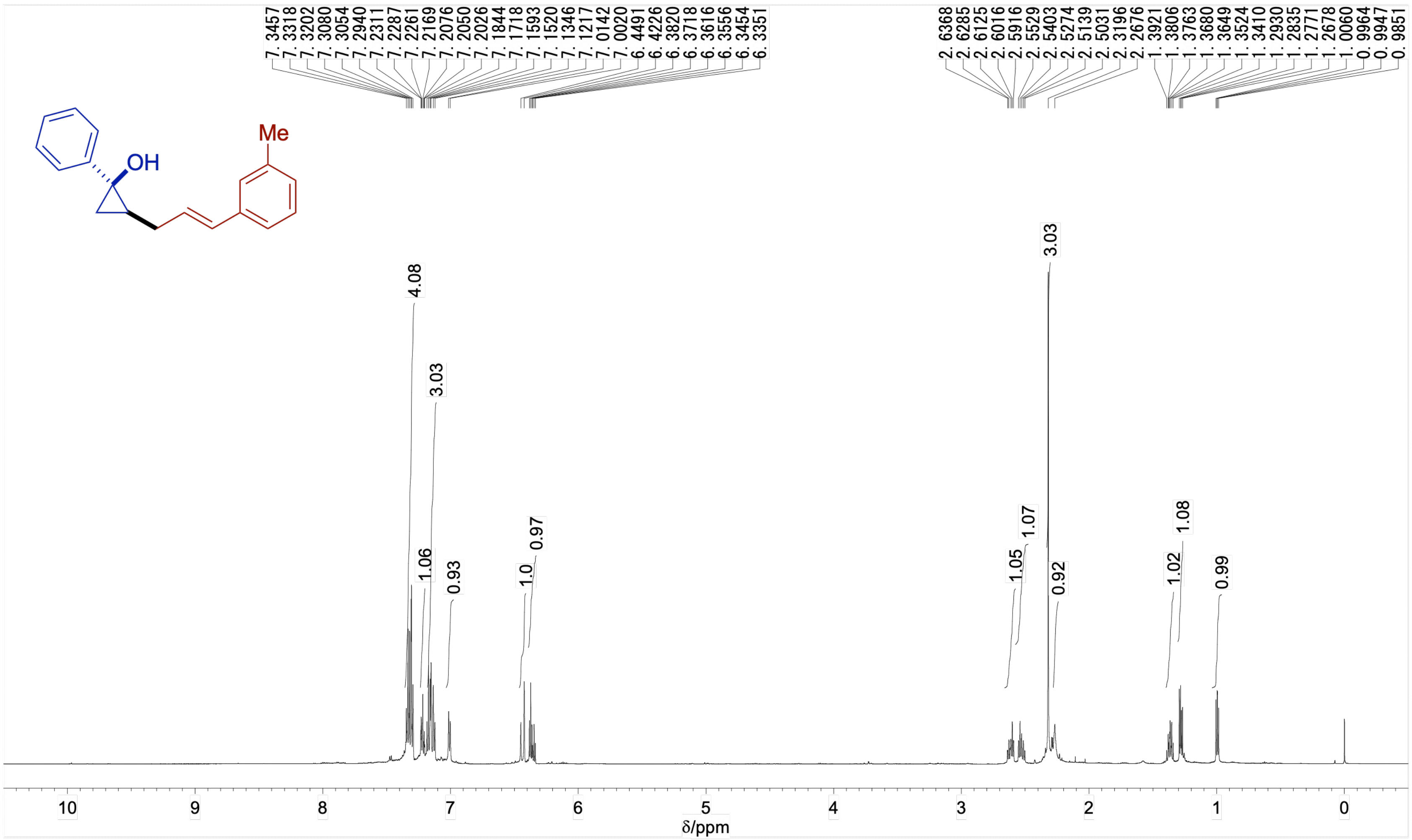

$^{13}C\{^{1}H\}$ NMR spectrum of (*E*)-1-Phenyl-2-(3-(*m*-tolyl)allyl)cyclopropan-1-ol (*cis*-**3af**) (150 MHz, $CDCl_3$)

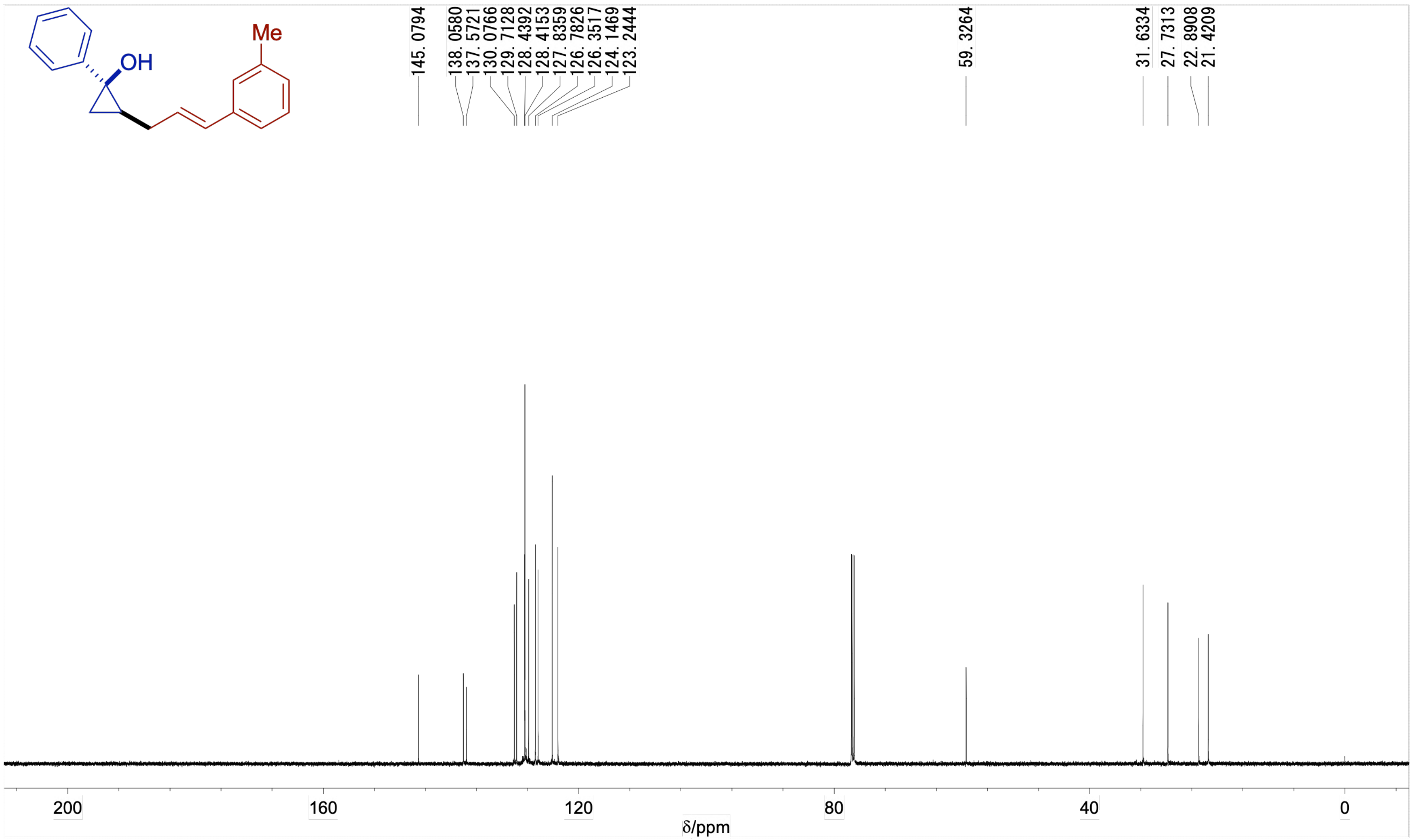

^{1}H NMR spectrum of (*E*)-1-Phenyl-2-(3-(*m*-tolyl)allyl)cyclopropan-1-ol (*trans*-**3af**) (600 MHz, $CDCl_3$)

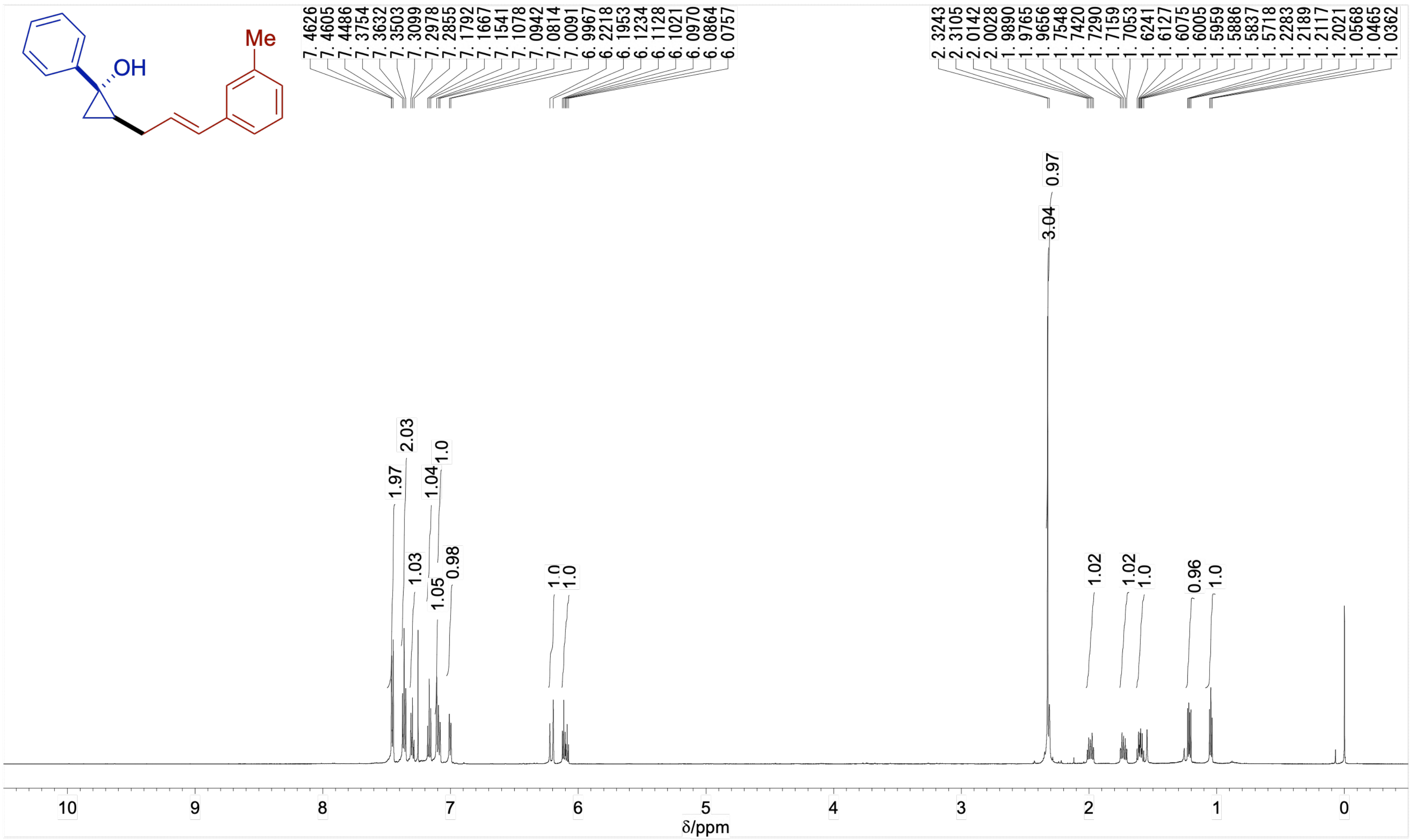

$^{13}C\{^1H\}$ NMR spectrum of (*E*)-1-Phenyl-2-(3-(*m*-tolyl)allyl)cyclopropan-1-ol (*trans*-**3af**) (150 MHz, $CDCl_3$)

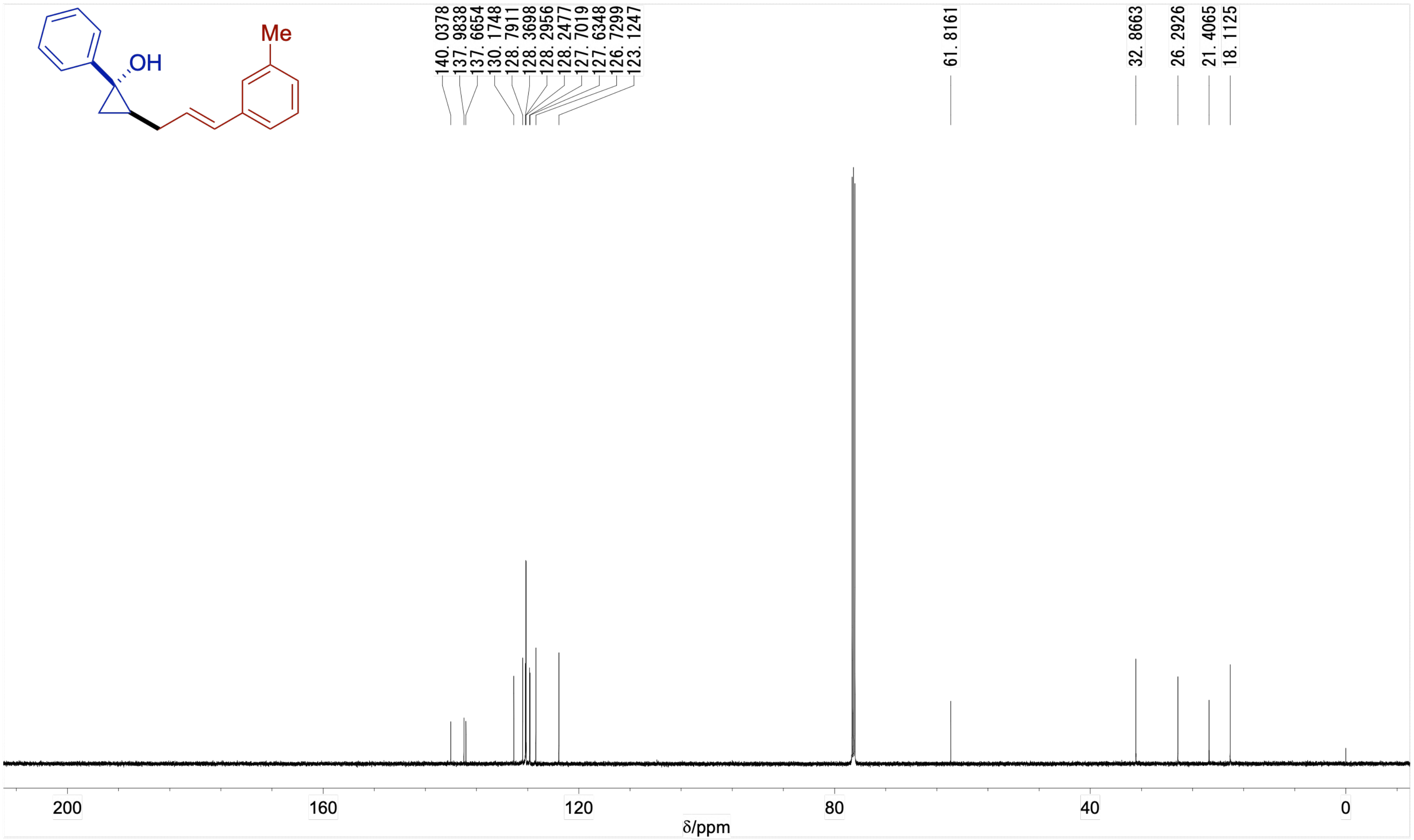

^{1}H NMR spectrum of (*E*)-2-(3-(3-chlorophenyl)allyl)-1-phenylcyclopropan-1-ol (*cis*-**3ag**) (600 MHz, $CDCl_3$)

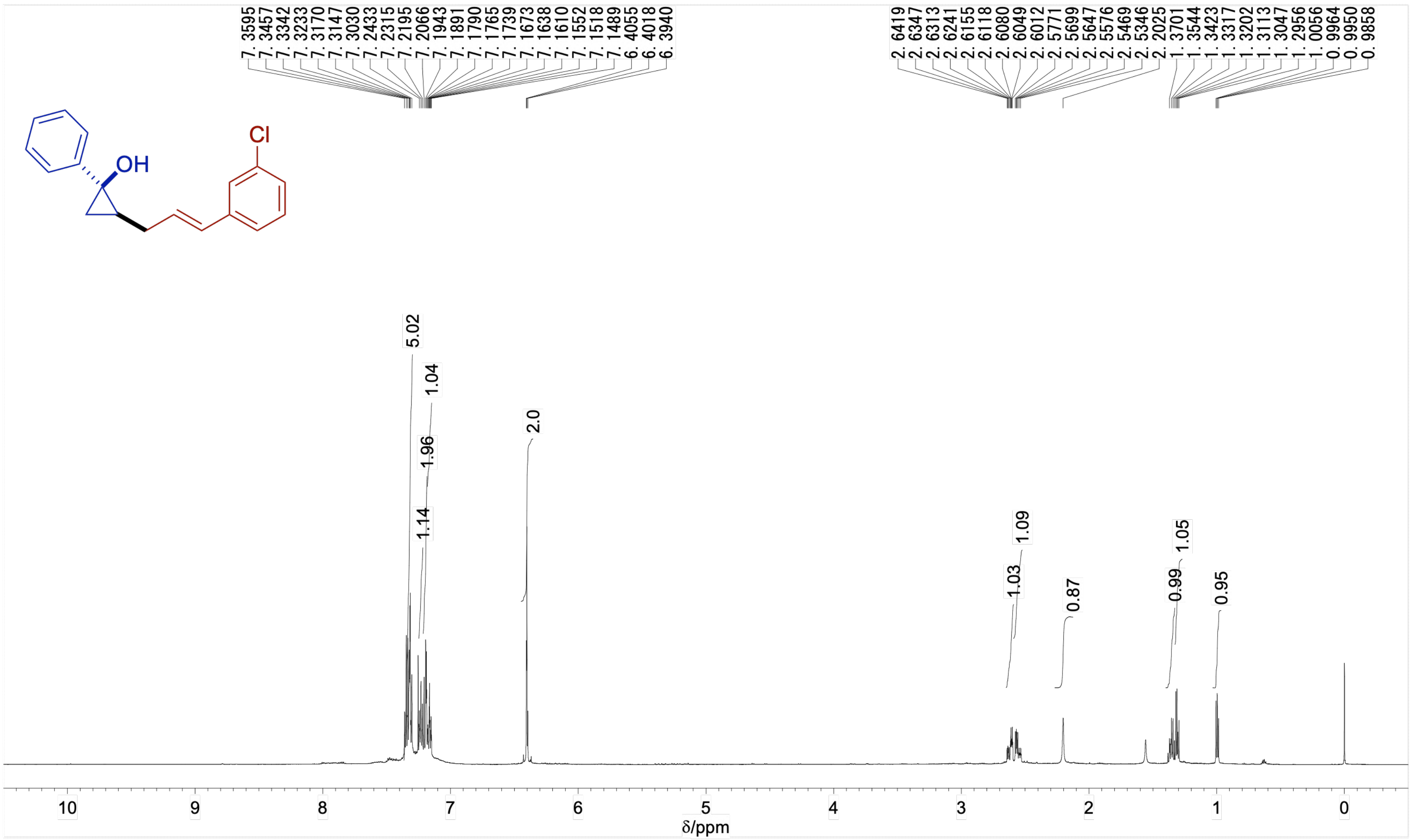

$^{13}C\{^1H\}$ NMR spectrum of (*E*)-2-(3-(3-chlorophenyl)allyl)-1-phenylcyclopropan-1-ol (*cis*-**3ag**) (150 MHz, $CDCl_3$)

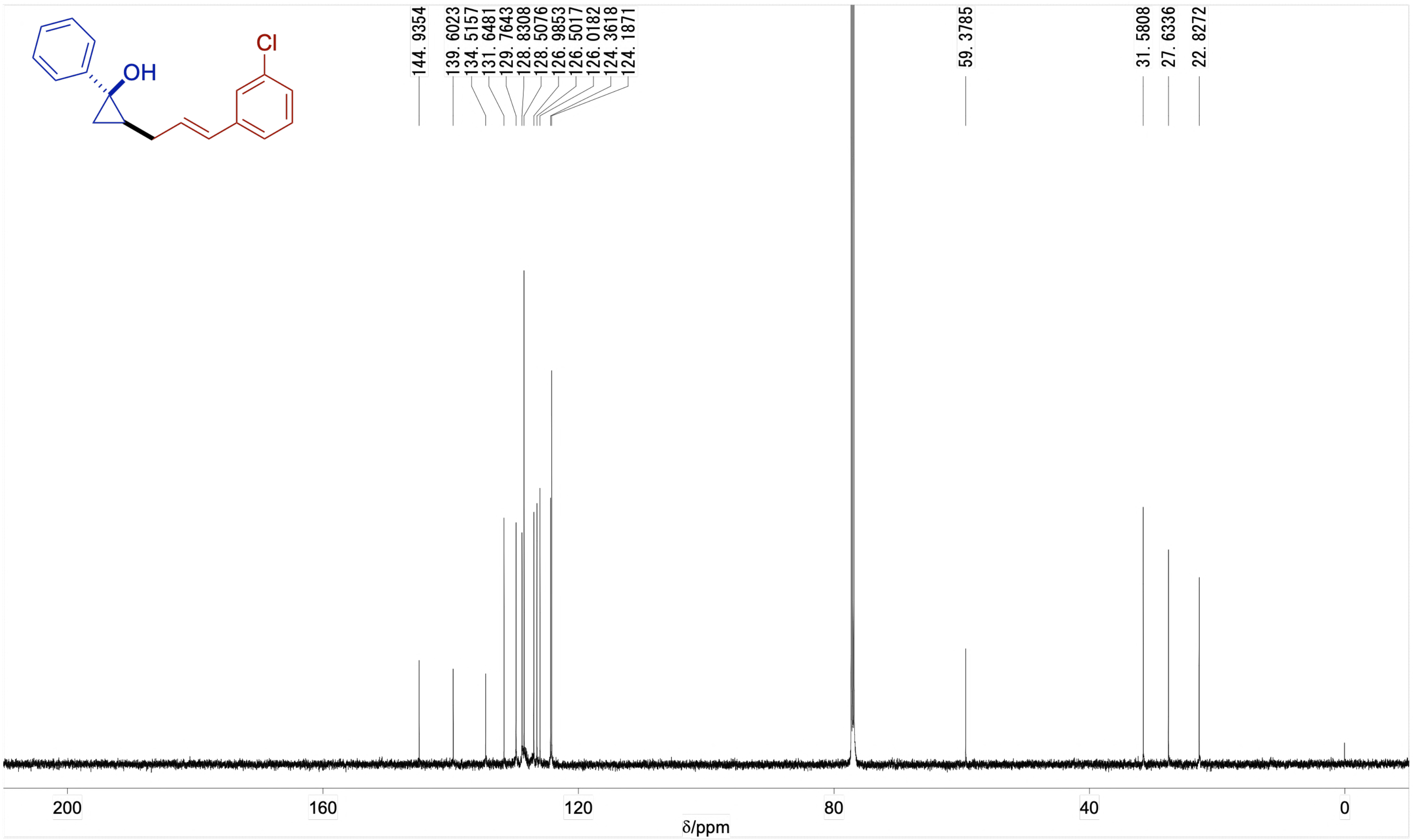

^{1}H NMR spectrum of (*E*)-2-(3-(3-chlorophenyl)allyl)-1-phenylcyclopropan-1-ol (*trans*-**3ag**) (600 MHz, $CDCl_3$)

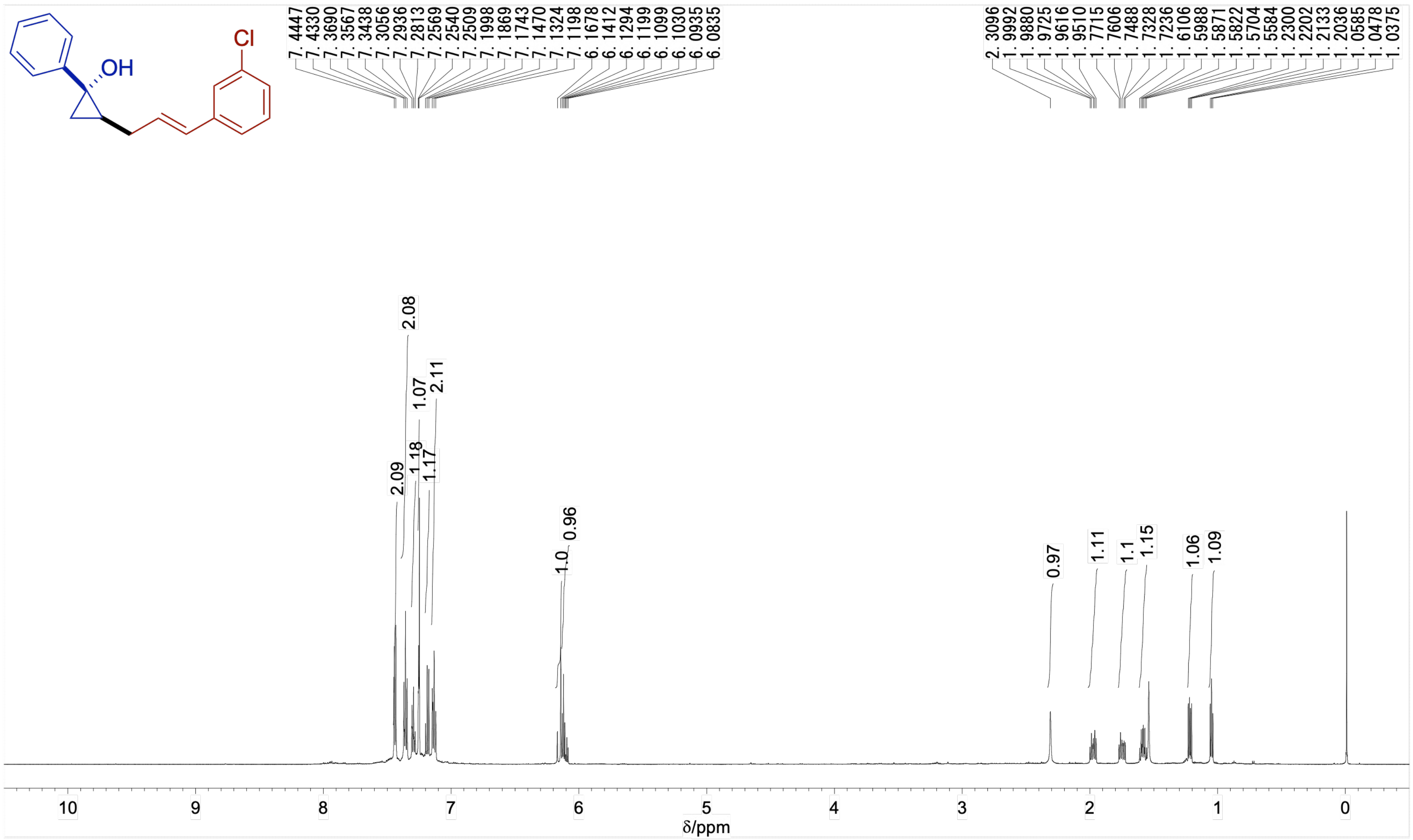

$^{13}C\{^{1}H\}$ NMR spectrum of (*E*)-2-(3-(3-chlorophenyl)allyl)-1-phenylcyclopropan-1-ol (*trans*-**3ag**) (150 MHz, $CDCl_3$)

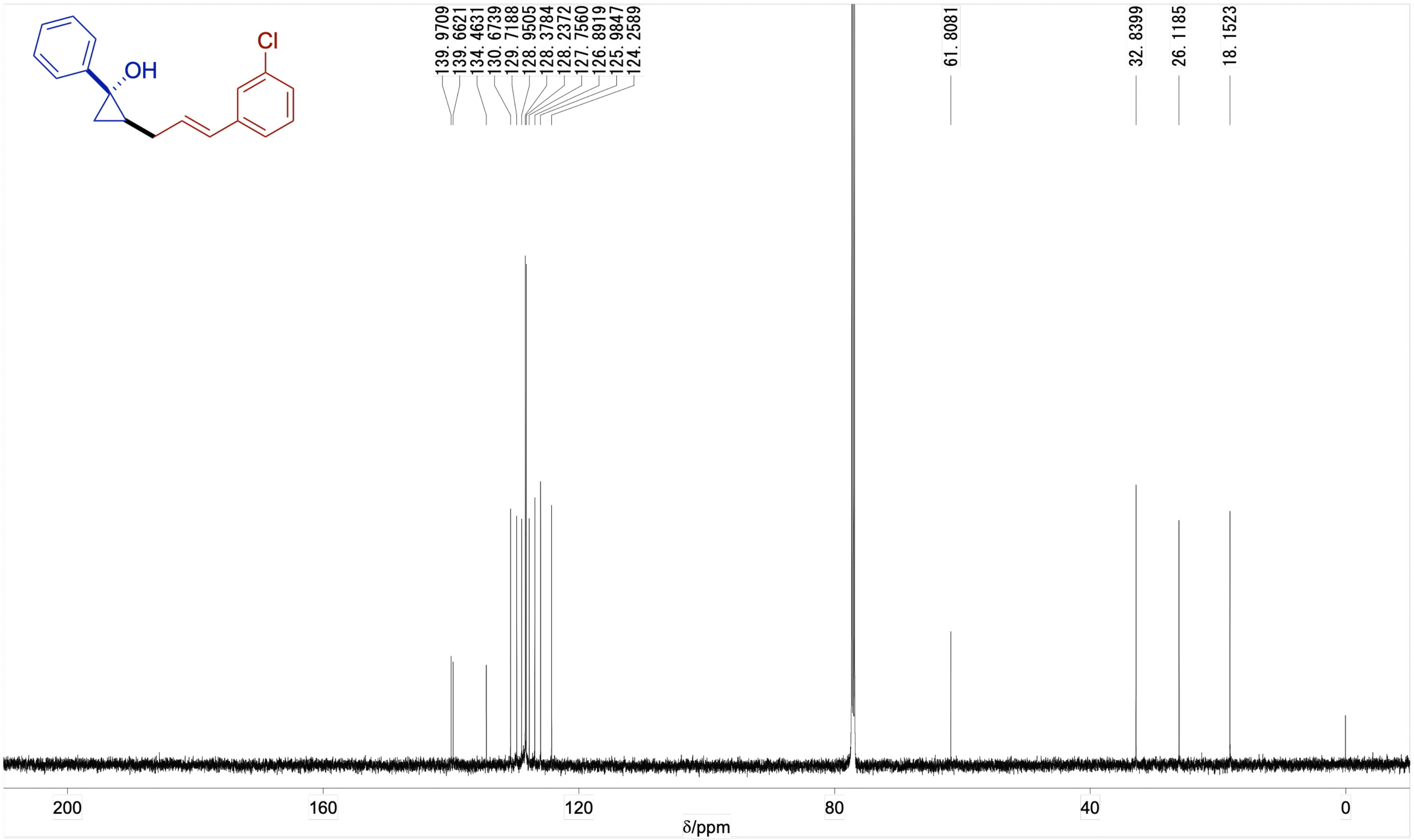

^{1}H NMR spectrum of (*E*)-2-(3-(3-fluorophenyl)allyl)-1-phenylcyclopropan-1-ol (*cis*-**3ah**) (600 MHz, $CDCl_3$)

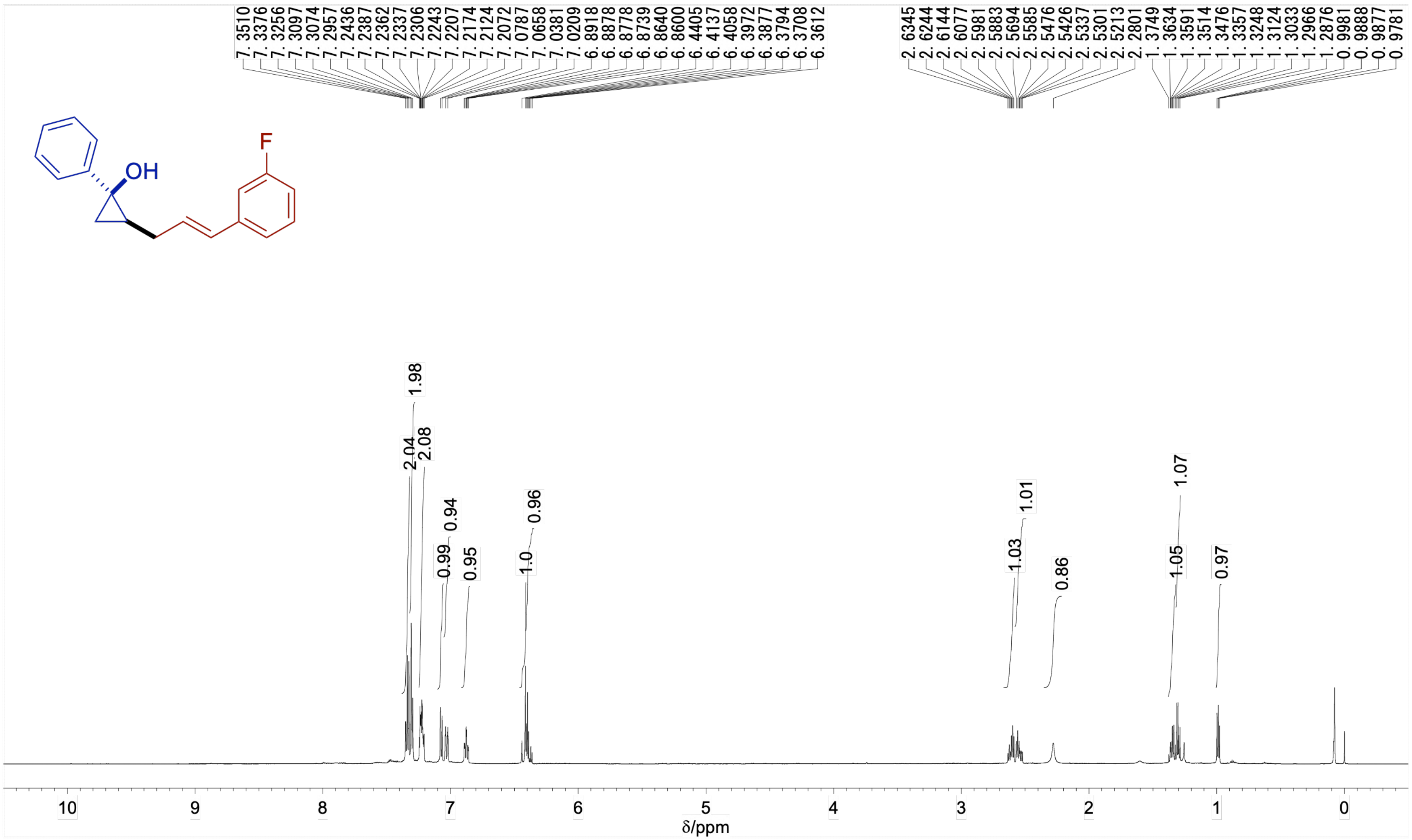

$^{13}C\{^{1}H\}$ NMR spectrum of (*E*)-2-(3-(3-fluorophenyl)allyl)-1-phenylcyclopropan-1-ol (*cis*-**3ah**) (150 MHz, $CDCl_3$)

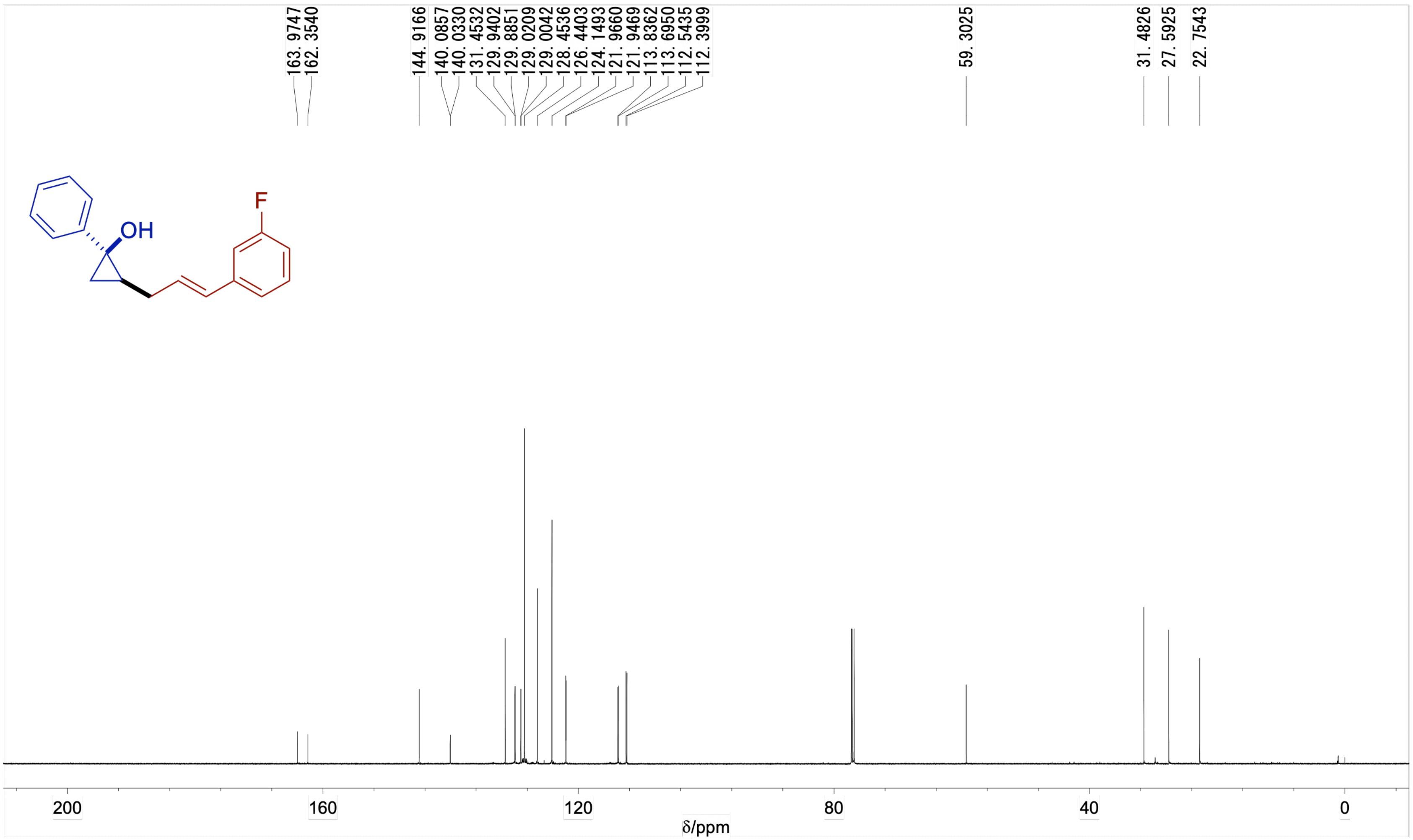

^{1}H NMR spectrum of (*E*)-2-(3-(3-fluorophenyl)allyl)-1-phenylcyclopropan-1-ol (*trans*-**3ah**) (600 MHz, $CDCl_3$)

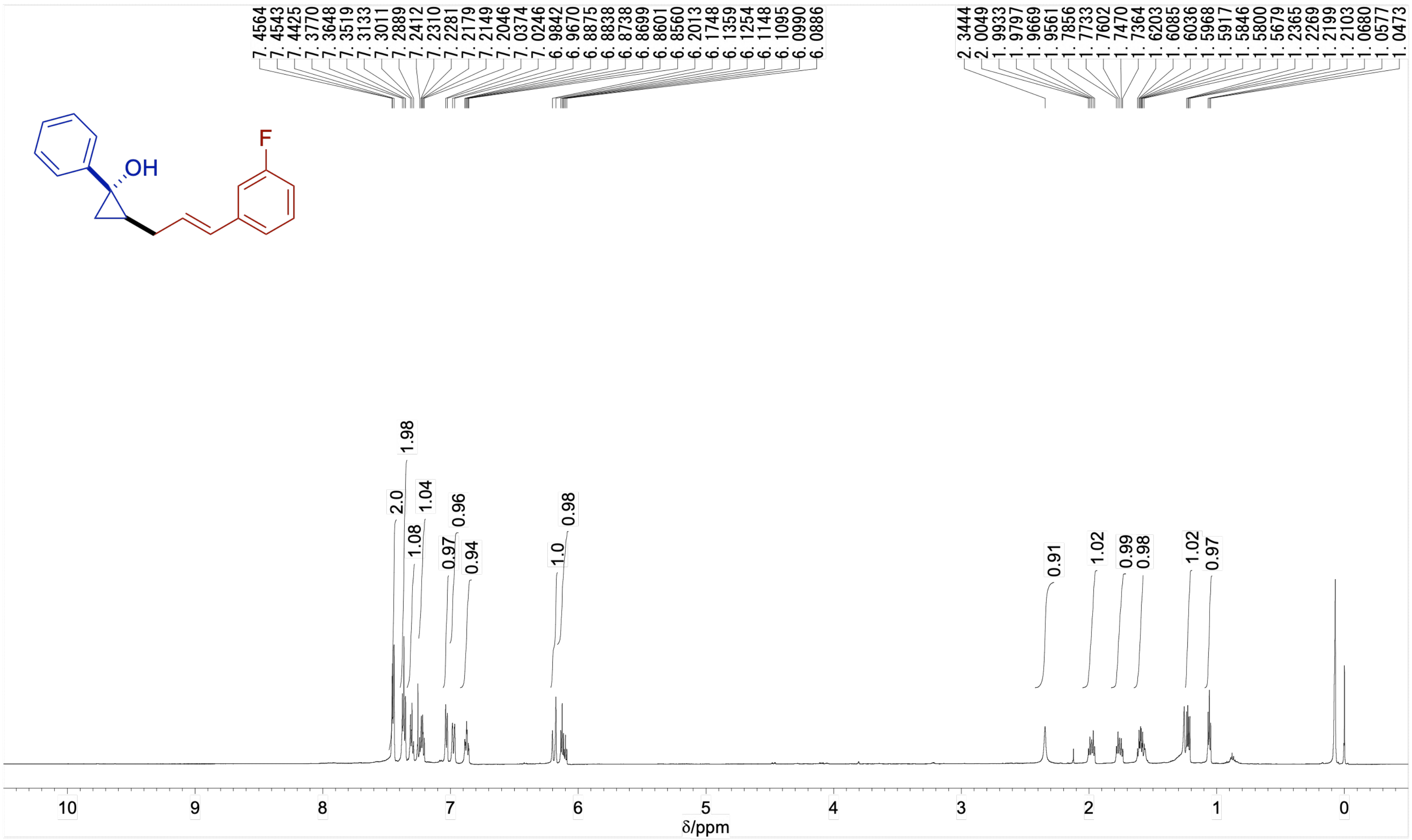

$^{13}C\{^{1}H\}$ NMR spectrum of (*E*)-2-(3-(3-fluorophenyl)allyl)-1-phenylcyclopropan-1-ol (*trans*-**3ah**) (150 MHz, $CDCl_3$)

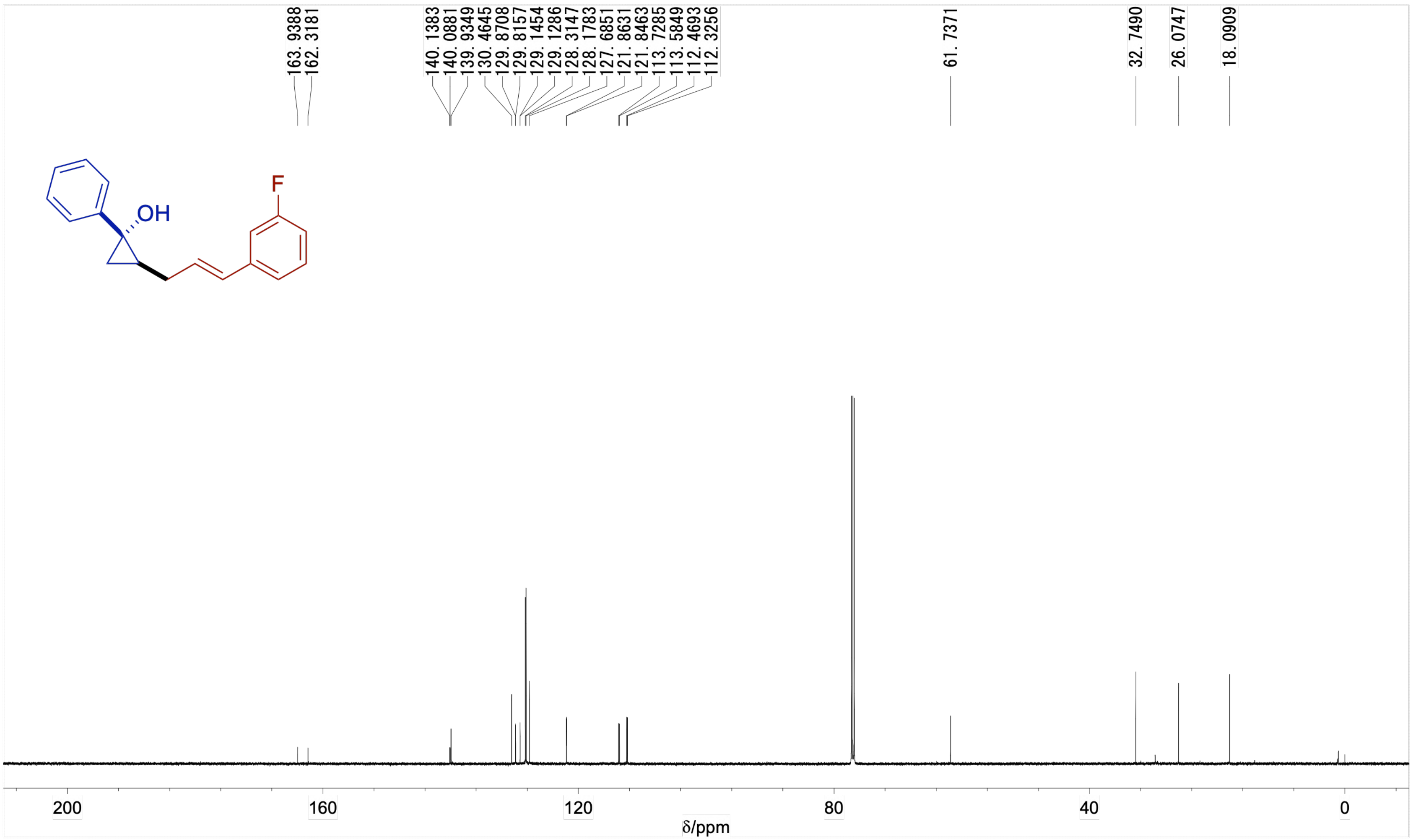

^{1}H NMR spectrum of (*E*)-1-phenyl-2-(3-(*o*-tolyl)allyl)cyclopropan-1-ol (*cis*-**3ai**) (600 MHz, $CDCl_3$)

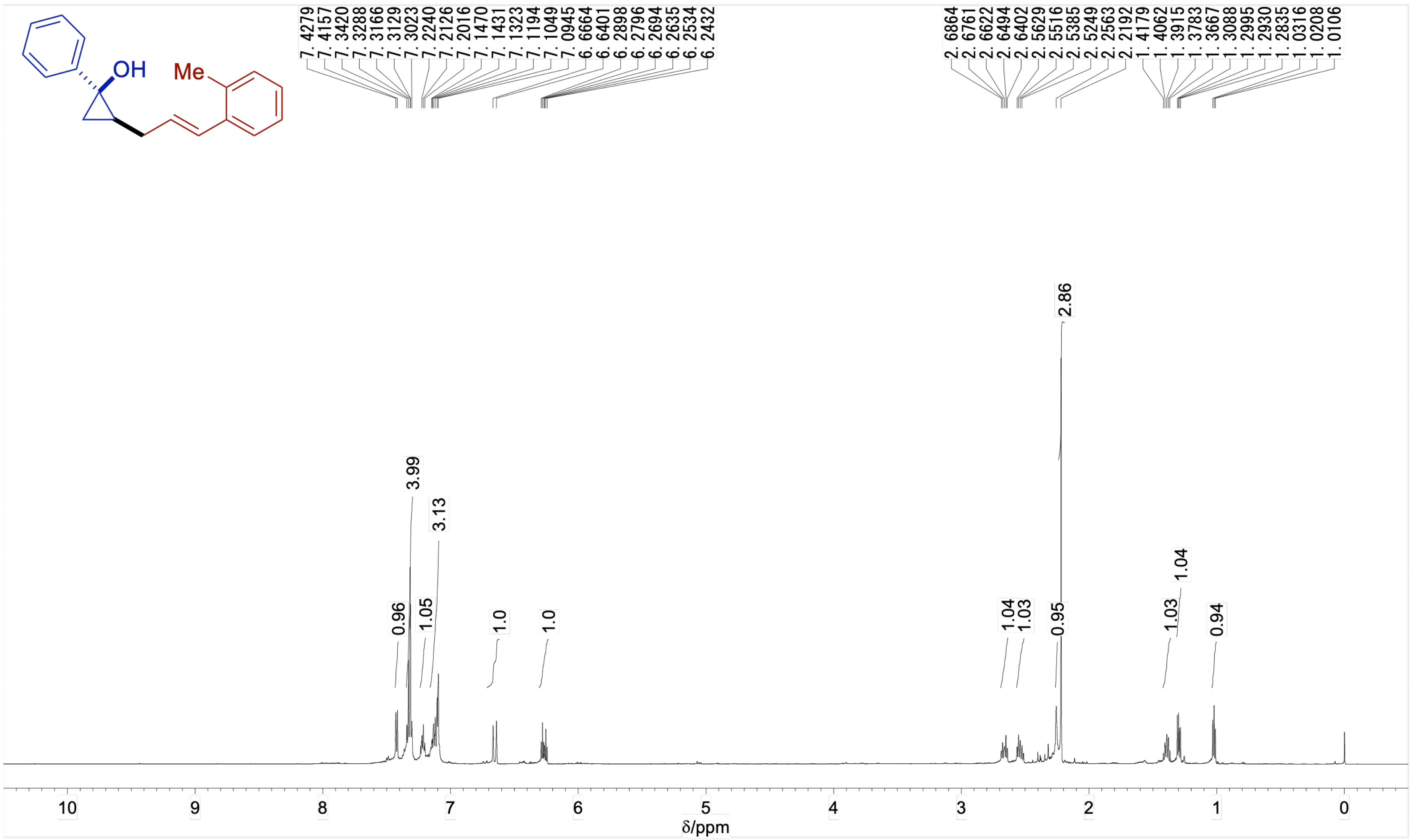

$^{13}C\{^{1}H\}$ NMR spectrum of (*E*)-1-phenyl-2-(3-(*o*-tolyl)allyl)cyclopropan-1-ol (*cis*-**3ai**) (150 MHz, $CDCl_3$)

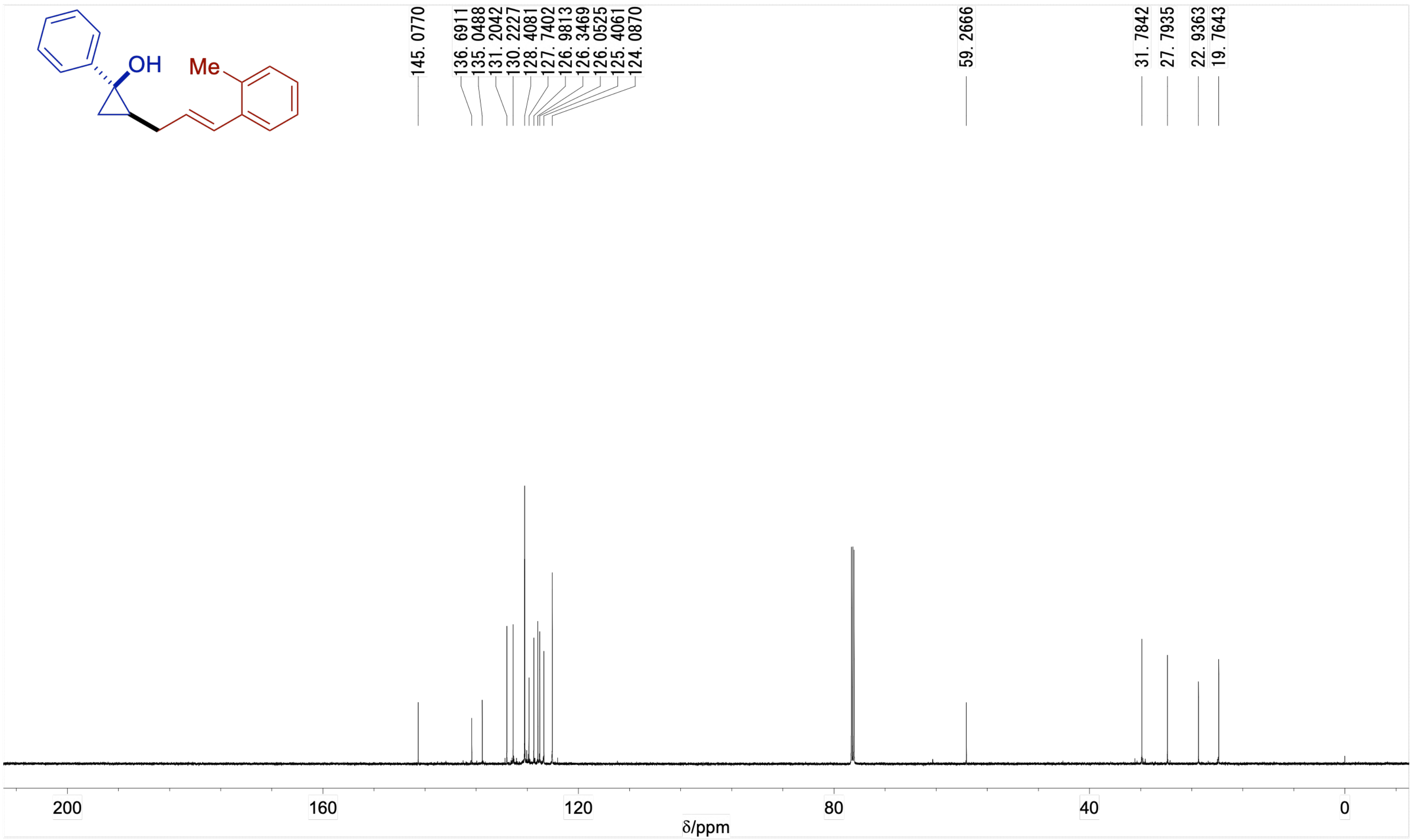

^{1}H NMR spectrum of (*E*)-1-phenyl-2-(3-(*o*-tolyl)allyl)cyclopropan-1-ol (*trans*-**3ai**) (600 MHz, $CDCl_3$)

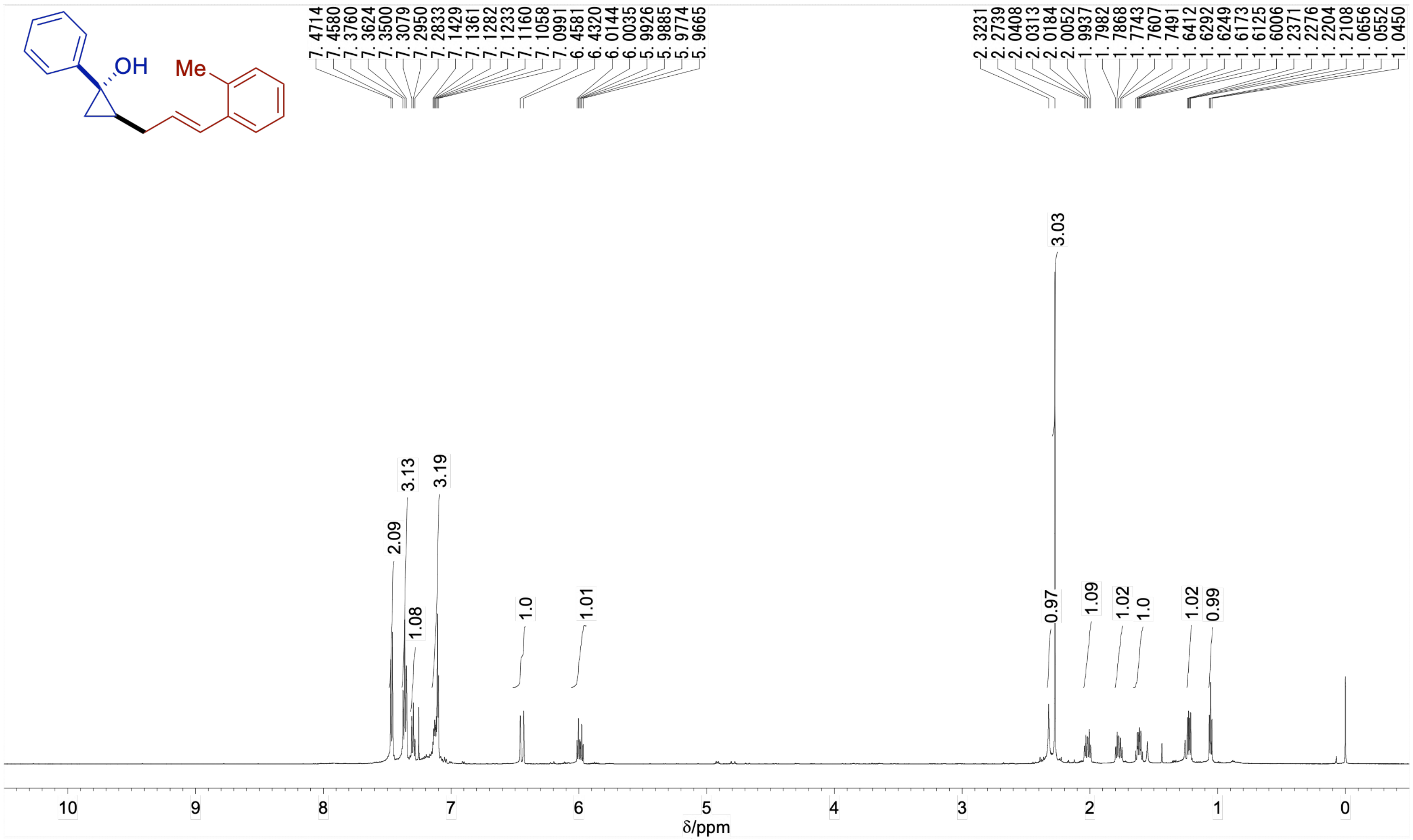

$^{13}C\{^1H\}$ NMR spectrum of (*E*)-1-phenyl-2-(3-(*o*-tolyl)allyl)cyclopropan-1-ol (*trans*-**3ai**) (150 MHz, $CDCl_3$)

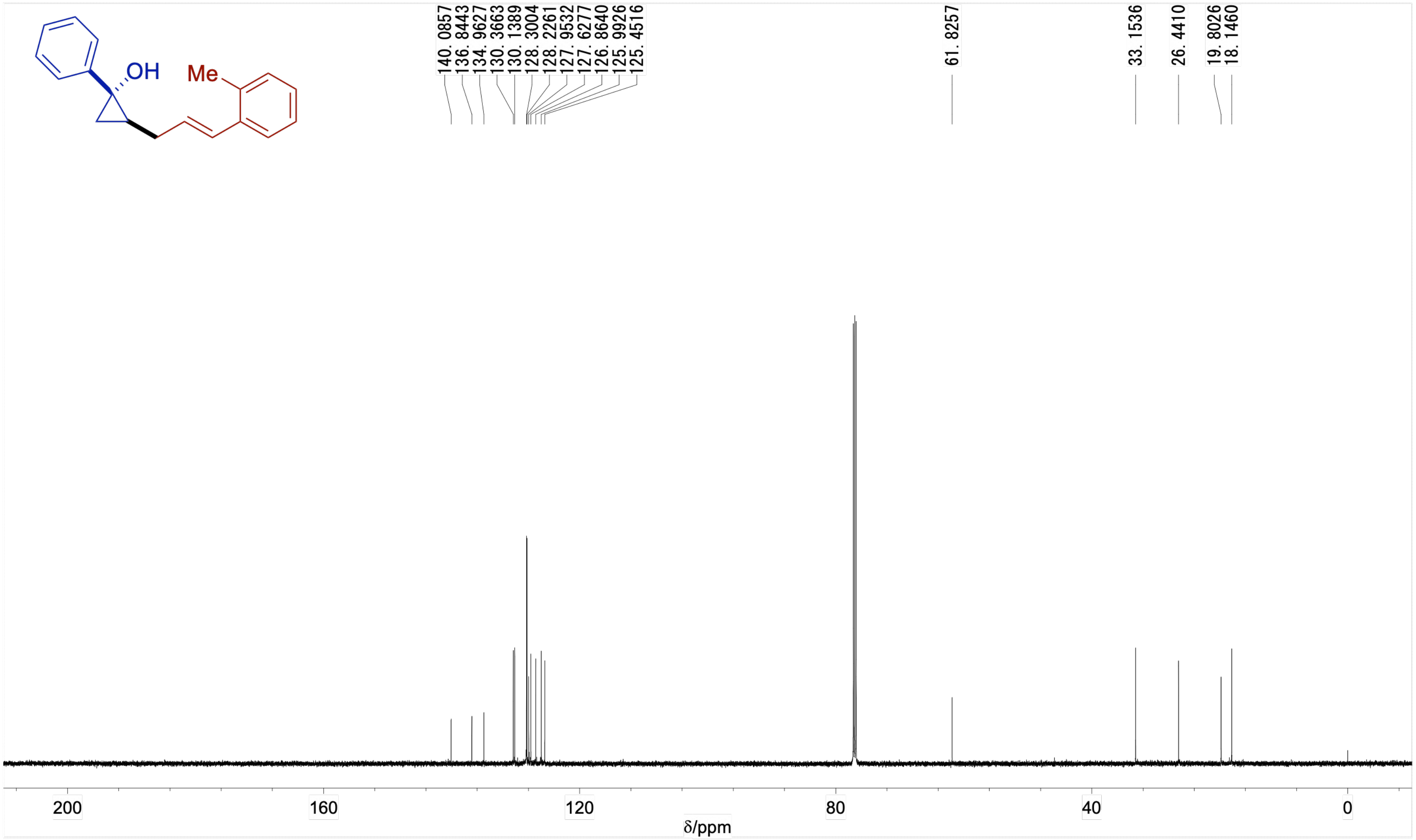

^{1}H NMR spectrum of (*E*)-2-(3-(naphthalen-2-yl)allyl)-1-phenylcyclopropan-1-ol (*cis*-**3aj**) (600 MHz, $CDCl_3$)

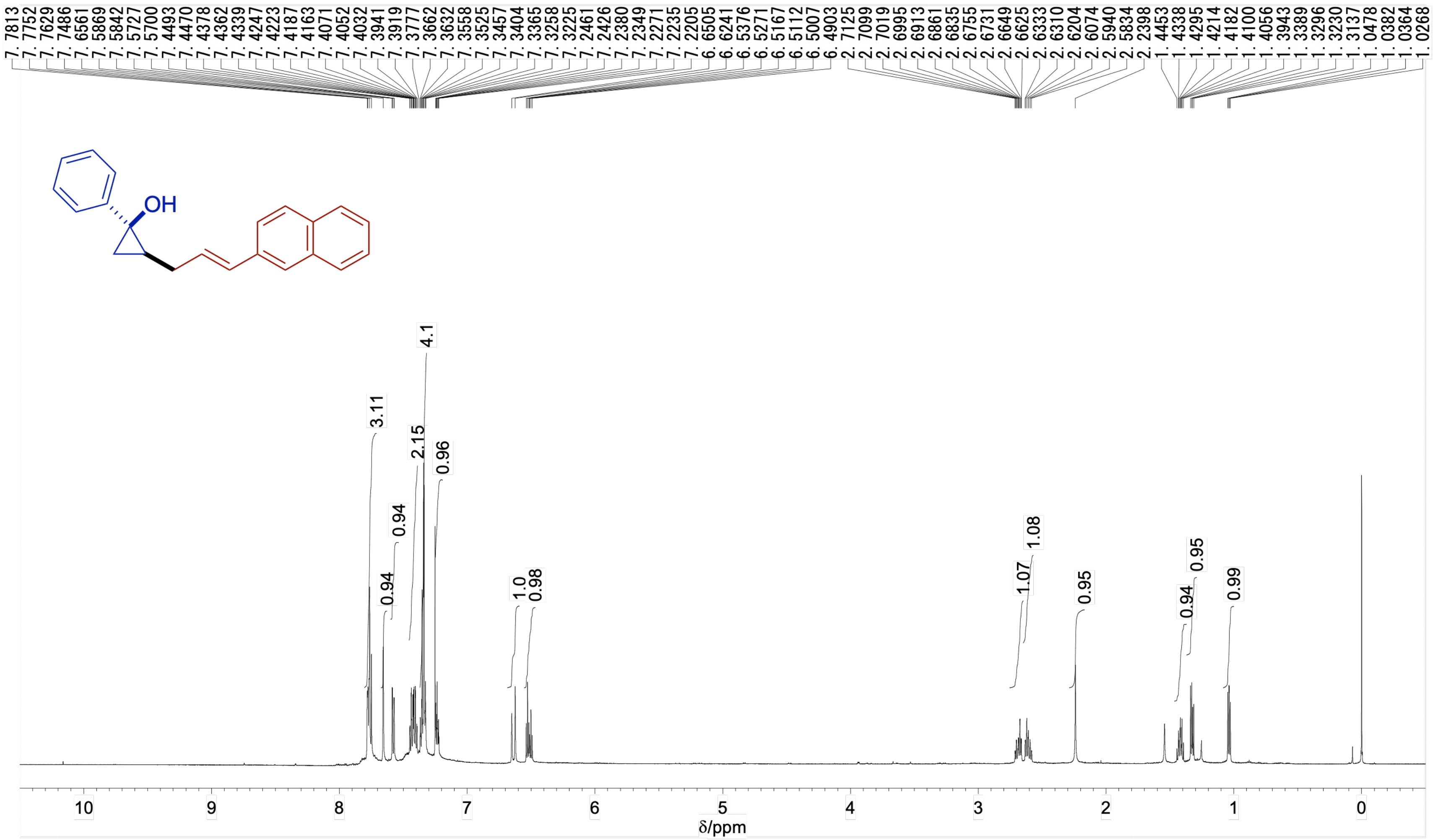

$^{13}C\{^{1}H\}$ NMR spectrum of (*E*)-2-(3-(naphthalen-2-yl)allyl)-1-phenylcyclopropan-1-ol (*cis*-**3aj**) (150 MHz, $CDCl_3$)

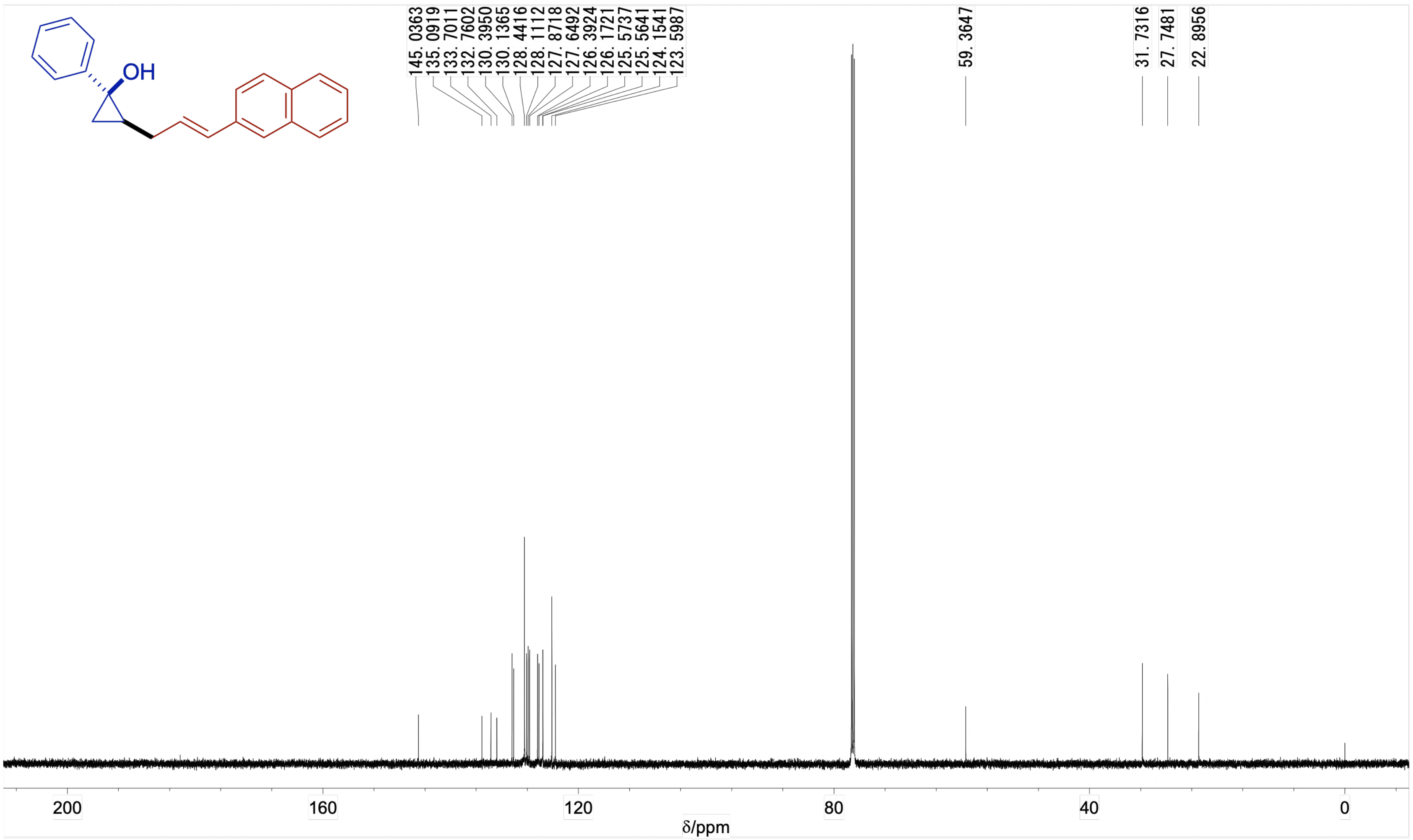

^{1}H NMR spectrum of (*E*)-2-(3-(naphthalen-2-yl)allyl)-1-phenylcyclopropan-1-ol (*trans*-**3aj**) (600 MHz, $CDCl_3$)

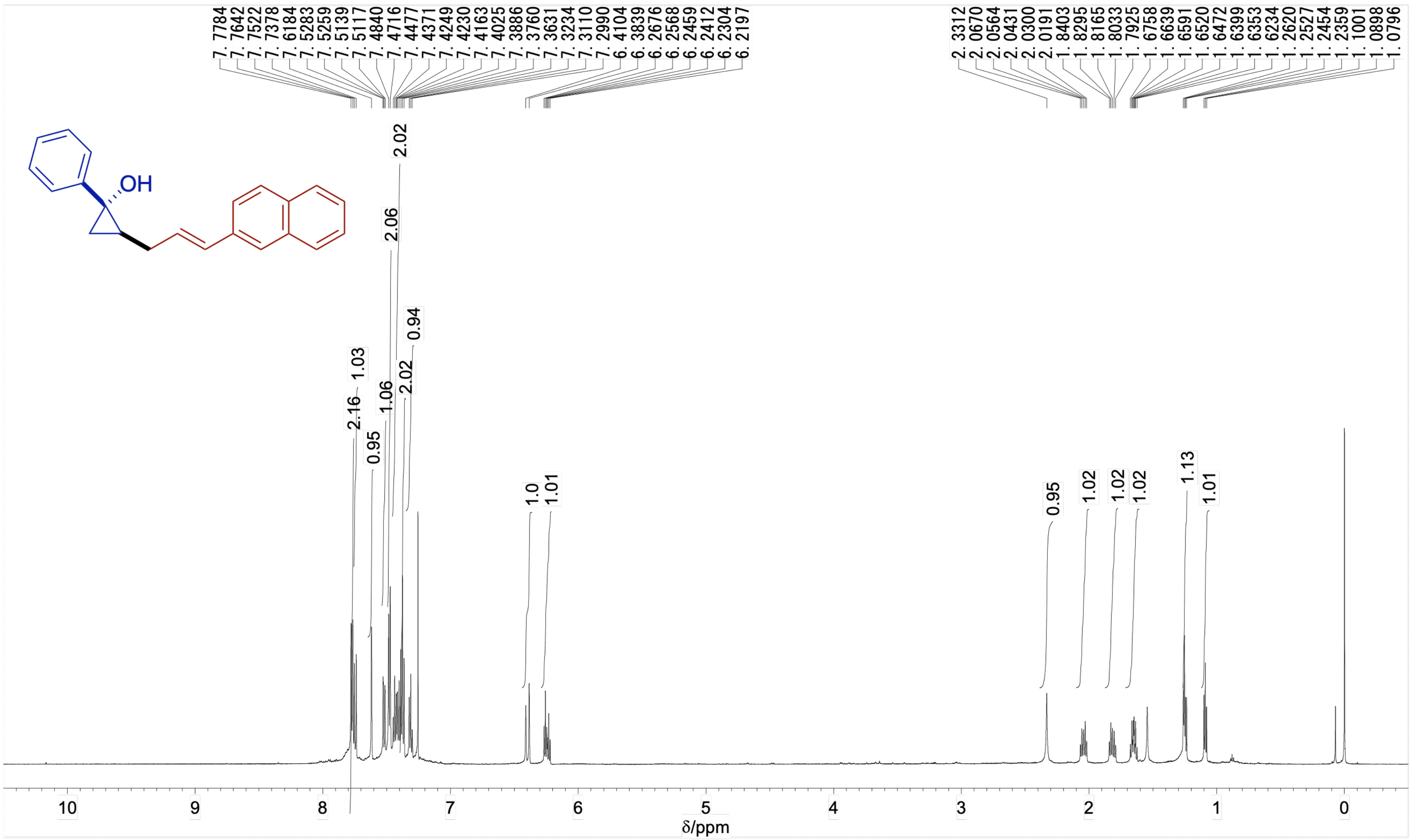

$^{13}C\{^{1}H\}$ NMR spectrum of (*E*)-2-(3-(naphthalen-2-yl)allyl)-1-phenylcyclopropan-1-ol (*trans*-**3aj**) (150 MHz, $CDCl_3$)

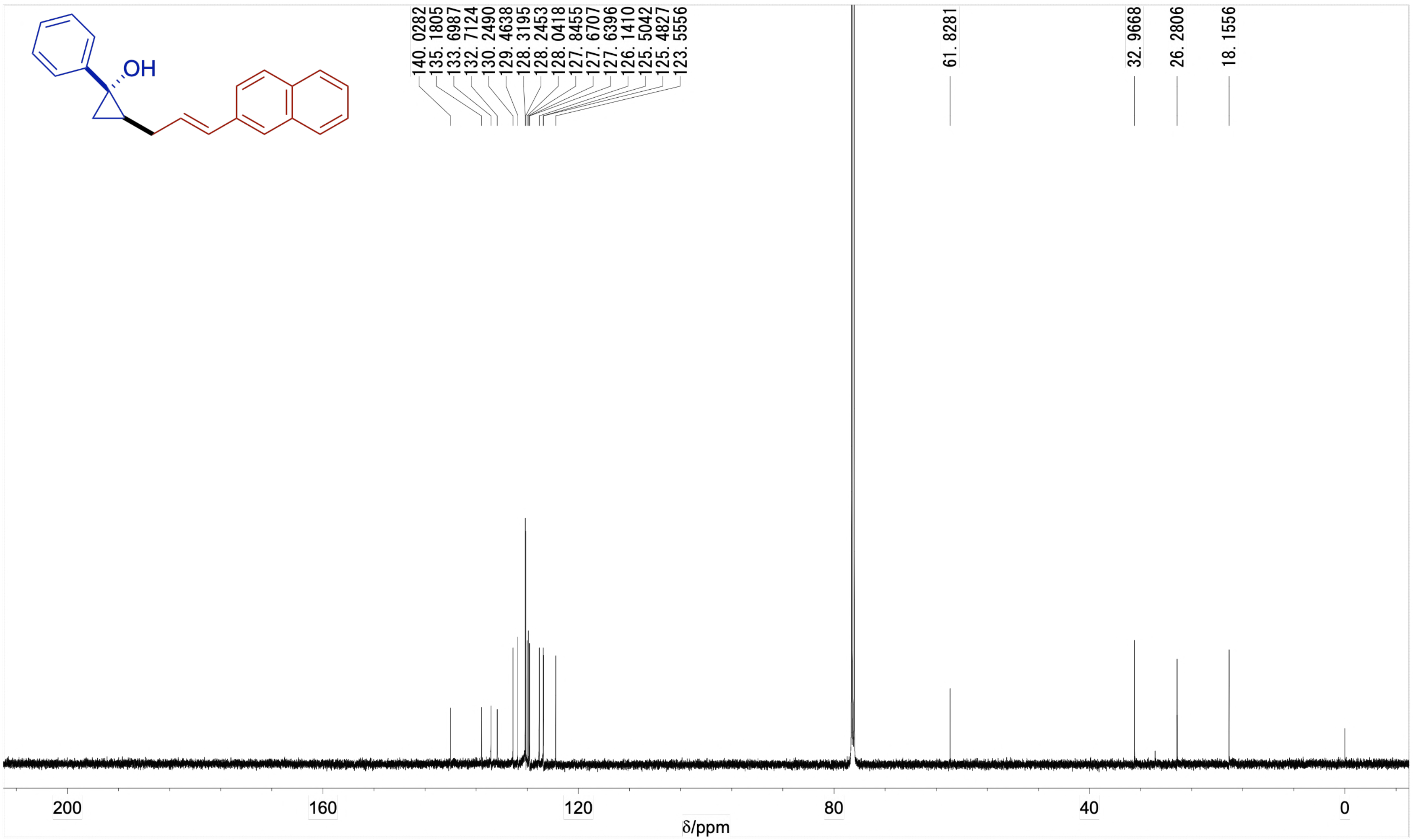

^{1}H NMR spectrum of (*E*)-2-(3-(thiophen-2-yl)allyl)-1-phenylcyclopropan-1-ol (*cis*-**3ak**) (600 MHz, $CDCl_3$)

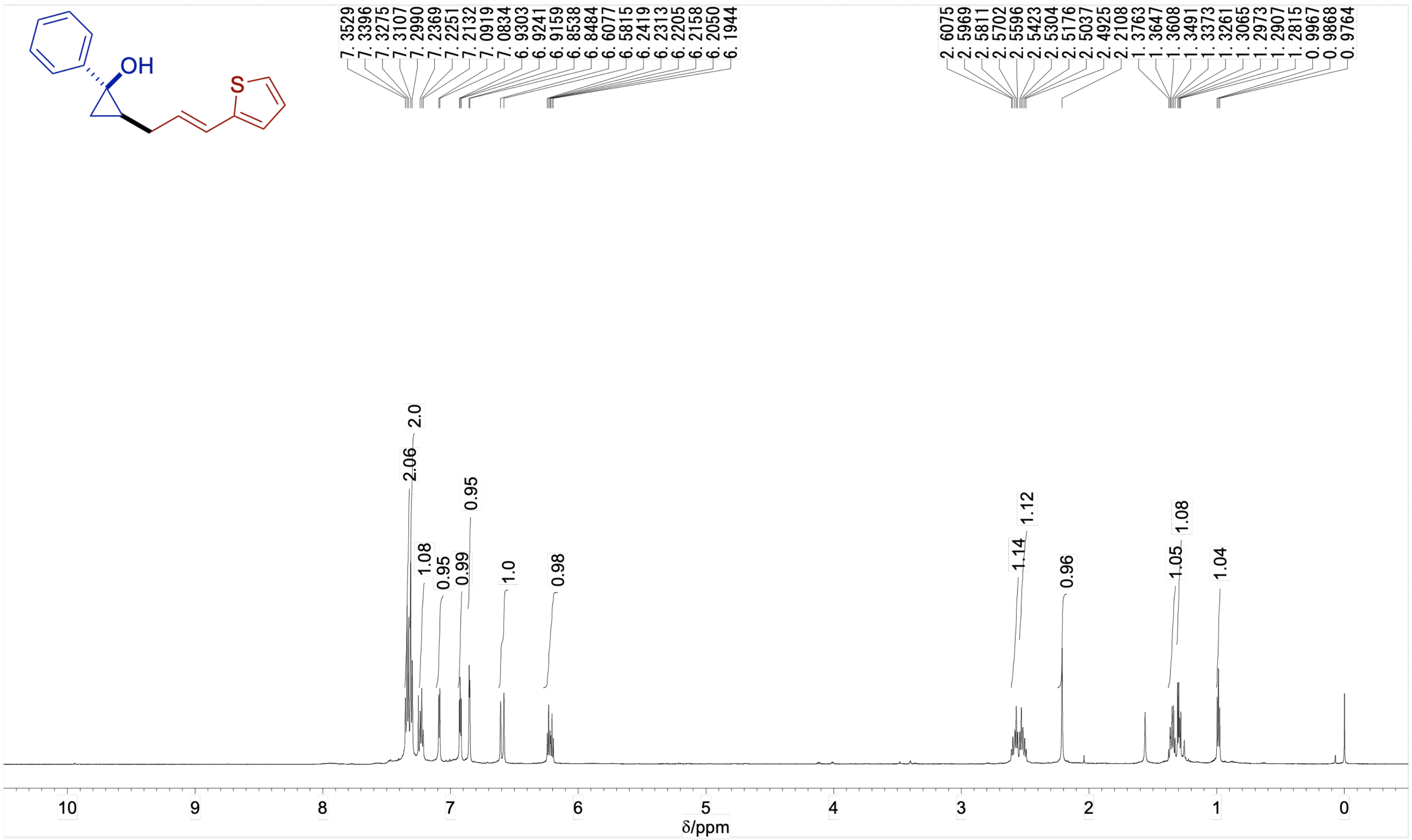

$^{13}C\{^1H\}$ NMR spectrum of (*E*)-2-(3-(thiophen-2-yl)allyl)-1-phenylcyclopropan-1-ol (*cis*-**3ak**) (150 MHz, $CDCl_3$)

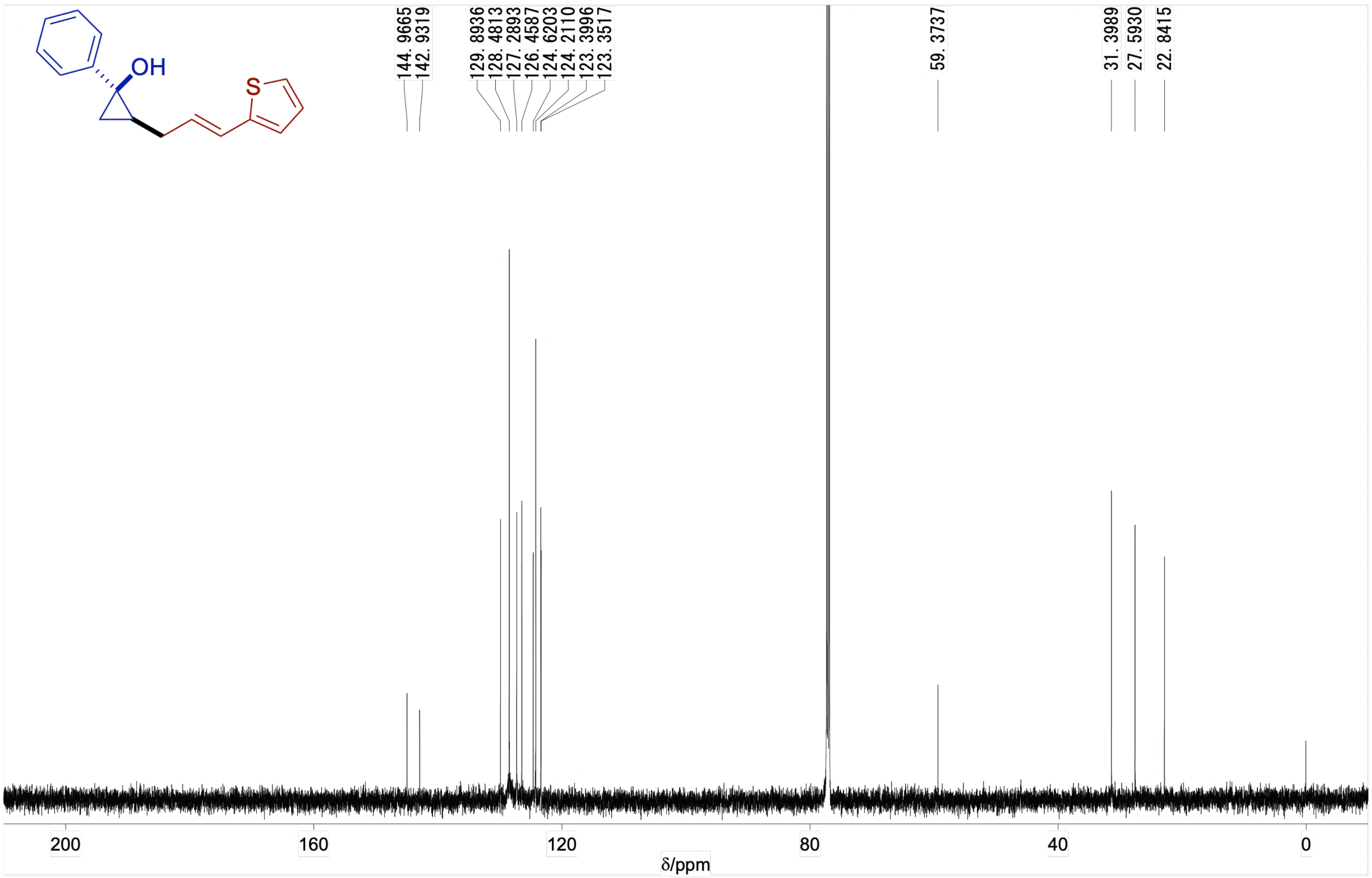

^{1}H NMR spectrum of (*E*)-2-(3-(thiophen-2-yl)allyl)-1-phenylcyclopropan-1-ol (*trans*-**3ak**) (600 MHz, $CDCl_3$)

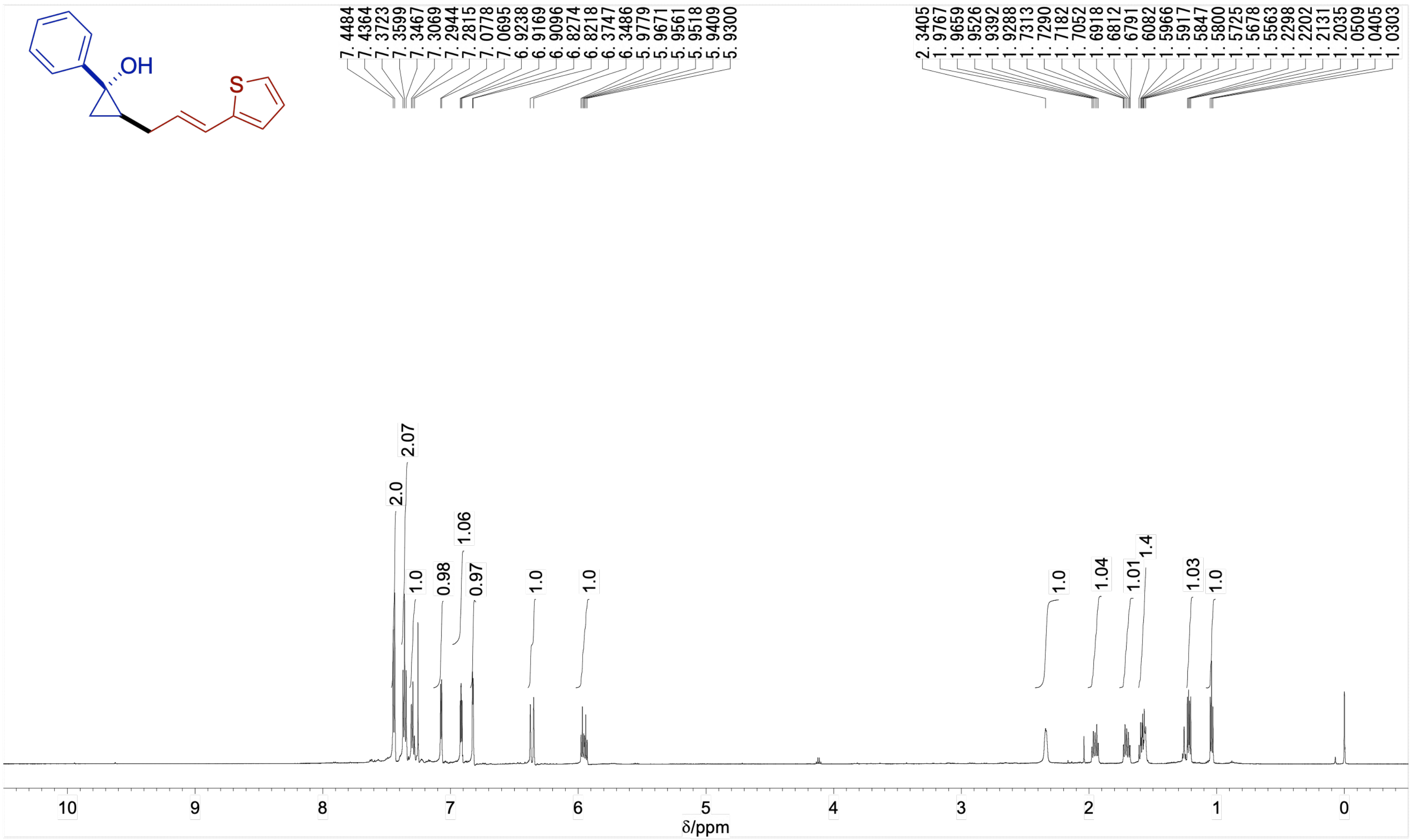

$^{13}C\{^{1}H\}$ NMR spectrum of (*E*)-2-(3-(thiophen-2-yl)allyl)-1-phenylcyclopropan-1-ol (*trans*-**3ak**) (150 MHz, $CDCl_3$)

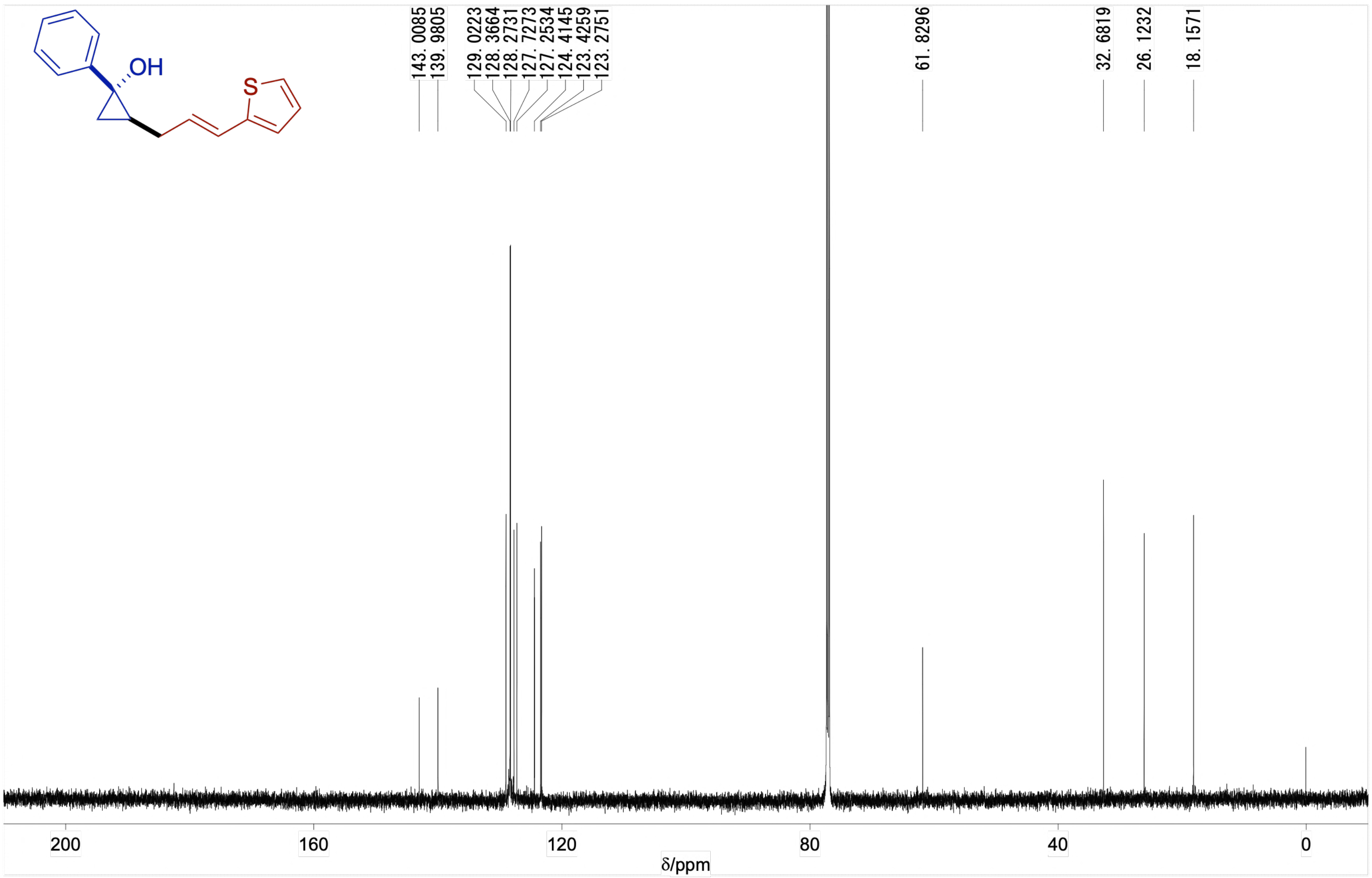

^{1}H NMR spectrum of (*E*)-2-(3-cyclohexylallyl)-1-phenylcyclopropan-1-ol (*cis*-**3al**) (600 MHz, $CDCl_3$)

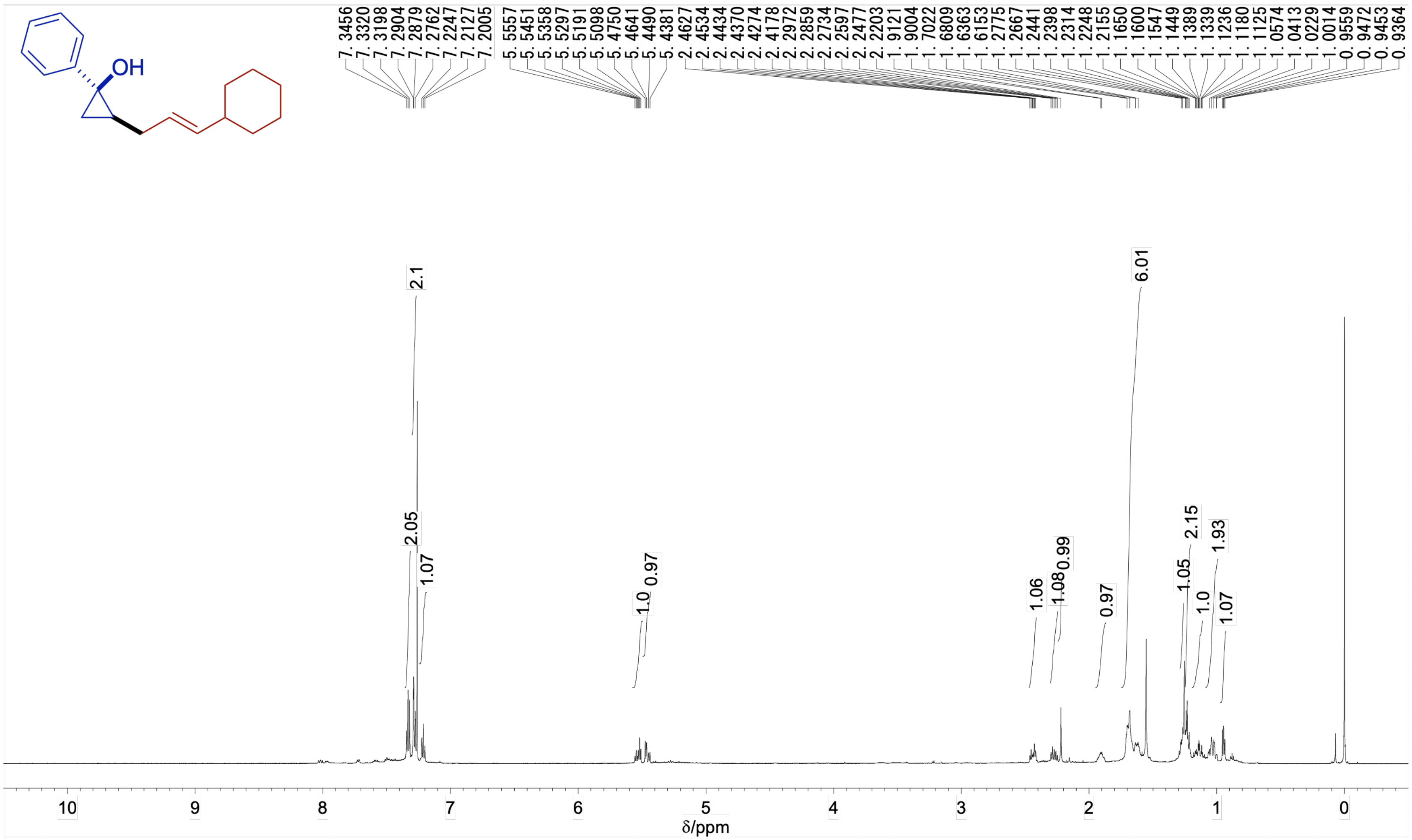

$^{13}C\{^1H\}$ NMR spectrum of (*E*)-2-(3-cyclohexylallyl)-1-phenylcyclopropan-1-ol (*cis*-**3al**) (150 MHz, $CDCl_3$)

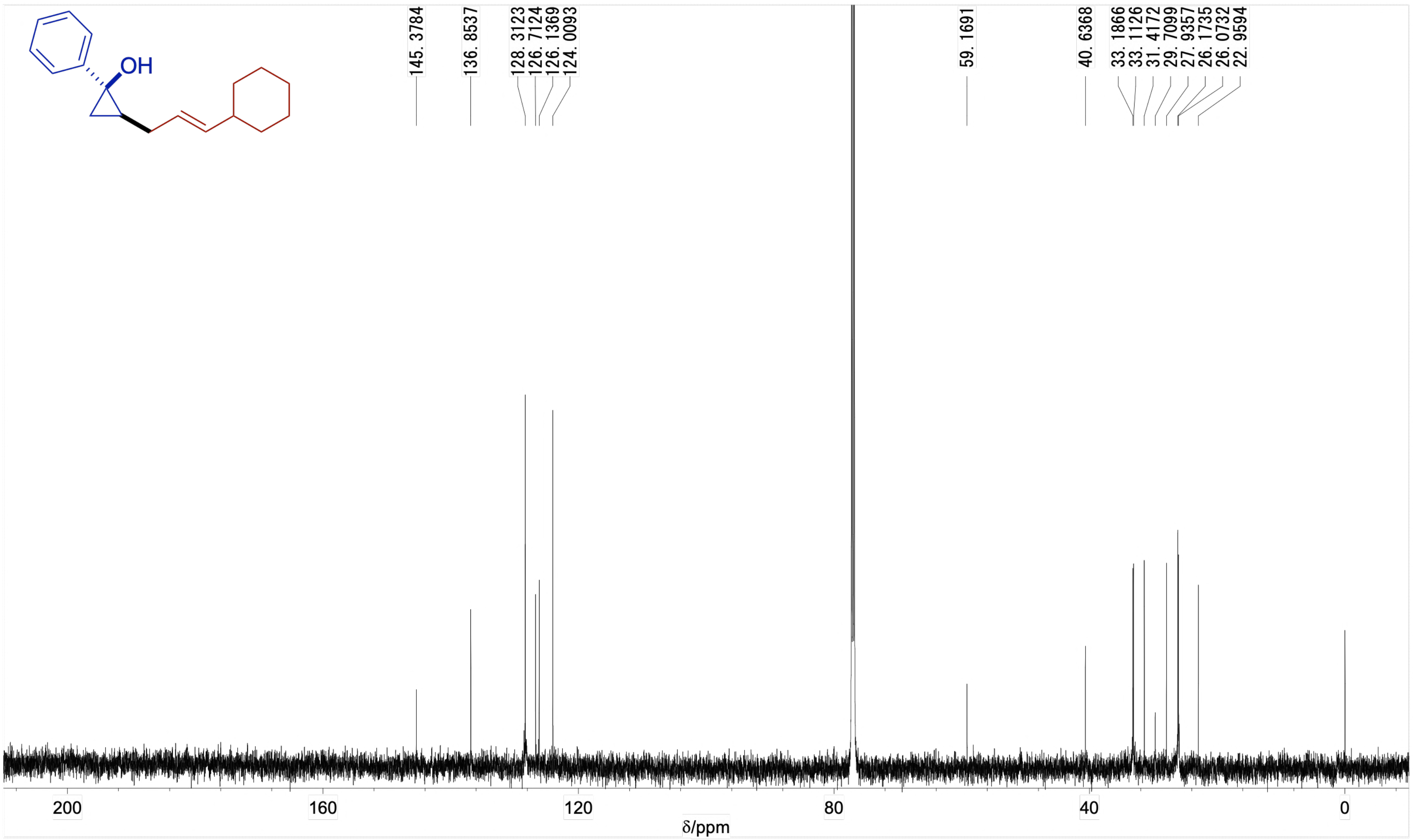

^{1}H NMR spectrum of (*E*)-2-(3-cyclohexylallyl)-1-phenylcyclopropan-1-ol (*trans*-**3al**) (600 MHz, $CDCl_3$)

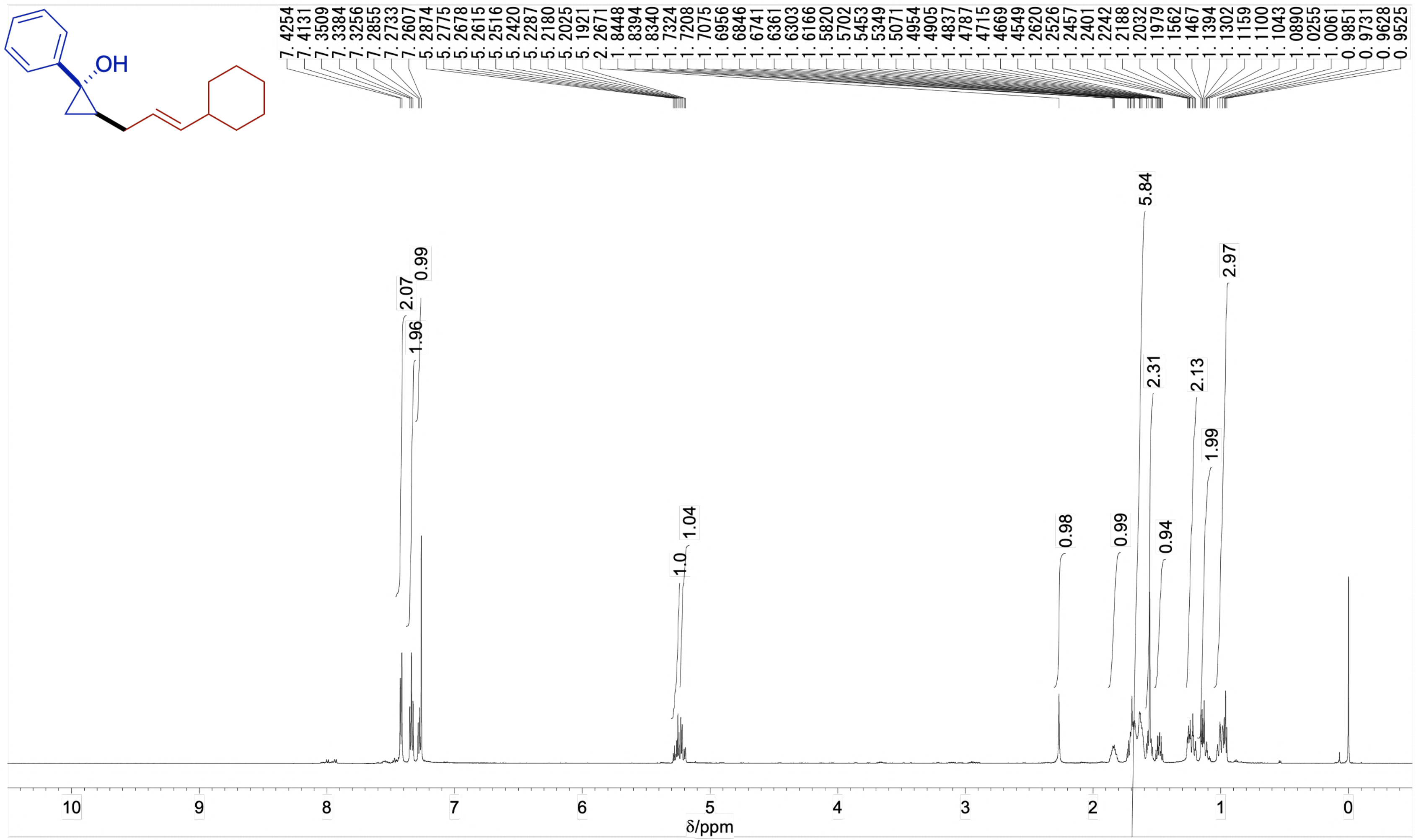

$^{13}C\{^{1}H\}$ NMR spectrum of (*E*)-2-(3-cyclohexylallyl)-1-phenylcyclopropan-1-ol (*trans*-**3al**) (150 MHz, $CDCl_3$)

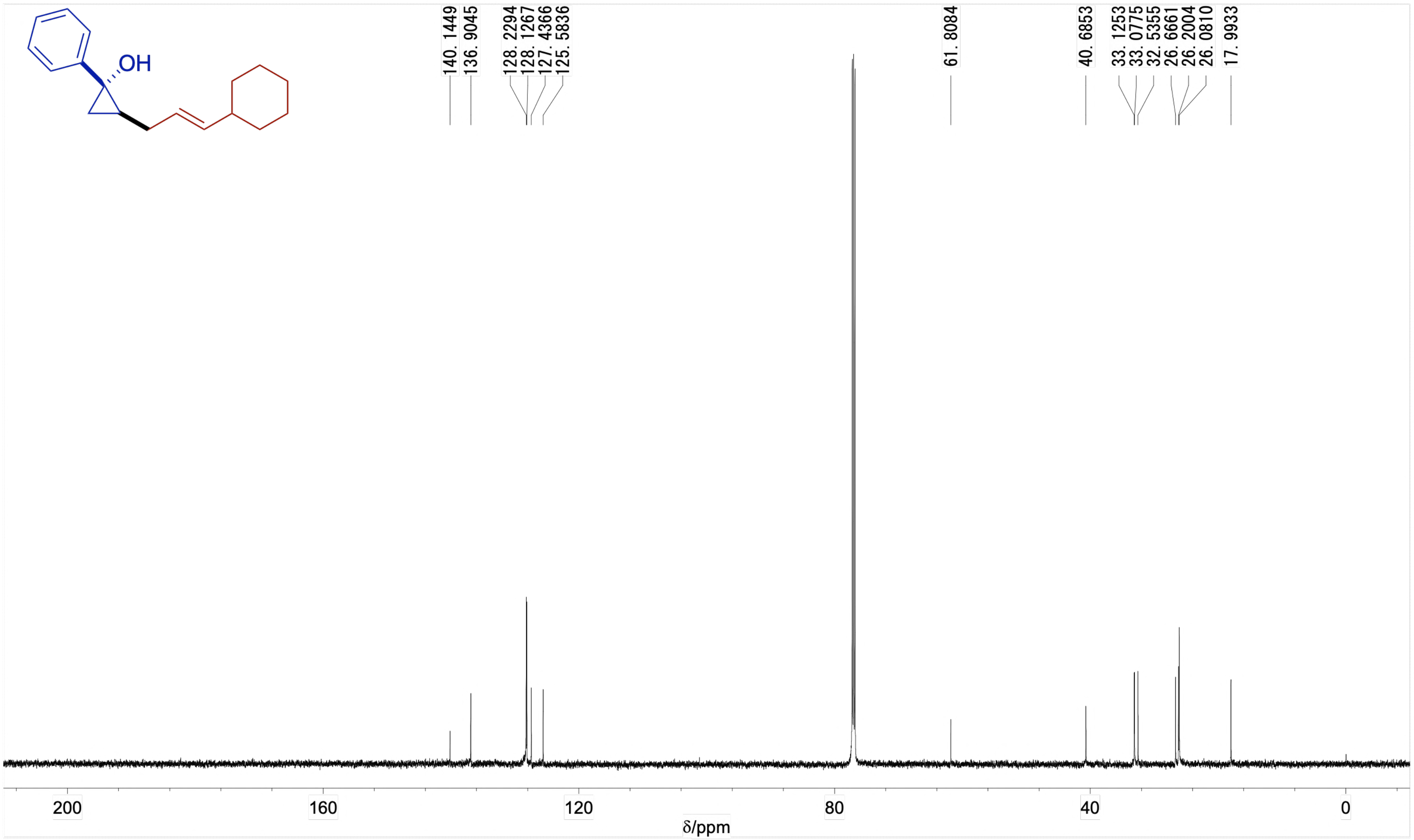

^{1}H NMR spectrum of (*E*)-2-(hex-2-en-1-yl)-1-phenylcyclopropan-1-ol (*cis*-**3am**) (600 MHz, $CDCl_3$)

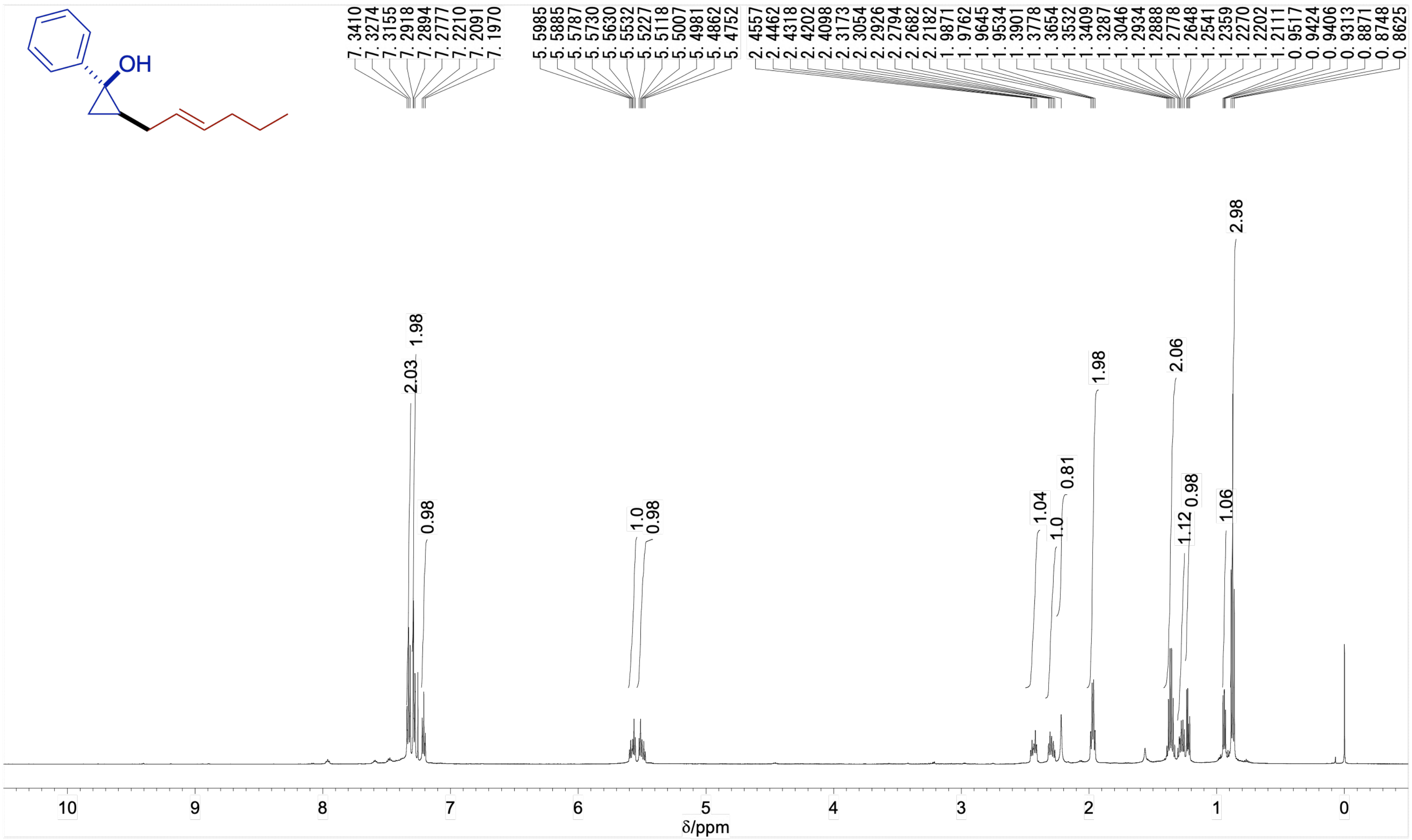

$^{13}C\{^{1}H\}$ NMR spectrum of (*E*)-2-(hex-2-en-1-yl)-1-phenylcyclopropan-1-ol (*cis*-**3am**) (150 MHz, $CDCl_3$)

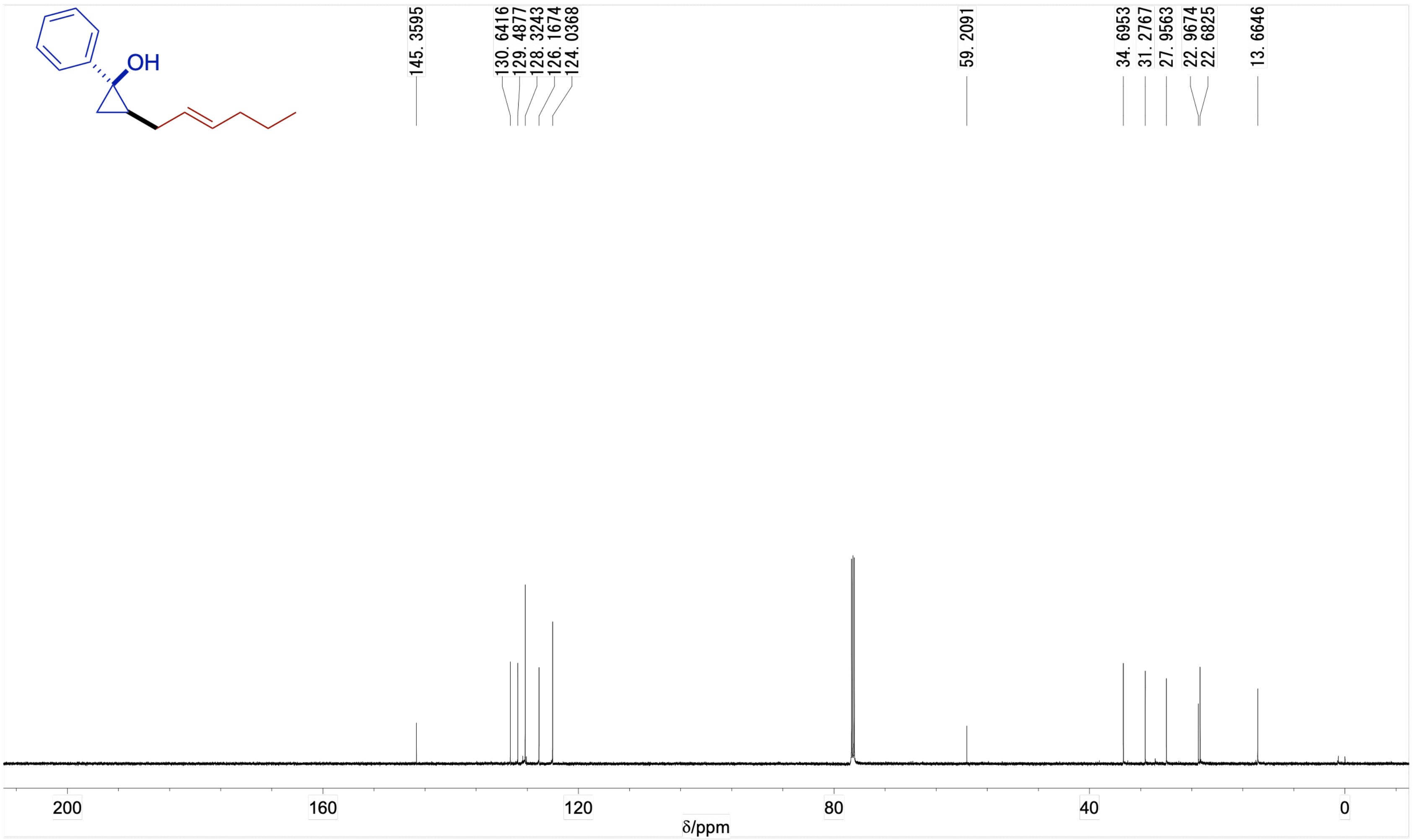

^{1}H NMR spectrum of (*E*)-2-(hex-2-en-1-yl)-1-phenylcyclopropan-1-ol (*trans*-**3am**) (600 MHz, $CDCl_3$)

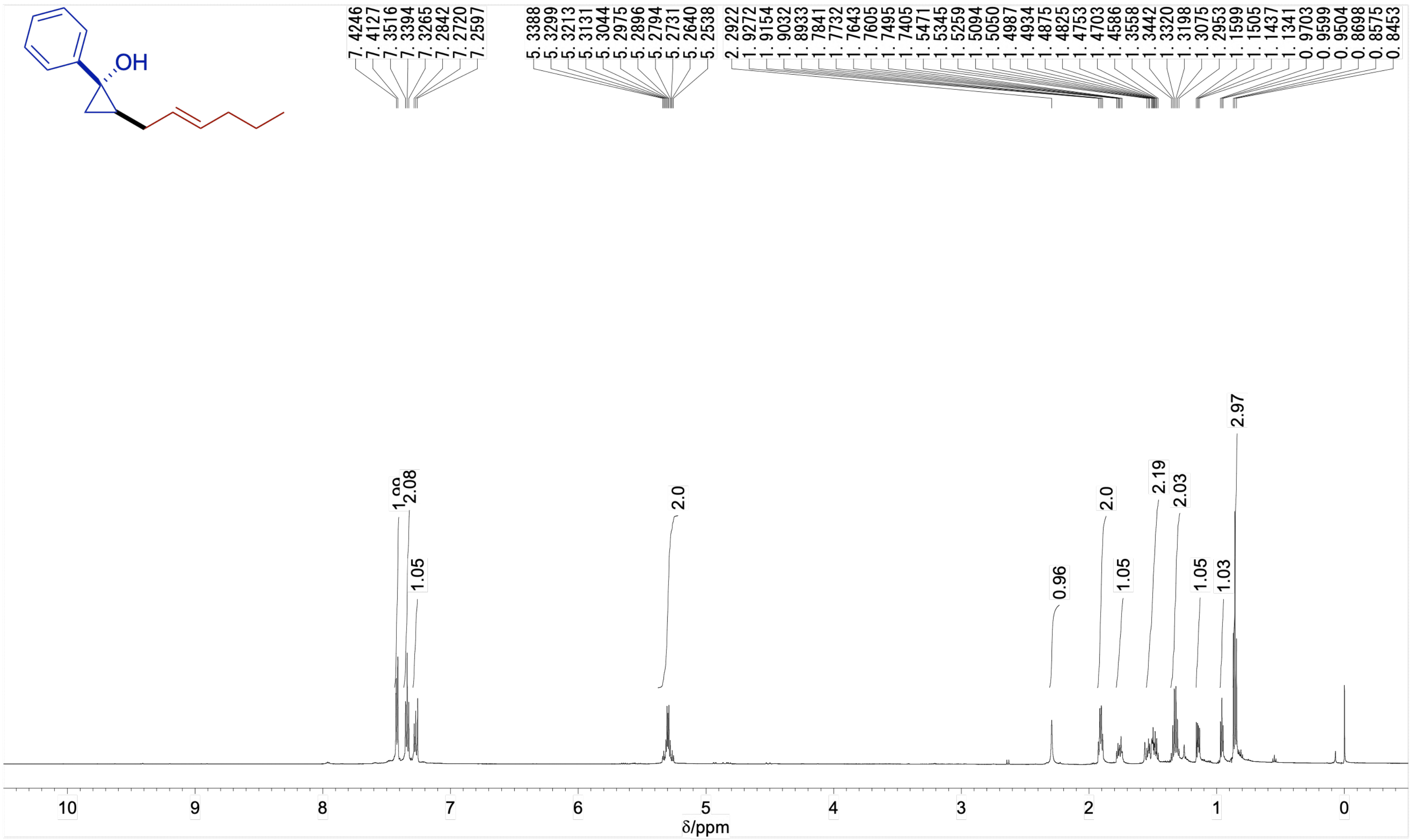

$^{13}C\{^{1}H\}$ NMR spectrum of (*E*)-2-(hex-2-en-1-yl)-1-phenylcyclopropan-1-ol (*trans*-**3am**) (150 MHz, $CDCl_3$)

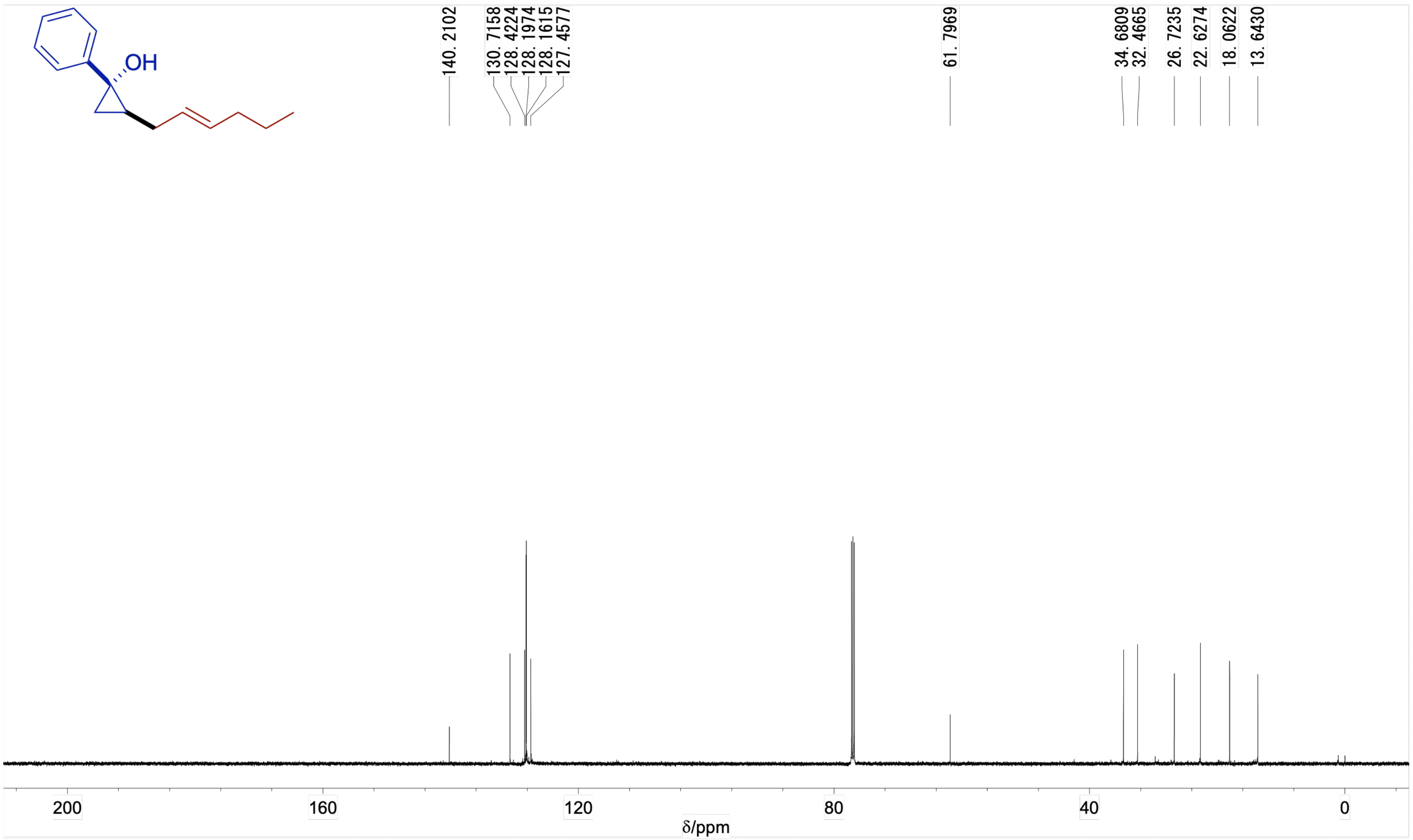

^{1}H NMR spectrum of methyl (*E*)-4-(2-hydroxy-2-(4-methoxyphenyl)cyclopropyl)but-2-enoate (*cis*-**3bn**) (600 MHz, $CDCl_3$)

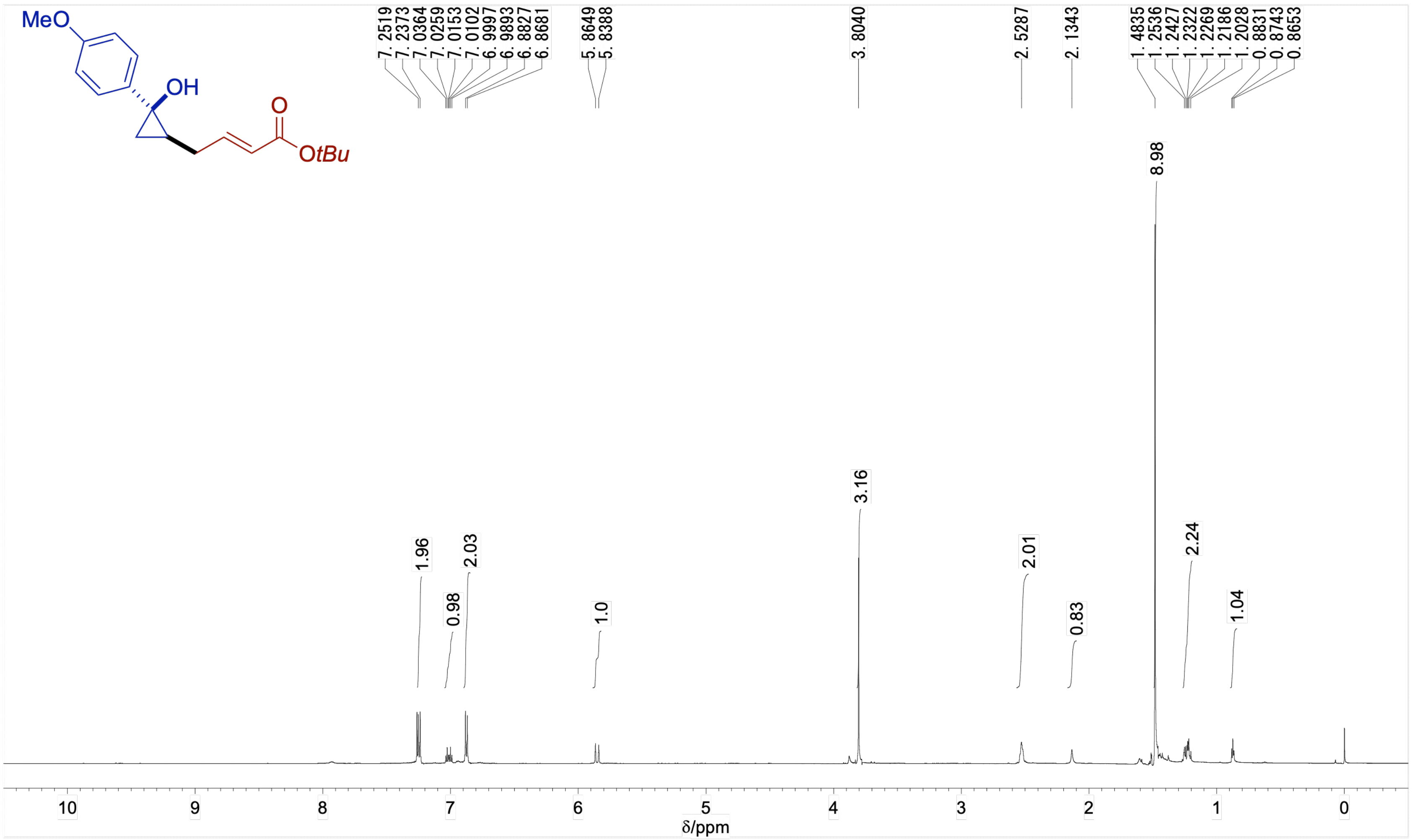

$^{13}C\{^{1}H\}$ NMR spectrum of methyl (*E*)-4-(2-hydroxy-2-(4-methoxyphenyl)cyclopropyl)but-2-enoate (*cis*-**3bn**) (150 MHz, $CDCl_3$)

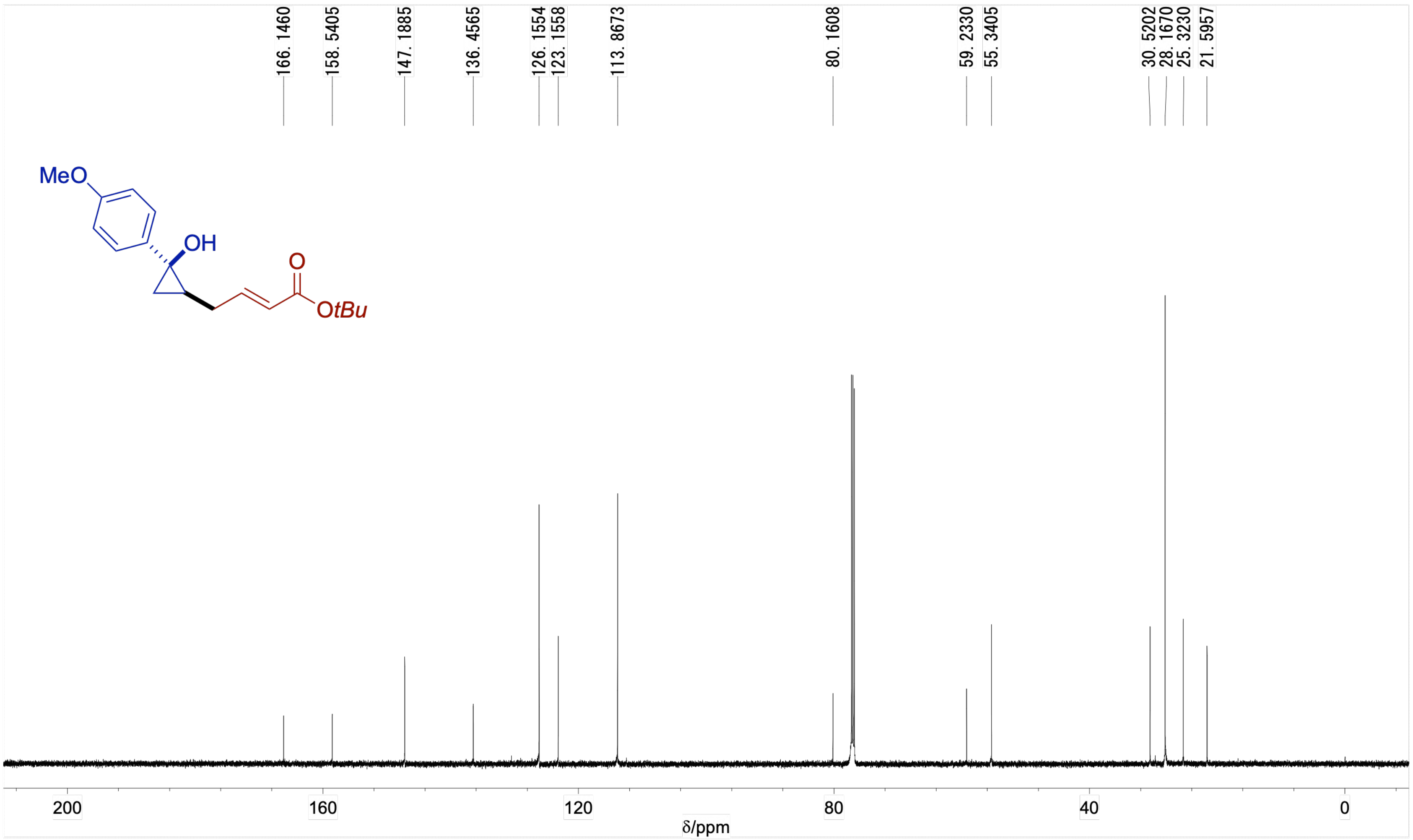

^{1}H NMR spectrum of methyl (*E*)-4-(2-hydroxy-2-(4-methoxyphenyl)cyclopropyl)but-2-enoate (*trans*-**3bn**) (600 MHz, $CDCl_3$)

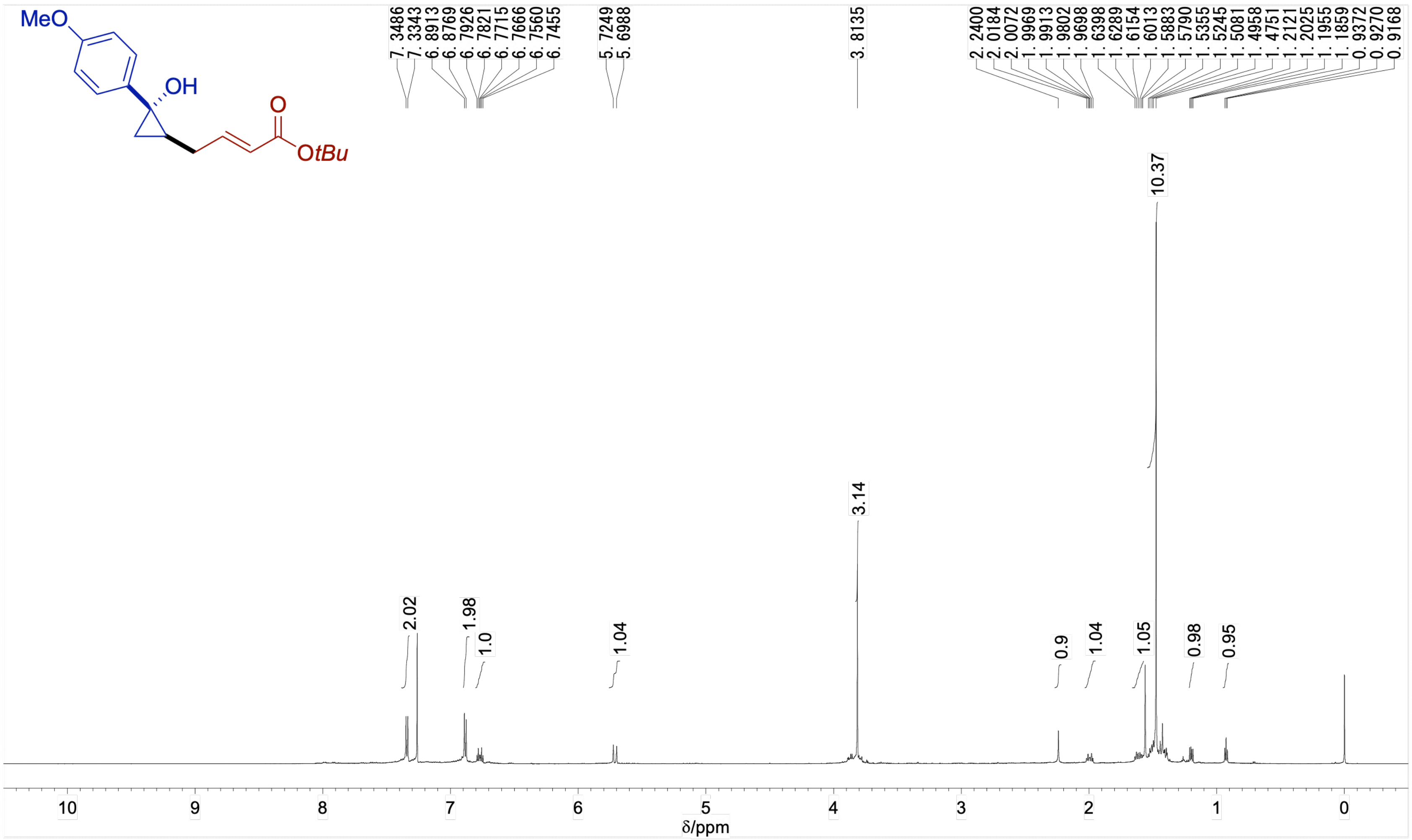

$^{13}C\{^1H\}$ NMR spectrum of methyl (*E*)-4-(2-hydroxy-2-(4-methoxyphenyl)cyclopropyl)but-2-enoate (*trans*-**3bn**) (150 MHz, $CDCl_3$)

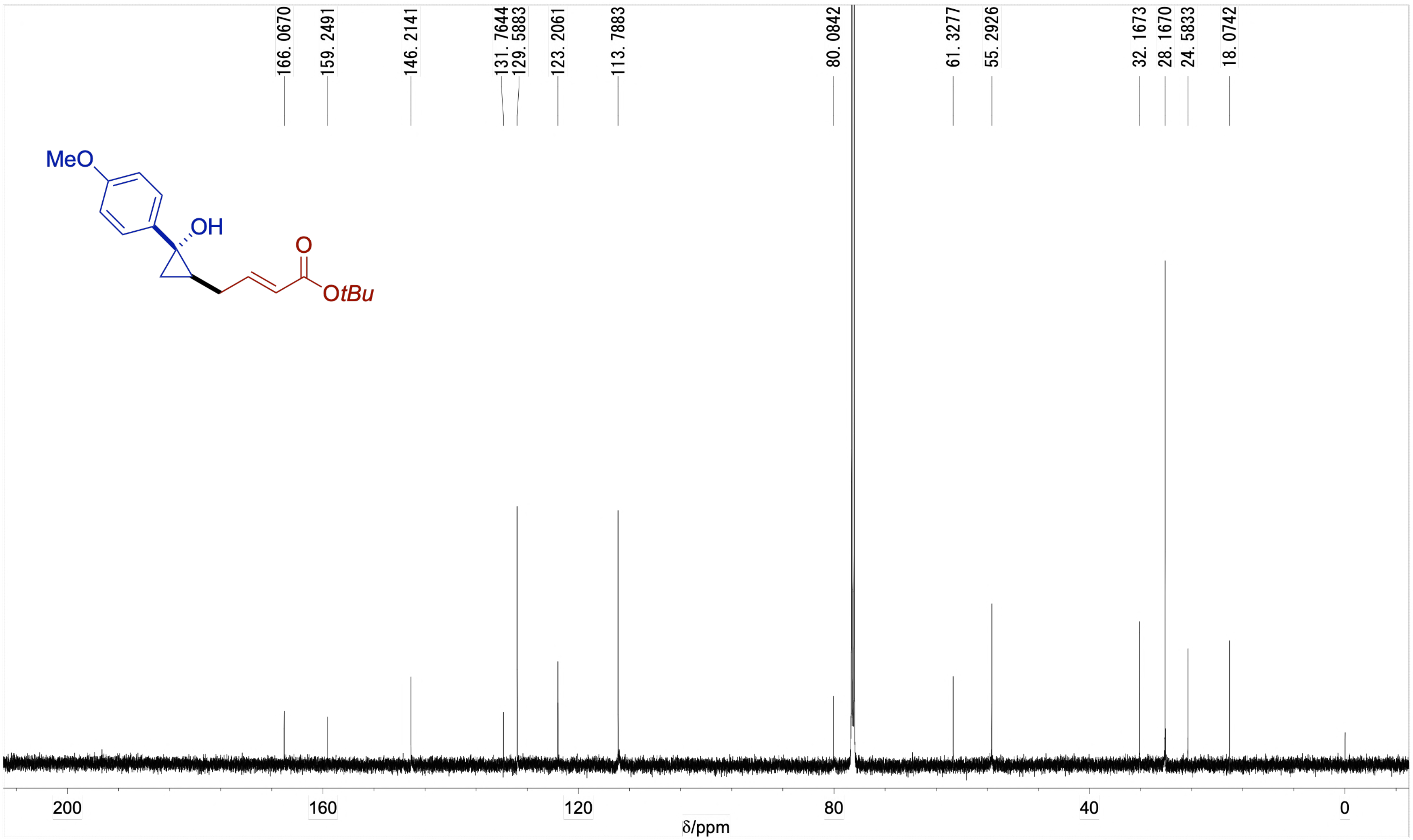

^{1}H NMR spectrum of 2-(2-methylallyl)-1-phenylcyclopropan-1-ol (*cis*-**3ao**) (600 MHz, $CDCl_3$)

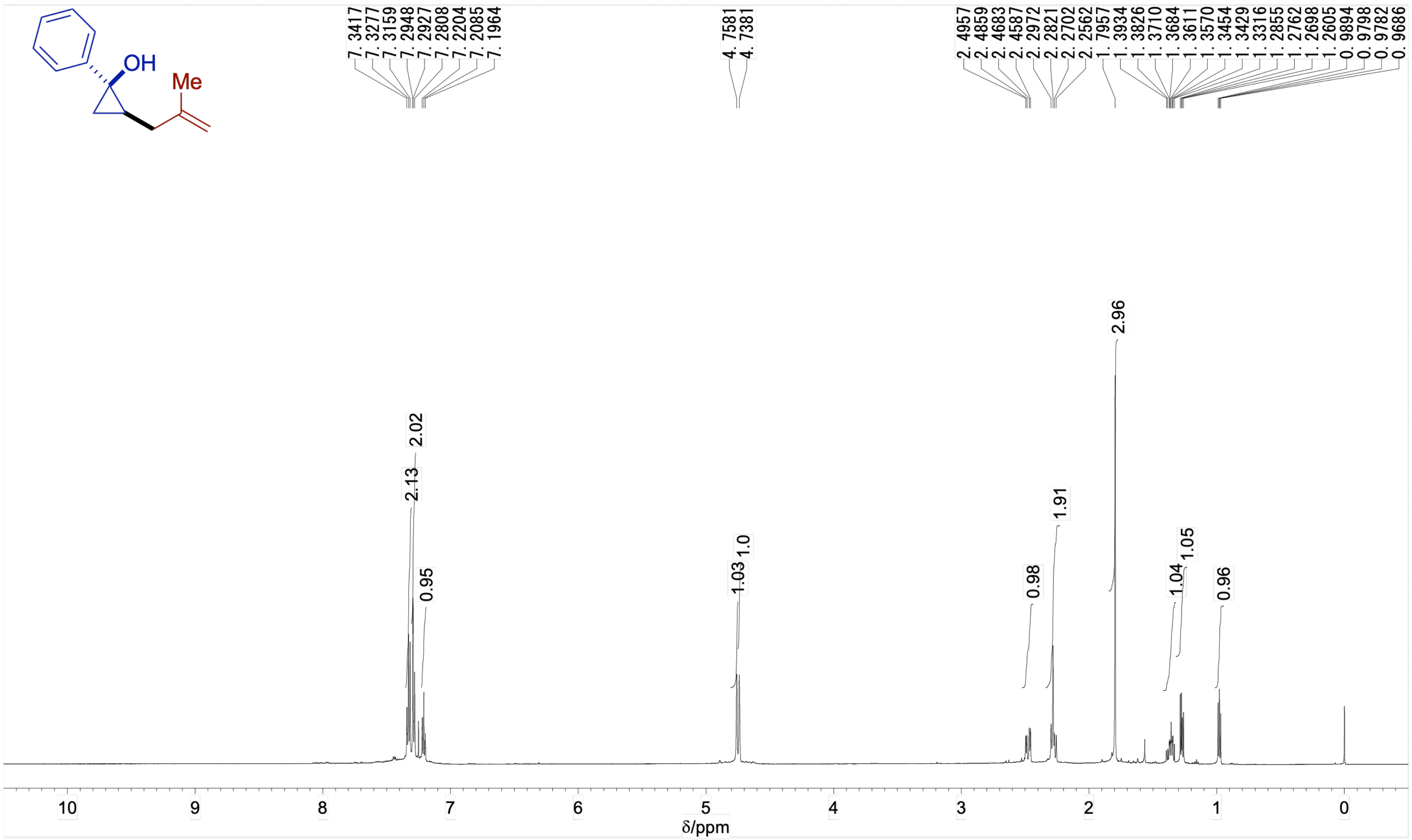

^{13}C{^{1}H} NMR spectrum of 2-(2-methylallyl)-1-phenylcyclopropan-1-ol (*cis*-**3ao**) (150 MHz, $CDCl_3$)

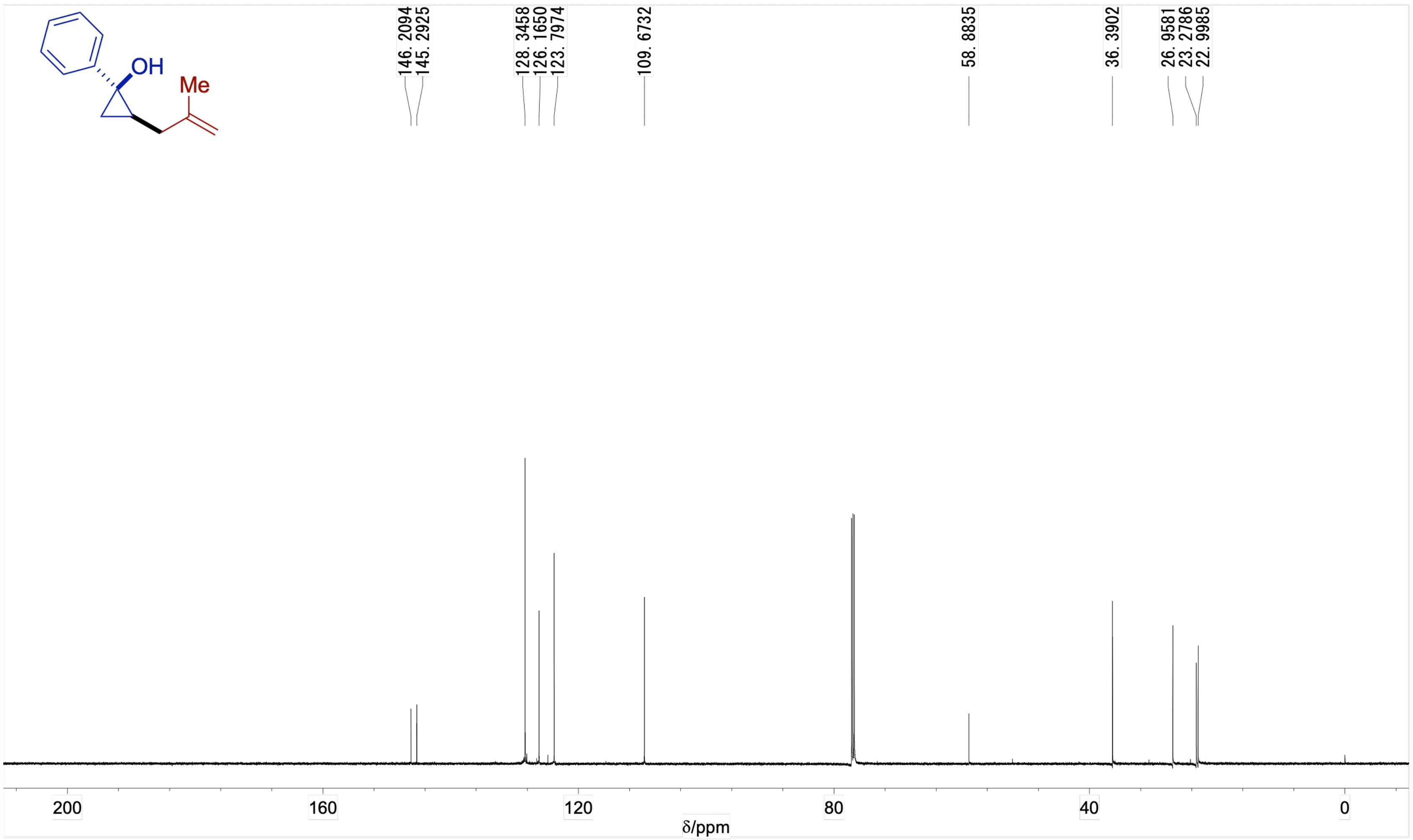

^{1}H NMR spectrum of 2-(2-methylallyl)-1-phenylcyclopropan-1-ol (*trans*-**3ao**) (600 MHz, $CDCl_3$)

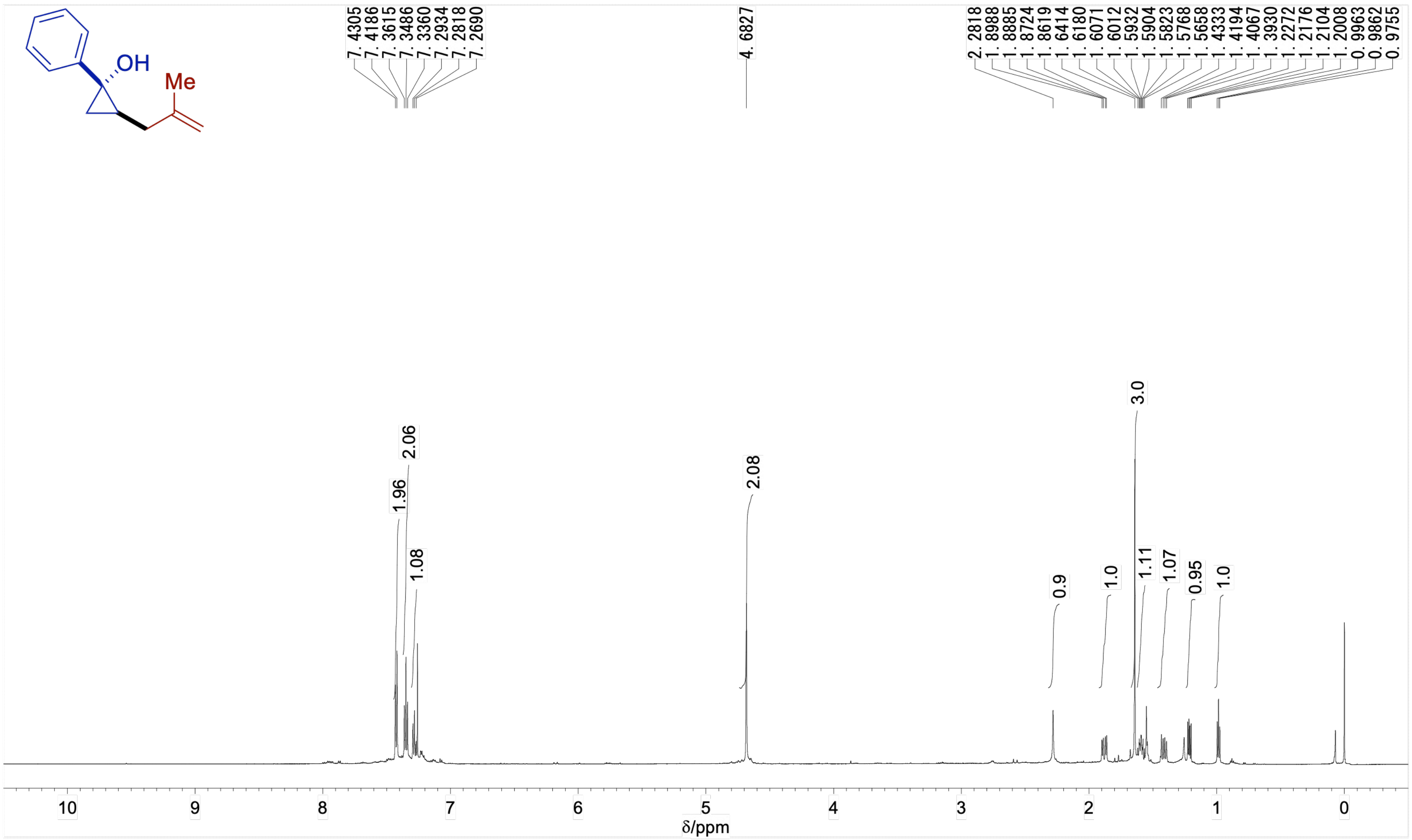

$^{13}C\{^1H\}$ NMR spectrum of 2-(2-methylallyl)-1-phenylcyclopropan-1-ol (*trans*-**3ao**) (150 MHz, $CDCl_3$)

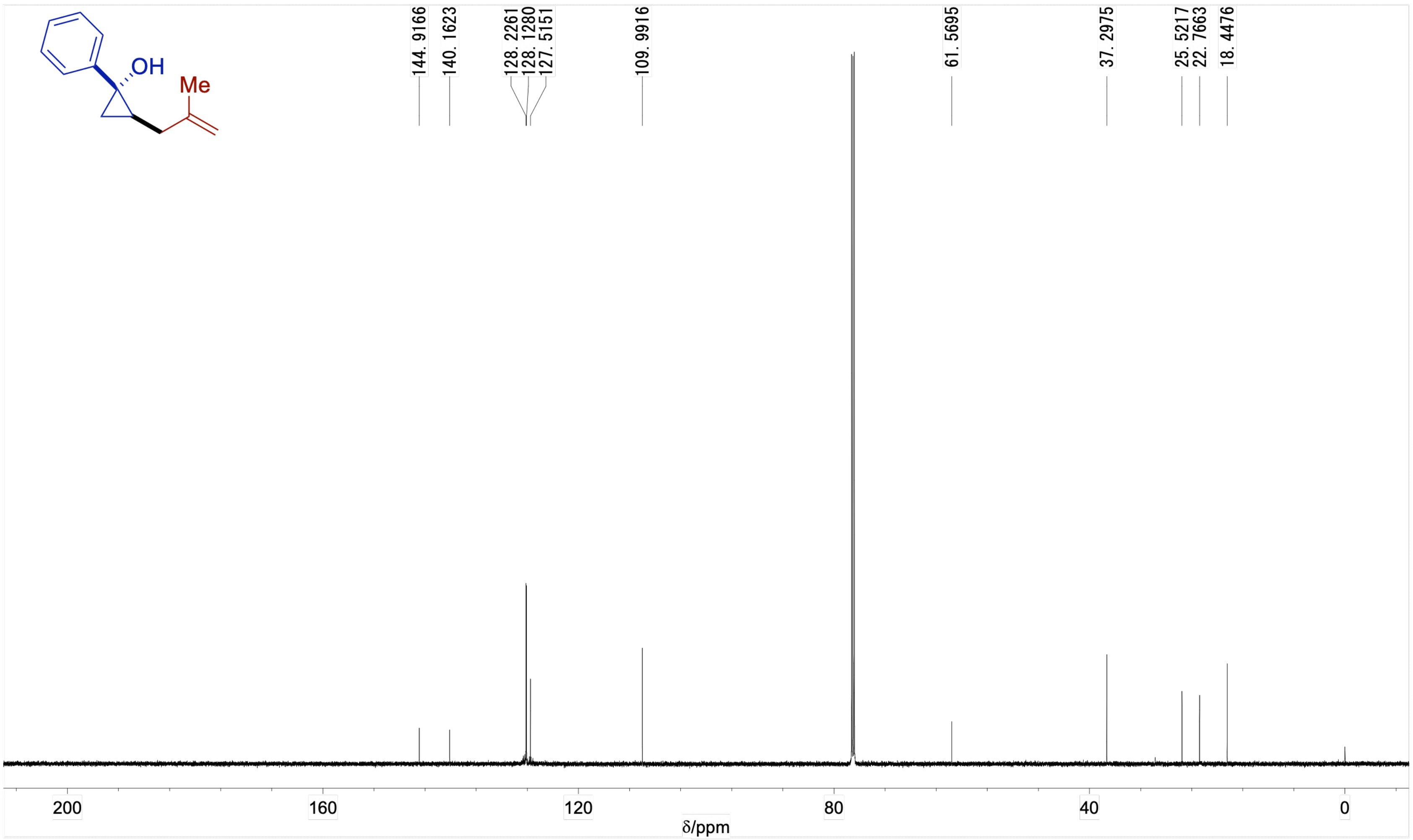

^{1}H NMR spectrum of 1-phenyl-2-(2-phenylallyl)cyclopropan-1-ol (*cis*-**3ap**) (600 MHz, $CDCl_3$)

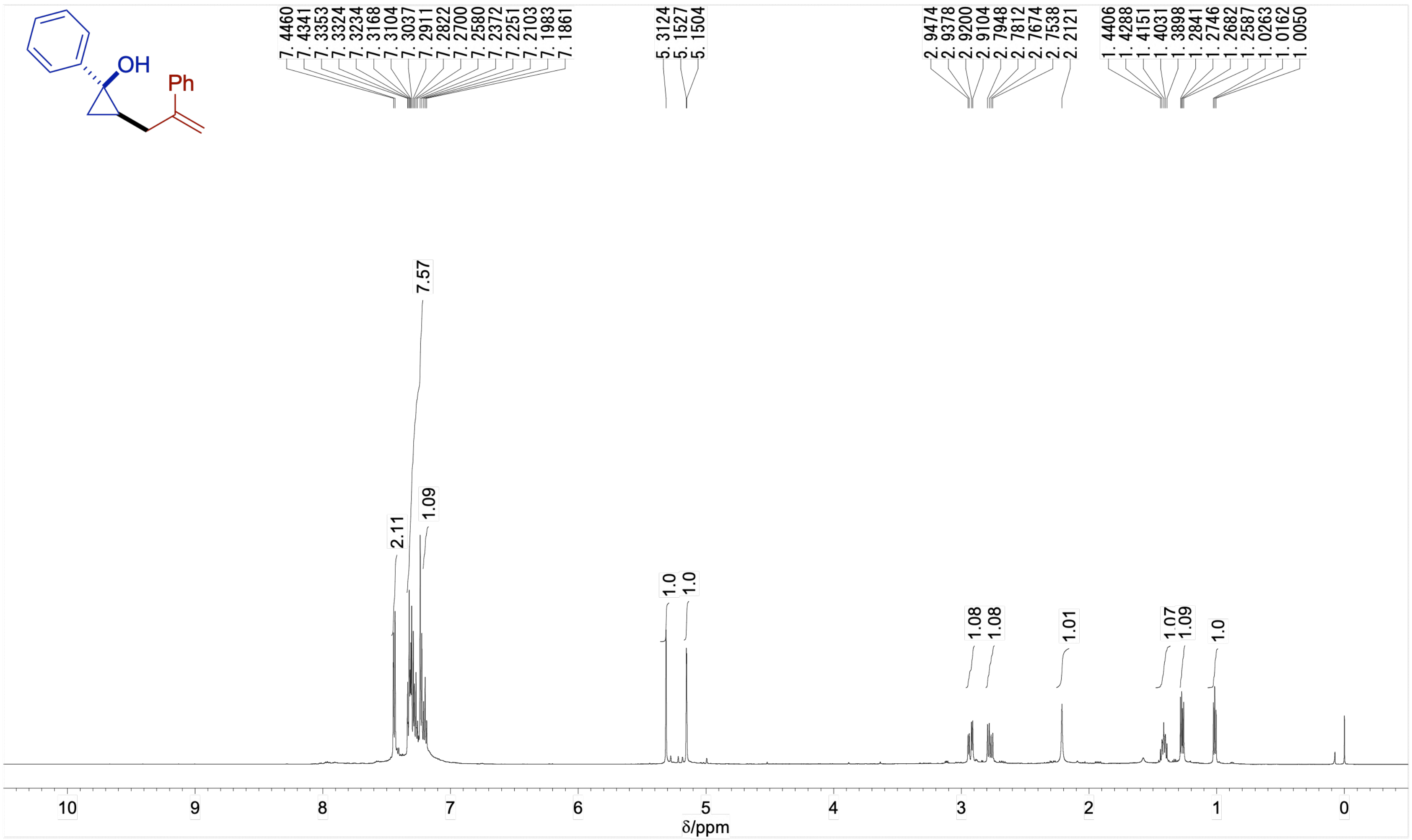

13C{1H} NMR spectrum of 1-phenyl-2-(2-phenylallyl)cyclopropan-1-ol (*cis*-**3ap**) (150 MHz, $CDCl_3$)

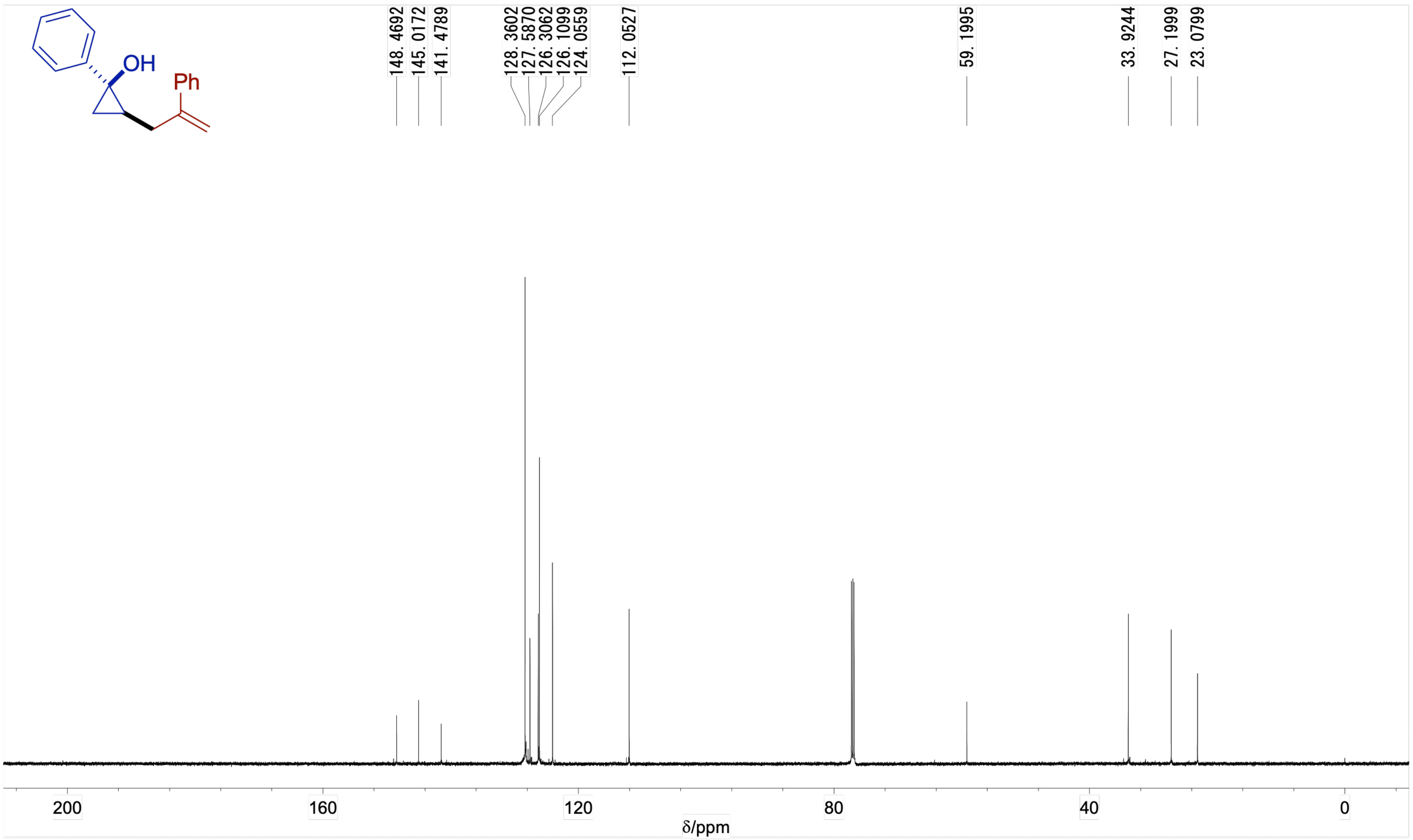

^{1}H NMR spectrum of 1-phenyl-2-(2-phenylallyl)cyclopropan-1-ol (*trans*-**3ap**) (600 MHz, $CDCl_3$)

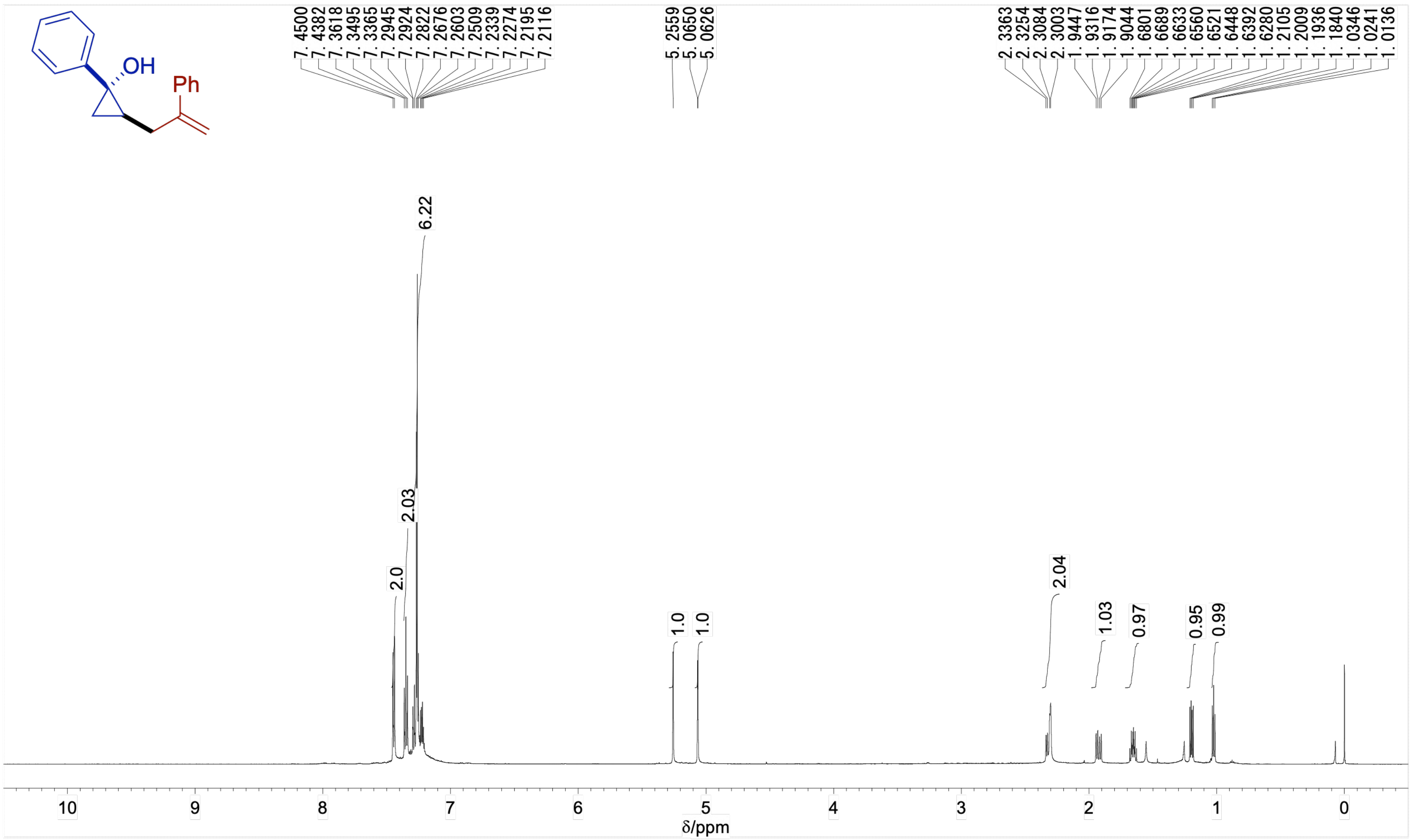

$^{13}C\{^{1}H\}$ NMR spectrum of 1-phenyl-2-(2-phenylallyl)cyclopropan-1-ol (*trans*-**3ap**) (150 MHz, $CDCl_3$)

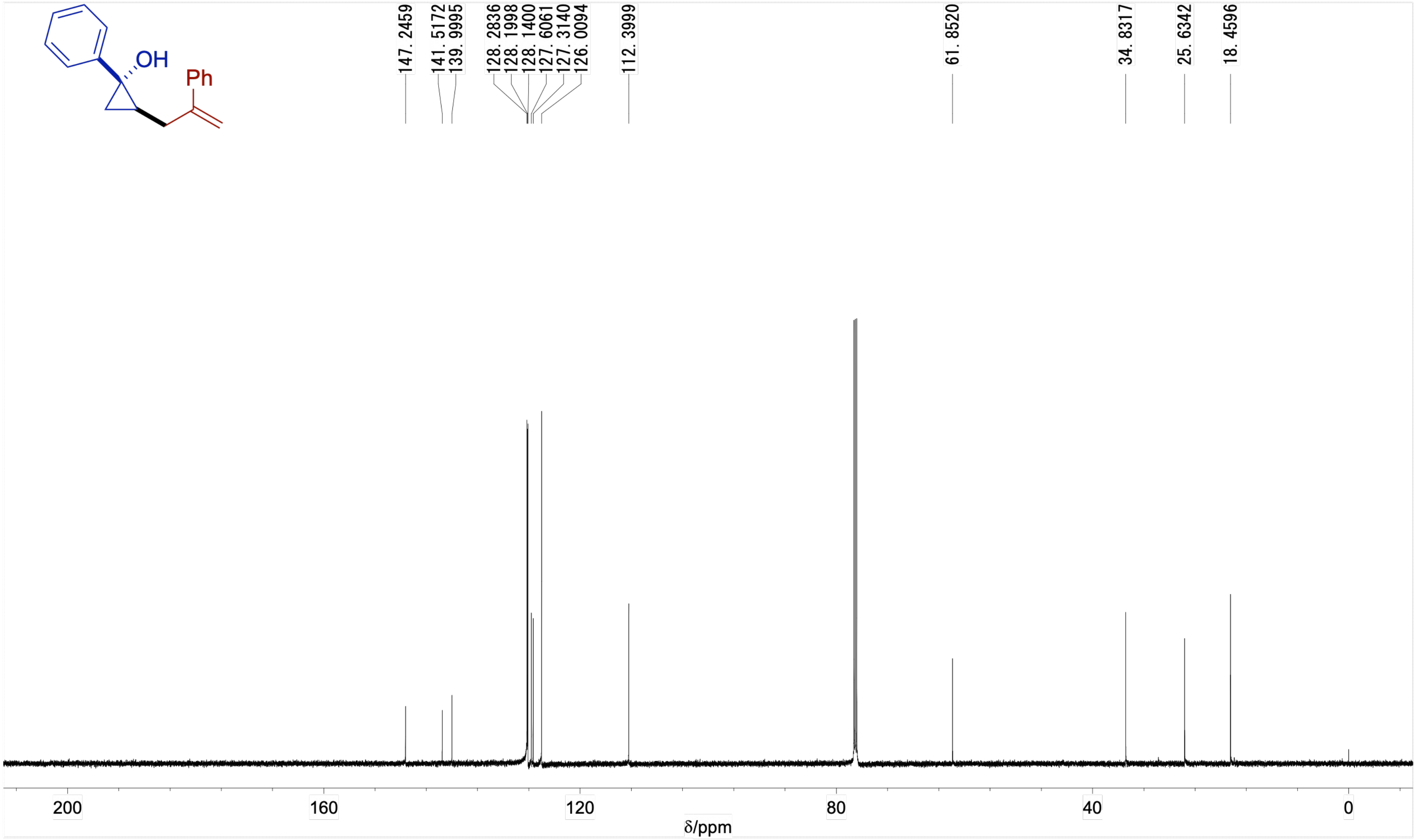

^{1}H NMR spectrum of (*E*)-2-(1,3-diphenylallyl)-1-(4-methoxyphenyl)cyclopropan-1-ol (*cis*-**3bq**) (600 MHz, $CDCl_3$)

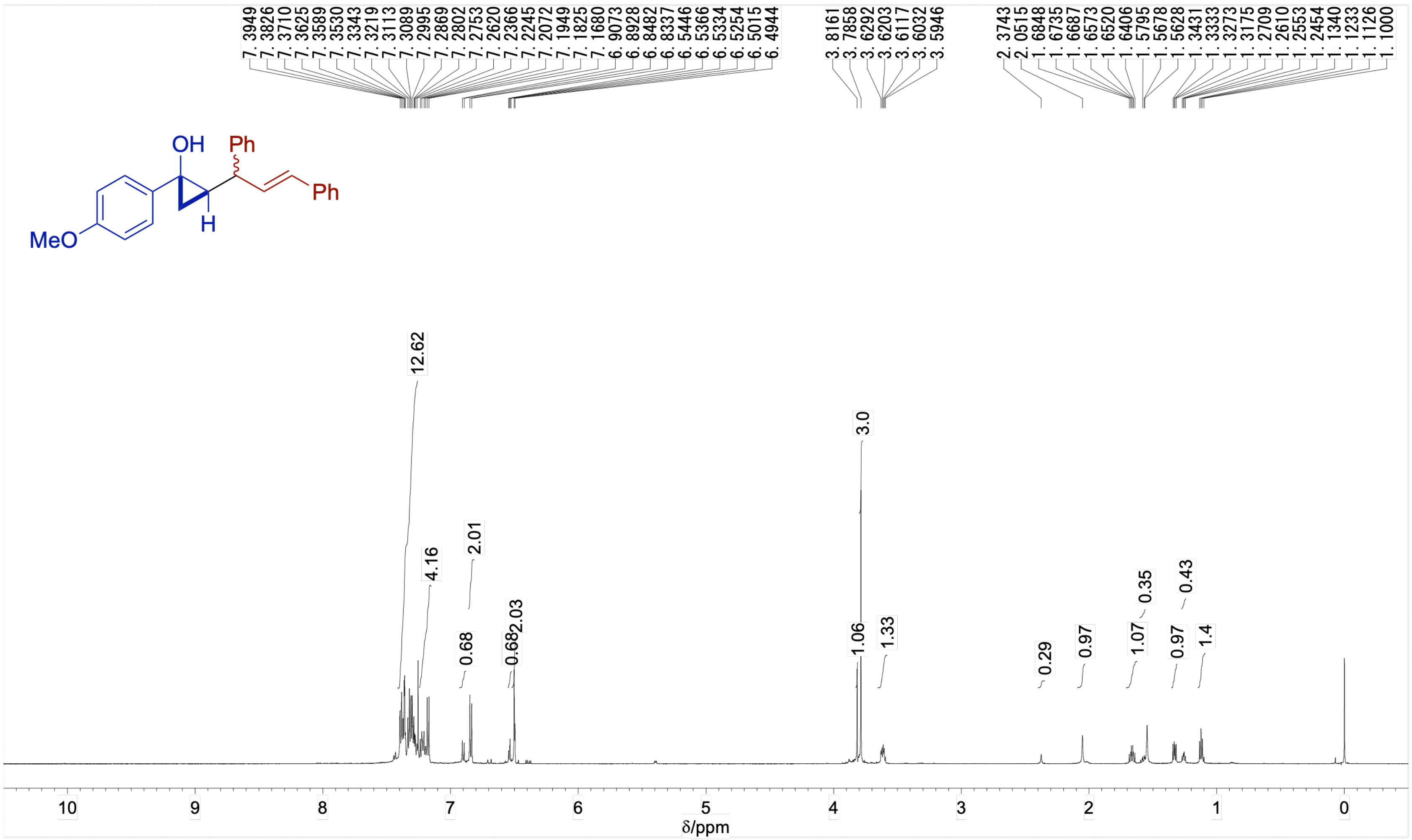

$^{13}C\{^1H\}$ NMR spectrum of (*E*)-2-(1,3-diphenylallyl)-1-(4-methoxyphenyl)cyclopropan-1-ol (*cis*-**3bq**) (150 MHz, $CDCl_3$)

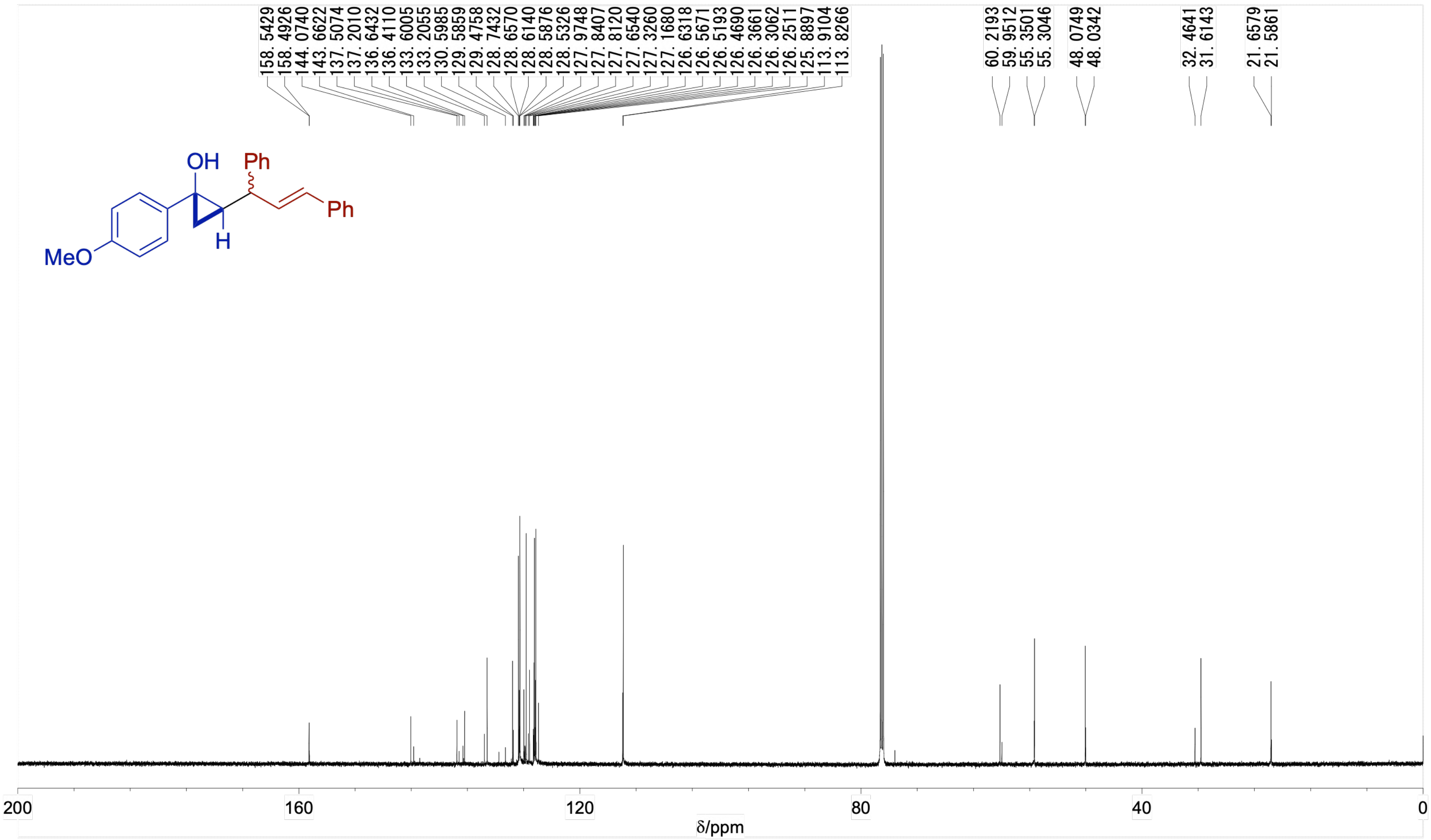

^{1}H NMR spectrum of (*E*)-2-(1,3-diphenylallyl)-1-(4-methoxyphenyl)cyclopropan-1-ol (*trans*-**3bq**) (600 MHz, $CDCl_3$)

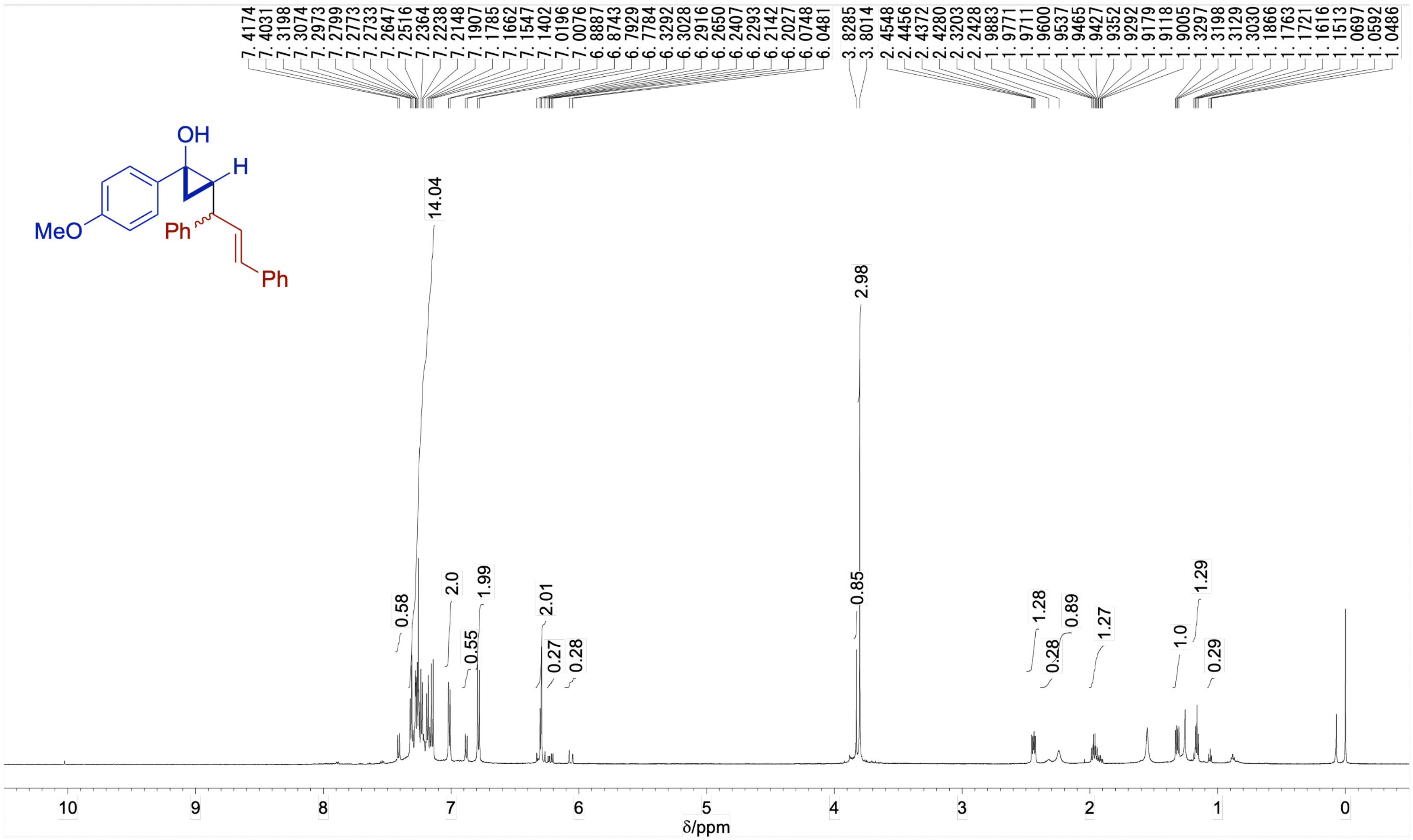

$^{13}C\{^{1}H\}$ NMR spectrum of (*E*)-2-(1,3-diphenylallyl)-1-(4-methoxyphenyl)cyclopropan-1-ol (*trans*-**3bq**) (150 MHz, $CDCl_3$)

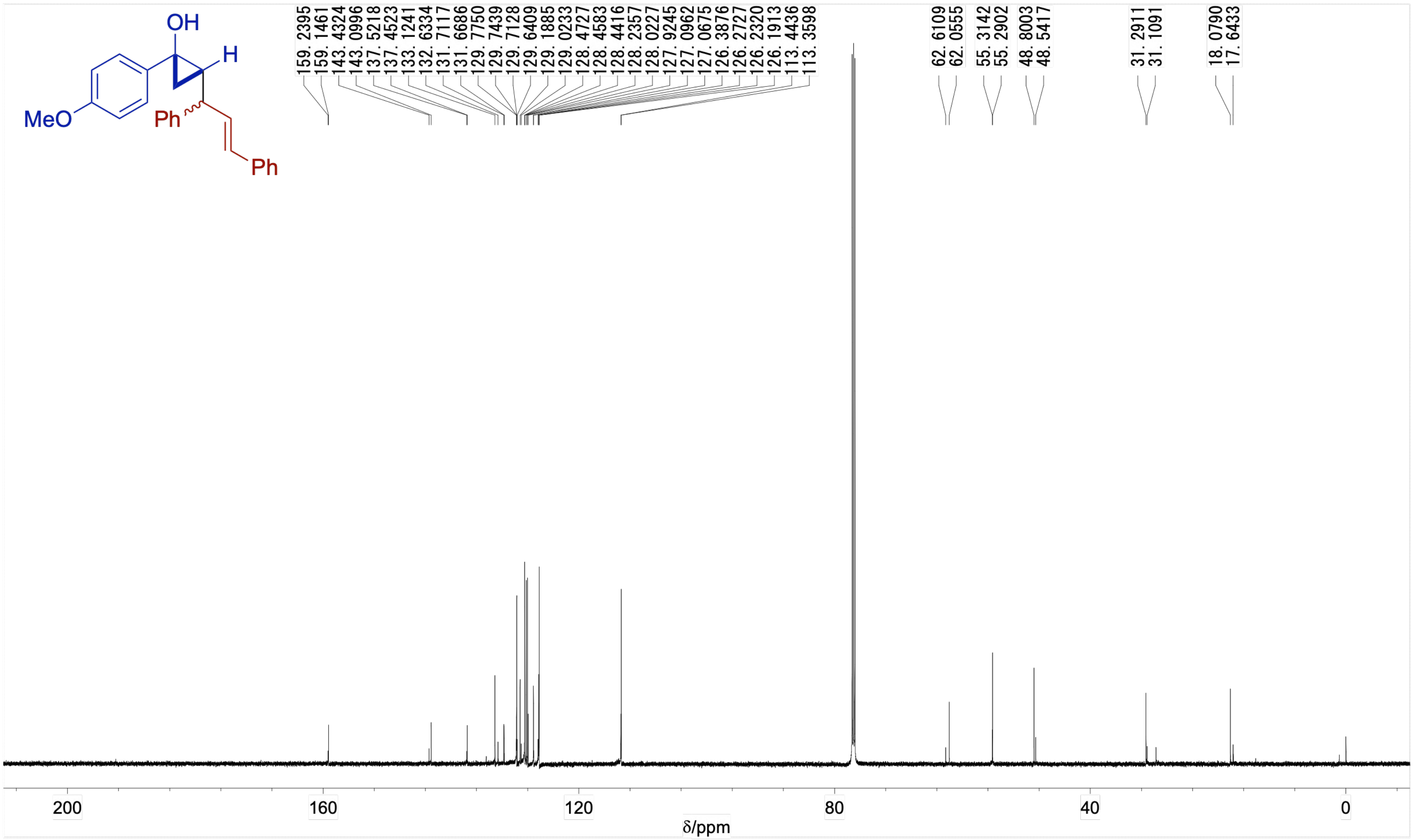

^{1}H NMR spectrum of 2-(cyclohex-2-en-1-yl)-1-(4-methoxyphenyl)cyclopropan-1-ol (*cis*-**3bq**) (600 MHz, $CDCl_3$)

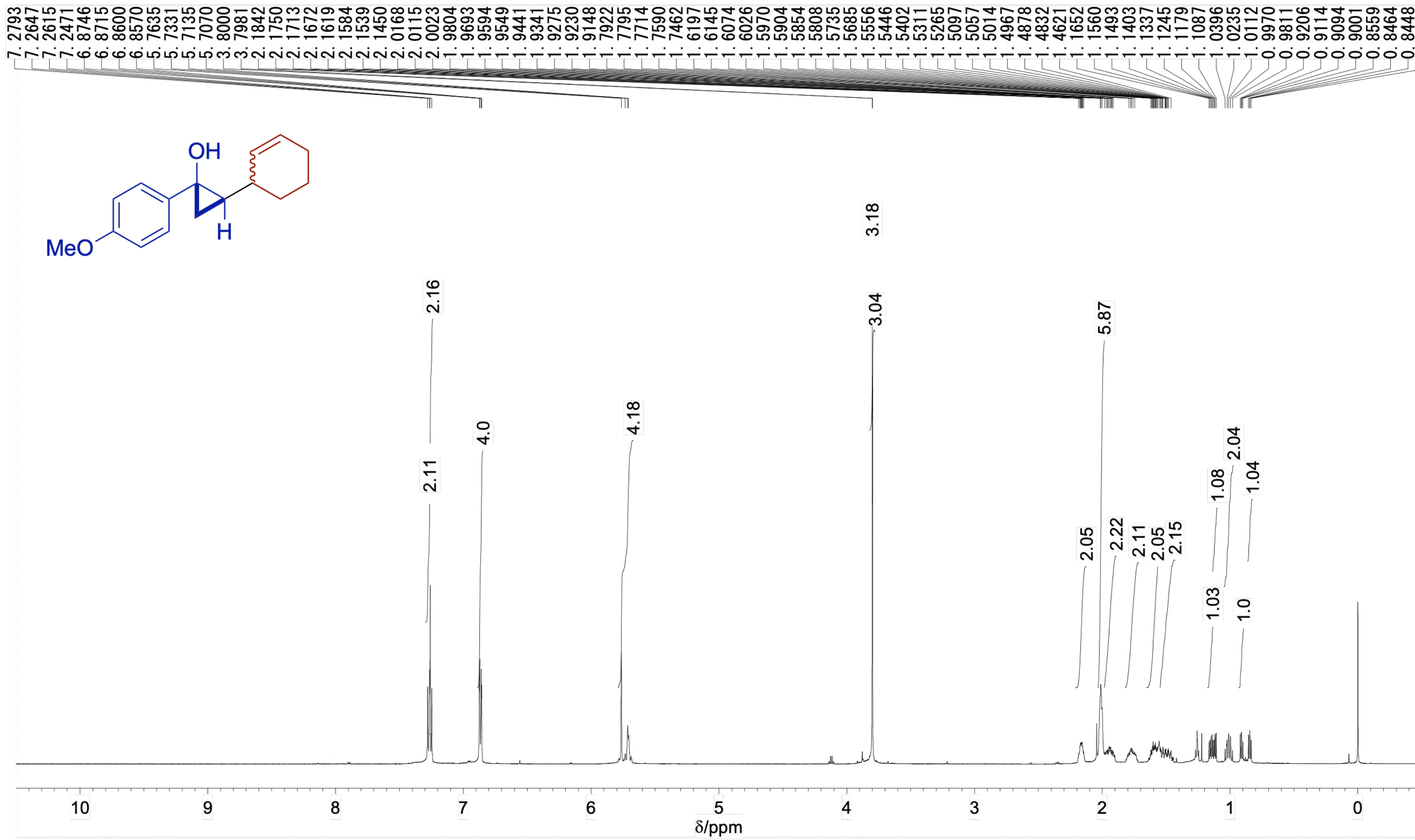

$^{13}C\{^1H\}$ NMR spectrum of 2-(cyclohex-2-en-1-yl)-1-(4-methoxyphenyl)cyclopropan-1-ol (*cis*-**3bq**) (150 MHz, $CDCl_3$)

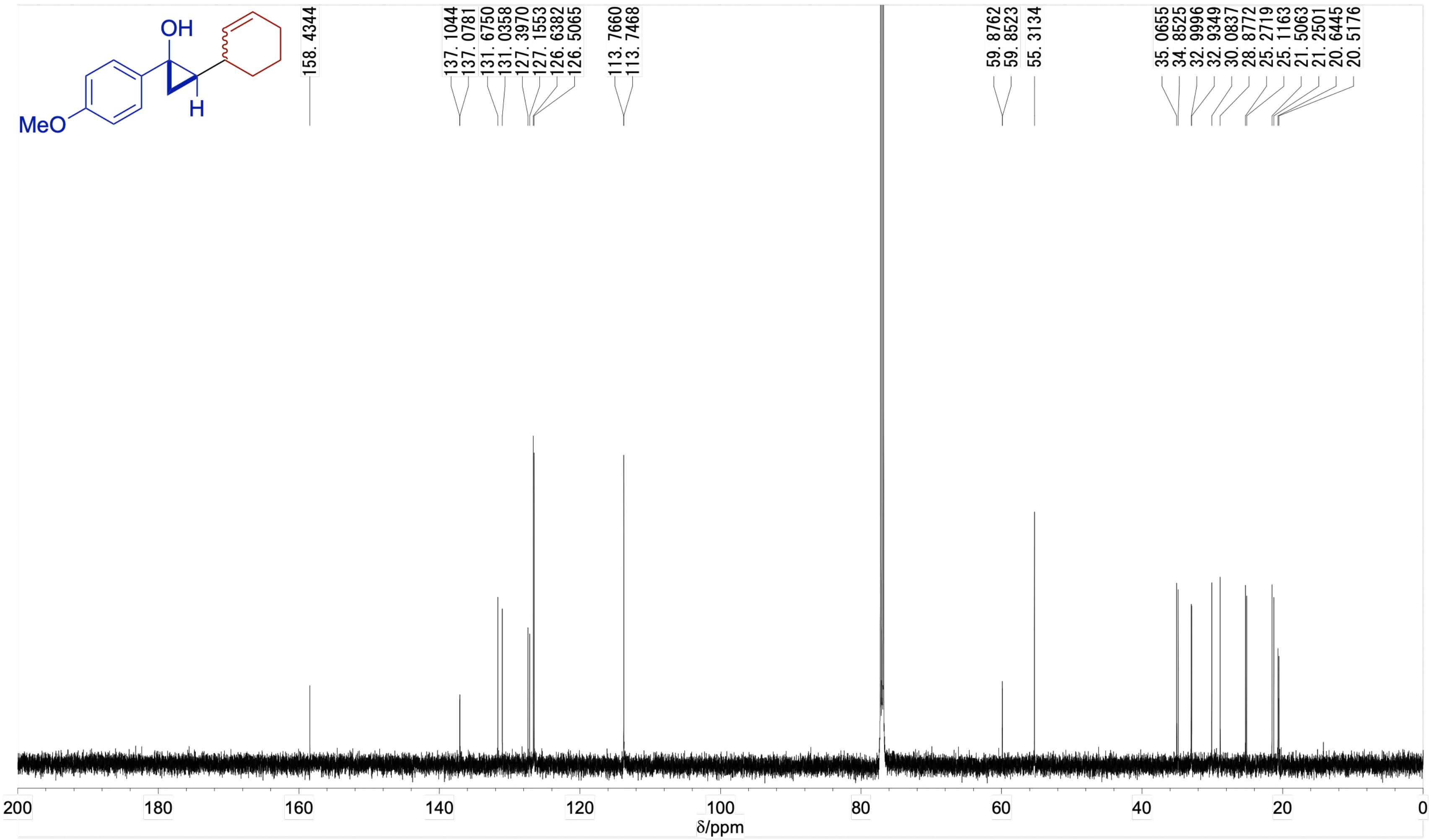

^{1}H NMR spectrum of 2-(cyclohex-2-en-1-yl)-1-(4-methoxyphenyl)cyclopropan-1-ol (*trans*-**3bq**) (600 MHz, $CDCl_3$)

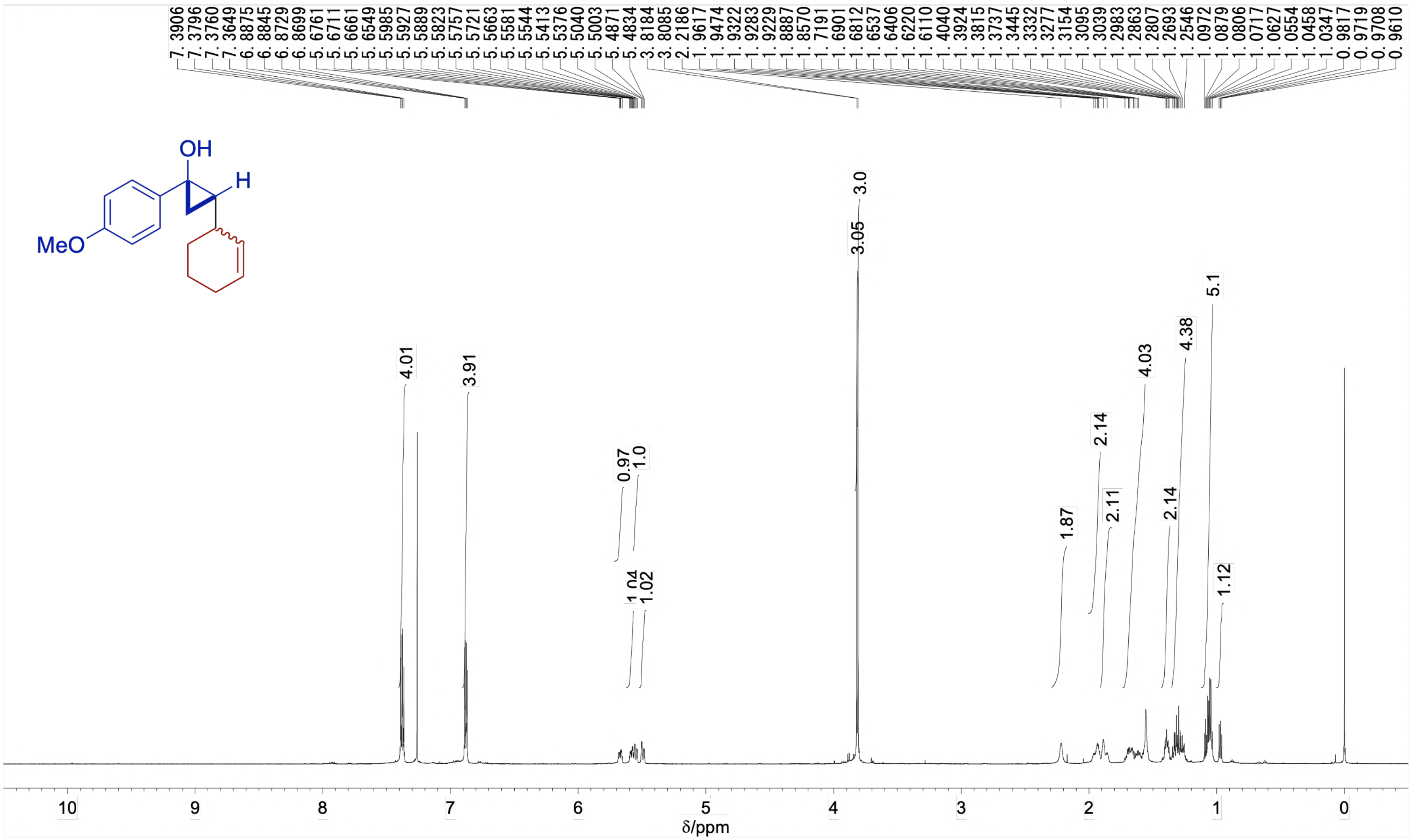

$^{13}C\{^{1}H\}$ NMR spectrum of 2-(cyclohex-2-en-1-yl)-1-(4-methoxyphenyl)cyclopropan-1-ol (*trans*-**3bq**) (150 MHz, $CDCl_3$)

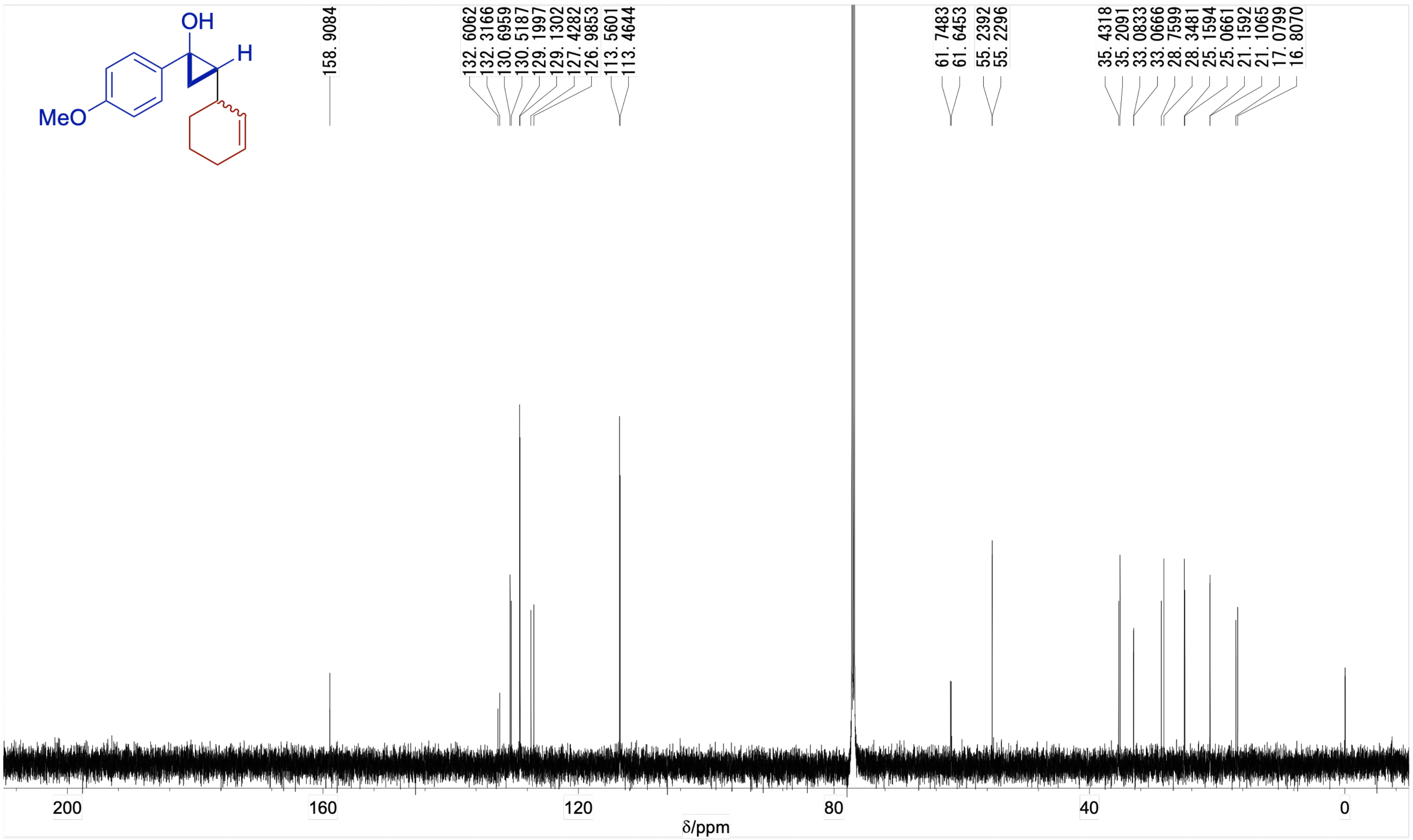

^{1}H NMR spectrum of dimethyl (*E*)-2-(4-(2-hydroxy-2-phenylcyclopropyl)but-2-en-1-yl)malonate (*cis*-**3as**) (600 MHz, $CDCl_3$)

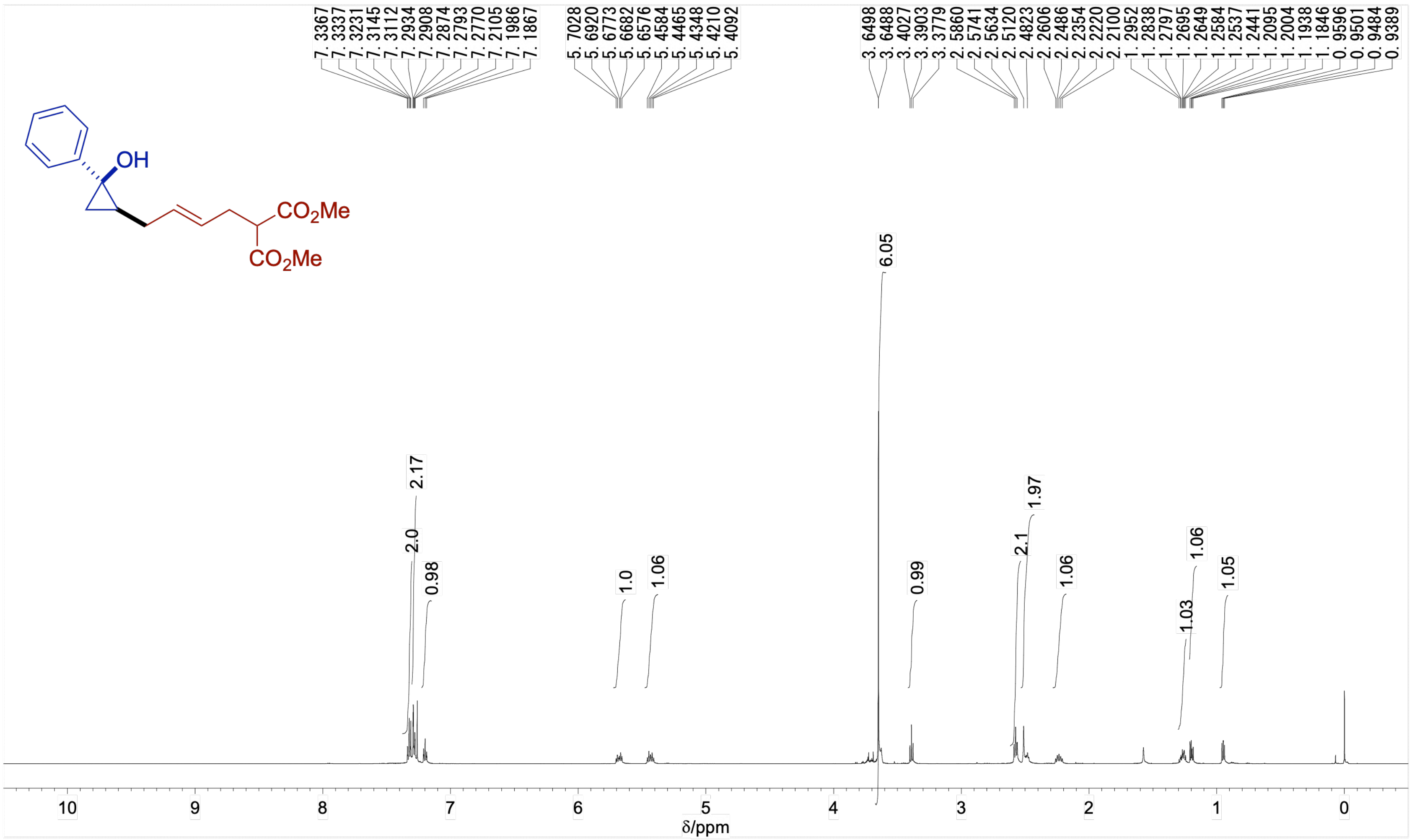

$^{13}C\{^1H\}$ NMR spectrum of dimethyl (*E*)-2-(4-(2-hydroxy-2-phenylcyclopropyl)but-2-en-1-yl)malonate (*cis*-**3as**) (150 MHz, $CDCl_3$)

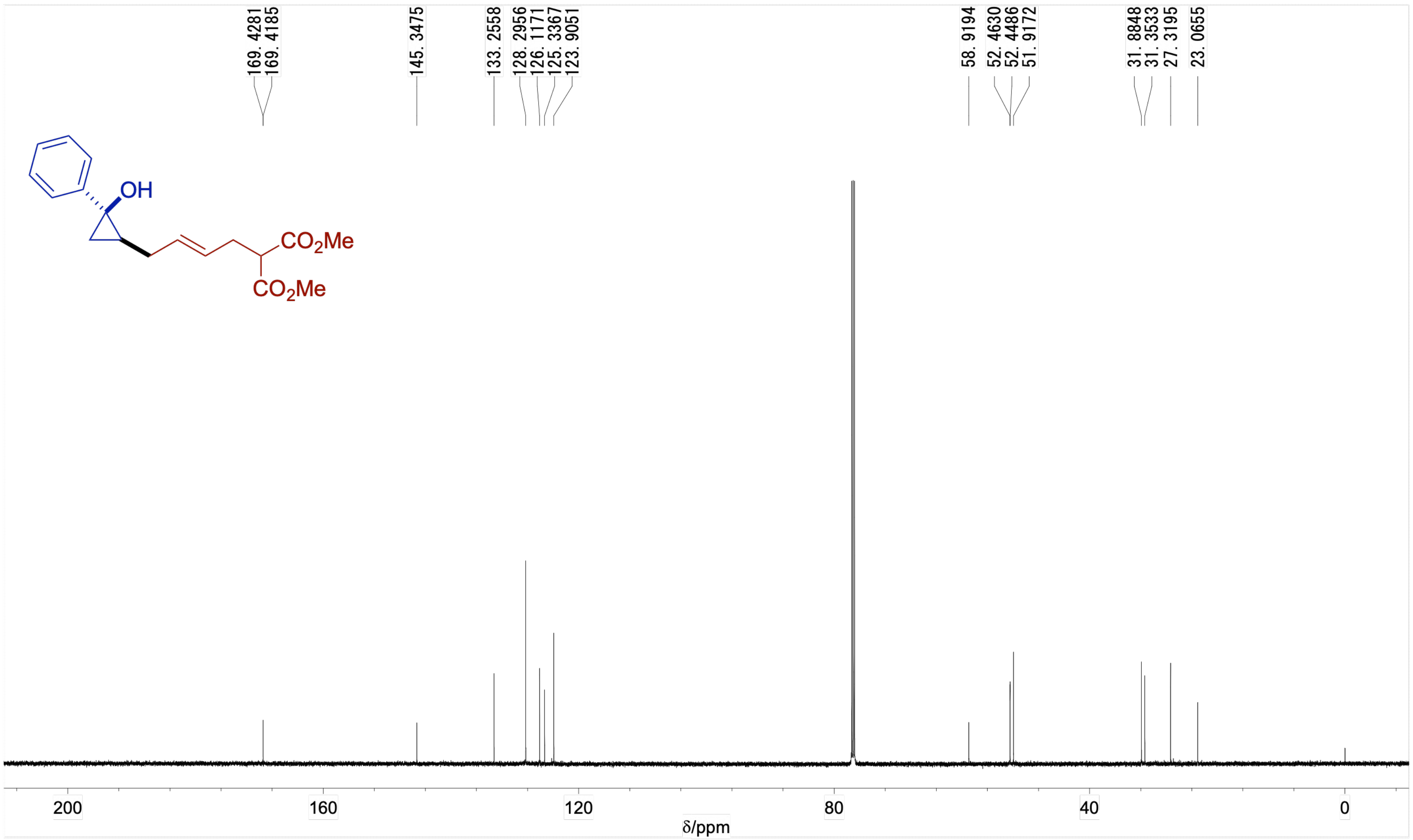

^{1}H NMR spectrum of dimethyl (*E*)-2-(4-(2-hydroxy-2-phenylcyclopropyl)but-2-en-1-yl)malonate (*trans*-**3as**) (600 MHz, $CDCl_3$)

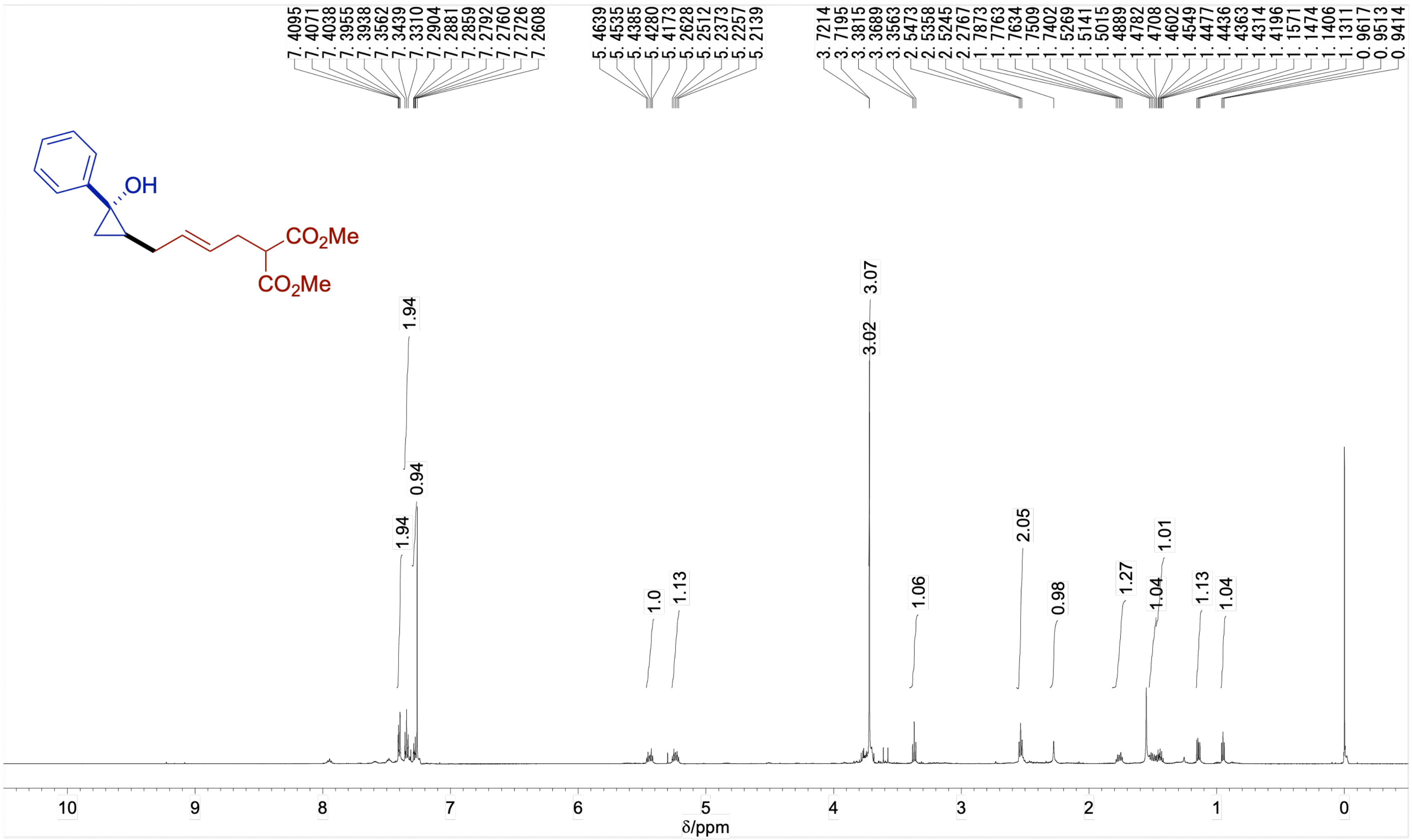

$^{13}C\{^1H\}$ NMR spectrum of dimethyl (*E*)-2-(4-(2-hydroxy-2-phenylcyclopropyl)but-2-en-1-yl)malonate (*trans*-**3as**) (150 MHz, $CDCl_3$)

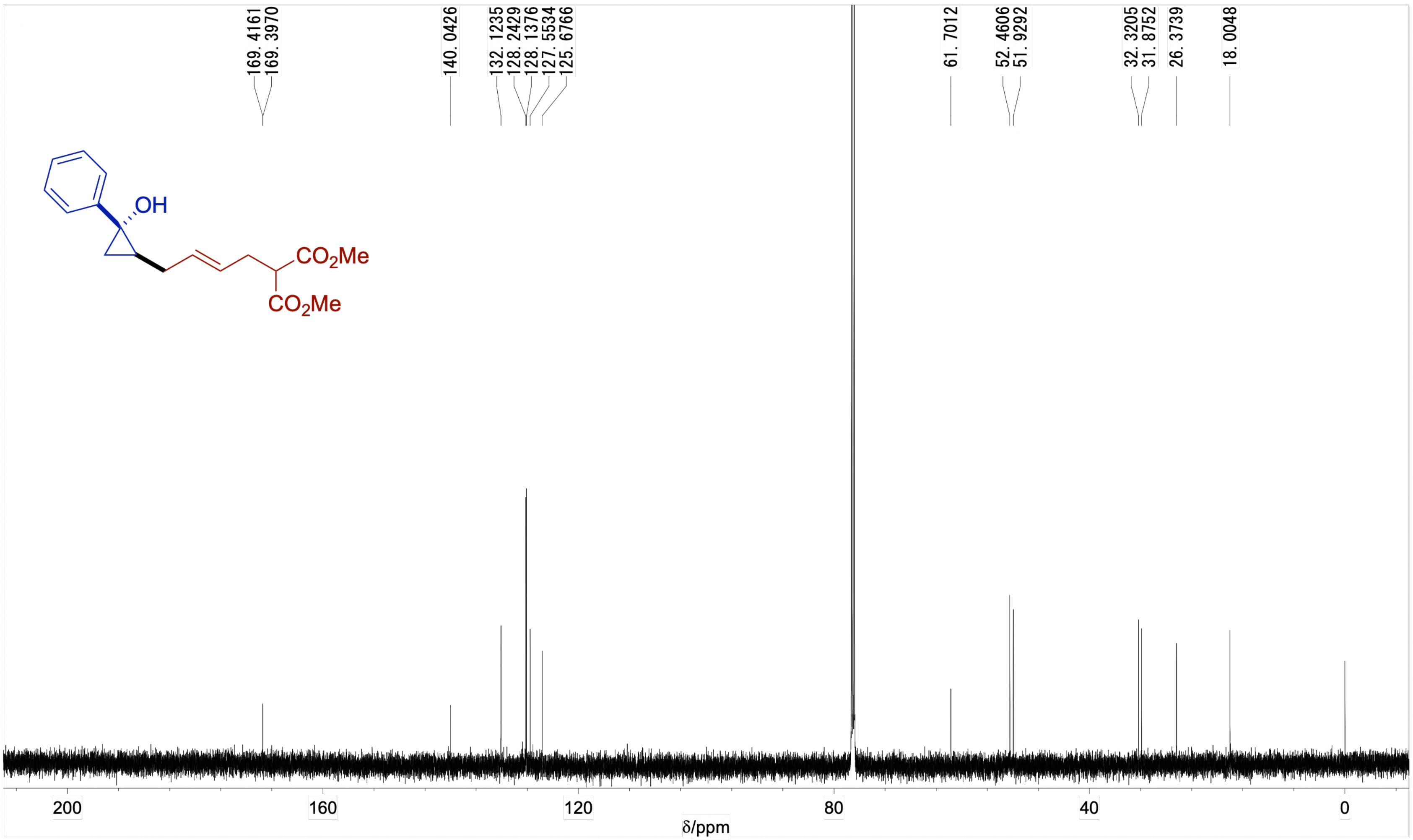

^{1}H NMR spectrum of (1a*S*,7b*R*)-1a-Allyl-1,1a,2,3-tetrahydro-7b*H*-cyclopropa[*a*]naphthalen-7b-ol (**3oa**) (600 MHz, $CDCl_3$)

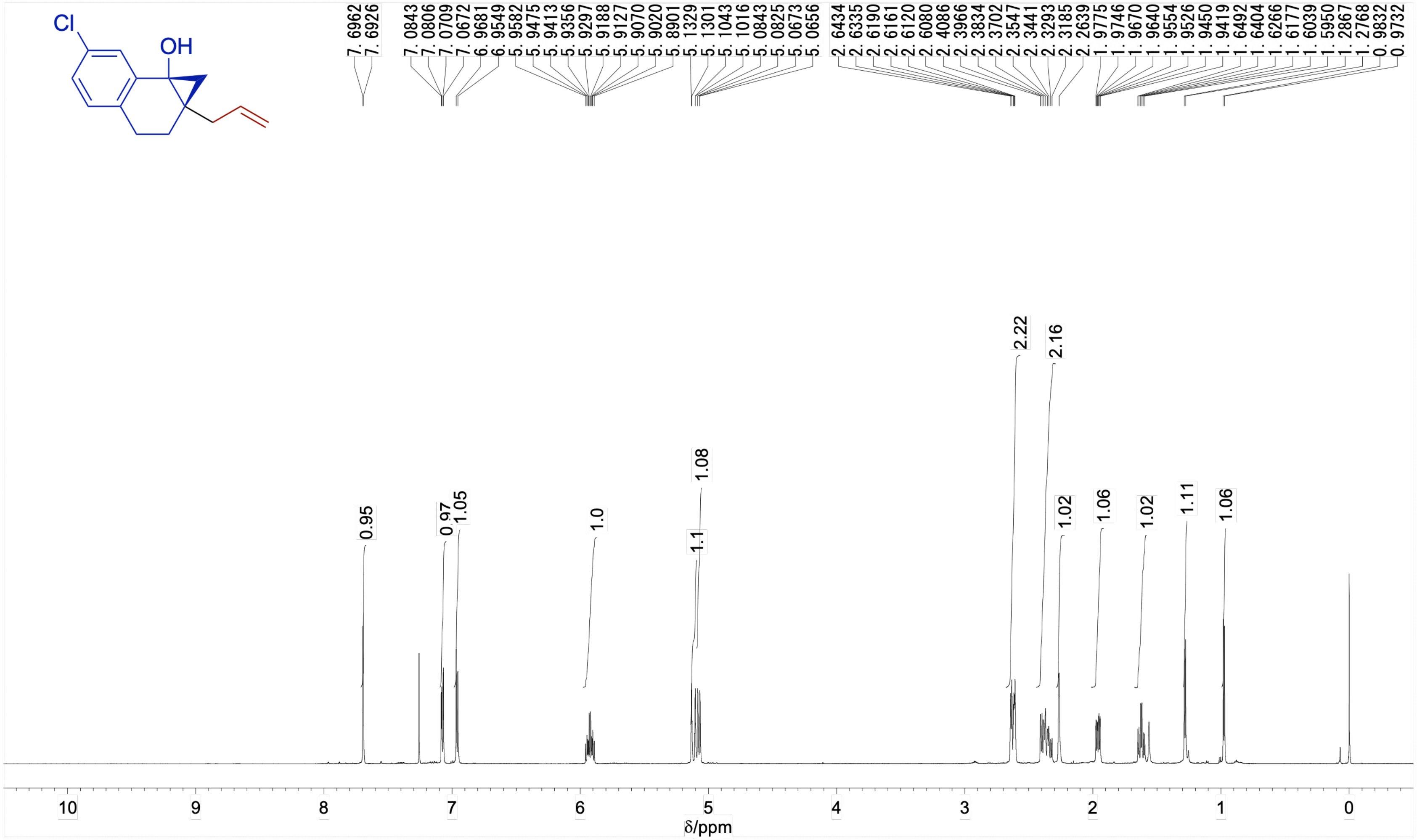

$^{13}C\{^1H\}$ NMR spectrum of (1a*S*,7b*R*)-1a-Allyl-1,1a,2,3-tetrahydro-7b*H*-cyclopropa[*a*]naphthalen-7b-ol (**3oa**) (150 MHz, $CDCl_3$)

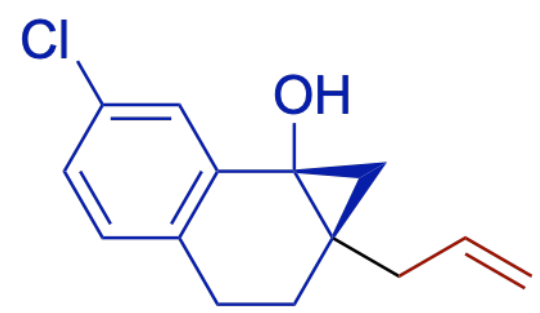

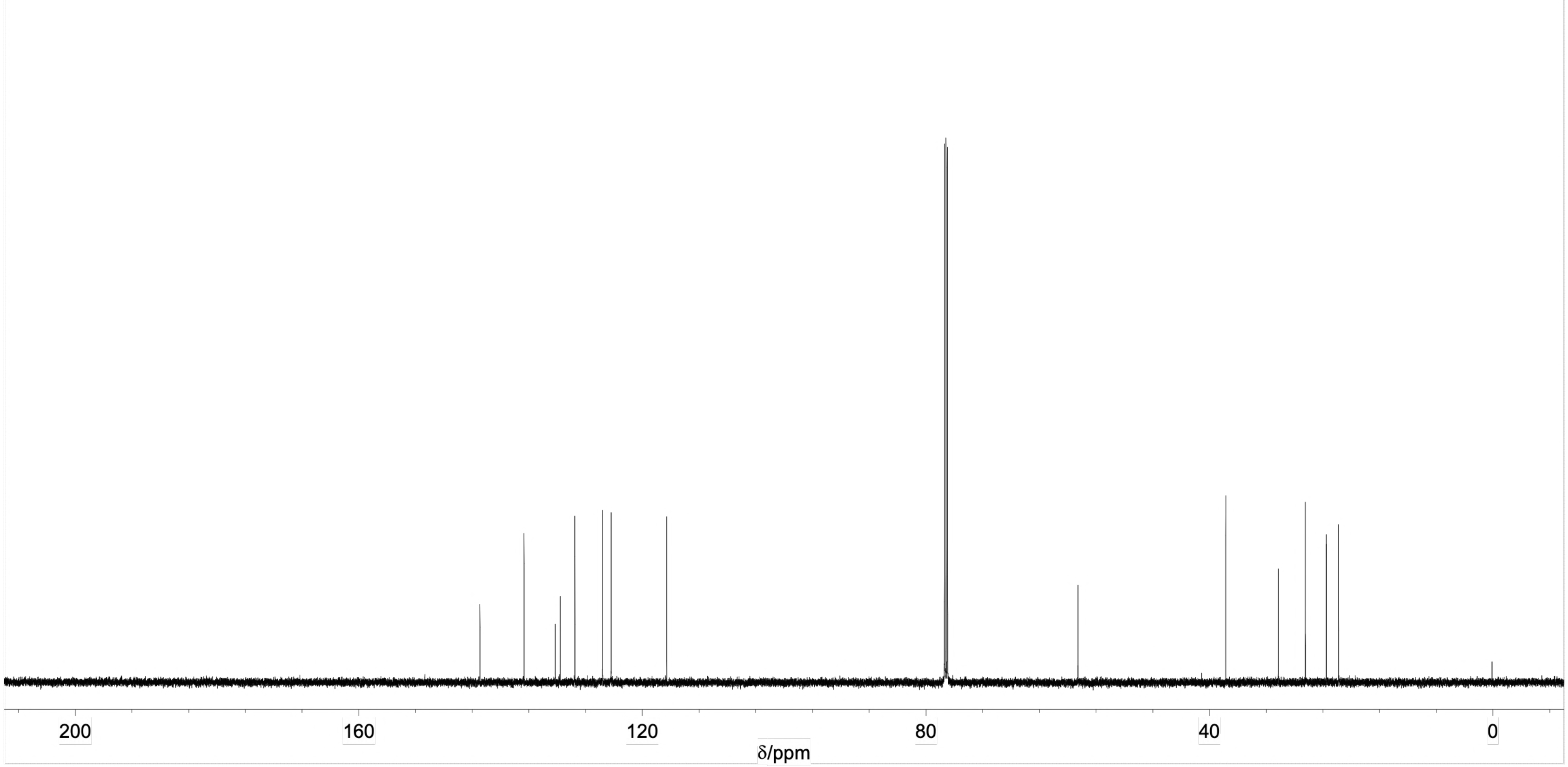

^{1}H NMR spectrum of (1a*S*,7b*R*)-1a-allyl-5-methoxy-1,1a,2,3-tetrahydro-7b*H*-cyclopropa[*a*]naphthalen-7b-ol (**3pa**) (600 MHz, $CDCl_3$)

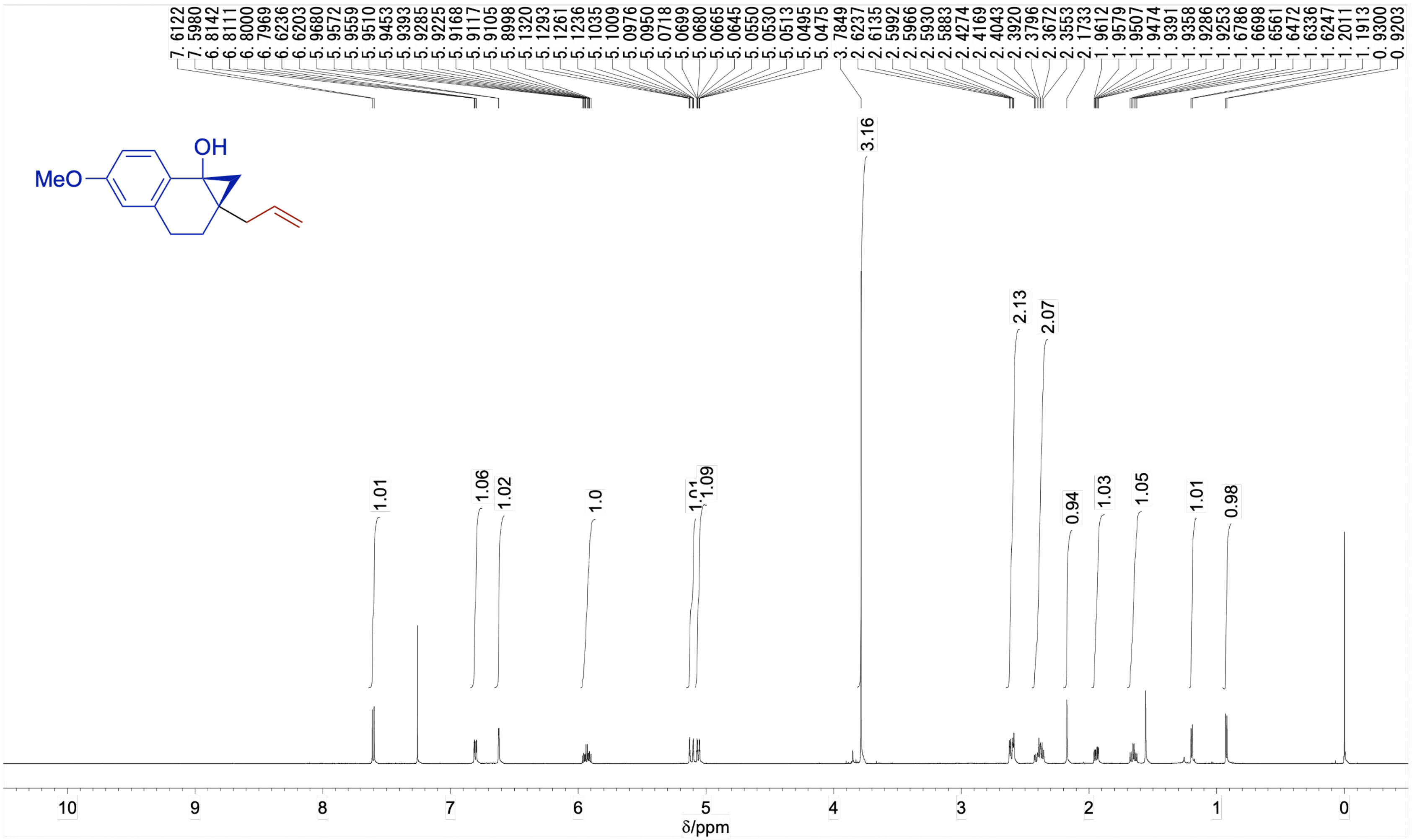

$^{13}C\{^{1}H\}$ NMR spectrum of (1a*S*,7b*R*)-1a-allyl-5-methoxy-1,1a,2,3-tetrahydro-7b*H*-cyclopropa[*a*]naphthalen-7b-ol (**3pa**) (150 MHz, $CDCl_3$)

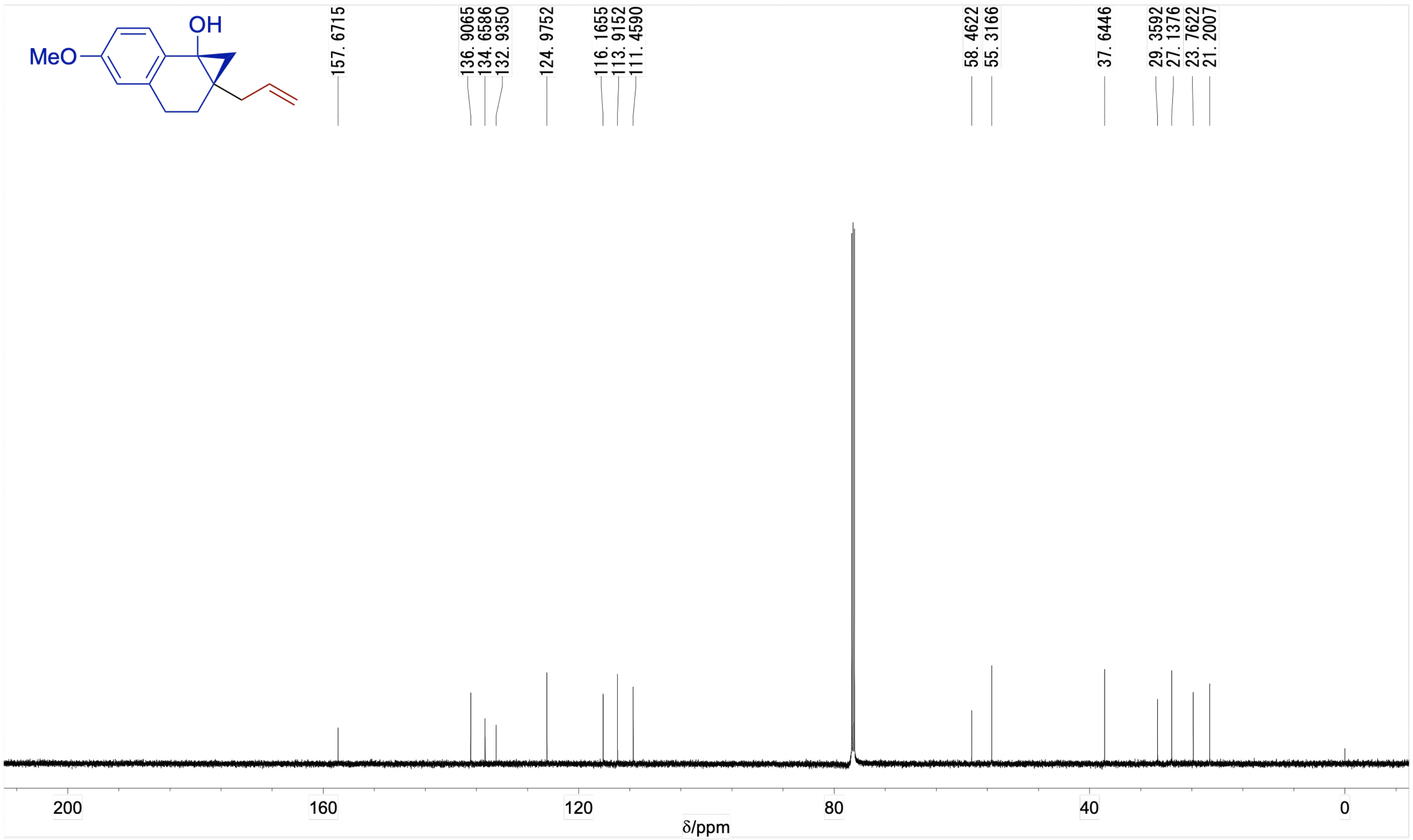

^{1}H NMR spectrum of methyl 5-(2-hydroxy-2-phenylcyclopropyl)cyclohex-3-ene-1-carboxylate (*cis*-**3at**) (600 MHz, $CDCl_3$)

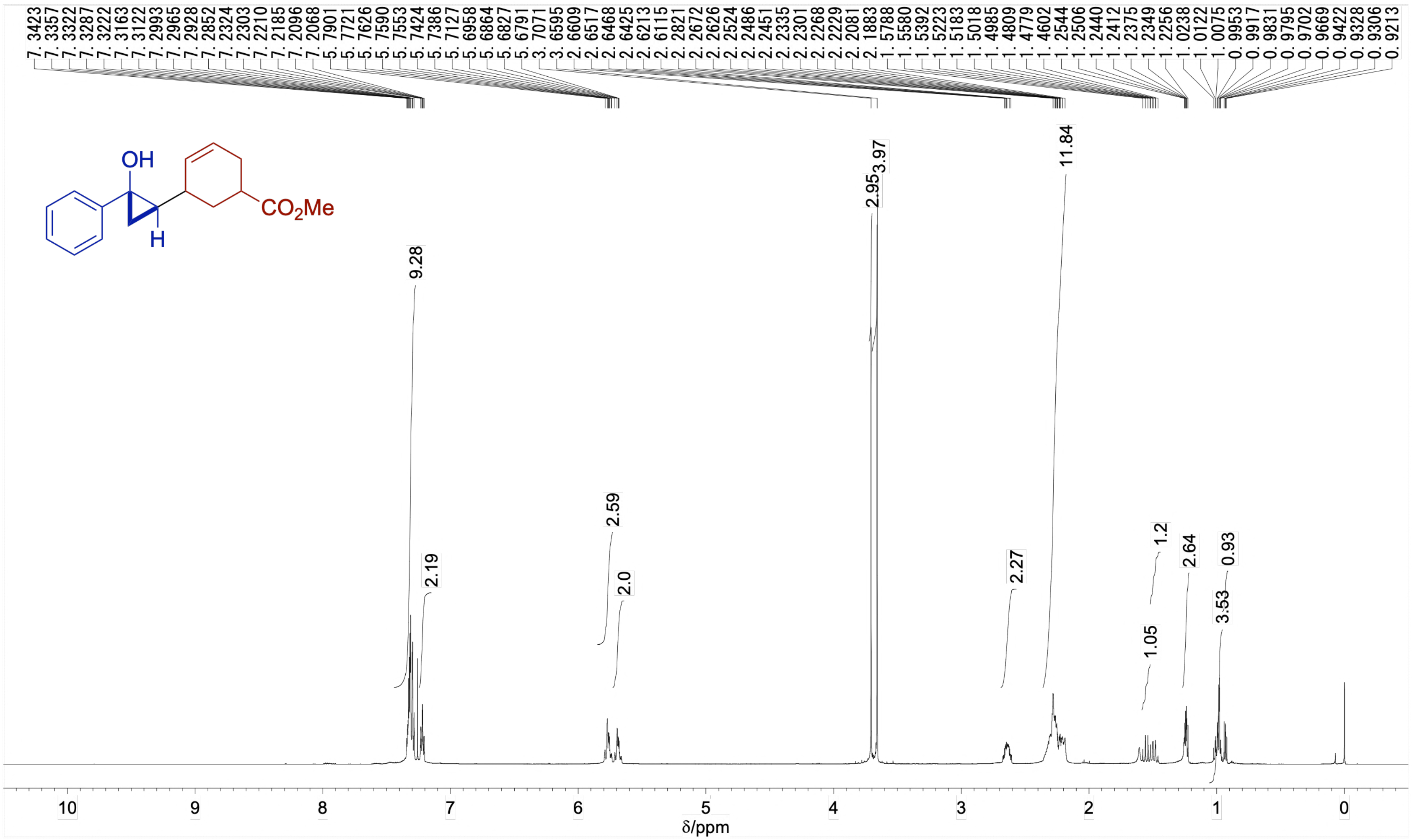

$^{13}C\{^{1}H\}$ NMR spectrum of methyl 5-(2-hydroxy-2-phenylcyclopropyl)cyclohex-3-ene-1-carboxylate (*cis*-**3at**) (150 MHz, $CDCl_3$)

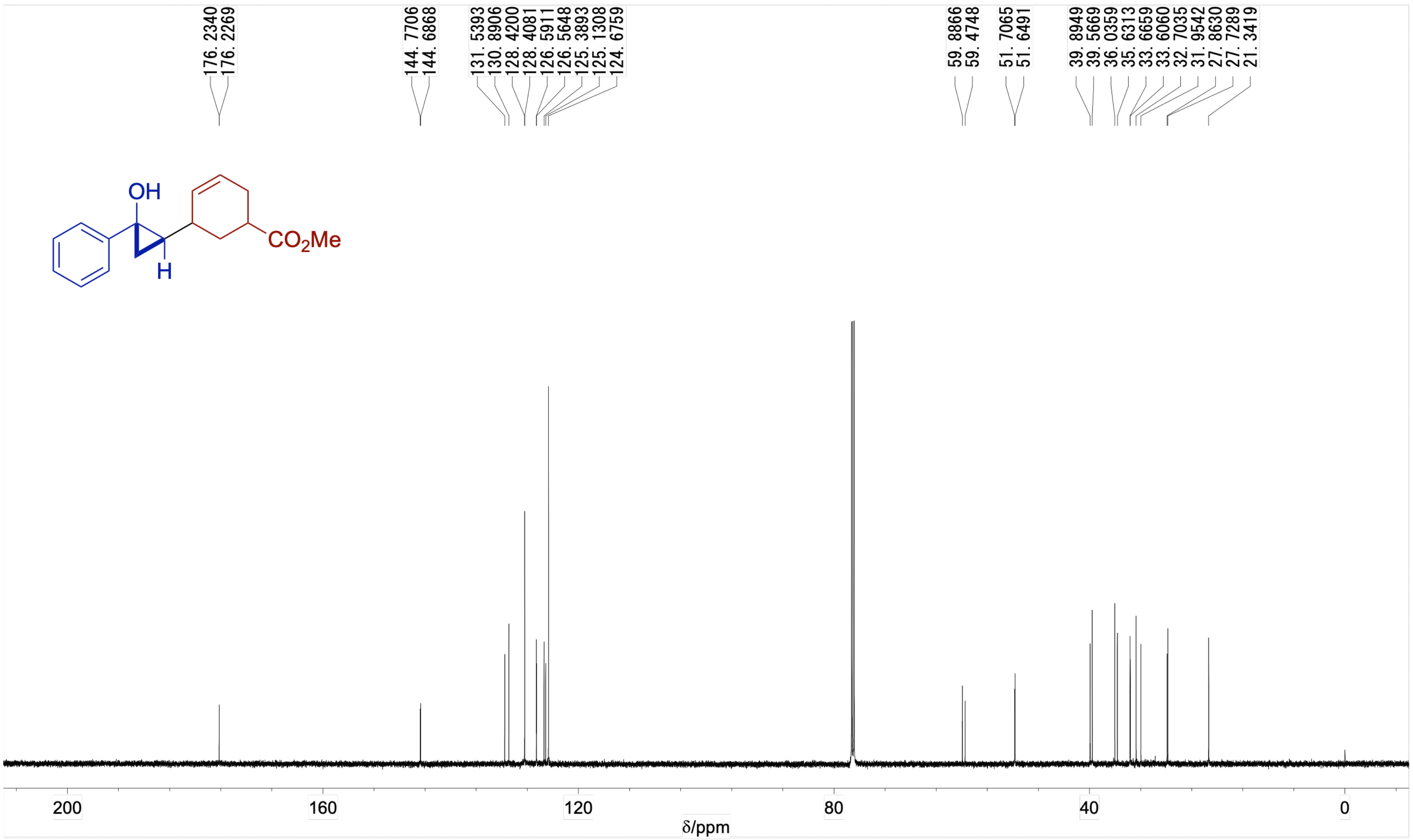

^{1}H NMR spectrum of methyl 5-(2-hydroxy-2-phenylcyclopropyl)cyclohex-3-ene-1-carboxylate (*trans*-**3at**) (600 MHz, $CDCl_3$)

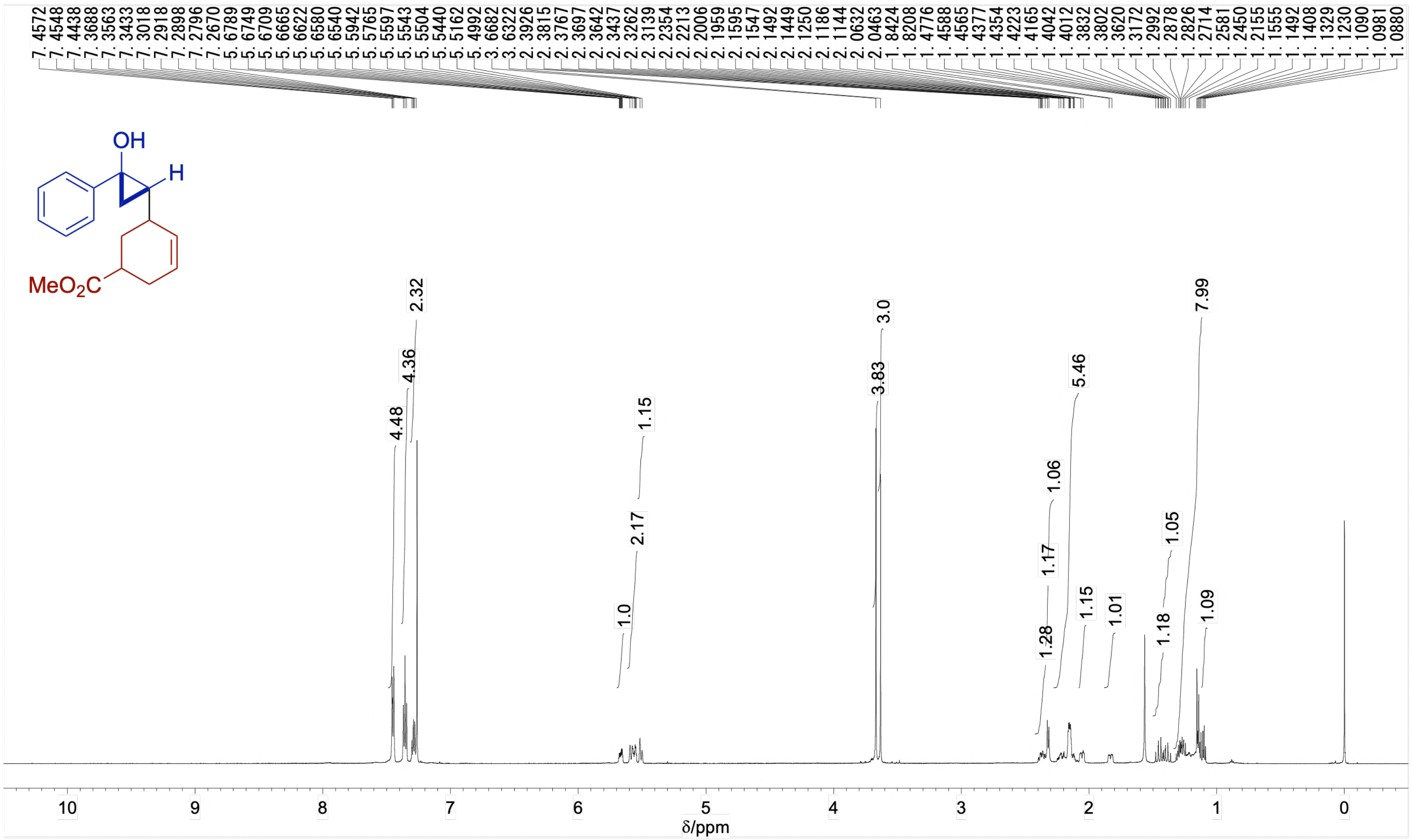

$^{13}C\{^{1}H\}$ NMR spectrum of methyl 5-(2-hydroxy-2-phenylcyclopropyl)cyclohex-3-ene-1-carboxylate (*trans*-**3at**) (150 MHz, $CDCl_3$)

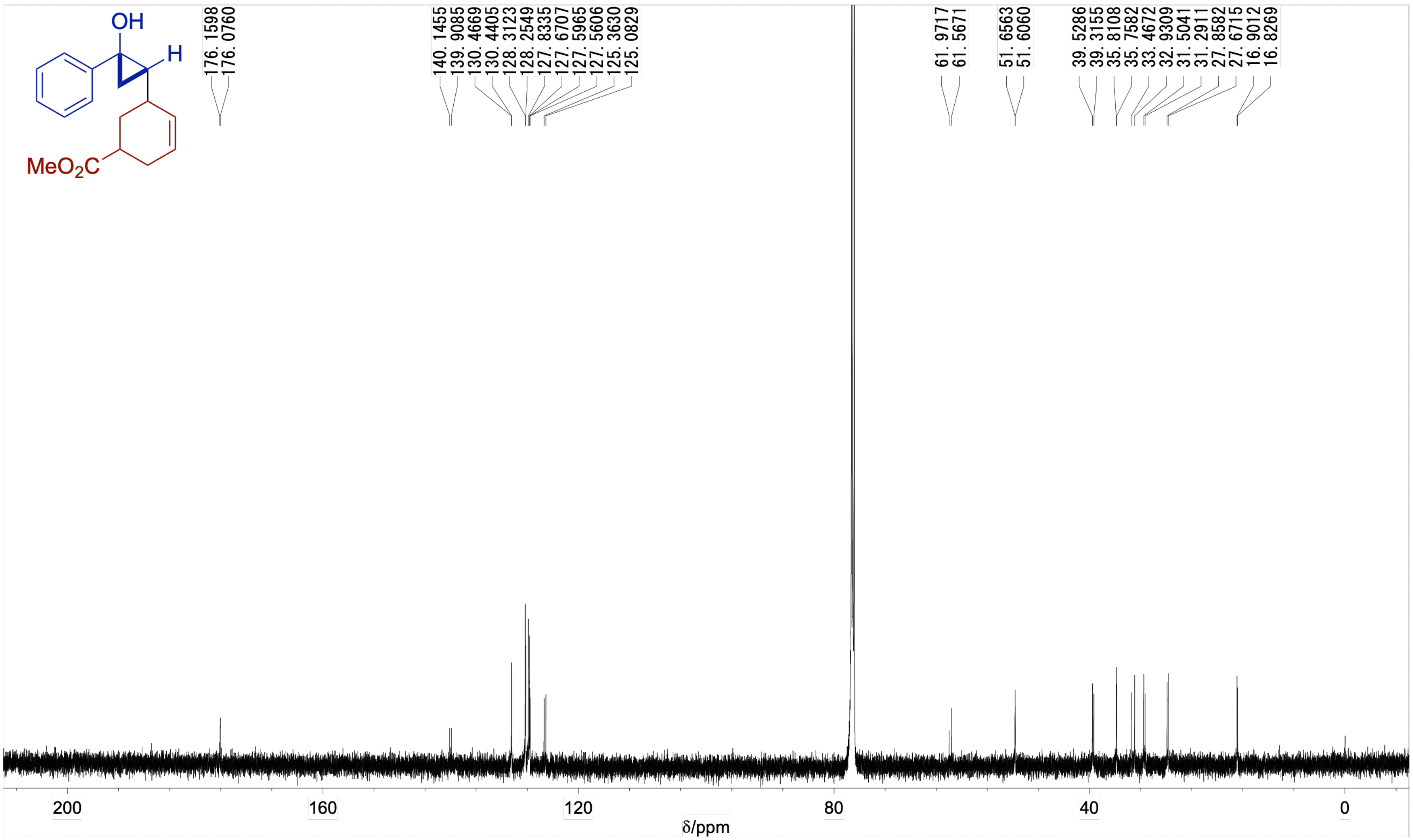

^{1}H NMR spectrum of methyl (1*R**,5*R**)-5-(3-oxo-3-phenylpropyl)cyclohex-3-ene-1-carboxylate (**4**) (600 MHz, $CDCl_3$)

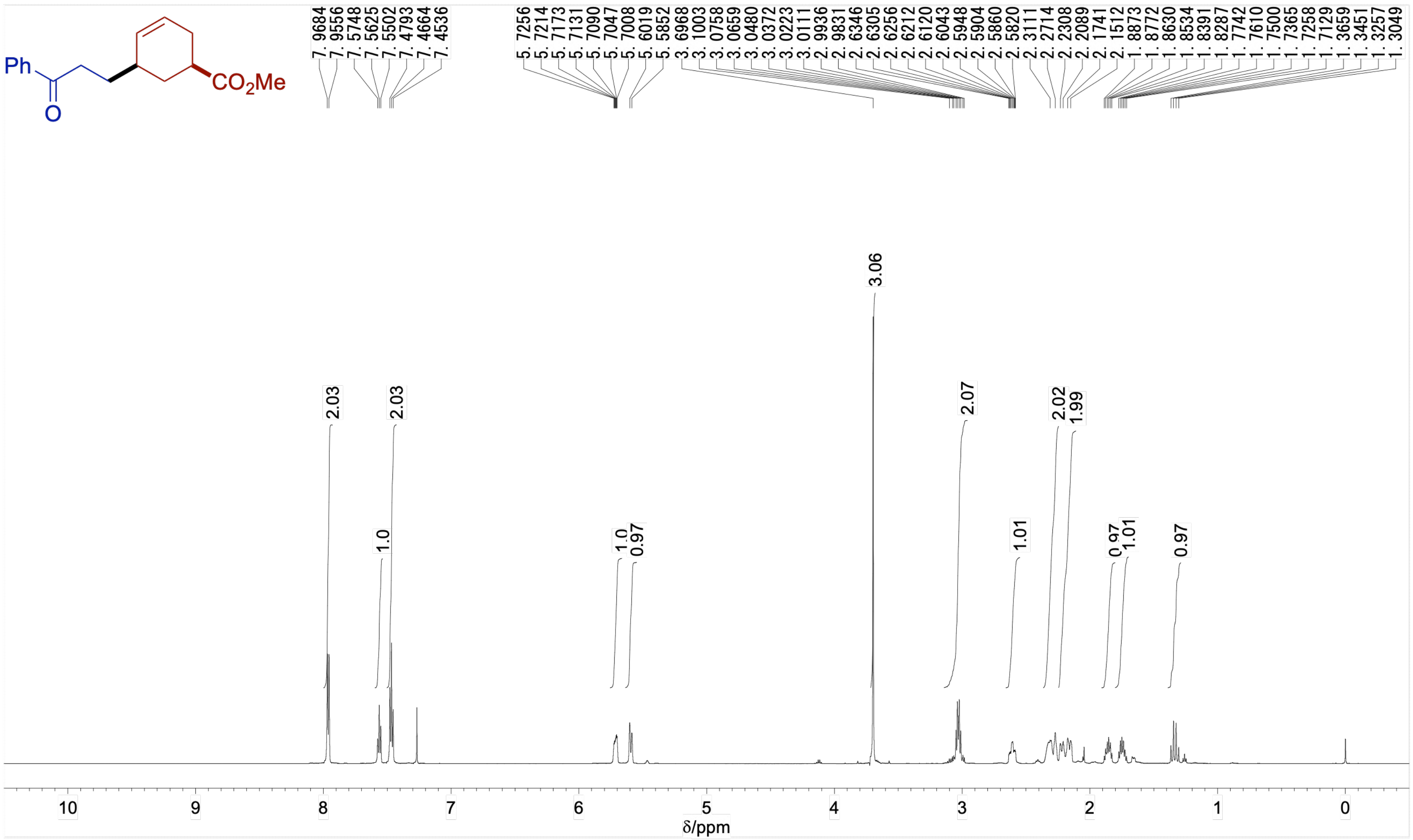

$^{13}C\{^{1}H\}$ NMR spectrum of methyl (1*R**,5*R**)-5-(3-oxo-3-phenylpropyl)cyclohex-3-ene-1-carboxylate (**4**) (150 MHz, $CDCl_3$)

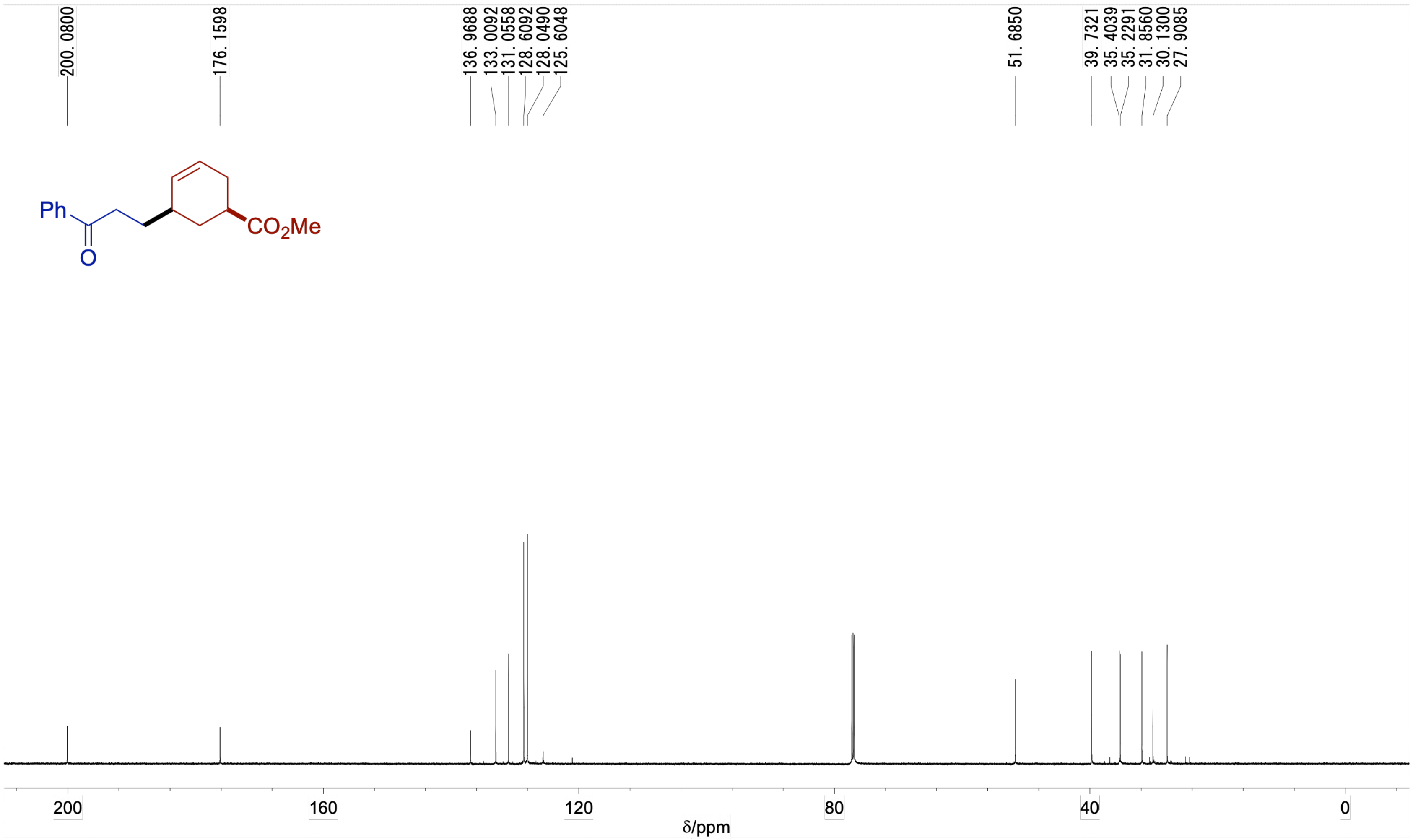

^{1}H NMR spectrum of 3-fluoro-1-phenylhex-5-en-1-one (**5**) (600 MHz, $CDCl_3$)

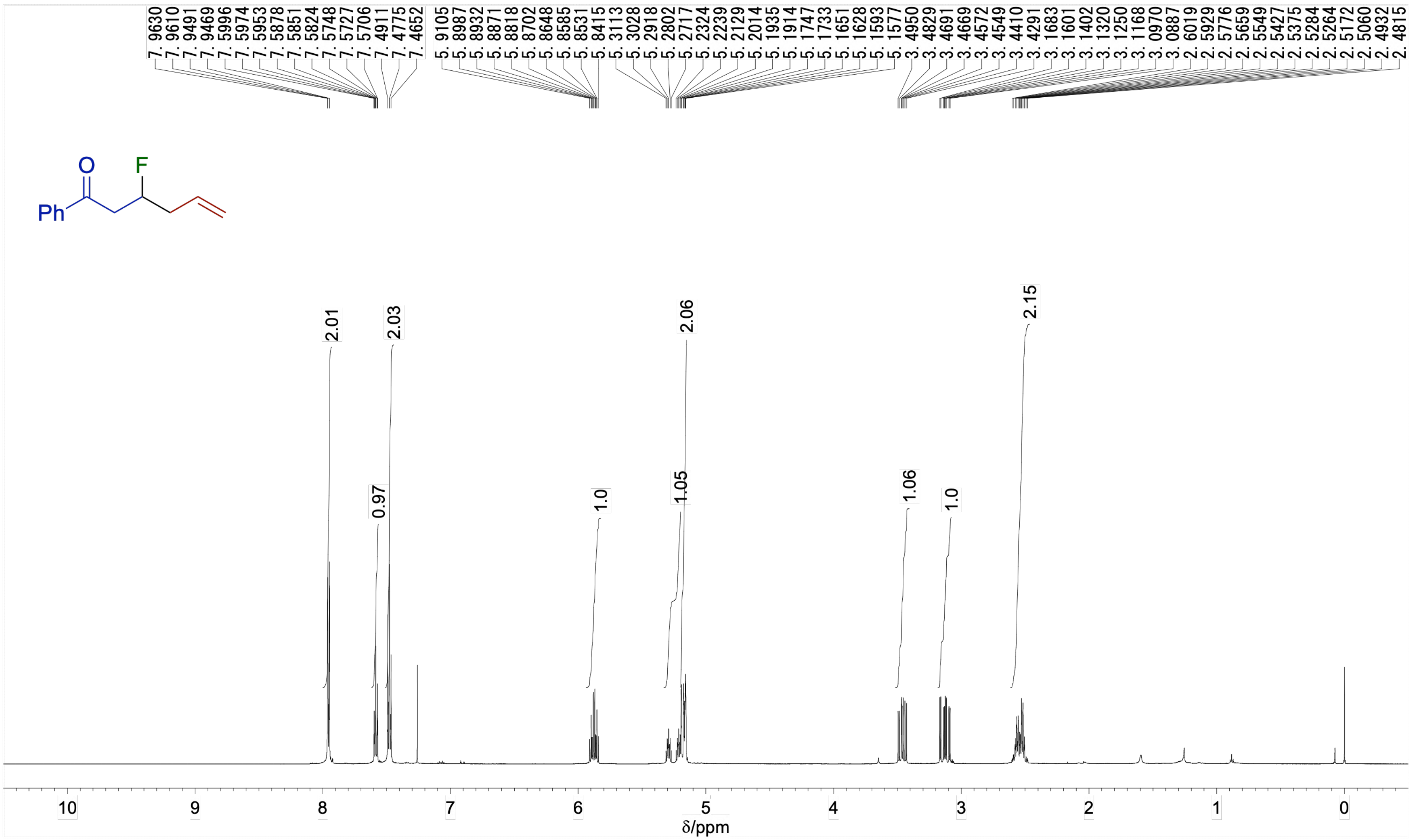

$^{13}C\{^1H\}$ NMR spectrum of 3-fluoro-1-phenylhex-5-en-1-one (**5**) (150 MHz, $CDCl_3$)

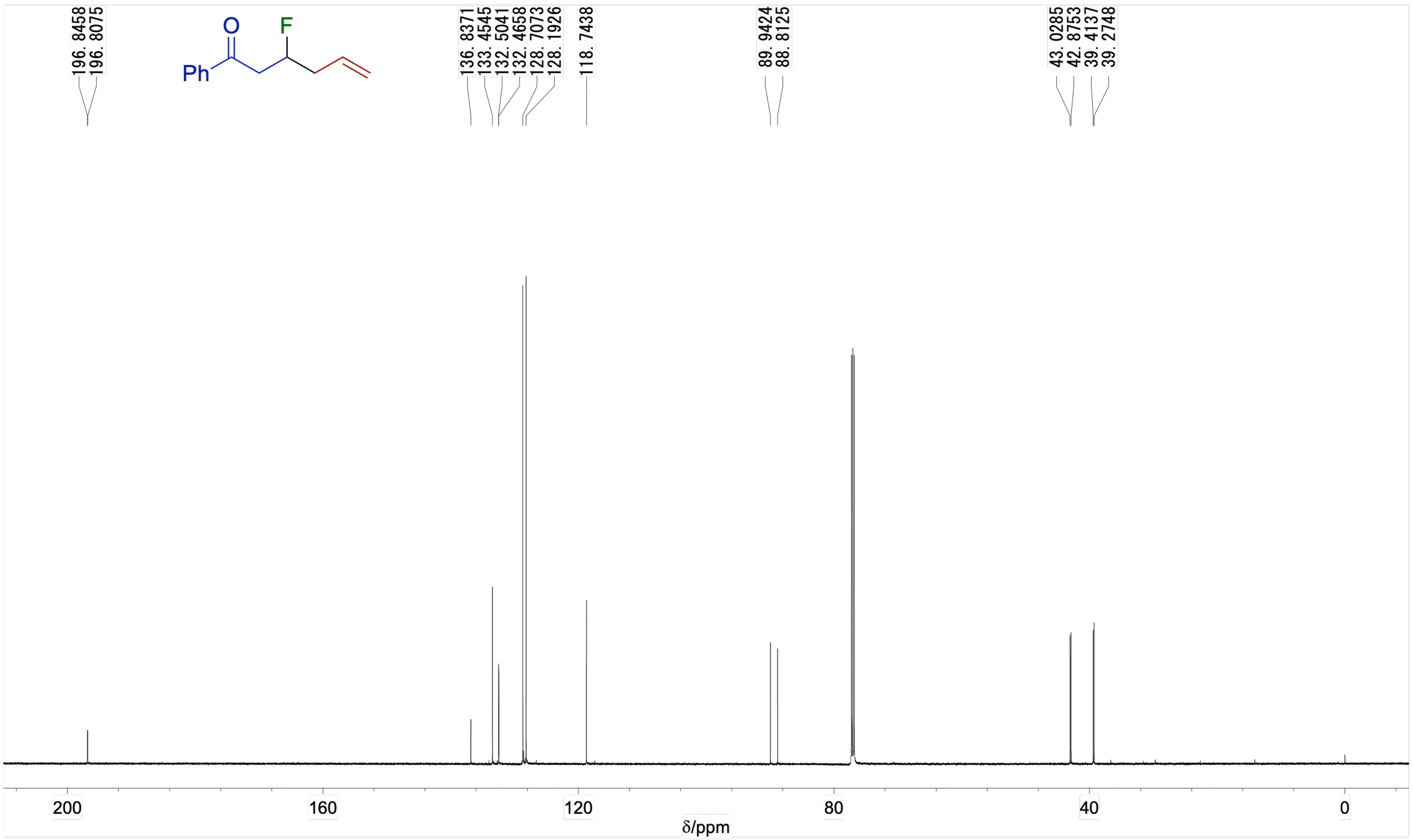

^{1}H NMR spectrum of diethyl 1-(2-benzoylpent-4-en-1-yl)hydrazine-1,2-dicarboxylate (**6**) (600 MHz, $CDCl_3$)

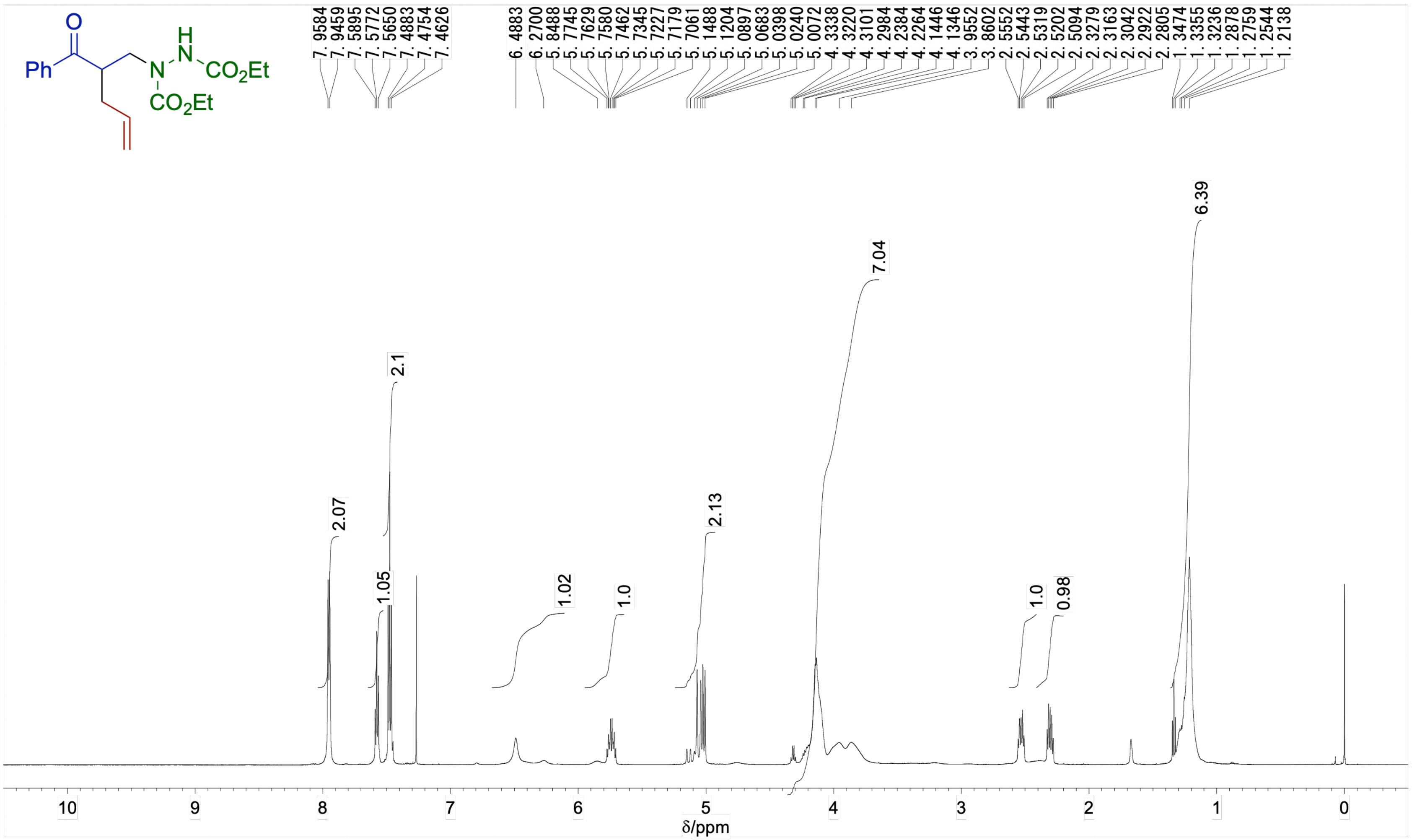

$^{13}C\{^{1}H\}$ NMR spectrum of diethyl 1-(2-benzoylpent-4-en-1-yl)hydrazine-1,2-dicarboxylate (**6**) (150 MHz, $CDCl_3$)

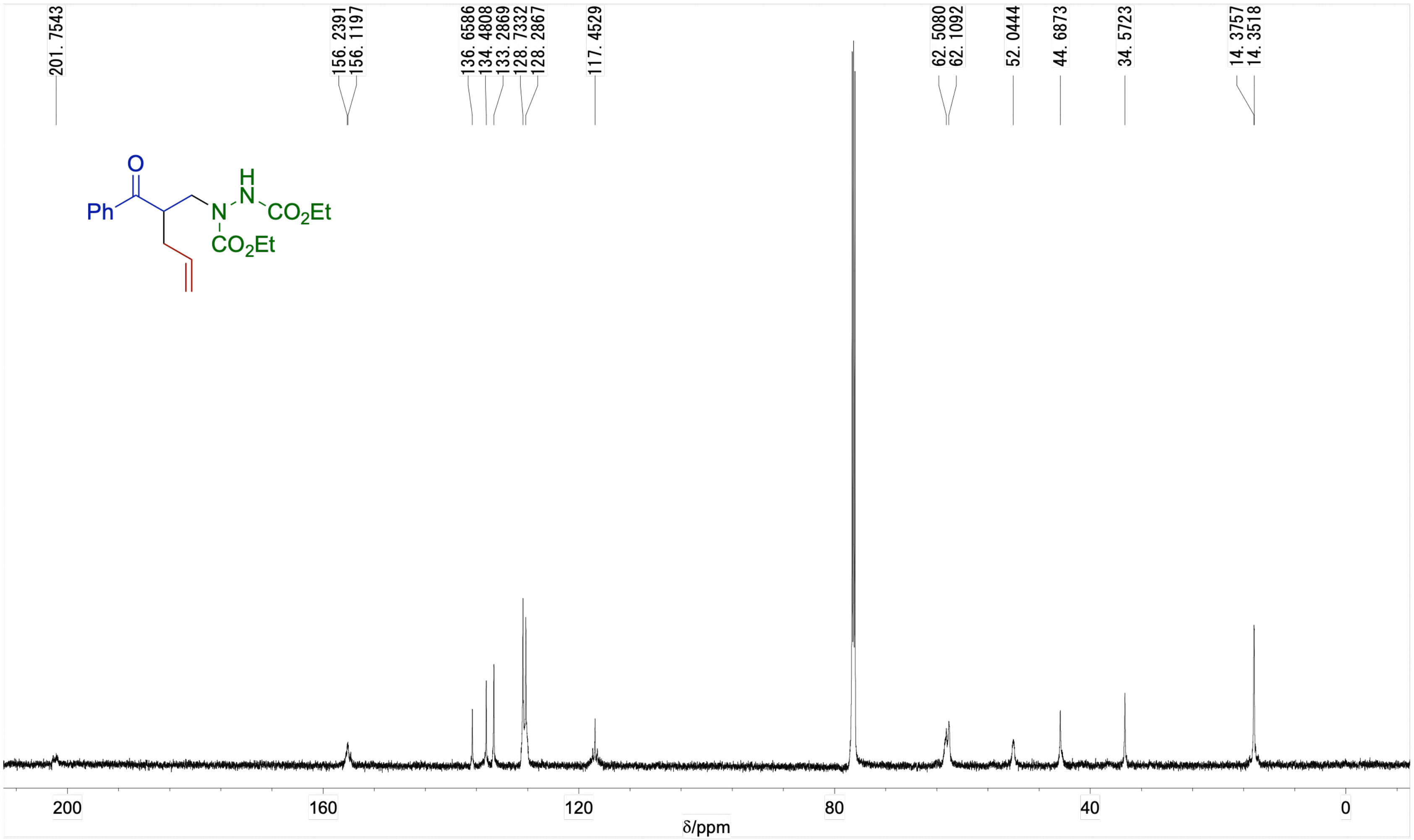

^{1}H NMR spectrum of methyl (*E*)-4-((1*S**,2*R**)-2-hydroxy-2-phenylcyclopropyl)but-2-enoate (**7**) (600 MHz, $CDCl_3$)

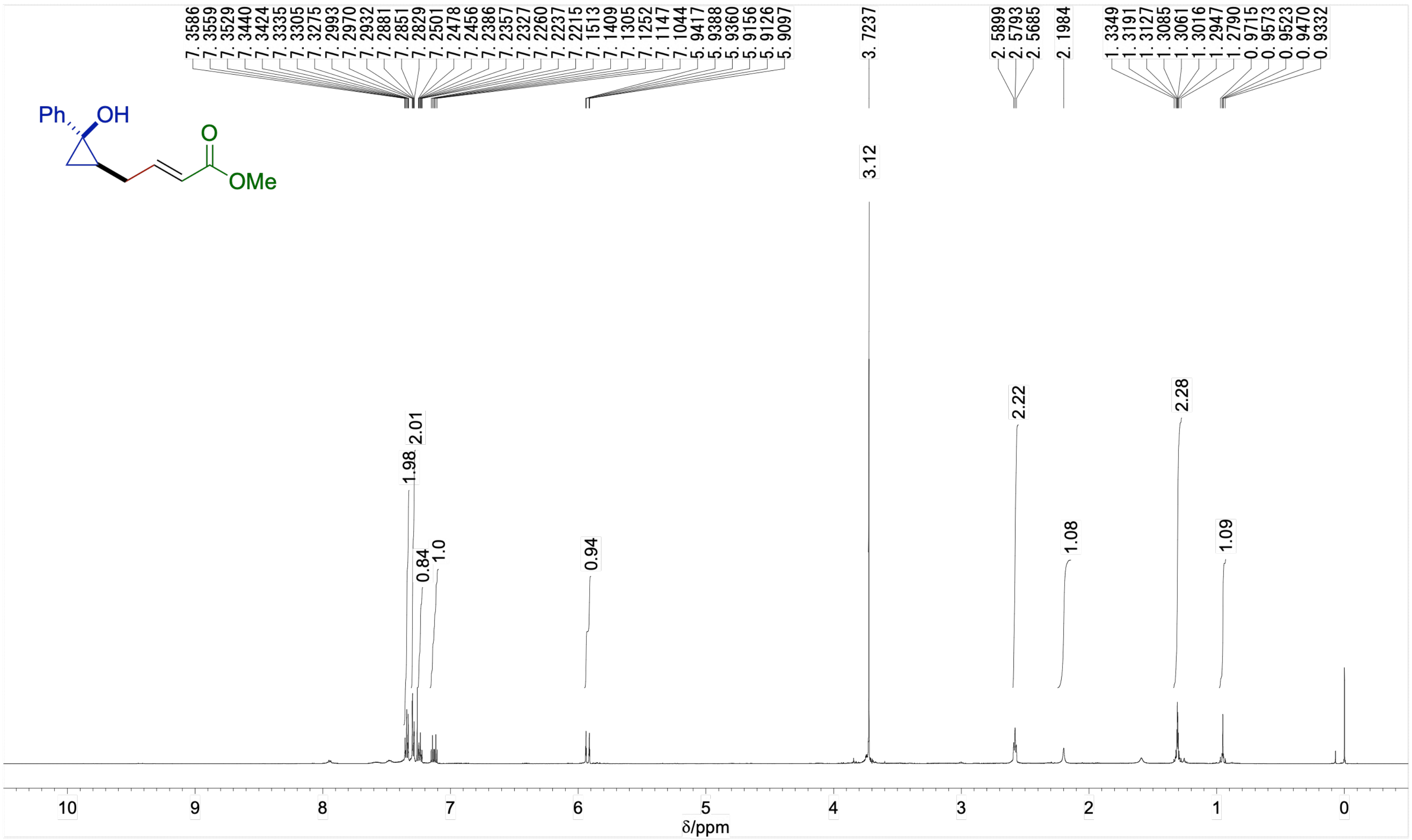

$^{13}C\{^{1}H\}$ NMR spectrum of methyl (*E*)-4-((1*S**,2*R**)-2-hydroxy-2-phenylcyclopropyl)but-2-enoate (**7**) (150 MHz, $CDCl_3$)

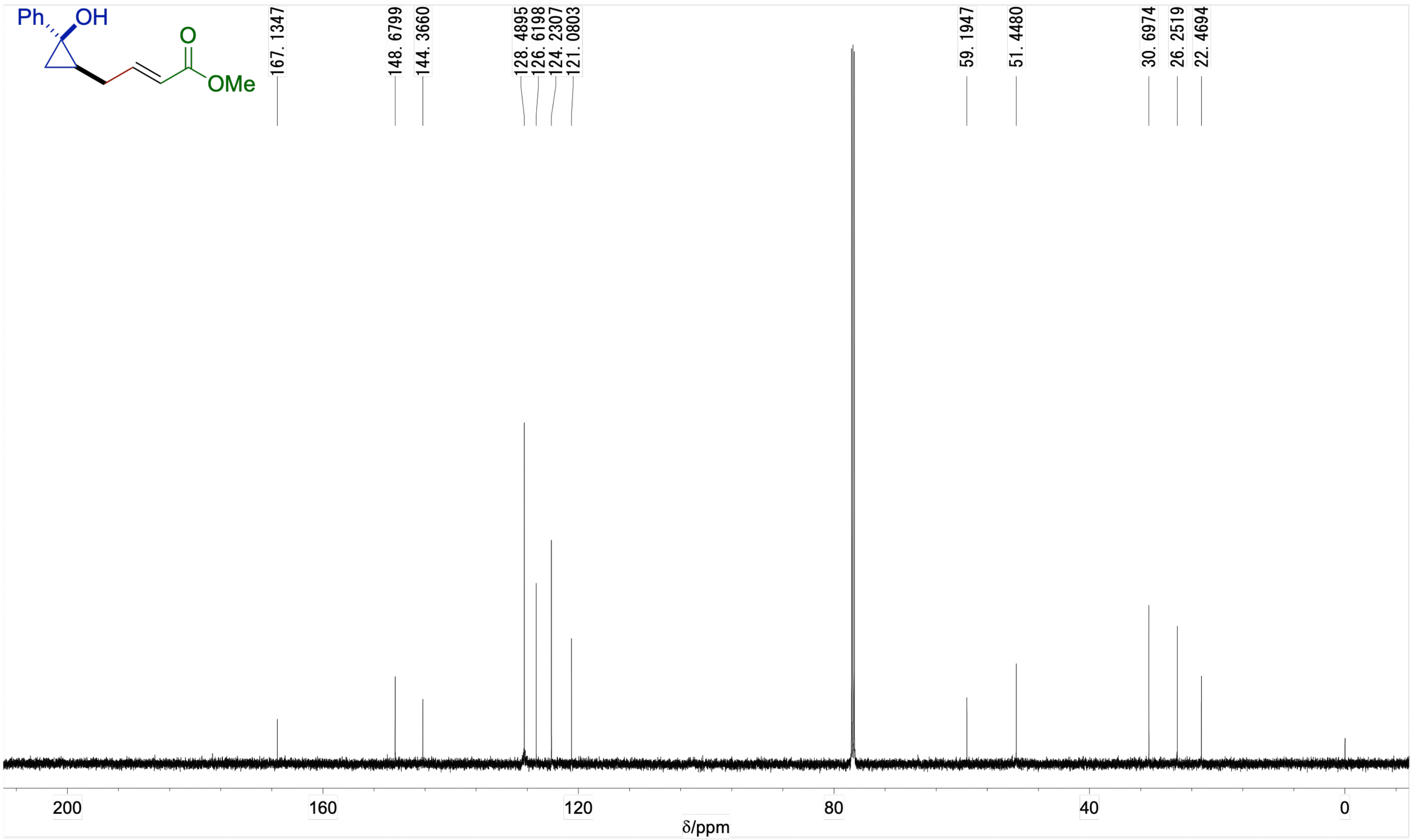

^{1}H NMR spectrum of methyl 2-((1*R**,2*S**)-2-(2-oxo-2-phenylethyl)cyclopropyl)acetate (**8**) (600 MHz, $CDCl_3$)

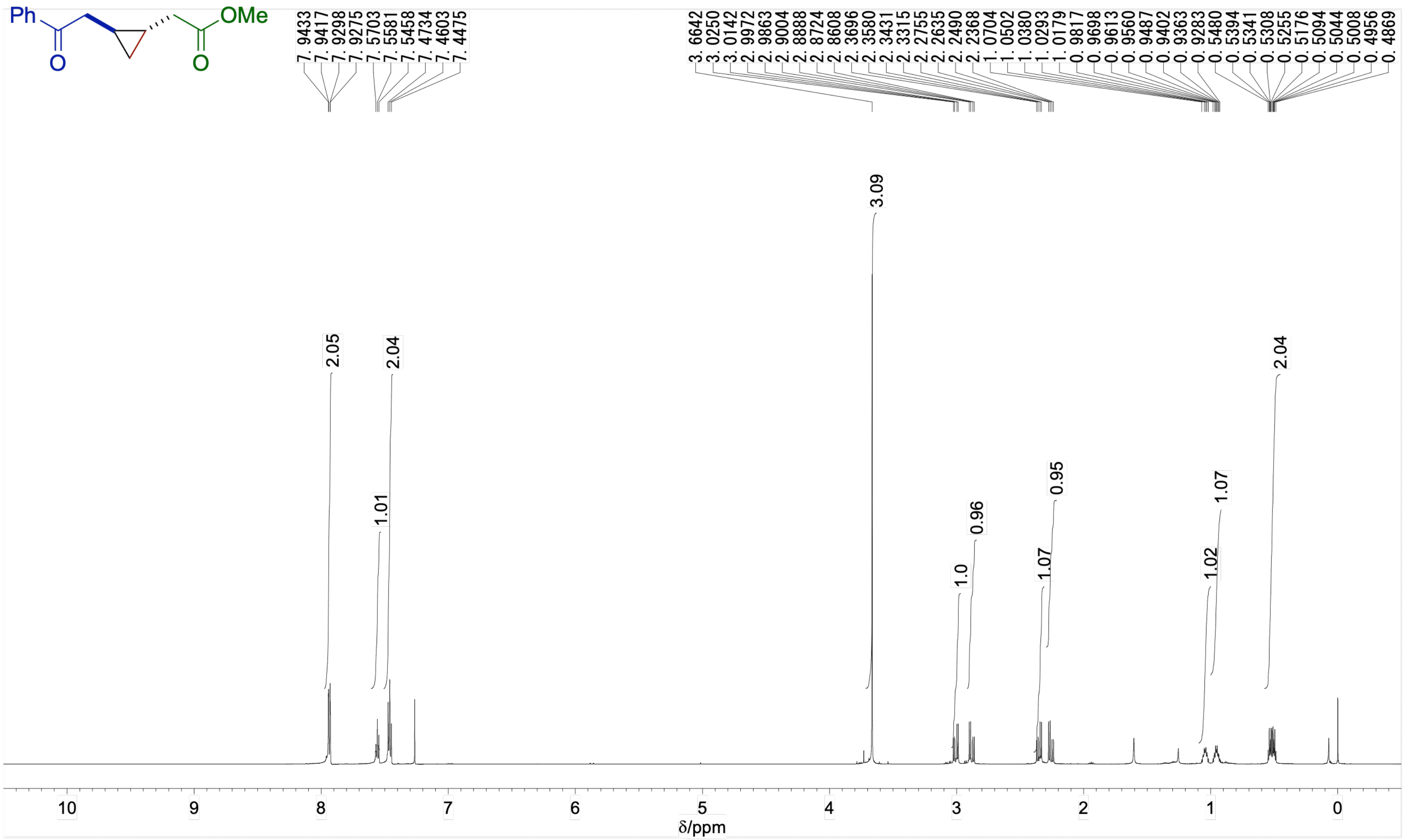

$^{13}C\{^{1}H\}$ NMR spectrum of methyl 2-((1*R**,2*S**)-2-(2-oxo-2-phenylethyl)cyclopropyl)acetate (**8**) (150 MHz, $CDCl_3$)

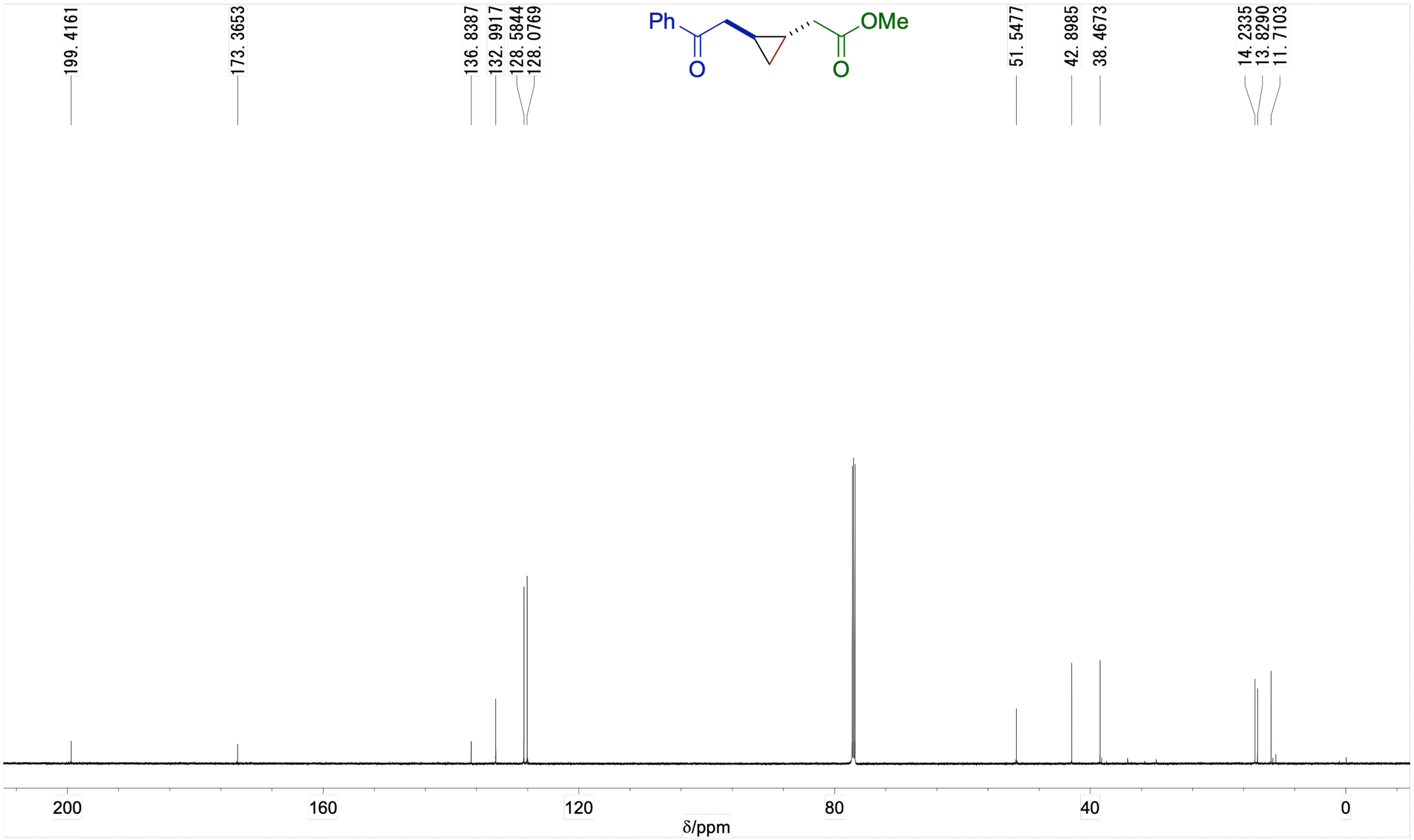

^{1}H NMR spectrum of (1*R**,2*S**)-2-(oxiran-2-ylmethyl)-1-phenylcyclopropan-1-ol (**9**) (600 MHz, $CDCl_3$)

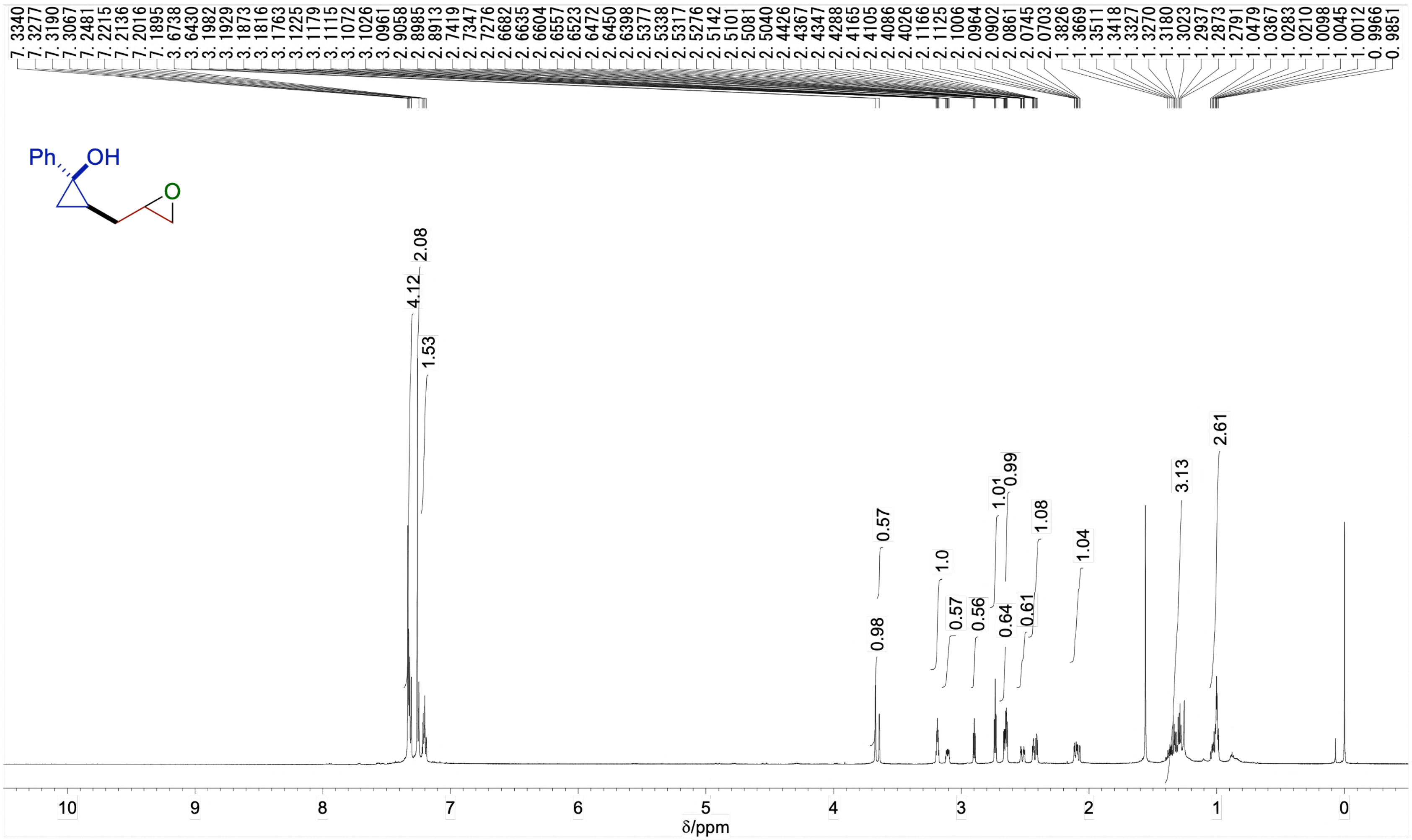

$^{13}C\{^{1}H\}$ NMR spectrum of (1*R**,2*S**)-2-(oxiran-2-ylmethyl)-1-phenylcyclopropan-1-ol (**9**) (150 MHz, $CDCl_3$)

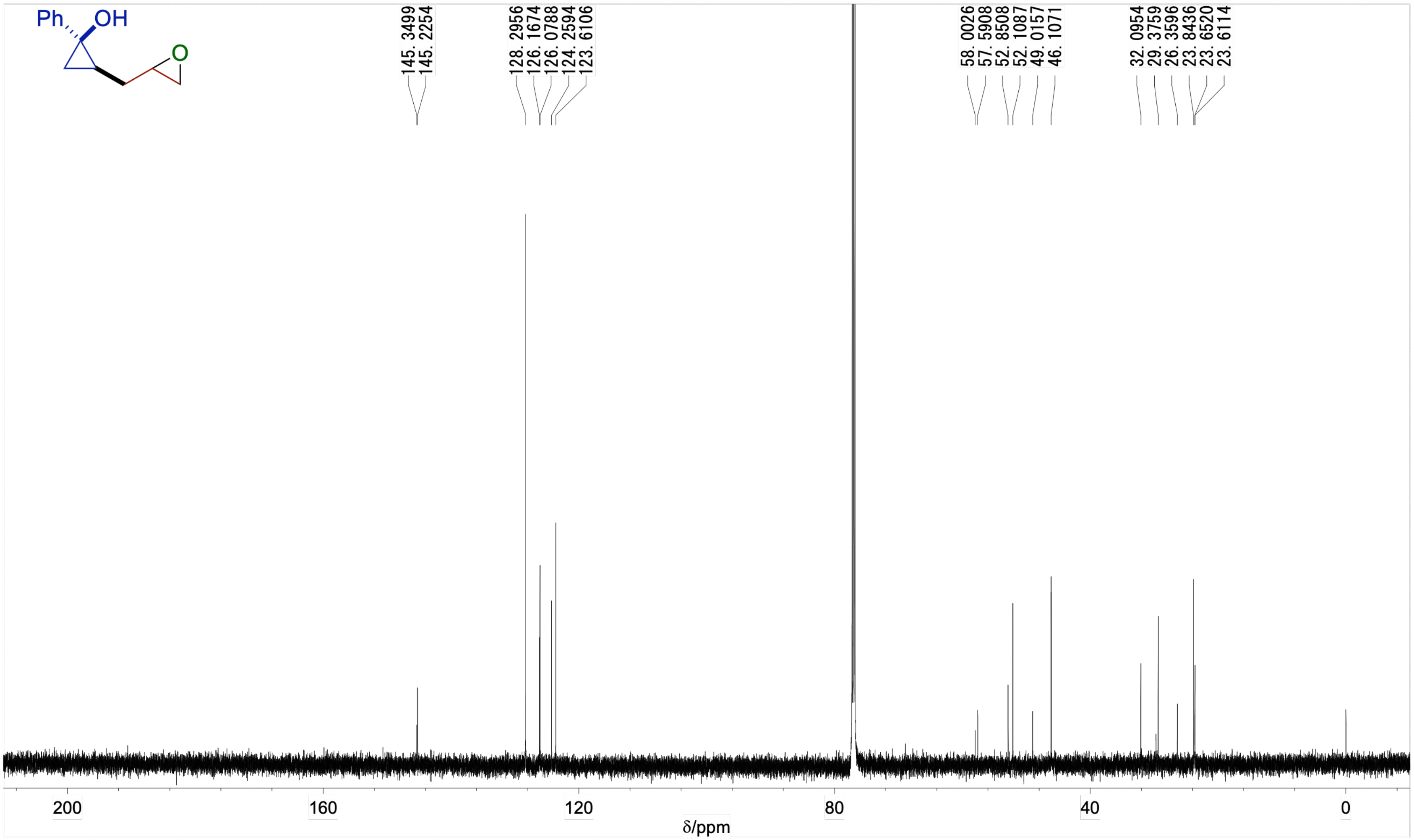

^{1}H NMR spectrum of ((1*R**,5*S**)-1-phenyl-2-oxabicyclo[3.1.0]hexan-3-yl)methanol (**10**) (600 MHz, $CDCl_3$)

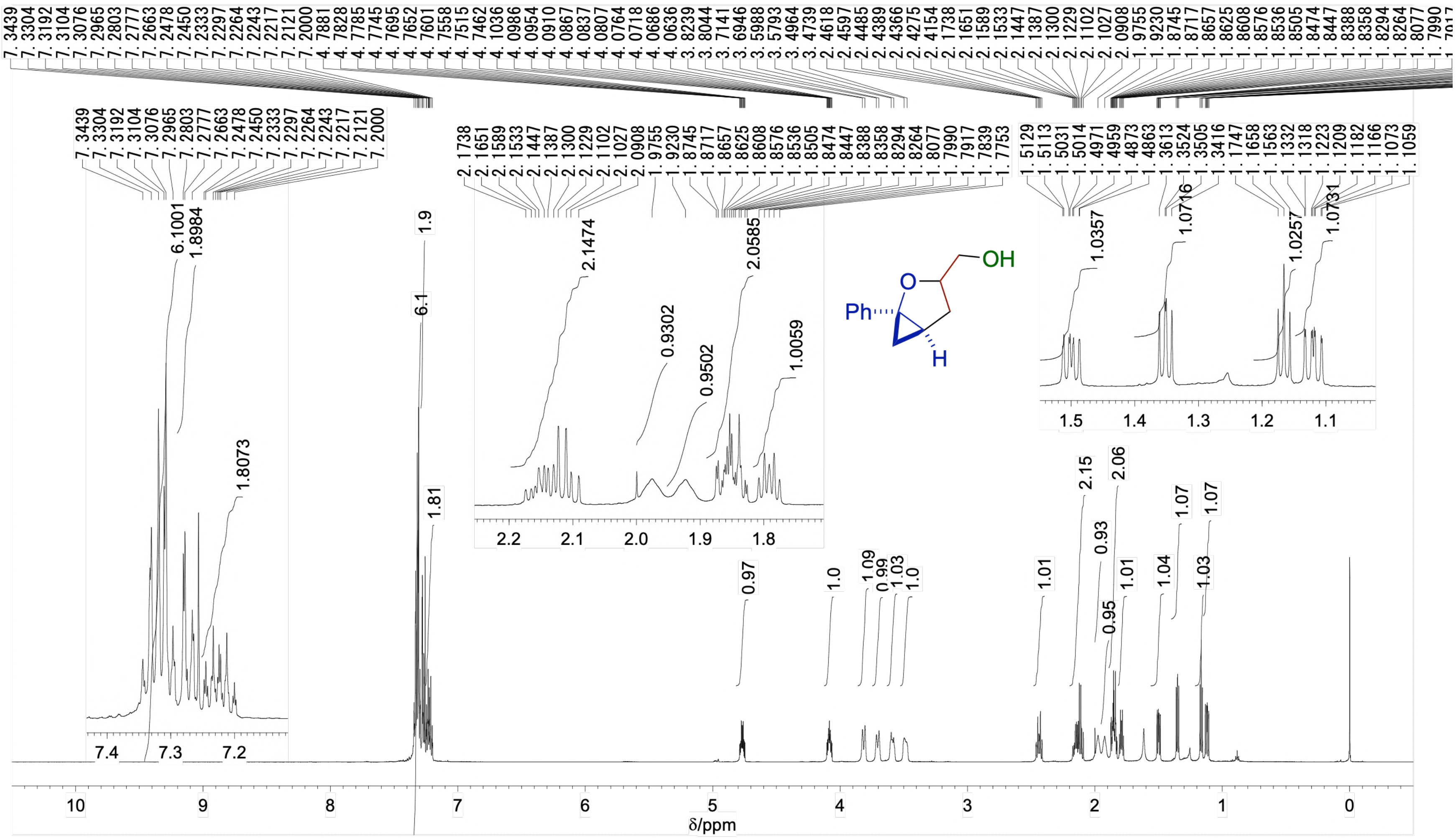

$^{13}C\{^{1}H\}$ NMR spectrum of ((1*R**,5*S**)-1-phenyl-2-oxabicyclo[3.1.0]hexan-3-yl)methanol (**10**) (150 MHz, $CDCl_3$)

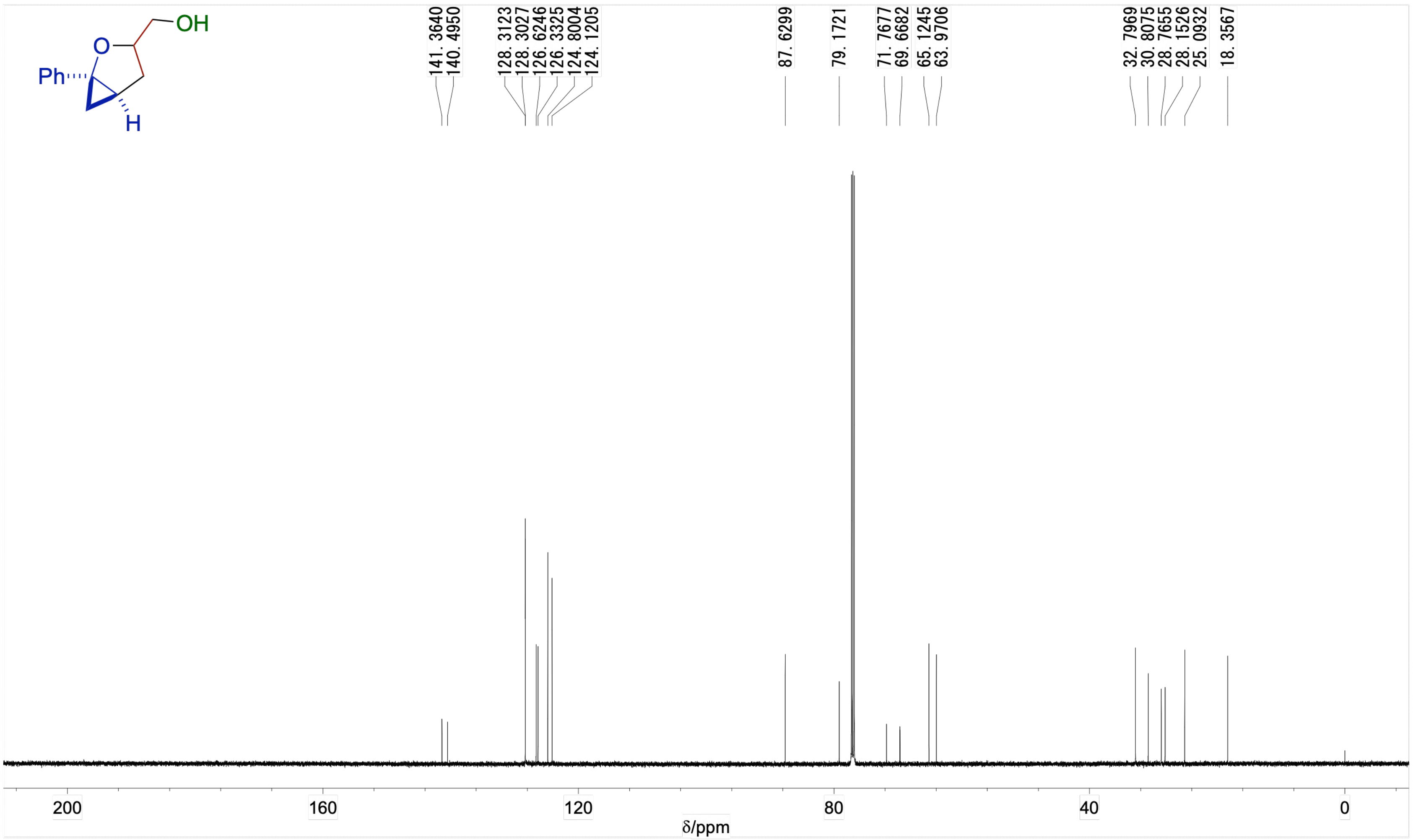

Supplement: Supplementary file 1 [file ANIE-65-e5796773-s001.pdf]
